# Supplementary material for: Qualitative study exploring lessons from Liberia and the UK for building a people-centred resilient health systems response to COVID-19
Source: BMJ Open. 2022 Aug 1;12(8):e058626. doi: 10.1136/bmjopen-2021-058626 (PMC9344595; doi:10.1136/bmjopen-2021-058626)
Supplement: Supplementary data [file bmjopen-2021-058626supp001.pdf]

BMJ Publishing Group Limited (BMJ) disclaims all liability and responsibility arising from any reliance placed on this supplemental material which has been supplied by the author(s)

Appendix 1: COVID-19 Key Informant Interview Topic Guides

Contents

Appendix 1: COVID-19 Key Informant Interview Topic Guides..1
Key Informant Interviews Topic Guide –MOH Liberia 2
Key Informant Interviews Topic Guide –Merseyside Regional Decision Makers 4
Key Informant Interviews Topic Guide – Health Workers 6
Key Informant Interviews Topic Guide –Merseyside Laboratory and Blood Transfusion Staff..........8

McCollum R, *et al. BMJ Open* 2022; 12:e058626. doi: 10.1136/bmjopen-2021-058626

Key Informant Interviews Topic Guide –MOH Liberia

All possible questions to be asked of key informants are described in the following guide. Prior to interviewing each stakeholder, specific guides for these individuals will be made. One interview that covers relevant research themes will be completed with each stakeholder. Ordering of questions will also be revised to ensure logical flow through the interview and to avoid repetition.

Background

Please can you tell me your position and how long you have worked in your current role?
How has your role changed due to the current COVID-19 crisis?

Responses to Shock and the General Health System

1. How do you think the health system has coped with the COVID-19 crisis? How did it compare with previous crisis? How have routine services been impacted?
2. How is the current shock (COVID-19) the health system is experiencing similar or different to those you have experienced before?
3. What are the key learnings from previous shocks (Ebola/ conflict/ economic crisis)? How are they being used to respond now?
4. How do you think routine health systems functions are being impacted by the current crisis (COVID19/economic)?
5. What do you think could be done to support continuation of routine services? How is this informed or shaped by learnings from during the Ebola period?
    a. How would you describe the quality of services usually? How is quality of care being maintained throughout the COVID-19 response?

6. <u>What policy or guidelines are supporting with the current COVID response? What additional guidelines or policies could be helpful for the COVID response?</u>

Service Specific Impacts

Questions in this section to be reviewed/modified for cross-cutting MOH functions, e.g. M&E, research division prior to starting interview

7. Can you tell me about how service delivery within your programme/section (adapt to include name of section depending on who talking too) has been affected by the COVID pandemic?
    a. Which of your services would you say have been most impacted so far? Why?
    b. Which services would you envisage will be most impacted moving forwards? Why?
8. How have your routine services been modified or adapted? Which components of your service do you view as essential? Why?
9. Which specific sub-populations is routine care most impacted for? Are there any marginalised groups who may struggle to use services since the onset of the COVID-19 crisis? (Probe: e.g gender, dis/ability, rural/urban; wealth; geographic regions; age etc)
10. Have there been any innovations within service delivery in response to the COVID-19 crisis, and have they been useful in any way?
11. Has there been any innovations in response to COVID-19 that have concerned you?

Human Resource Management

12. How have you planned for staffing to meet the changing additional workload in response to COVID? Any tools/ guidance from the human resource section? Successes and challenges? (Prompt for role of new community health cadres, for those providing face to face care and for MOH staff)
13. What additional skill development have you provided and how in response to COVID? Successes and challenges?
14. How are you able to support staff so they can continue to work effectively during the COVID pandemic
    a. How have you supported staff through communication?
    b. How have you supported staff for occupational safety including PPE?

COVID-19 Key Informant Interview Topic Guides

c. How have you supported staff through with psychosocial support?
d. What have been the successes and challenges with supporting staff?

Service and System Impacts: Governance and Decision Making

Questions in this section to be reviewed/modified to make these questions more service-specific, depending on the interviewee's programme area

15. How are decisions made about which services should or should not be prioritised as part of the COVID response? (prompt for in relation to their specific service and also in relation to general health system, prompt for donor influence)
16. How does decision-making as part of the COVID response influence routine planning activities? What has been the impact of resource re-distribution as part of the COVID response?
17. Who is involved in this decision making and what are the processes? What are the challenges?

18. <u>What do you think are the key ethical impacts of making these decisions? What ethical guidelines are currently in place and important in decision making during this period?</u>
19. <u>What guidance documents are available to support you in making decisions regarding COVID?</u>
20. <u>What guidance documents would help to support maintaining routine services?</u>

Closing Questions

21. What does a resilient health system look like to you? What are your three recommendations would you make to improve or maintain the resilience of the Liberian health system during this period?
22. What are your three recommendations would you make post crisis to ensure the return to routine function of the health system as effectively as possible?

Additional questions for Director of personnel only

23. What are the main sources of additional staffing (e.g. secondment/redeployment, task-shifting, improved productivity, early graduation/students, returnees, volunteers)? Successes and challenges? Optional: Impact on the wage bill?
24. What areas of service are now struggling with staffing?
25. What are you able to do to retain staff? Successes and challenges?
26. What impact did/is down-sizing of "non-essential staff' have on your programme during the crisis?

Thank-you
Any other comments?

Key Informant Interviews Topic Guide –Merseyside Regional Decision Makers

Version1.1_01052020

All possible questions to be asked of key informants are described in the following guide. Prior to interviewing each stakeholder, specific guides for these individuals will be made. One interview that covers relevant research themes will be completed with each stakeholder. Ordering of questions will also be revised to ensure logical flow through the interview and to avoid repetition.

Question List

Background

Please can you tell me your position and how long you have worked in your current role?

Impact of COVID 19 on Routine Service Delivery

1. What are defined as essential routine services?
2. Which are the main scheduled and unscheduled services affected by COVID-19 and how have these been adapted over time?
3. Have there been any innovations within service delivery, and what have these been?
4. Have there been any changes that have concerned you? Why?
5. What would help to support maintaining routine services?

Governance and Decision Making

6. What has informed your decision-making, such as guidance documents or governance decision-making processes?
7. Who is involved in decisions made about which services should or should not be prioritised?
8. How are decisions made about which services should or should not be prioritised?
9. Describe how and who is involved in operationalising decisions?
10. What challenges have you faced in making these decisions?
11. What are the main differences between various sites in the trust, especially between Aintree and the Royal Hospitals?
12. How are changes in service delivery communicated? How can this be improved? There are multiple guidelines at national and local levels, how are these disseminated? How well does this work? How rapidly? How do health care workers respond to these changes?

Human Resource Management

13. How have you [may be the employer in general] planned for staffing to meet the changing additional workload? Any tools/ guidance from national authorities? Successes and challenges?
14. How have you planned for the increase in staff absence?
15. What additional skill development have you provided and how? What have been the successes and challenges?
16. How are you able to support staff so they can continue to work effectively (e.g. communication, occupational safety including PPE, psychosocial support)? What have been the successes and challenges?

Recovery post COVID-19

17. Are there any COVID-19-related changes to routine health services that you think it would be useful to continue after COVID-19? Which ones and why?

18. What next steps do you believe should be taken now to support the health system to recover post COVID-19?

COVID-19 Key Informant Interview Topic Guides

Thank-you

Do you have any further suggestions for improvements to delivery of routine services?

Any other comments?

COVID-19 Key Informant Interview Topic Guides

Key Informant Interviews Topic Guide – Health Workers

Version1.1_01052020

All possible questions to be asked of key informants are described in the following guide. Prior to interviewing each stakeholder, specific guides for these individuals will be made. One interview that covers relevant research themes will be completed with each stakeholder. Ordering of questions will also be revised to ensure logical flow through the interview and to avoid repetition.

Question List

Background

Please can you tell me your usual position and how long you have worked in that role?
Are you currently working in your usual role and department?
If no, what role and department are you now working in?

Impact of COVID 19 on Routine Essential Service Delivery

1. Can you tell me about how health service delivery has been affected by the COVID pandemic? What was the processes for this, how was it communicated and do you have any ideas about how this can be improved? How prepared did you feel for these?
2. What do you consider to be routine essential health services in your work?
3. Which are the main scheduled and unscheduled services affected by COVID-19 in your department and how have these been adapted over time?
4. What have been the strengths and challenges with these changes? How has quality been affected?
5. How should these changes be evaluated? What indicators should be used?
6. What is worrying you most about your service now?
7. Which services would you envisage will be most impacted moving forwards as the pandemic progresses? (e.g. hospital based, community care, disease specific services, etc) Why?
8. Who do you think are the people most impacted by the changes in routine service delivery? Would you say that patients with specific socio-demographic characteristics are more impacted by service disruption/ distortion than others? Why? (e.g. gender, dis/ability; rural/urban; wealth; geographic regions; age etc) What can be done to ensure that these patients can still use health services when they need them?

Ethics and Decision Making

9. Have you encountered any health systems issues which you found troubling since the start of the COVID-19 pandemic? Would you be willing to tell me more about these issues?
10. What is the impact of these issues on you as a health worker? What would be helpful to support you in dealing with these issues?
11. Do you know of any ethical guidelines in place to guide you as you make difficult decisions during this time? What are these? How are these ethical guidelines operationalised? Are they useful?
12. Have you been involved with making decisions about the changes to health services since the COVID-19 pandemic? What was your role in making these decisions? How were these decisions made?
13. When there are changes in how health services are delivered how are these communicated with you? How has this worked? What do you think is the best way to be informed?

Human Resource Management

14. How has your role changed since the start of the COVID-19 pandemic? What have been the successes and challenges with how your role has changed? Probe workload
15. Is there anything about your role that concerns you? What?
    a. Probe working outside are of expertise
    b. No indemnity if make an error
    c. Communication about working across disciplines

16. What preparation for the changes to your role have you had and how was it delivered (skills - key ones, psychological support)? What have been the successes and challenges?
    a. Probe PPE training
    b. COVID clinical training
    c. Support mechanisms
    d. Team formation
17. What kind of support (e.g. communication, occupational safety including PPE, psychosocial support) are you receiving to do your job from your team/manager/employer? What have been the successes and challenges?

Recovery post COVID-19

18 . Are there any COVID-19-related changes or innovations to routine health services that you think it would be useful to continue after COVID-19? Which ones and why?

19. What next steps do you believe should be taken now to support the health system to recover post COVID-19?

20. What is worrying you most as the response moves forward?

Thank-you

Do you have any further suggestions for improvements to delivery of routine services?

Any other comments?

Key Informant Interviews Topic Guide –Merseyside Laboratory and Blood Transfusion Staff

Version1.1_01052020
All possible questions to be asked of key informants are described in the following guide. Prior to interviewing each stakeholder, specific guides for these individuals will be made. One interview that covers relevant research themes will be completed with each stakeholder. Ordering of questions will also be revised to ensure logical flow through the interview and to avoid repetition.

Background
Please can you tell me your position and how long you have worked in your current role?

Governance and Decision Making - Relating Directly to COVID-19
1. What has been the decision-making process for the laboratory's response to COVID-19 testing services and when did discussions start around re-adjusting services for COVID-19?
2. Who held overall responsibility for how COVID-19 testing was going to be conducted at LCL?
3. In addition to PHE, have the Liverpool Clinical Laboratory services worked closely/ collaborated with any other external partners for COVID-19 testing? If so whom and in what capacity?

Governance and Decision Making - Relating to Maintaining Routine Service Delivery
4. How are decisions made about which services should or should not be prioritised; which ones were considered to be essential and why? Who is involved in this decision making? How were these decisions communicated?
5. What guidance documents were most useful to you in making these decisions? In what way were they useful?
6. What key challenges have you faced in making these decisions? Do you have any support needs here?

Impact of COVID-19 on Routine Laboratory Service Delivery
7. Can you tell me about how routine clinical laboratory service delivery has been affected by the COVID pandemic?
COVID-19 Testing service specific
8. How did the laboratories adapt to scale up COVID-19 testing? (analysers, staff capacity, staff training, standard operating procedures, risk assessments)
9. What challenges did the laboratory face when implementing COVID-19 testing? How were they overcome? What worked well? (e.g. resources, human resource, process change, governance, culture, leadership etc)
10. Which routine services would you envisage will be most impacted moving forwards? (e.g. hospital based-testing, disease specific services, etc) Why?

Recovery post COVID-19
11. Are there any COVID-19-related changes to the laboratory service that you think it would be useful to continue after COVID-19? Which ones and why?
12. What next steps do you believe should be taken now to support the laboratory system to recover post COVID-19?
13. Are there any changes/ innovations introduced in response to COVID-19 changes which you think should be continued? Why?

Thank you
Do you have any questions for me? Resources (re labs) link https://www.rcpath.org/uploads/assets/90111431-8aca-4614-b06633d07e2a3dd9/Guidance-and-SOP-COVID-19-Testing-NHS-Laboratories.pdf

COVID-19 Key Informant Interview Topic Guides
