# Supplementary material for: Qualitative study exploring lessons from Liberia and the UK for building a people-centred resilient health systems response to COVID-19
Source: BMJ Open. 2022 Aug 1;12(8):e058626. doi: 10.1136/bmjopen-2021-058626 (PMC9344595; doi:10.1136/bmjopen-2021-058626)
Supplement: Author's manuscript [file bmjopen-2021-058626.draft_revisions.pdf]

BMJ Open

BMJ Open is committed to open peer review. As part of this commitment we make the peer review history of every article we publish publicly available.

When an article is published we post the peer reviewers' comments and the authors' responses online. We also post the versions of the paper that were used during peer review. These are the versions that the peer review comments apply to.

The versions of the paper that follow are the versions that were submitted during the peer review process. They are not the versions of record or the final published versions. They should not be cited or distributed as the published version of this manuscript.

BMJ Open is an open access journal and the full, final, typeset and author-corrected version of record of the manuscript is available on our site with no access controls, subscription charges or pay-per-view fees (http://bmjopen.bmj.com).

If you have any questions on BMJ Open's open peer review process please email info.bmjopen@bmj.com

BMJ Open

Shared lessons from Liberia and the UK for building a people-centred resilient health systems response to COVID-19

Journal:	*BMJ Open*
Manuscript ID	bmjopen-2021-058626
Article Type:	Original research
Date Submitted by the Author:	27-Oct-2021
Complete List of Authors:	McCollum, Rosalind ; Liverpool School of Tropical Medicine, International Public Health<br>Zaizay, Zeela ; Actions Transforming Lives<br>Dean, Laura; Liverpool School of Tropical Medicine, Department of International Public Health<br>Watson, Victoria; Liverpool School of Tropical Medicine (LSTM), International Public Health<br>Frith, Lucy; The University of Manchester, Centre for Social Ethics & Policy, School of Law<br>Alhassan, Yussif; Liverpool School of Tropical Medicine, International Public Health<br>Kollie, Karsor; Ministry of Health<br>Piotrowski, Helen; Liverpool School of Tropical Medicine, Department of International Public Health<br>Bates, Imelda; Liverpool School of Tropical Medicine<br>Anderson De Cuevas, Rachel; University of Liverpool, Institute of Population Health<br>Harris, Rebecca ; University of Liverpool, Institute of Population Health<br>Chowdhury, Shahreen; Liverpool School of Tropical Medicine, International Public Health<br>Berrian, Hannah; University of Liberia-Pacific Institute for Research and Evaluation<br>Smith, John; University of Liberia-Pacific Institute for Research and Evaluation<br>Tate, Wede; University of Liberia-Pacific Institute for Research and Evaluation<br>El Hajj, Taghreed; Liverpool School of Tropical Medicine<br>Ozano, Kim; Liverpool School of Tropical Medicine<br>Hastie, Olivia; Liverpool School of Tropical Medicine<br>Parker, Colleen; Ministry of Health Liberia<br>Kollie, Jerry; University of Liberia-Pacific Institute for Research and Evaluation<br>Zawolo, Georgina; University of Liberia-Pacific Institute for Research and Evaluation<br>Ding, Yan; Liverpool School of Tropical Medicine, Centre for Capacity Research<br>Dacombe, Russell; Liverpool School of Tropical Medicine, International Public Health<br>Taegtmeyer, Miriam; Liverpool School of Tropical Medicine, International

Public Health
Theobald, Sally ; Liverpool School of Tropical Medicine, International Public Health

Keywords: COVID-19, HEALTH SERVICES ADMINISTRATION & MANAGEMENT, PUBLIC HEALTH, QUALITATIVE RESEARCH

SCHOLARONE™ Manuscripts

I, the Submitting Author has the right to grant and does grant on behalf of all authors of the Work (as defined in the below author licence), an exclusive licence and/or a non-exclusive licence for contributions from authors who are: i) UK Crown employees; ii) where BMJ has agreed a CC-BY licence shall apply, and/or iii) in accordance with the terms applicable for US Federal Government officers or employees acting as part of their official duties; on a worldwide, perpetual, irrevocable, royalty-free basis to BMJ Publishing Group Ltd ("BMJ") its licensees and where the relevant Journal is co-owned by BMJ to the co-owners of the Journal, to publish the Work in this journal and any other BMJ products and to exploit all rights, as set out in our licence.

The Submitting Author accepts and understands that any supply made under these terms is made by BMJ to the Submitting Author unless you are acting as an employee on behalf of your employer or a postgraduate student of an affiliated institution which is paying any applicable article publishing charge ("APC") for Open Access articles. Where the Submitting Author wishes to make the Work available on an Open Access basis (and intends to pay the relevant APC), the terms of reuse of such Open Access shall be governed by a Creative Commons licence – details of these licences and which Creative Commons licence will apply to this Work are set out in our licence referred to above.

Other than as permitted in any relevant BMJ Author's Self Archiving Policies, I confirm this Work has not been accepted for publication elsewhere, is not being considered for publication elsewhere and does not duplicate material already published. I confirm all authors consent to publication of this Work and authorise the granting of this licence.

For peer review only

Title: Shared lessons from Liberia and the UK for building a people-centred resilient health systems response to COVID-19

Authors: Rosalind McCollum*^, Zeela Zaizay*, Laura Dean, Victoria Watson, Lucy Frith, Yussif Alhassan, Karsor Kollie, Helen Piotrowski, Imelda Bates, Rachel Anderson De Cuevas, Rebecca Harris, Shahreen Chowdhury, Hannah Berrian, John Smith, Wede Tate, Taghreed El Hajj, Kim Ozano, Olivia Hastie, Colleen Parker, Jerry Kollie, Georgina Zawolo, Yan Ding, Russell Dacombe, Miriam Taegtmeyer, Sally Theobald

*Joint first authors

^Corresponding author email address: rosalindmccollum@gmail.com

For peer review only

Abstract

Introduction: COVID-19 has tested the resilience of health systems globally and exposed existing strengths and weaknesses. This study uses the concept of people-centred health systems to explore the applicability of the Foreign, Commonwealth and Development Office (FCDO) principles for health systems' resilience in two contrasting contexts – Liberia and the UK.

Methods: We carried out qualitative interviews with 24 health decision-makers at National and County Level in Liberia and 42 health actors at County and hospital level in the UK (Merseyside). We explored these health systems' decision-making processes and capacity to adapt and continue essential service delivery in response to COVID-19 in both contexts.

Results: Study respondents in Liberia and Merseyside had similar experiences in responding to COVID-19, despite significant differences in health systems context, and there is an opportunity for multi-directional learning between health systems in the global south and global north. The need for early preparedness; strong community engagement; clear communication within the health system, and health service delivery adaptations for essential health services emerged strongly in both settings. We found the FCDO principles valuable for reviewing health systems changes in response to a shock such as a natural disaster or pandemic, and based on our findings we identified two additional principles; 1) the need for functional structures and mechanisms for preparation and 2) adaptable governance and leadership structures to facilitate timely decision-making and response coordination. We find a people-centred approach can help ensure service adaptations are acceptable to, and understood by, patients and health workers, and continue the provision of 'routine services' for individuals during health systems shocks.

Conclusion: Our study highlights the importance of a people-centred approach, which places the person at the centre of study and analysis of the health system, and value in applying the FCDO principles across diverse settings.

For peer review only

Strengths and Limitations of the Study

- A key strength of this study is the multi-directional learning between health systems in the global south and global north, which involved a wide range of researchers across both settings, and the breadth of perspectives captured from frontline staff and key decision-makers.
- We find that the FCDO principles can be usefully applied across diverse contexts, with identification of two additional new principles, related to mechanisms for advanced preparedness and adaptable governance and leadership structures.
- The greatest limitation of this study is that it was carried out at a single point in time, towards the end of the first wave in the UK and before there had been a large increase in cases in Liberia. Response measures have evolved in both settings in subsequent stages of the pandemic.
- The study was limited by the differing range of respondents across study settings, with participants from across a range of health system levels including primary care, hospital frontline workers and decision-makers as well as regional decision-makers within Merseyside, UK; compared with national and county level decision-makers, technicians and supervisors of frontline staff in Liberia, which may result in differing perspectives.

For peer review only

Introduction

The COVID-19 pandemic has forever altered our world. It's impact has been felt across all nations, demonstrating the importance of resilient health systems in protecting global health security.[1] Health systems have been forced to adapt to new ways of working alongside the continued provision of essential services including: prevention of communicable diseases; sexual and reproductive health; care for vulnerable populations; ongoing management of chronic illness (including mental health conditions); continuity of critical inpatient therapies; management of emergency health conditions; and auxiliary services, including diagnostic imaging, laboratory and transfusion services.[2]

In April 2020, the United Nations expressed concern that, within Africa, up to 3.3 million people could lose their lives as a direct result of COVID-19 and many more through the indirect effects of disruption to health services and worsening socioeconomic conditions.[3] Conditions considered to increase the risk of infection include overcrowded and poorly serviced slum dwellings; limited access to basic handwashing facilities; high levels of informal employment limiting ability to work from home; high levels of malnutrition and lower ratios of beds and health workers to the population.[3] A commentary published by Agyeman et al. (2020) at the outset of the pandemic highlighted a rapid response within many African settings, including focus on early introduction of screening procedures at ports of entry, need for effective community engagement to educate about the mode of transmission. Key protective behaviours were emphasised, along with the need to prepare intensive care beds and clear government strategies regarding how to deal with hospitalised COVID-19 patients to avoid disrupting the health system and to prevent non-COVID-19 related deaths.[4] Subsequent studies have revealed that indirect health impacts from COVID-19 disproportionately impact women and children.[5,6] Diversion of resources (financial, material, human) from existing health services to address the pandemic, impacts their care.[5,6] This includes supply and demand side disruptions that can result in lower utilization of health care and, in some cases, impact on quality of care.[7] Bayani et al (2021) surmise that "less

For peer review only

health care will result in more ill health and deaths because health services have been suspended, displaced, or inaccessible."(page 5 [7])

Our study was carried out immediately following the first wave of COVID-19 in Liberia and UK (interviews carried out June to September 2020) in response to an expressed need by stakeholders for this research following dialogue in both contexts. The pandemic has continued to evolve across both settings, with both Liberia and UK experiencing much larger waves of COVID-19 since this original study was carried out. These findings from the first wave can provide valuable lessons to inform continued response to COVID-19 and other health systems shocks.

The pandemic has revealed monopolies of knowledge production, which disempower lower and middle-income countries;[8] whilst pandemic responses in 'developed democracies' have been inadequate, with cuts to health and social services and limited commitment to equity or governance.[8] So-called "global powerhouses with tried and tested health systems have struggled to contain the COVID-19 pandemic"[9] and health systems have been stretched to the limit, resulting in negative implications for the health of all populations, particularly when access for patients with other acute and chronic illness is limited.[8] As of 01/09/21, UK (population 67.2 million)[10] has 6,821,356 confirmed cases and 132,859 COVID-19 related deaths.[11] In the UK, the National Health Service delivers care for most of the population. Meanwhile during the same time period, Liberia (population 5.1 million)[10] has had 5594 confirmed cases, with 245 confirmed COVID-19 related deaths.[11] Liberia was initially hailed as one of the top countries in fighting COVID-19, being one of the first countries to start screening at ports of entry (January 2020) and to adopt other control measures such as rapid testing, contact tracing and quarantine.[12,13]

"Improving resilience within health systems can build on pre-existing strengths to enhance the readiness of health system actors to respond to crises, while also maintaining core functions."(page 1 [1]). People-centred health systems are a critical framing in shaping resilience as they place people and communities at the centre whilst also promoting strategic and collaborative multi-sectoral

For peer review only

leadership which is necessary in delivering a co-ordinated response to a public health crisis.[14] In this paper, we compare health systems responses at a single point in time (June to September 2020) within Monrovia, Liberia and Merseyside, UK to distil lessons for health systems resilience to a pandemic through comparative case studies which explore aspects of health systems resilience.[15] Both contexts have a commitment to the development of people-centred health systems, and so within this paper we combine the Foreign, Commonwealth and Development Office (FCDO) eight key principles for promoting resilient health systems with key domains and values of people-centred health systems to frame our findings in relation to the COVID-19 response.[16] Through our discussion we reflect on these principles against our conceptual framework (figure 1), which is based on a people-centred approach. In response to a recent call for on-the-ground analysis of the response to COVID-19 within the Global South and comparative case studies that use co-creation and coproduction approaches which go beyond researchers including policy makers, practitioners and the public,[15,17] we seek to share learning from the response within Liberia and the UK, along with opportunities for multi-directional knowledge sharing.[17] It is our hope that this paper will help inform health policy makers across global contexts, for the current pandemic response and as they plan towards more resilient people-centred health systems to meet future shocks.

Methods

<u>Study context</u>

Liberia and UK have had very different strategies and case rates from the outset of the pandemic, although there were some similarities in the adoption of infection prevention control measures across both contexts. Liberia is amongst the world's poorest in terms of GDP and living conditions. According to the World Bank 2016 poverty headcount ratio, 44.4% of Liberians live below the international poverty benchmark of $1.90 USD per day.[18] The UNDP Human Development Report 2020 ranks Liberia low at 175 out of 189 countries and territories.[19] Inequities between females and males are remarkable with literacy rates (secondary education) of 18.5% and 40.1% respectively.[19] Liberia has

For peer review only

prior experiences of shocks in the form of two civil wars, and the 2014-2015 Ebola Virus Disease (EVD) epidemic. In response to these experiences, Liberia has prioritised rebuilding a resilient health system, which acknowledges the critical role communities play in addressing their own health needs through the 'Investment Plan for Building a Resilient Health System in Liberia' and the community health services policy (2016-2021).[20,21] By contrast, Merseyside is a Metropolitan County in the North West of England, comprising five boroughs, including the City of Liverpool, including some of the most deprived council areas in England.[22] It has a population of 1.42 million and has had some of the highest numbers of COVID-19 cases in the UK.[23] Within Merseyside, the Liverpool City Region Combined Authority has prioritised tackling deprivation and reducing health inequalities through people-centred care, with integration of health and social care services.[24] Liverpool has a long history of public health innovation, but also a strong sense of local history, culture and place. Throughout the pandemic Liverpool has been at the forefront of community-based innovations and public health strategies, e.g. piloting community open access testing for COVID-19.[25]

Liberia introduced stringent border control measures from January 2020, with the establishment of a Special Presidential Advisory Committee on Coronavirus (SPACOC) over two months prior to the first recorded cases in the country.[26],[27] Liberia's response to COVID-19, prioritised a call to maintain the delivery of routine health services at all levels. Hospitals and clinics continued to provide health services with health facility workers trained in infection prevention control (IPC) before the first case was identified in country.[27] Physical distancing measures were introduced and use of face masks encouraged.[28]

Within the UK, health service delivery was restructured as part of the COVID-19 response, with routine non-urgent elective care suspended and later re-started in April 2020.[29] Adaptations to minimise potential risk of COVID-19 infection include the use of telemedicine and phone consultations; and changes to essential services for patients, such as changed treatment plans and delays to surgeries.[30] Hospital patient pathways were altered to appropriately triage and cohort the care of COVID-19 patients,

For peer review only

reducing the risk of transmission to others and allowing essential services to continue. There was also reduction in routine blood test screening to prioritize COVID-19 PCR testing in response to the UKs 'test and trace' strategy.

Study aim, design and conceptual framework

Aim: To understand COVID-19 adaptations and decision-making in Liberia and Merseyside, UK

This qualitative study explored inductively the differing experiences, perspectives and recommendations of participants in order to understand COVID-19 adaptations and decision-making in Liberia and Merseyside, UK.[31,32] We selected qualitative methods to give "due emphasis to the meanings, experiences, and views of all the participants"(page 43 [31]) and understand decision-making and the impact of health systems adaptations as a result of COVID-19.

A conceptual framework was jointly developed, following a series of meetings held with researchers in each setting (7 Liberia-based researchers and 18 UK-based researchers). This framework sought to consider a people-centred approach towards the health system's ability to respond to shock, whilst reflecting the realities experienced in the face of multiple routine challenges (Figure 1).[33] The nature of a shock to the health system, whether due to infectious disease outbreak, natural disaster, or conflict, influences the rest of the framework.[34] It adopts a people-centred approach at it's heart,[14,35,36] while incorporating literature relating to the health system's ability to respond to a sudden shock, and the extent to which it is able to absorb, adapt and transform in response (Figure 1).[34,37–41]

People-centred health systems prioritise the collective right to health through integrated and targeted approaches that favour the needs of the most vulnerable.[14,42] Collective action and social solidarity are viewed as essential to the art and science of the development of people centred systems that are organised around people's health care needs and expectations as opposed to diseases, ensuring a continuum of care throughout the life course.[14] This approach embraces the human character of

For peer review only

health systems, by viewing individuals, communities and health workers as co-producers of health care, placing people and families at the centre.[43] Systems must adapt to meet a range of challenges to support the development of strategies that seek to improve health care access and encourage universal coverage. This is particularly important as many individuals transition and oscillate between multiple roles of patient, family and sometimes health care provider within one system.

Interview topic guides were informed by the framework and developed across both settings to explore key areas of health systems functioning in response to COVID-19 (Appendix 1). Questions included: governance and decision-making; use of ethical guidelines; human resource management, infrastructure (information technology and communications) and health care worker support; introduction of innovations; and perceptions of the equity and quality of service delivery. Adaptations were made according to the health systems context in each country, for example in Liberia, additional questions were included to explore how learning from the EVD epidemic and other health systems shocks informed COVID-19 response planning.

Figure 1 placed here

Study participants and data collection

The study was carried out at different levels of the health system across both settings (Table 1). In Liberia, we conducted key informant interviews in June and July 2020 with 21 national level and three county level decision-makers (Nimba, Margibi and Montserrado Counties) purposively selected because of their involvement with COVID-19 planning and/or routine service delivery. Some had also played key roles in the EVD epidemic response. In Merseyside we conducted 42 key informant interviews between July to September 2020, with regional, hospital and primary care decision-makers (general practitioners and residential care home manager) and front-line workers selected because of their involvement with COVID-19 planning and/ or the delivery of COVID-19 or routine services (see Table 1). More interviews were carried out within the UK across health systems levels, due to demand for research across multiple levels and the presence of a larger team of researchers. In Liberia, by

For peer review only

contrast the demand for research was focused at national level, and the research team was smaller in size.

Table 1 Study participants' role

Participant Role	Number of Participants Interviewed
**Merseyside, UK**	
Regional decision-maker	5
Hospital decision-maker (Clinical director, medical director, ward manager)	4
Hospital consultant	11
Hospital health worker (junior doctors, nurses)	10
Health worker in community (GP, district nurse, residential care home)	7
Liverpool Clinical Laboratory staff	5
**Total**	**42**
**Liberia participants**	
National decision-maker	21
County decision-maker	3
**Total**	**24**

Interviews were predominantly carried out remotely by researchers experienced in qualitative interviewing in English language, via online platforms such as Microsoft Teams or Skype. A minority were carried out in person with physical distancing measures in place, according to local guidance at the time. All interviews were audio-recorded. Data collection stopped when no new themes emerged from additional data collected.[44] Interviews lasted approximately 30 to 60 minutes.

Data Analysis

Audio recordings were transcribed verbatim, with quality assurance conducted by a second researcher against the recording. In both Liberia and UK, preliminary data analysis workshops were held with the research team members involved with data collection. Prior to the workshops all participants reviewed transcripts to familiarise themselves with the data. Through these workshops key emerging themes were identified and used to generate a separate coding framework for each setting. All transcripts were imported into NVivo Version 12 qualitative data analysis software for coding (QSR

For peer review only

International Pty Ltd. Version 12, 2018). Most of the emerging themes aligned closely with the FCDO principles and were mapped accordingly. Only two of the identified themes were not covered by the FCDO principles, and therefore formed two new additional principles relating to “mechanisms for advance preparation” (Principle 9) and “adaptable governance and leadership structures” (Principle 10). Detailed findings and recommendations were developed into two policy briefs in accordance with these principles and were shared and discussed with relevant stakeholders from both study settings.[28,45] The relationship of the findings to the original conceptual framework was reviewed and findings compared between settings during a final on-line workshop, attended by all those involved with data collection in both settings, with key similarities and differences jointly discussed.

Ethics

Ethical approval was received from the Liverpool School of Tropical Medicine Research Ethics Committee (Protocol ID 20-045); the University of Liverpool Ethics Committee (Reference 7811) and the University of Liberia-Pacific Institute for Research and Evaluation Institutional Review Board; National Health Service Health Research Authority and Health and Care Research, Research Ethics Committee (Reference 20/HRA/2597); Integrated Research Application System (Project ID 284143). All study participants were provided with a participation information leaflet at least 48 hours prior to interview. All participants provided written, or audio recorded consent to participate.

Patient and public involvement

Neither patients nor the general public were involved in the design, conduct, reporting or dissemination of our research.

Results

For peer review only

We present findings according to the FCDO principles (Box 1)[16] (key illustrative quotes are summarised for each principle in table 2). We then reflect on the findings in light of people-centred health systems within the discussion.

Box 1 Ten Principles of Health Systems Resilience in the Context of COVID-19 Response

Principle 1 Develop flexible pathways for medical supplies
Principle 2 Prioritise a list of essential health services [*and continued provision of quality and equitable routine services*]
Principle 3 Build trust with local communities
Principle 4 Foster good communication at all system levels
Principle 5 Support, recognise and encourage staff
Principle 6 Facilitate rapid resource flow and greater flexibility in its use
Principle 7 Ensure agile tracking of health information
Principle 8 Cultivate effective partnerships and networks
Principle 9 Structures and mechanisms for advanced preparedness (**New principle**)
Principle 10 Adapt governance and leadership structures to facilitate timely decision-making and effective coordination of response **(New principle)**

Table 2 Illustrative quotations from Liberia and Merseyside related to each FCDO Principle

Principle	Quotations
**Principle 1: Develop flexible pathways for medical supplies**	*"Supply chain are affected greatly because their concentration is on how to provide the COVID response activities meaning the …medicines and medical supplies that are needed [for] NTDs (Neglected Tropical Diseases), lack of attention will now be paid to that." (LIB national decision maker 029)*<br>*"With regards to PPE, there was national guidance about what we should do and there was a huge amount of fear amongst nurses and medics and everyone else understandably. Everyone was scared. I was scared. If someone said they weren't scared, then they're lying or they're a fool. The national guidance was confused, and availability of PPE fluctuated. Procurement here [NHS hospital] did a very good job, but sometimes it just wasn't delivered nationally. And we went through other supply chains…" (LIV hospital decision maker, Merseyside UK 014)*
**Principle 2: Prioritise a list of essential health services [and continued provision of quality and equitable routine services]**	*"So we just have to be robust and do the necessary investment into routine health services, preventive in terms of creating awareness and education among health workers about covid and how we can continue to care for our patients, with fighting the infection at the same time." (LIB national decision maker 001)*<br>*"There's the whole big risk around the screening program…the screening program was stopped, restarting that it's gonna be really challenging. And I suppose that's another risk in terms of people with delayed diagnosis and the right treatment, as a result of not having had that screening mammograms." (LIV hospital decision maker Merseyside UK 051)*
**Principle 3: Build trust with local communities**	*"Some of the useful things that we have been using from Ebola time is, as I said before, to involve the communities …The community aspect is very important because it will help us for the COVID-19 where communities, family members, all of those at the community level are influential group they will be able to comply like we did in the Ebola." (LIB national decision maker 005)*<br>*"The elderly population have been shielding because of comorbidities and all that. I think they probably not being as vocal about things that they're concerned about because they're worried about that they will be asked to come in. They fear that that*

Principle	Quotations
	they will catch Covid when they come here." (LIV hospital health worker Merseyside UK 048)
**Principle 4: Foster good communication at all system levels**	*"One of the things that quickly used to come to me is to able to adapt to working with social media technology and all of that, because that's the first thing if you have to communicate with people in this manner you need to understand zooming, skyping, how to take notes.." (LIB national decision maker 029)* *"And there's so many different sources of information that say different things from what people hear within the hospital talking to friends on the corridor, that you've got to come out with a consistent message. And I think it took longer than was ideal to get a central source of information…But people need to be told what the situation is rather than try to be falsely reassured sometimes as well." (LIV hospital decision maker, Merseyside UK 004)*
**Principle 5: Support, recognise and encourage staff**	*"Like take for example, when COVID came some of our workers from the [name] Hospital was recruited to go at the front line and [hospital name] is for routine services so taking employees from there to go at the front line that tells you it kind of understaff… So routine services kind of slow down and every attention was placed on COVID but going forward, with the system in place, routine services have gotten back on its feet." (LIB national decision maker 010)* *"And it felt like there was unequal share of knowledge and also an unequal kind of confidence in protective clothing. … And I think the people that spent the most time with the patient, the patient areas, for instance, the health care assistants and the cleaning staff didn't have all of the information [at the] beginning or any PPE training." (LIV hospital health worker Merseyside UK 017)*
**Principle 6: Facilitate rapid resource flow and greater flexibility in it's use**	*"The first thing is, we need ownership by government, ownership is not depending on other countries to provide us the resources, to provide the technical capacity. So that is the best recommendation I would say. The ownership has to be there, resources have to be available and the infrastructure has to be available in terms of being resilient." (LIB national decision maker 029)* *"To be honest, it was a fairly novel experience because it was a situation where if we asked we more or less got [funding]." (LIV hospital decision maker, Merseyside UK 004)*
**Principle 7: Ensure agile tracking of health information**	*"Another recommendation is that we could include COVID-19 to our regular disease surveillance. Like we have the measles, the Lassa, and thing. I think we should include COVID because COVID maybe all around. Like we included Ebola, there should be a document on COVID-19 that will form part of our regular surveillance." (LIB county decision maker 024)* *""…there's some value in looking at the things that we were looking at before COVID, because at least we have some longitudinal data on that so that we can see what the effect of COVID is." (LIV hospital health worker, Merseyside UK 020)*
**Principle 8: Cultivate effective partnerships and networks**	*"Involvement of multi-sectorial stakeholders in the response; that was one major thing that we learned from Ebola. And that has been brought to be on this response, so there has been a spark from the level of the presidency where they have key ministries and agency heads heading pillars on the COVID-19 response, involving the community people." (LIB national decision maker 028)* *"I think one thing, it's really highlighted is the divide between hospital and primary care. We didn't work together very well before the epidemic, and we are still not working together very well. And I think if things were to get better, the whole health system needs to work better." (LIV community-level health worker, Merseyside UK 033)*
**Principle 9: Structures and mechanisms for advanced preparedness**	*"If you don't prepare well and you are caught unaware you will have a lot of issues, so we didn't wait for COVID to enter Liberia before we prepositioned basic PPE and those are all part of the preparedness phase." (LIB county decision maker 026)* *""It was blatantly obvious that anything we've ever planned for in relation to a pandemic or anything along those lines was not the plans that we needed… So I think going forward there needs to be almost a better planning system in place…it's not just*

For peer review only

Principle	Quotations
	a matter of just saying any pandemic it's about what kind of pandemic." (LIV hospital decision maker, Merseyside UK 069)
**Principle 10: Adapt governance and leadership structures to facilitate timely decision making and effective coordination of response**	*"So, at this point in time we think if you give the resources, put the money in the hands of the county health team to buy what they need, that will be more effective … So, we want decision should be given back to the people on the frontline so that they make the decision rather than a centralized point in Monrovia where people sit and decide for people in the lower level and the people choices made the right kind of thing they might need at that level." (LIB national decision maker 028)*<br>*"… we were having to work, to a large extent, in the dark. The amount of guidance that came through nationally and even regionally, was actually relatively limited at that stage and we were having to do what felt like quite a lot of planning in isolation." (LIV decision maker Merseyside UK 008)*

For peer review only

Principle 1 Develop flexible pathways for medical supplies: Across both settings supply chains were disturbed due to global shortages and price inflation. In Merseyside there was a lack of personal protective equipment (PPE) and laboratory reagents needed for COVID-19 testing. Meanwhile, in Liberia, the disturbances related to routine supplies as supply chains shifted to focus on COVID-19 related procurement. In both settings, these challenges were felt to relate to global shortages, but were worsened by failure to maintain buffer stocks at local and national levels. In both settings, participants expressed the need for greater decentralisation of procurement decisions.

Principle 2 Prioritise a list of essential health services [*and continued provision of quality and equitable routine services*]: Participants from Merseyside expressed fears that there was too much emphasis on COVID-19 care, at times creating redundant capacity, while limiting access and quality of routine essential services. The blanket discontinuation of all elective non-urgent care at the height of the first wave in Merseyside, UK was felt to be unhelpful, and a more nuanced approach which seeks to balance long-term as well as short term risks associated with health conditions was recommended. In contrast, Liberia's early emphasis on routine health services was described as a key learning prioritised by decision-making platforms following the country's experience with the EVD epidemic.

COVID-19 adaptations in the UK led to increased telemedicine, with some respondents raising access-related equity concerns, particularly for elderly populations, who may struggle to engage with telemedicine. There were also concerns raised about quality of care, with some participants in

Merseyside fearing delayed-diagnosis, misdiagnosis or sub-optimal care due to restrictions limiting physical contact with patients. In Liberia, limited opportunities for supervision, diversion of funds and staff for routine services towards COVID-19 response, and limited community outreach activities (due to physical distancing) were felt to impact quality of care. Across both settings innovations in service delivery have emerged (see policy briefs for details).[28,45]

Principle 3 Build trust with local communities: In both settings, community trust to seek health services declined, which reduced utilisation of services. In Liberia, fear among the population during the start of the pandemic led to reduction in the uptake of health services including national routine vaccination programmes and health facility-based delivery. This was felt to relate to a combination of fear of contracting COVID-19 at facilities and to reduced community outreach activities. Innovative community engagement and social mobilization strategies were introduced, for example follow-up visits to pregnant women, which led to patients returning to use services after a few months. Another example is the selective outreach home visits by the Neglected Tropical Disease (NTD) programme to NTD affected patients, in order to avoid interruption in treatment provision. In Merseyside, utilisation of non-COVID related services remained supressed for much longer. This was deemed to relate to widespread community mistrust, and Government campaigns which initially discouraged the public from visiting health facilities via the national 'Stay at home' messaging. Applying learning from Liberia's experience with EVD, the Government of Liberia placed a strong emphasis on working alongside community governance structures, involving local authorities as part of COVID-19 response.

Principle 4 Foster good communication at all system levels: The need for effective communication within the health system appeared to be a significant theme, particularly within findings from Merseyside. The rapidly changing context during the early months of the pandemic created a wealth of daily new information. Virtual forms of communication rapidly expanded in both settings, with WhatsApp and online meeting platforms used extensively. Within Merseyside, referred to challenges such as multiple sources of guidance and communication channels struggling to keep pace with the

For peer review only

changing guidance, which at times created contradictory messaging and confusion among health workers. By contrast, Liberia developed a centralised messaging procedure with approval needed from the department of Health Promotion before dissemination. In Merseyside, use of emails were typically less popular with staff as these could often be too long and wordy. Participants expressed limited scope for frontline staff to feedback on the information that had been shared.

Principle 5 Support, recognise and encourage staff: Staff redeployment was common across both settings, contributing to varied workloads. In Liberia, health worker redeployment to COVID-19 treatment centres, alongside largely unchanged utilisation rates contributed to increased workload for remaining health workers responsible for provision of routine services. By contrast in Merseyside, redeployment resulted in over-staffing in certain COVID-19 wards. Although there was disparity between health workers, with nurses experiencing increased workload. Due to the reduced volume of patients seeking routine care in the UK, workload was variable for those providing these services. The degree to which health workers received training about COVID-19 prior to having to manage COVID-19 patients varied between settings, with Liberia carrying out training in identification, isolation and infection, prevention and control before the first case of COVID-19 arrived in country, as a result of lessons learned following experiences responding to EVD. By contrast in Merseyside, the roll out of training varied widely by cadre, with some participants identifying that health care assistants and cleaning staff did not receive PPE training until later in the pandemic, compared with doctors and nurses (see table 2).

Anticipated mental health implications for health workers emerged from the Merseyside data, due to high rates of COVID-19 infection, exhaustion and high future anticipated post-traumatic stress disorder (PTSD). This was associated with fear of making treatment mistakes, stress surrounding patient escalation decision making, anxiety over potential COVID-19 infection (both personal and for family), trauma surrounding high COVID-19 infections and deaths and reduced psychosocial support due to remote working. Measures to support staff wellbeing were introduced (including counselling,

For peer review only

reflective therapy, peer support and mentoring, information made available about local support services), with varied levels of uptake. This was not widely discussed in Liberia. Although measures in Liberia to support staff wellbeing include psychosocial teams, roaming mental health counsellors providing services to health workers are in place. In Merseyside, community support, strong solidarity and teamwork were considered enablers of staff resilience.

Principle 6 Facilitate rapid resource flow and greater flexibility in its use: Historic underfunding of the health system in both settings has been highlighted by the pandemic. In Merseyside, this was considered to be due to nearly a decade of austerity, which has created weariness and uncertainty; whereas in Liberia it related to perception of reliance on external donors which predated the pandemic. Our findings confirmed the need for adequate funding to ensure the building blocks of the health system have received investment prior to the onset of any shock. With the arrival of the pandemic the availability and flexibility of funding differed between settings. In Merseyside, UK there was increased central government funding, which was mostly freed of usual bureaucratic checks. Managers noted that the removal of these bottlenecks allowed for swift action and rapid adoption of innovations. Frontline managers' ability to make operational decisions was viewed as central to resilience. In Liberia, however, there was an identified need for greater Government of Liberia ownership. Some sectors of the health system, particularly those which are donor reliant struggled in response to reduced partner support following the pandemic. Initially funding was not made available, however funds for routine service delivery were re-allocated to COVID-19 response, with implications for quality (see principle 2). Participants complained about excessive bureaucracy associated with use of funds, which created delays.

Principle 7 Ensure agile tracking of health information: Health information systems (HIS) were rapidly developed in the UK to collect huge quantities of surveillance data on COVID-19 and essential services. However, there was need for improved skills to usefully interpret this data. Respondents in Liberia stated that regular and timely submission of data, particularly from the community level had declined

For peer review only

since the onset of COVID-19. This was considered to relate to reduced data validation, with decreased supervision visits due to physical distancing. In Merseyside complex new systems were designed to collect pandemic surveillance data, however, data was frequently not analysed or made readily accessible to staff to influence timely monitoring and quality improvement in services. In Merseyside, respondents also noted that a number of new initiatives were introduced during the pandemic, such as virtual consultations, but have not yet been systematically evaluated.

Principle 8 Cultivate effective partnerships and networks: The need for well-established partnerships emerged in both settings, with Liberia already having clear multi-sectoral participation in decision-making following the Incident Management System developed following EVD. Merseyside data highlighted pre-existing weaknesses in collaboration between primary and secondary/ tertiary care have been exacerbated. In both settings the need for greater engagement with the private sector was affirmed, with respondents from UK highlighting the need for stronger links regarding PPE supply chain shortages and in Liberia the need to strengthen collaboration given perceived weakness in private facility IPC standards. Partnerships were established within Merseyside, in a range of aspects of service delivery, including: regional network of laboratory providers to address equipment challenges and ensure COVID testing; between GPs to create service hubs; between disciplines and departments within hospital to address staff shortages and share information. In Liberia, a reduction in the number of partners providing response support was noted. This was a marked contrast to the EVD response.

Principle 9 Structures and mechanisms for advanced preparedness (newly identified principle from our findings): Within Liberia in particular, but also in Merseyside, there was discussion about advanced preparedness. Respondents in Liberia emphasised how their experiences with previous shocks, particularly EVD, had facilitated learning around early recognition of the need for preparedness. For instance, there was consensus among respondents that waiting for COVID-19 to reach Liberia before responding would be too late. There was early rapid mobilisation of existing

For peer review only

emergency response systems which had been established during the EVD response including; health check controls and quarantines at border points from January 2020; health worker COVID-19 training before the first confirmed case; enhanced hygiene practices; restriction of physical contact and sustained use of PPE, building on institutional memory gained through the EVD epidemic. In contrast, respondents in Merseyside expressed that the COVID-19 response was impeded by a lack of pandemic preparedness for new emerging infectious diseases.

Principle 10 Adapt governance and leadership structures to facilitate timely decision-making and effective coordination of response (newly identified principle from our findings): Being able to adapt governance and leadership structures to facilitate timely response coordination emerged from both settings. Liberia had previously established the incident management system (IMS) in 2014 as part of the response to EVD. It was re-activated in March 2020 to guide planning their pandemic response, led by the Minister of Health. This multi-sectoral team included a range of political and public health decision-makers, donors and partner representatives. At the time the study was carried out, most decisions were made centrally, with implementation at county level. In Merseyside, early response was hindered by slow and centralised guidance and decision-making, which was perceived to be oriented towards achieving political goals, rather than providing much needed clarity and recognition of local reality. The limited scope for local autonomy was considered to strain relationships between local senior leadership who sought to enforce central directives, and frontline staff, who wanted scope to influence them. In both settings, there was interest in greater de-centralisation of decision-making to lower levels.

Discussion

Our findings indicate that a resilient health system is a people-centred health system (Figure 2). Maintaining a people-centred approach can help ensure that COVID-19 related adaptations are acceptable, understood and meet the needs of individuals (both patients and health workers). The values which underpin people-centred health systems emphasise the need for equity, orienting health

For peer review only

services towards a health system which puts “people and communities at their centre, and surrounds them with responsive services that are coordinated both within and beyond the health sector, irrespectively of country setting and development status.”(page 9 [14])

Adapting a people-centred framework

Box 2 Recommendations from our adaptation of FCDO principles

1. Supply chains should pre-position adequate stocks, diversify sources and seek decentralisation of procurement. Collaboration between providers can prove valuable in securing continuity of supplies.
2. Routine services should be prioritised with a view to long term as well as short term impact, with prioritisation re-evaluated regularly as the pandemic progresses.
3. Maintain consistent communication and engagement with community leaders as partners to participate in pandemic planning within their respective communities.
4. Keep communication channels open, with regular updates for staff which highlight the key information, preferably through meetings, rather than email.
5. Ensure adequate provision of training, with sufficient PPE for health staff particularly for those staff at highest risk of COVID-19 infection, alongside measures to balance workload and promote staff wellbeing. Prioritise compassionate leadership which is supportive of staffing levels and rotas, along with staff mental wellbeing. Investment in psychosocial wellbeing throughout and after the pandemic response.
6. Health systems need to be adequately funded during ‘normal times’ if they are to be able to respond when a shock arises. There is urgent need for investment to clear the backlog of delayed routine services.
7. Health information systems need greater investment in both the systems and the human element to be able to analyse, interpret and respond to emerging data trends.
8. Opportunities for multisectoral collaboration should be sought out, with engagement with private sector where possible.
9. Develop a proactive approach, with advance plans for health shocks, along with escalation and de-escalation plans throughout the crisis.
10. Promote greater opportunities for de-centralised staff involvement in decision-making where feasible. Governments to prioritise an outward focus towards global solidarity.

All ten principles are mapped against the original conceptual framework, to demonstrate the connection between our findings and existing literature about resilience (Figure 2) and recommendations in response to each principle are outlined in box 2.

Figure 2 placed here

Capacity and knowledge exchange

The continuation of routine essential service delivery following a shock to the health system, has previously been highlighted as an area of concern across a range of sectors.[46,47] Health systems need the capacity to continue to deliver services of good quality alongside responding to wider health challenges.[41] Our findings for principle 2 highlighted that COVID-19 adaptations in the UK led to the cancelling or postponing of many essential services, including those related to cancer care, which has been anticipated to decrease life expectancy and survival.[47,48] Meanwhile, Liberia emphasised the need for continuation of routine services and the promotion of patient confidence to use these services. This is in contrast to the EVD epidemic, where over 80% reductions in maternal delivery care in EVD affected areas were described and form part of the reason why routine care was prioritised so strongly as part of the COVID-19 response.[49]

Our findings relating to supply chain (principle 1) resonate with literature from previous shocks and research emerging from the COVID-19 pandemic.[50,51] We found the need for greater flexibility, with engagement with a more diverse range of suppliers and greater decentralised control over supply chain across both settings. This is in keeping with a recent systematic review of supply chain resilience literature, which identified the importance of diversity and the social aspects of supply chains during a pandemic response.[50] Supplying commodities without investing in health systems strengthening will not produce a robust supply chain, limiting ability to respond quickly and effectively to future demands.[50]

We found a strong focus on the need for support for the health workforce, particularly in UK (principle 5). This was not as widely discussed in Liberia (though this may be a limitation relating to differing levels of participants between countries). However, a previous study in Sierra Leone and Liberia, highlighted that many providers may carry unresolved trauma from earlier shocks (including the Ebola epidemic), which may have implications for them during the COVID-19 response.[52,53] Research among health workers treating patients with COVID-19 in China, revealed health workers had a higher

For peer review only

prevalence of insomnia, anxiety, depression, somatisation and obsessive-compulsive symptoms compared with nonmedical health workers, indicating the need for support and recovery programs for these staff.[54] Stressors identified among workers in China, include many of those described by participants in both settings within our study, particularly within Merseyside, including difficulties feeling safe at work, lack of infection prevention and control (IPC) measures and COVID-19 knowledge, long term workload, high risk of exposure to COVID-19, shortage of PPE and lack of rest, among others.[54]

Our findings regarding resource flow to frontline providers (Principle 6), are in keeping with previous study which identified funding as a core dimension within a health systems' ability to adapt and respond to shocks.[55] A recent systematic review found aggregate public spending for health is associated with improved life expectancy, reduced child and infant mortality and more equitable health outcomes.[51]

Relational and teamwork components

The relational components which exist are shaped by risk, trust, values, power, norms, and culture.[41] These components play a role in determining the success (or failure) in response to a health systems shock or crisis. In contrast to the FCDO recommendation for good communication between actors (principle 4), our findings highlight challenges, particularly in the UK, where communication channels struggled to keep pace with changing guidance creating contradictory messaging and confusion among health workers. This is in keeping with previous study which found differences in lines of authority and acceptability of communication pathways can contribute to problems in communication.[33] In response, key principles were identified including participation for all, respect, information sharing, collaboration and problem-solving.[33]

The need for strong governance structures and leadership which adapts to the response (principle 10), was identified as a gap within early response in Merseyside. This was felt to have been hindered by slow and centralised guidance and decision-making with a perceived limited scope for autonomy

For peer review only

within decision-making at lower levels. Within Liberia learning from the EVD response, and establishing an incident management system (IMS) (led by the Minister of Health) and Special Presidential Advisory Committee on Coronavirus (SPACC) (led by the President) early in planning their pandemic response enabled timely decision-making.[26] In both settings, there was interest in greater de-centralisation of decision-making to lower levels. Blanchet et al (2017) emphasised the need for legitimacy within resilience, with requirement of capacity to develop socially and contextually accepted institutions and norms.[39]

Looking more broadly, the conceptual framework highlights community engagement, with the community being active participants of any health systems response (principle 3).[38] Our findings emphasise the value of community engagement within the response within Liberia, based on lessons from the EVD pandemic and in keeping with WHO recommendation that this be a key pillar within COVID-19 country response.[8] Liberians across all socio-demographic groups responding to a recent survey said they were very well, or somewhat well informed about the COVID-19 pandemic, with only 5% feeling not very well/ not at all informed.[26] This also emerged as a key finding in Singapore, with engagement through new and social media channels monitored, with clarification of misinformation by MOH.[56] In contrast to the findings from Liberia, participants from Merseyside highlighted the need for stronger communication (although there were some examples of creative ways to engage with diverse communities).

Learning from our study has emphasised the need to better prepare for, and respond to, health emergency crises through integrated services (Principle 9).[43] A recent survey found most of the population felt the Liberian government was doing well in managing the pandemic.[43] This contrasted with findings from the UK where there was felt to have been a lack of adequate advance planning and preparation. Two previous literature reviews highlighted that “preparedness depends on health systems ability to learn from prior pandemics”, with responses often reactive rather than proactive.[51,57]

For peer review only

The people-centred approach stresses the need for awareness and recognition of the interdependencies of the health system with the community and other social systems, including education, social protection and food security and their relationship with social determinants of health (principle 8).[58] Our findings emphasise the need for strong partnerships with other sectors across settings, in keeping with an identified success in Singapore's response,[56] and is a key aspect of Blanchet et al.'s resilience framework, ensuring the capacity to engage with and handle multiple actors and dynamics.[39]

Our findings, particularly from Merseyside emphasise the vast quantities of data being generated through the COVID-19 response, but there are gaps in how this data is analysed and utilised within the health system. The importance of adequate HIS is in keeping with previous studies.[39,55] A health system's ability to identify and respond to an emerging threat is needed if it is to appropriately meet emerging needs during a rapidly evolving health crisis or shock (principle 7).[39,40] A robust health management information system (HMIS) is crucial to a health systems capacity to respond to shock.[55] Health systems need to have the ability to combine and integrate different forms of knowledge and to anticipate and cope with uncertainties and unplanned events.[39]

Reflections on the need for global solidarity within pandemic response

The COVID-19 pandemic has emphasised the need for global collective action, rather than an individual response for there to be genuine resilience, with COVID-19 having reflected and exacerbated existing social inequalities.[8] 'Global powerhouses' and 'so-called' advanced democracies' have struggled in their response to COVID-19 due to a failure to adequately adopt people-centred approaches within the response, with reductions in the quality of governance and a lack of commitment to equity in health service delivery and supporting health workers' wellbeing.[8] Excessive self-interest and a lack of global solidarity on the part of some richer countries, particularly with regards to vaccination are dominating the current phase of the pandemic (September 2021). The hoarding of vaccinations by some richer countries, while health workers and vulnerable populations

For peer review only

elsewhere remain unvaccinated has been widely criticised from both a moral perspective, and from a scientific one, since "until we're all safe, none of us is safe".[59]

Strengths and Limitations

The strengths of this study include the quality of data analysis, which involved a wide range of researchers across both settings, and the breadth of perspectives captured from frontline staff and key decision-makers early in the course of the pandemic. Our study had a number of limitations. Within Merseyside, study participants were selected from across a range of health system levels including primary care, hospital frontline workers and decision-makers as well as regional decision-makers. By contrast, in Liberia participants included national and county level decision-makers, technicians and supervisors of frontline staff, with no direct frontline workers included. This may result in some of the differences in findings, related to these differing perspectives. Perhaps the greatest limitation of this study is that it was carried out at a single point in time. In Merseyside we collected data towards the end of the first wave, at a time when there were few inpatients and people were reflecting on the first wave. Meanwhile in Liberia it was carried out before there had been a large increase in cases. Since the study was carried out there have been subsequent even greater waves of cases within Merseyside, UK and Liberia has experienced a large surge in cases of the delta variant (59% of cases recorded in Liberia up until 17th July 2021, occurred during a six week period from June 1 2021 to 17th July 2021).[60] By the weeks beginning July 24th to August 7th 2021 number of confirmed cases had declined between zero to 43. Response measures have evolved in both settings, and limitations identified through the study may have been addressed in subsequent stages of the pandemic.

Conclusion

We found the ability of health systems to be able to absorb, adapt and transform in response to the COVID-19 pandemic in two very different settings closely relates to the eight FCDO principles of resilience.[16,39] We expanded these principles to include strong structures and mechanisms for

For peer review only

advance preparation, and adaptable governance and leadership structures to facilitate timely decision-making and response coordination. At the heart of our findings lies the centrality of the people-centred health system, where the person, is placed within their family, community and the health system.[14] When all aspects work together the outcome is the extent of resilience demonstrated within a health system in response to shock.[39] This includes both the provision of specific services in response to the shock experienced, as well as continued provision of and demand for 'routine care'. Our study highlights the need to maintain a people-centred approach for a resilient health system response.

Acknowledgement

We would like to thank all the participants who made time to share their experience and reflections to make this research possible. We recognise and thank Abiola Aiyenigba, who sadly passed away during the study, for her inputs. This research was funded by the NIHR Health Protection Research Unit in Emerging and Zoonotic Infections, and the Centre of Excellence in Infectious Diseases Research, and the Alder Hey Charity. We also acknowledge support of Liverpool Health Partners and the Liverpool-Malawi-Covid-19 Consortium. Additional funding to support this work came from the NIHR REDRESS Programme (NIHR2001129) and FCDO COUNTDOWN (PO6407) programme. We thank Tim Martineau, and Joanna Raven (LSTM) for early inputs into study design and Susie Crossman for managing the study budget.

Competing Interests

None declared.

Author Statement

RM prepared the first draft of the paper with inputs from all; Study design, conceptualisation, ethics (ST, LD, MT, LF, IB, ZZ, RM, VW, HP, RAdC, RH, KK); conducted interviews in UK – RM, VW, MT, KO, HP, SC, ST, TEH, RH, RD, YD, OH; conducted interviews in Liberia - ZZ, WT, HB, JK, JSS, CP, GZ, RM. All

For peer review only

interviewers participated in the cross-country analysis which was led by YA in the UK with inputs from those who conducted UK interviews and LD, RM, ZZ, HB, WT, JK, JSS, GZ, CP in Liberia. All authors were involved in critical review of the approach, inputted into and approved the final draft of the manuscript.

For peer review only

References

1. Meyer D, Bishai D, Ravi SJ, et al. A checklist to improve health system resilience to infectious disease outbreaks and natural hazards. *BMJ Glob Heal*. 2020;5(8):1–7.

2. World Health Organization. Operational guidance for maintaining essential health services during an outbreak [Internet]. 2020. Available from: https://apps.who.int/iris/bitstream/handle/10665/331561/WHO-2019-nCoV-essential_health_services-2020.1-eng.pdf?sequence=1&isAllowed=y

3. United Nations Economic Commission for Africa. COVID-19 in Africa: Protecting Lives and Economies [Internet]. 2020. Available from: https://repository.uneca.org/bitstream/handle/10855/43756/b11983206.pdf?sequence=1&isAllowed=y

4. Agyeman AA, Laar A, Ofori-Asenso R. Will COVID-19 be a litmus test for post-Ebola sub-Saharan Africa? *J Med Virol*. 2020;92(9):1373–5.

5. Zar HJ, Dawa J, Bueno G, et al. Challenges of COVID-19 in children in low- and middle- income countries. *Paediatr Respir Rev Rev*. 2020;(January).

6. Burki T. The indirect impact of COVID-19 on women. *Lancet Infect Dis* [Internet]. 2020;20(8):904–5. Available from: http://dx.doi.org/10.1016/S1473-3099(20)30568-5

7. Bayani DB, Krubiner C, Barasa E, et al. The Indirect Health Effects of COVID-19: Emerging Findings from Kenya, the Philippines, South Africa, and Uganda. *Cent Glob Dev*. (July 2021).

8. D'Ambruoso L, Abbott P, Binagwaho A. Building back fairer in public health policy requires collective action with and for the most vulnerable in society. *BMJ Glob Heal*. 2021;6(3):8–11.

9. Dzinamarira T, Dzobo M, Chitungo I. COVID-19: A perspective on Africa's capacity and response. *J Med Virol*. 2020;92(11):2465–72.

For peer review only

10. World Bank. World Bank Population Data [Internet]. World Bank. 2020. Available from: http://data.worldbank.org/indicator/SP.POP.TOTL?locations=ET

11. JHU. John Hopkins Coronavirus Resource Center [Internet]. Johns Hopkins University & Medicine. 2021 [cited 2021 Jun 7]. Available from: https://coronavirus.jhu.edu/

12. FPA Reporter. Former Center for Disease Control Boss Hails Liberia for COVID-19 Prevention [Internet]. Front Page Africa. [cited 2021 Jun 7]. Available from: https://frontpageafricaonline.com/news/former-center-for-disease-control-boss-hails-liberia-for-covid-19-prevention/

13. Frieden T. Which Countries Have Responded Best to Covid-19? Wall Street Journal [Internet]. 2021 Jan 1; Available from: https://www.wsj.com/articles/which-countries-have-responded-best-to-covid-19-11609516800

14. World Health Organisation. WHO Global Strategy on people-centred and integrated health services, Interim Report [Internet]. Geneva: World Health Organisation; 2015. Available from: https://apps.who.int/iris/bitstream/handle/10665/155002/WHO_HIS_SDS_2015.6_eng.pdf;jsessionid=B2F4B22FC042ACD2BEF20C5F34803E56?sequence=1

15. Saulnier DD, Blanchet K, Canila C, et al. A health systems resilience research agenda: moving from concept to practice. *BMJ Glob Heal*. 2021;6(8):e006779.

16. Department for International Development [DFID]. Principles of Health Systems Resilience in the Context of Covid-19 Response [Internet]. 2020. Available from: https://assets.publishing.service.gov.uk/media/5ea2b33de90e070498c5538c/Principles_of_Health_Systems_Resilience_in_the_Context_of_COVID_Research_Brief_April_2020.pdf

17. Van Damme W, Dahake R, Delamou A, et al. The COVID-19 pandemic: Diverse contexts; Different epidemics - How and why? *BMJ Glob Heal*. 2020;5(7):1–16.

18. The World Bank. Liberia poverty headcount ratio [Internet]. 2016. Available from:

For peer review only

https://data.worldbank.org/indicator/SI.POV.DDAY?locations=LR

19. UNDP. Human Development Report 2020: The Next Frontier Human Development and the Anthropocene [Internet]. UNDP: New York, NY, USA. 2020. Available from: http://hdr.undp.org/sites/all/themes/hdr_theme/country-notes/LBR.pdf

20. Ministry of Health and Social Welfare (MoHSW) L. Liberia National Community Health Services Policy.

21. Ministry of Health Liberia. Investment plan for building a resilient health system in Liberia [Internet]. Vol. 12, Monrovia, Ministry of Health. Monrovia, Liberia; 2015. Available from: https://www.resilientinstitutionsafrica.org/sites/default/files/2018-09/%5BLIBERIA%5D Investment Plan for Building a Resilient Health System %282015-2021%29.pdf

22. Liverpool City Council. Liverpool City Council: public health transformation six years on [Internet]. 2019. Available from: https://www.local.gov.uk/liverpool-city-council-public-health-transformation-six-years

23. Statista. Number of coronavirus (COVID-19) cases in the United Kingdom (UK) as of April 5, 2021, by country/region [Internet]. 2020 [cited 2021 Jun 7]. Available from: https://www.statista.com/statistics/1102151/coronavirus-cases-by-region-in-the-uk/

24. Mayor of Liverpool. A STRONG AND GROWING CITY BUILT ON FAIRNESS. 2018.

25. University of Liverpool, Public Health England, Joint Biosecurity Centre, et al. Liverpool Covid-SMART Community Testing Pilot [Internet]. Liverpool; 2021. Available from: https://www.liverpool.ac.uk/media/livacuk/research/Mass,testing,evaluation.pdf

26. Sanny JA-N, Bloh O. Liberians laud government ' s COVID -19 response but claim unfair distribution of relief assistance. *Afrobarometer*. 2021;(420):1–16.

27. Kateh F. How Ebola helped Liberia prepare for covid [Internet]. MIT Technology Review.

For peer review only

2020. Available from: https://www.technologyreview.com/2020/08/19/1007139/liberia-kateh-covid-coronavirus-ebola/

28. Parker C, Zawolo G, Kollie J, et al. PRINCIPLES FOR PROMOTING RESILIENT HEALTH SYSTEMS COVID-19 RESPONSE : LEARNING FROM LIBERIA Principle One : Develop flexible. 2020;

29. Stevens S, Pritchard A. Letter to Chief Executives of all NHS trusts and foundation trusts regarding second phase of NHS response to COVID19 [Internet]. NHS; 2020 p. 1–9. Available from: https://www.england.nhs.uk/coronavirus/wp-content/uploads/sites/52/2020/04/second-phase-of-nhs-response-to-covid-19-letter-to-chief-execs-29-april-2020.pdf

30. Rosenbaum L. Medicine a nd Society The Untold Toll — The Pandemic ' s Effects on Patients without Covid-19. *N Engl J Med*. 2020;1–4.

31. Pope C, Mays N. Qualitative Research: Reaching the parts other methods cannot reach: an introduction to qualitative methods in health and health services research. *BMJ*. 1995 Jul 1;311:42–5.

32. Kuper A, Reeves S, Levinson W. An introduction to reading and appraising qualitative research. *BMJ Br Med J*. 2008;337:404–9.

33. Gilson L, Barasa E, Nxumalo N, et al. Everyday resilience in district health systems: emerging insights from the front lines in Kenya and South Africa. *BMJ Glob Heal* [Internet]. 2017;2(2):e000224. Available from: http://gh.bmj.com/lookup/doi/10.1136/bmjgh-2016-000224

34. Biddle L, Wahedi K, Bozorgmehr K. Health system resilience: a literature review of empirical research. *Health Policy Plan*. 2020;1–26.

35. Sheikh K, George A, Gilson L. People-centred science: strengthening the practice of health policy and systems research. *Heal Res Policy Syst*. 2014;12(1):19.

For peer review only

36. Sheikh K, Ranson MK, Gilson L. Explorations on people centredness in health systems. *Health Policy Plan*. 2014;29(Suppl 2):ii1.

37. Gilson L, Barasa E, Nxumalo N, et al. Everyday resilience in district health systems: Emerging insights from the front lines in Kenya and South Africa. *BMJ Glob Heal*. 2017;2(2):1–15.

38. Newton-lewis T, Witter S, Fortnam M, et al. Working Paper : What is a Shock-Responsive Health System ? *Maint Res Support Soc Serv to Adapt to Shock*. 2020;(June):1–11.

39. Blanchet K, Nam SL, Ramalingam B, et al. Governance and capacity to manage resilience of health systems: Towards a new conceptual framework. *Int J Heal Policy Manag* [Internet]. 2017;6(8):431–5. Available from: http://dx.doi.org/10.15171/ijhpm.2017.36

40. Kruk ME, Ling EJ, Bitton A, et al. Building resilient health systems: A proposal for a resilience index. *BMJ* [Internet]. 2017;357(May):1–8. Available from: http://dx.doi.org/doi:10.1136/bmj.j2323

41. Barasa EW, Cloete K, Gilson L. From bouncing back, to nurturing emergence: Reframing the concept of resilience in health systems strengthening. *Health Policy Plan*. 2017;32(1):iii91–4.

42. Starfield B. Is patient-centered care the same as person-focused care? *Perm J*. 2011;15(2):63.

43. World Health Organization (WHO). The WHO global strategy on people- centred integrated health services services delivery and safety department [Internet]. Service Delivery and Safety. 2015. Available from: https://apps.who.int/iris/bitstream/handle/10665/155002/WHO_HIS_SDS_2015.6_eng.pdf?sequence=1&isAllowed=y

44. Saunders B, Sim J, Kingstone T, et al. Saturation in qualitative research: exploring its conceptualization and operationalization. *Qual Quant*. 2018;52(4):1893–907.

45. Alhassan Y, McCollum R, Watson V, et al. PRINCIPLES FOR PROMOTING RESILIENT HEALTH

For peer review only

SYSTEMS COVID-19 RESPONSE : [Internet]. 2021 [cited 2021 Jun 7]. Available from: https://www.redressliberia.org/wp-content/uploads/2021/06/Merseyside-Policy-Brief.pdf

46. Akseer N, Kandru G, Keats EC, et al. COVID-19 pandemic and mitigation strategies: Implications for maternal and child health and nutrition. *Am J Clin Nutr*. 2020;112(2):251–6.

47. Rajan S, Chakrabarti D, Akhtar N, et al. Searching for answers: Cancer care during the COVID pandemic. *Clin Epidemiol Glob Heal*. 2021;10(January).

48. Thorlby R, Fraser C, Gardner T. Non-COVID-19 NHS Care During the Pandemic [Internet]. The Health Foundation. 2020. Available from: https://www.health.org.uk/news-and-comment/charts-and-infographics/non-covid-19-nhs-care-during-the-pandemic

49. Elston JWT, Cartwright C, Ndumbi P, et al. The health impact of the 2014 - 15 Ebola outbreak. *Public Health* [Internet]. 2016;143:60–70. Available from: http://dx.doi.org/10.1016/j.puhe.2016.10.020

50. Golan MS, Jernegan LH, Linkov I. Trends and applications of resilience analytics in supply chain modeling: systematic literature review in the context of the COVID-19 pandemic. *Environ Syst Decis* [Internet]. 2020;40(2):222–43. Available from: https://doi.org/10.1007/s10669-020-09777-w

51. Witter S, Palmer N, Balabanova D, et al. Evidence review of what works for health system strenthening, where and when? 2021;(February):99. Available from: https://rebuildconsortium.com/resources/research-reports/evidence-review-of-what-works-for-health-systems-strengthening-where-and-when/

52. Dean L, Cooper J, Wurie H, et al. Psychological resilience, fragility and the health workforce: Lessons on pandemic preparedness from Liberia and Sierra Leone. *BMJ Glob Heal*. 2020;5(9).

53. Tengbe S. World Mental Health Day 2020 one LSTM student’s research on mental health amongst health workers in Sierra Leone [Internet]. Available from:

For peer review only

https://www.lstmed.ac.uk/news-events/news/world-mental-health-day-2020-one-lstm-student’s-research-on-mental-health-amongst

54. Zhang WR, Wang K, Yin L, et al. Mental Health and Psychosocial Problems of Medical Health Workers during the COVID-19 Epidemic in China. *Psychother Psychosom*. 2020;89(4):242–50.

55. Hanefeld J, Mayhew S, Legido-Quigley H, et al. Towards an understanding of resilience: Responding to health systems shocks. *Health Policy Plan*. 2018;33(3):355–67.

56. Chua AQ, Tan MMJ, Verma M, et al. Health system resilience in managing the COVID-19 pandemic: lessons from Singapore. *BMJ Glob Heal*. 2020;5(9):e003317.

57. Sharma A, Borah S, Moses A. Responses to COVID-19: The role of governance, healthcare infrastructure, and learning from past pandemics. *J Bus Res*. 2021;(January):597–607.

58. UNICEF. Children and AIDS: third stocktaking report. TT - [Internet]. New York, New York, UNICEF, 2008.; 2008. Available from: http://www.unicef.org/devpro/files/CnA_TSR_LoRes_PDF_EN_USLetter_11202008.pdf

59. Richard H. It’s self-interest to share our vaccines globally. *BMJ*. 2021;372:2021.

60. Ministry of Health Liberia. Liberia COVID-19 Daily Sitrep 510, 7th August 2021. Monrovia, Liberia; 2021.

For peer review only

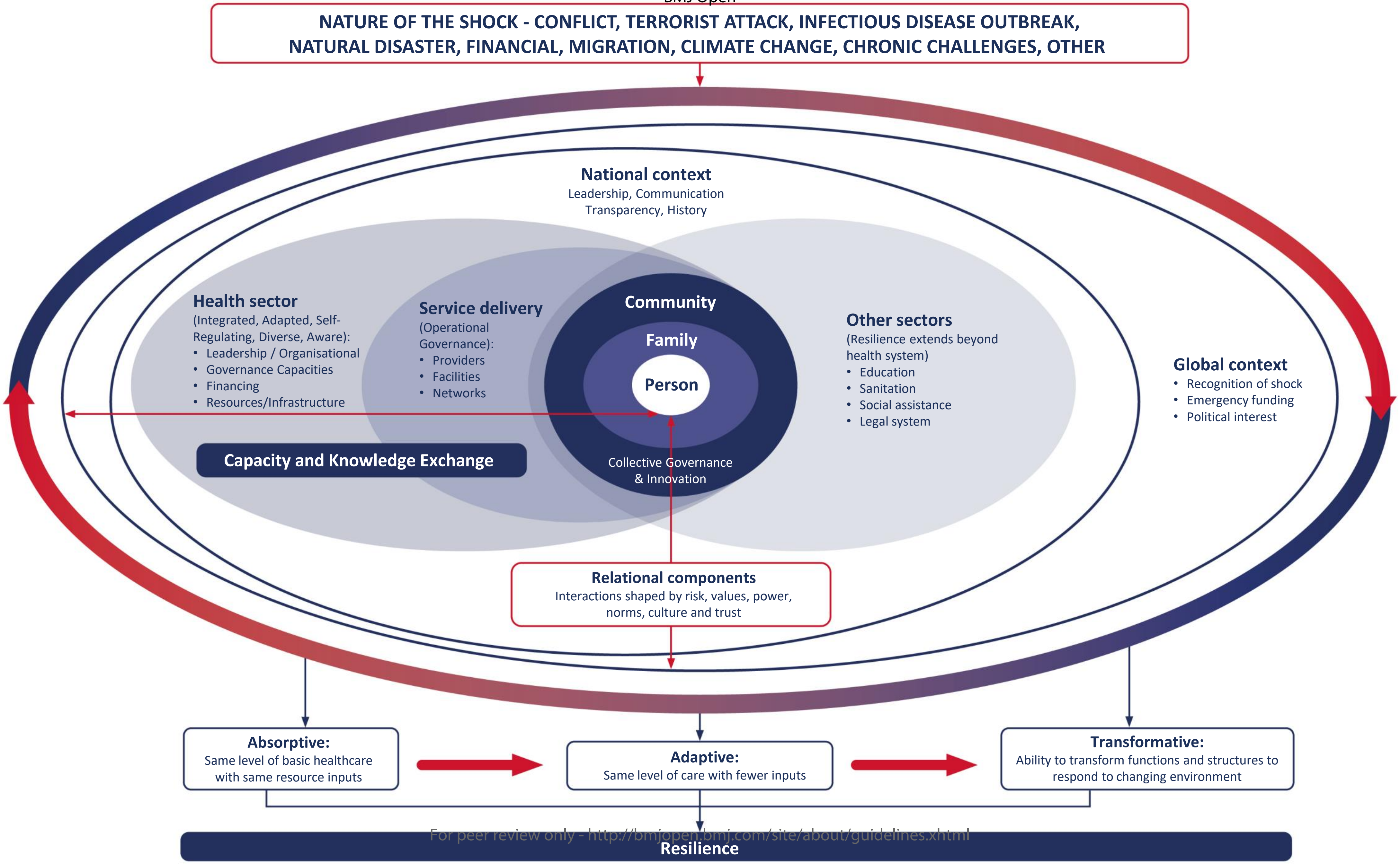

For peer review only - http://bmjopen.bmj.com/site/about/guidelines.xhtml

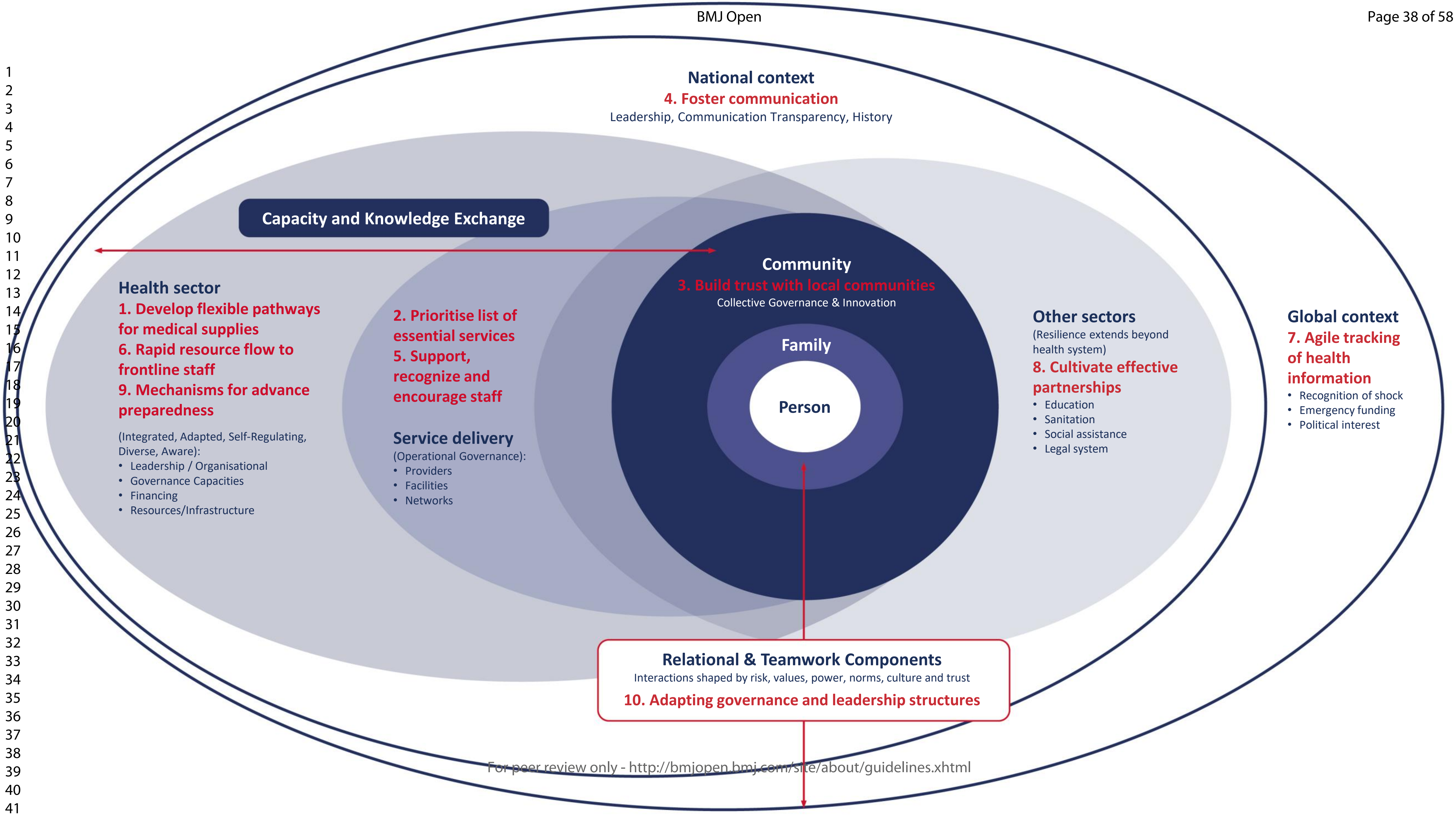

For peer review only - http://bmjopen.bmj.com/site/about/guidelines.xhtml

Appendix 1: COVID-19 Key Informant Interview Topic Guides

Contents

Appendix 1: COVID-19 Key Informant Interview Topic Guides 1
Key Informant Interviews Topic Guide –MOH Liberia 2
Key Informant Interviews Topic Guide –Merseyside Regional Decision Makers 4
Key Informant Interviews Topic Guide – Health Workers 6
Key Informant Interviews Topic Guide –Merseyside Laboratory and Blood Transfusion Staff 8

For peer review only

For peer review only - http://bmjopen.bmj.com/site/about/guidelines.xhtml

Key Informant Interviews Topic Guide –MOH Liberia

All possible questions to be asked of key informants are described in the following guide. Prior to interviewing each stakeholder, specific guides for these individuals will be made. One interview that covers relevant research themes will be completed with each stakeholder. Ordering of questions will also be revised to ensure logical flow through the interview and to avoid repetition.

Background

Please can you tell me your position and how long you have worked in your current role?

How has your role changed due to the current COVID-19 crisis?

Responses to Shock and the General Health System

1. How do you think the health system has coped with the COVID-19 crisis? How did it compare with previous crisis? How have routine services been impacted?
2. How is the current shock (COVID-19) the health system is experiencing similar or different to those you have experienced before?
3. What are the key learnings from previous shocks (Ebola/ conflict/ economic crisis)? How are they being used to respond now?
4. How do you think routine health systems functions are being impacted by the current crisis (COVID19/economic)?
5. What do you think could be done to support continuation of routine services? How is this informed or shaped by learnings from during the Ebola period?
    a. How would you describe the quality of services usually? How is quality of care being maintained throughout the COVID-19 response?

6. What policy or guidelines are supporting with the current COVID response? What additional guidelines or policies could be helpful for the COVID response?

Service Specific Impacts

Questions in this section to be reviewed/modified for cross-cutting MOH functions, e.g. M&E, research division prior to starting interview

7. Can you tell me about how service delivery within your programme/section (adapt to include name of section depending on who talking too) has been affected by the COVID pandemic?
    a. Which of your services would you say have been most impacted so far? Why?
    b. Which services would you envisage will be most impacted moving forwards? Why?
8. How have your routine services been modified or adapted? Which components of your service do you view as essential? Why?
9. Which specific sub-populations is routine care most impacted for? Are there any marginalised groups who may struggle to use services since the onset of the COVID-19 crisis? (Probe: e.g gender, dis/ability, rural/urban; wealth; geographic regions; age etc)
10. Have there been any innovations within service delivery in response to the COVID-19 crisis, and have they been useful in any way?
11. Has there been any innovations in response to COVID-19 that have concerned you?

Human Resource Management

12. How have you planned for staffing to meet the changing additional workload in response to COVID? Any tools/ guidance from the human resource section? Successes and challenges? (Prompt for role of new community health cadres, for those providing face to face care and for MOH staff)
13. What additional skill development have you provided and how in response to COVID? Successes and challenges?
14. How are you able to support staff so they can continue to work effectively during the COVID pandemic
    a. How have you supported staff through communication?
    b. How have you supported staff for occupational safety including PPE?

COVID-19 Key Informant Interview Topic Guides

c. How have you supported staff through with psychosocial support?
d. What have been the successes and challenges with supporting staff?

Service and System Impacts: Governance and Decision Making
Questions in this section to be reviewed/modified to make these questions more service-specific, depending on the interviewee's programme area

15. How are decisions made about which services should or should not be prioritised as part of the COVID response? (prompt for in relation to their specific service and also in relation to general health system, prompt for donor influence)
16. How does decision-making as part of the COVID response influence routine planning activities? What has been the impact of resource re-distribution as part of the COVID response?
17. Who is involved in this decision making and what are the processes? What are the challenges?

18. What do you think are the key ethical impacts of making these decisions? What ethical guidelines are currently in place and important in decision making during this period?
19. What guidance documents are available to support you in making decisions regarding COVID?
20. What guidance documents would help to support maintaining routine services?

Closing Questions

21. What does a resilient health system look like to you? What are your three recommendations would you make to improve or maintain the resilience of the Liberian health system during this period?
22. What are your three recommendations would you make post crisis to ensure the return to routine function of the health system as effectively as possible?

Additional questions for Director of personnel only

23. What are the main sources of additional staffing (e.g. secondment/redeployment, task-shifting, improved productivity, early graduation/students, returnees, volunteers)? Successes and challenges? Optional: Impact on the wage bill?
24. What areas of service are now struggling with staffing?
25. What are you able to do to retain staff? Successes and challenges?
26. What impact did/is down-sizing of "non-essential staff' have on your programme during the crisis?

Thank-you
Any other comments?

For peer review only - http://bmjopen.bmj.com/site/about/guidelines.xhtml

Key Informant Interviews Topic Guide –Merseyside Regional Decision Makers

Version1.1_01052020

All possible questions to be asked of key informants are described in the following guide. Prior to interviewing each stakeholder, specific guides for these individuals will be made. One interview that covers relevant research themes will be completed with each stakeholder. Ordering of questions will also be revised to ensure logical flow through the interview and to avoid repetition.

Question List

Background

Please can you tell me your position and how long you have worked in your current role?

Impact of COVID 19 on Routine Service Delivery

1. What are defined as essential routine services?
2. Which are the main scheduled and unscheduled services affected by COVID-19 and how have these been adapted over time?
3. Have there been any innovations within service delivery, and what have these been?
4. Have there been any changes that have concerned you? Why?
5. What would help to support maintaining routine services?

Governance and Decision Making

6. What has informed your decision-making, such as guidance documents or governance decision-making processes?
7. Who is involved in decisions made about which services should or should not be prioritised?
8. How are decisions made about which services should or should not be prioritised?
9. Describe how and who is involved in operationalising decisions?
10. What challenges have you faced in making these decisions?
11. What are the main differences between various sites in the trust, especially between Aintree and the Royal Hospitals?
12. How are changes in service delivery communicated? How can this be improved? There are multiple guidelines at national and local levels, how are these disseminated? How well does this work? How rapidly? How do health care workers respond to these changes?

Human Resource Management

13. How have you [may be the employer in general] planned for staffing to meet the changing additional workload? Any tools/ guidance from national authorities? Successes and challenges?
14. How have you planned for the increase in staff absence?
15. What additional skill development have you provided and how? What have been the successes and challenges?
16. How are you able to support staff so they can continue to work effectively (e.g. communication, occupational safety including PPE, psychosocial support)? What have been the successes and challenges?

Recovery post COVID-19

17. Are there any COVID-19-related changes to routine health services that you think it would be useful to continue after COVID-19? Which ones and why?
18. What next steps do you believe should be taken now to support the health system to recover post COVID-19?

COVID-19 Key Informant Interview Topic Guides

For peer review only - http://bmjopen.bmj.com/site/about/guidelines.xhtml

Thank-you

Do you have any further suggestions for improvements to delivery of routine services?

Any other comments?

For peer review only

For peer review only - http://bmjopen.bmj.com/site/about/guidelines.xhtml

Key Informant Interviews Topic Guide – Health Workers

Version1.1_01052020
All possible questions to be asked of key informants are described in the following guide. Prior to interviewing each stakeholder, specific guides for these individuals will be made. One interview that covers relevant research themes will be completed with each stakeholder. Ordering of questions will also be revised to ensure logical flow through the interview and to avoid repetition.

Question List
Background
Please can you tell me your usual position and how long you have worked in that role?
Are you currently working in your usual role and department?
If no, what role and department are you now working in?

Impact of COVID 19 on Routine Essential Service Delivery
1. Can you tell me about how health service delivery has been affected by the COVID pandemic? What was the processes for this, how was it communicated and do you have any ideas about how this can be improved? How prepared did you feel for these?
2. What do you consider to be routine essential health services in your work?
3. Which are the main scheduled and unscheduled services affected by COVID-19 in your department and how have these been adapted over time?
4. What have been the strengths and challenges with these changes? How has quality been affected?
5. How should these changes be evaluated? What indicators should be used?
6. What is worrying you most about your service now?
7. Which services would you envisage will be most impacted moving forwards as the pandemic progresses? (e.g. hospital based, community care, disease specific services, etc) Why?
8. Who do you think are the people most impacted by the changes in routine service delivery? Would you say that patients with specific socio-demographic characteristics are more impacted by service disruption/ distortion than others? Why? (e.g. gender, dis/ability; rural/urban; wealth; geographic regions; age etc) What can be done to ensure that these patients can still use health services when they need them?

Ethics and Decision Making
9. Have you encountered any health systems issues which you found troubling since the start of the COVID-19 pandemic? Would you be willing to tell me more about these issues?
10. What is the impact of these issues on you as a health worker? What would be helpful to support you in dealing with these issues?
11. Do you know of any ethical guidelines in place to guide you as you make difficult decisions during this time? What are these? How are these ethical guidelines operationalised? Are they useful?
12. Have you been involved with making decisions about the changes to health services since the COVID-19 pandemic? What was your role in making these decisions? How were these decisions made?
13. When there are changes in how health services are delivered how are these communicated with you? How has this worked? What do you think is the best way to be informed?

Human Resource Management
14. How has your role changed since the start of the COVID-19 pandemic? What have been the successes and challenges with how your role has changed? Probe workload
15. Is there anything about your role that concerns you? What?
    a. Probe working outside are of expertise
    b. No indemnity if make an error
    c. Communication about working across disciplines

For peer review only - http://bmjopen.bmj.com/site/about/guidelines.xhtml

16. What preparation for the changes to your role have you had and how was it delivered (skills - key ones, psychological support)? What have been the successes and challenges?
    a. Probe PPE training
    b. COVID clinical training
    c. Support mechanisms
    d. Team formation
17. What kind of support (e.g. communication, occupational safety including PPE, psychosocial support) are you receiving to do your job from your team/manager/employer? What have been the successes and challenges?

Recovery post COVID-19

18 . Are there any COVID-19-related changes or innovations to routine health services that you think it would be useful to continue after COVID-19? Which ones and why?

19. What next steps do you believe should be taken now to support the health system to recover post COVID-19?

20. What is worrying you most as the response moves forward?

Thank-you

Do you have any further suggestions for improvements to delivery of routine services?

Any other comments?

For peer review only

For peer review only - http://bmjopen.bmj.com/site/about/guidelines.xhtml

Key Informant Interviews Topic Guide –Merseyside Laboratory and Blood Transfusion Staff

Version1.1_01052020

All possible questions to be asked of key informants are described in the following guide. Prior to interviewing each stakeholder, specific guides for these individuals will be made. One interview that covers relevant research themes will be completed with each stakeholder. Ordering of questions will also be revised to ensure logical flow through the interview and to avoid repetition.

Background

Please can you tell me your position and how long you have worked in your current role?

Governance and Decision Making - Relating Directly to COVID-19

1. What has been the decision-making process for the laboratory's response to COVID-19 testing services and when did discussions start around re-adjusting services for COVID-19?
2. Who held overall responsibility for how COVID-19 testing was going to be conducted at LCL?
3. In addition to PHE, have the Liverpool Clinical Laboratory services worked closely/ collaborated with any other external partners for COVID-19 testing? If so whom and in what capacity?

Governance and Decision Making - Relating to Maintaining Routine Service Delivery

4. How are decisions made about which services should or should not be prioritised; which ones were considered to be essential and why? Who is involved in this decision making? How were these decisions communicated?
5. What guidance documents were most useful to you in making these decisions? In what way were they useful?
6. What key challenges have you faced in making these decisions? Do you have any support needs here?

Impact of COVID-19 on Routine Laboratory Service Delivery

7. Can you tell me about how routine clinical laboratory service delivery has been affected by the COVID pandemic?

COVID-19 Testing service specific

8. How did the laboratories adapt to scale up COVID-19 testing? (analysers, staff capacity, staff training, standard operating procedures, risk assessments)
9. What challenges did the laboratory face when implementing COVID-19 testing? How were they overcome? What worked well? (e.g. resources, human resource, process change, governance, culture, leadership etc)
10. Which routine services would you envisage will be most impacted moving forwards? (e.g. hospital based-testing, disease specific services, etc) Why?

Recovery post COVID-19

11. Are there any COVID-19-related changes to the laboratory service that you think it would be useful to continue after COVID-19? Which ones and why?
12. What next steps do you believe should be taken now to support the laboratory system to recover post COVID-19?
13. Are there any changes/ innovations introduced in response to COVID-19 changes which you think should be continued? Why?

Thank you

Do you have any questions for me? Resources (re labs) link https://www.rcpath.org/uploads/assets/90111431-8aca-4614-b06633d07e2a3dd9/Guidance-and-SOP-COVID-19-Testing-NHS-Laboratories.pdf

COVID-19 Key Informant Interview Topic Guides

For peer review only

Area D_Protocol

Optimising COVID-19 adaptations for ethical, equitable and quality delivery of essential health services and more resilient health systems

Investigators/research team: Professor Sally Theobald (LSTM); Karsor Kollie (MOH Liberia); Professor Imelda Bates (LSTM); Professor Miriam Taegtmeyer (LSTM); Dr Laura Dean (LSTM); Dr Lucy Frith (UoL). Dr Joanna Raven (LSTM); Dr Rachel Tolhurst (LSTM); Dr Kim Ozano (LSTM); with inputs from Taghreed El-Hajj (LSTM); Yan Ding (LSTM); Natasha Price (LSTM); Helen Piotrowski (LSTM); Russ Dacombe (LSTM); Victoria Watson (LSTM); Rozi McCollum (LSTM); Shahreen Chowdhury (LSTM), Abiola Aiyenigba(LSTM); Rachel Anderson de Cuevas (UoL); Deborah Nyirenda(LSTM/Malawi Wellcome); Nic Desmond (LSTM/Malawi Wellcome).

Research analysis team: Professor Sally Theobald (LSTM); Karsor Kollie (MOH Liberia); Professor Imelda Bates (LSTM); Professor Miriam Taegtmeyer (LSTM); Dr Laura Dean (LSTM); Dr Lucy Frith (UoL); Victoria Watson (LSTM); Rozi McCollum (LSTM).

Terminology

Essential health services: These include services which seek to prevent communicable diseases, to avert maternal and child morbidity and mortality, to prevent acute exacerbations of chronic conditions by maintaining established treatment regimens, and to ensure timely management of emergency conditions (1).

Resilient health system: "the capacity of health actors, institutions, and populations to prepare for ad effectively response to crises; to maintain core functions when a crisis hits; and, informed by lessons learned during the crisis, to reorganise if conditions require it." (2).

Background and Rationale

COVID-19 Overview

As of 24the April 2020, there have been 2,726,194 confirmed coronavirus cases globally, with 191,074 deaths. The UK has had 138,078 confirmed cases and 18,738 deaths, while Liberia has experienced 101 confirmed cases with 8 deaths to date (3). The actual number of cases and COVID-19 related deaths is anticipated to be much higher than those confirmed. Across the world, the COVID-19 pandemic has brought the need for re-structuring of health, social and economic systems. While SARS-CoV-2 does not discriminate, the risks of COVID-19 disproportionately affect vulnerable populations (4).

In the UK, the COVID-19 pandemic has led to the re-organisation and delivery of health services, in order to 1) Free up available resources (health workers, hospital beds, equipment etc.) for the management of COVID-19 patients requiring hospitalisation and 2) Protect patients and staff from unnecessary physical contact, with associated risk of infection.

The WHO has issued guidance surrounding the re-orientation of health services in response to the COVID-19 pandemic and recommends that essential health services include: services needed for essential prevention of communicable diseases; services related to reproductive health; care for vulnerable populations; provision of medications for the ongoing management of chronic disease (including mental health conditions); continuity of critical inpatient therapies; management of emergency health conditions; auxiliary services, including diagnostic imaging, laboratory and transfusion services (1). As health systems adapt and respond to COVID-19, while still delivering services for all citizens, previous experiences from the West African Ebola epidemic encourage us to review whether citizens and health care workers have the opportunity to inform decision-making around adaptations and whether changes introduced are acceptable to health care workers and for those most vulnerable, to engage with the health system when they most need care. Studies from a range of prolonged crises, including economic shocks, climate change disasters, disease outbreak and refugee influx, have revealed that inequalities typically grow during a crisis (5). Seeking to understand and review these adaptations as they roll out, we hope will help identify barriers to patients' use of quality, acceptable health services.

Health systems resilience during crisis

In addition to traditional health systems dimensions such as health and management information, funding mechanisms and health workforce, these factors were found to be shaped by two cross-cutting dimensions 'values and beliefs' and governance (5), see figure 1.

For peer review only - http://bmjopen.bmj.com/site/about/guidelines.xhtml

Figure 1 Learning from shock: a new approach to health systems resilience (Hanefield et al., 2018)

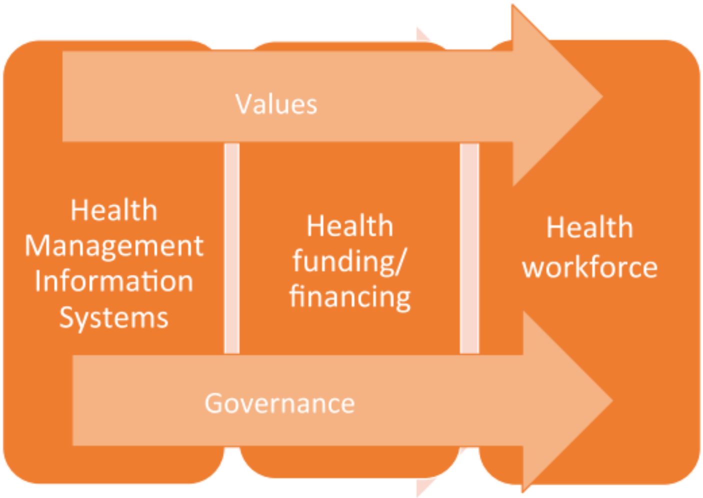

Decision-making

The National Institute for Health and Care Excellence (NICE) has issued guidance algorithms to assist clinicians with decisions surrounding critical care bed admission for patients with COVID-19, however, these do not take into consideration resource limitations or how clinicians should face decisions if the needed resources are not available (6). The British Medical Association (BMA) and Royal College of Physicians (RCP) haves provided briefing notes for health workers, highlighting some of the anticipated ethical dilemmas and providing guidance for workers facing these choices (7,8). While acknowledging the ethically challenging nature of these decisions, health workers are advised of the need to be prepared to modify their practice, so that decisions have a greater focus on public health population ethics, with a basis on utilitarian considerations for how to maximise overall benefit, rather than individual need (7). Health workers have highlighted that this raises the potential for “moral injury”, if they need to make decisions based upon limited availability of resources, rather than need for care (9).

When healthcare services are suspended or reconfigured, how prioritisation decisions are made and the extent to which health workers delivering these services and people using these services are involved with the decision-making process and/or are agreeable with these adaptations are of importance for their acceptability and ultimately whether these services are equitably used (or not). In light of the re-orientation of health services, many clinicians are concerned about their usual patients, including whether these patients are able to access services when needed for both essential emergency and routine care.

Periods of stress, such as conflict and natural disasters, have been shown to increase the occurrence of cardiovascular events (10), with heart attacks and strokes up to two to three times more common during an emergency than in pre-emergency circumstances (11). However, patient use of essential health services has reduced, with many non-emergency services described as ‘eerily quiet’ (12). Within Merseyside, health workers have observed much lower patient attendance to hospital for non-COVID-19 illnesses. Data from New York during the COVID-19 pandemic indicates that cardiac deaths have surged to 400% during the current pandemic compared with the usual number (13). The cause for the underlying increase remains unconfirmed, but is likely to be a combination of undiagnosed COVID19 cases, delayed health care seeking and increased cardio vascular events (CVE) during emergency situation. Early reports in the UK, indicate that patient deaths due to cardiovascular conditions are much higher than anticipated, and thought to relate to patient reluctance to seek health services (14). In light of the increased burden of cardiovascular events alongside COVID-19, the need for comprehensive and acceptable care for patients with chronic diseases is more important than ever.

Despite re-organisation to maximally utilise resources, there is the potential risk that the health system will be stretched beyond capacity, resulting in gaps in availability of personal protective equipment (PPE), intensive care unit (ICU) beds, antigen test for COVID positive patients and appropriately skilled health workers. This brings ethical dilemmas and stress for all affected health workers. Health workers in leadership positions, may bear additional responsibility for some of these decisions, and often feel a weight of responsibility for the wellbeing of more junior staff members.

For peer review only - http://bmjopen.bmj.com/site/about/guidelines.xhtml

Repeated gaps in provision of PPE, leaves frontline health workers faced with the decision of whether to treat patients and risk their own health or refuse to treat patients. Representative groups for frontline health workers, including the BMA and Royal College of Nurses have issued guidance to their members that they should not feel pressured into exposing themselves to unreasonable risk where they have not been provided with appropriate PPE (7,15). Yet, health workers will undoubtedly face immense ethical dilemmas when deciding whether to continue providing care, despite inadequate PPE. Media coverage has highlighted multiple stories about health workers who highlighted the lack of PPE, yet who made the decision to continue to treat their patients and who ultimately died (16).

Restructuring of health services in Merseyside, UK

Within Merseyside, hospital level essential services have been completely restructured and designated coding according to a traffic light system, in order to appropriately cohort care of COVID-19 patients and allow essential services to continue whilst minimising infection of COVID-19 negative patients within the hospital setting. The areas are colour coded as follows:

- White: These areas are designated for patients presenting with no COVID symptoms, for example, patients seeking care for minor injury or for management of chronic care/surgery or ongoing health care needs. Ideally, patients seeking services in these areas would be able to enter directly to the 'white area' without first having to pass through a 'yellow' area.
- Red: These areas are for patients who have tested positive for COVID-19.
- Yellow: This area is for patients with symptoms suspicious of COVID-19, who are awaiting test results, or who have a negative test result but a high level of clinical suspicion that they are COVID-19 positive. Depending on stage of the epidemic about 25 – 50% of patients in a yellow area do not have COVID-19 but are awaiting results (and have potentially been exposed)
- Green: Patients with COVID-19 symptoms who have tested negative in a yellow area, and for who there is low clinical suspicion of COVID-19 and patients who have been treated for COVID-19 are have subsequently been stepped down from a red area. Green patients who develop cough or fever are reswabbed and returned to yellow areas.

Patients moves between areas (as swab results come in or as they recover) are guided by clinical need and coordinated by a patient flow matron and senior infectious disease clinician. Each ward has a designated single colour, although some red/yellow wards have designated individual bays as red or yellow. The emergency department is similarly divided to allow patients in the white category minimum exposure to COVID-19 suspects. All outpatient clinics are regarded as white areas. The hospital has seen a large reduction in white area admissions, has proactively cancelled elective surgery and reduced clinic attendances as well as conducting out-patient consultations by phone/virtual technology.

Delivery of routine essential services

Beyond the hospital setting, there has been re-orientation of essential service delivery, with rapid re-structuring of primary health care services and a shift at hospital and primary care levels towards the use of telemedicine and phone consultations. Cancer care, which often involves both immunosuppressive therapy, tumour resection and inpatient treatment has been disproportionately affected throughout the pandemic (17). Necessary adaptations to service delivery have been made, in order to minimise potential risk of COVID-19 infection, including changes to mode of chemotherapy treatment and use of short-course radiotherapy treatment. For some patient's surgery may be delayed or cancelled (ibid). Many of these modifications may not ultimately affect long-term outcomes. There is the need however, to track and monitor outcomes for these patients, to learn lessons from modifications to usual recommended guidelines in response to COVID-19 adaptations.

Patients with ongoing care needs, include pregnant women needing maternal health care; patients with chronic disease and patients needing chronic cancer care. These patients often need frequent follow-up, including laboratory investigation and availability of blood transfusion services and also experience increased risk of severe illness and death from COVID-19 infection (18). Given the justifiably rapid nature of COVID-19 adaptations, there is the risk that vulnerable patients may become lost to the health system or that quality of care may be compromised in consequence. At this stage it is important to review these decisions, who was involved in these processes, along with their acceptability to health workers and populations, in order to identify and respond to any weaknesses.

For peer review only - http://bmjopen.bmj.com/site/about/guidelines.xhtml

Lessons from past epidemics

Countries in West Africa have recent experience of responding to the Ebola epidemic, with valuable lessons for the UK and beyond in the current COVID-19 pandemic. One lesson was the differing values placed on elements of resilience held by global and national actors, compared with health workers and community leaders. Few of the emergency interventions introduced in response to the Ebola epidemic were designed to promote resilience beyond the immediate crisis (19). While there is an awareness among those planning the COVID-19 response in Merseyside of the need to plan for recovery while responding to the pandemic, there is need to understand and inform how this is implemented.

Another key lesson from the Ebola epidemic relates to the need for the continuation of essential services and to maintain patient confidence in the health system to safely deliver essential services and to control infection risk within the facility (1). During the Ebola epidemic in West Africa substantial reductions in availability and use of critical essential health services were reported, leading to over 80% reductions in maternal delivery care in Ebola - affected areas (20). Analysis suggests that deaths attributable to health systems failures exceeded deaths from Ebola (1). Rapid health systems assessment in Liberia (with possible expansion to other West African settings during phases two and three), will be used to produce guidance for healthcare implementers about good practices.

Our study has been informed by health workers, delivering care within Merseyside for COVID-19 patients and by health decision-makers in Liberia involved with the Ebola response and COVID-19 preparedness planning. This study will seek to identify adaptations to routine essential service delivery introduced in response to COVID-19 pandemic; along with ways to evaluate the impact of these adaptations on routine clinical services in the short and longer term. Innovations and recommendations for improvement by those delivering these services will be identified during phase one, with phases two and three seeking to set up appropriate identified data collection avenues to measure impact of these modifications. This study will endeavour to identify the strengths of these adaptations, which add to the delivery of patient-centred care and should be carried over as the health system transitions beyond the immediate COVID-19 response.

Study phases

This study is anticipated to involve three phases, these will be described in more detail through the protocol (see figure 2 and 3). At this stage REC approval is being sought for Phase one only, in order to facilitate the timely collation and analysis of findings to inform COVID-19 response as it is ongoing.

Approval for phases two and three is not being sought through this REC application, since these phases will be heavily informed by findings generated from phase one. A further amendment will be submitted to the REC prior to commencing these phases. Phases two and three are described here to provide a clearer overview and understanding for phase one within the larger study.

Selection of study sites

Phase one

Merseyside, UK and Liberia were selected for phase one due to established connections (including members of the research team) working within both these regions as part of COVID-19 response efforts. As a result, this research protocol has been informed by health workers directly involved with the COVID-19 response within both contexts. Longstanding research collaboration exists between researchers in LSTM and MOH Liberia. These connections facilitate the research team's ability to start this study, informing practice as soon as possible. In both Merseyside and Liberia, there have been calls for this research to inform and learn from adaptations to essential services introduced as part of the COVID-19 response.

Merseyside, UK is an urban region in the North West of England, with a population of 1.42 million. To date, the North West region of England, which includes Merseyside, has experienced the second highest number of COVID-19 related deaths in the UK outside London (21). The health of people in Liverpool is generally worse than average in England and it is among 20% of the most deprived council areas in England (22). Liverpool City has prioritised tackling deprivation and reducing health inequalities (23). This includes a focus on person-centred care, with integration of health and social care services (ibid). Merseyside region has also established a Resilience Forum, which is a multi-agency partnership of organisations needed to prepare for and respond to any emergency (24).

For peer review only - http://bmjopen.bmj.com/site/about/guidelines.xhtml

Liberia, in West Africa, has a population of 4.8 million (25). Ten years after the conclusion of two civil wars, Liberia was severely affected by the 2014-2015 Ebola epidemic, with more than 10500 cases reported and nearly 5000 deaths (19). This prior epidemic response experience brings considerable lessons of value during the current pandemic response, with Liberia having introduced much more stringent border control measures at a much earlier stage of the current pandemic, in comparison to UK.

Within Merseyside, UK the focus of discussions during phase one about decisions will be at the regional through to health facility level. While in Liberia, the focus of these discussions during phase one will be at national level. The study will be carried out at different levels, due to stakeholders at these levels in each context, having expressed demand and need for this research and thereby, the opportunity to carry out this research and for it to potentially inform COVID-19 response.

While the responses to the COVID-19 pandemic within each context differ considerably, according to the pre-existing health system and external capacities, the level at which the study will be carried out – national in Liberia, compared with regional in Merseyside, there is still learning from past crises which reveals generic factors which can help or hinder the responsiveness of a health system (5).

Possible areas of commonality between contexts include:

1. Evaluations of innovations to routine essential health service delivery: The COVID-19 response has required that service delivery is re-oriented, this has provided the need for innovations which push the boundaries of usual standards of care, yet which may carry greater overall benefit for patients, examples identified by clinicians in Merseyside include: oral chemotherapy; short course of radiotherapy; telephone clinics; starting treatment remotely based on a photo; early discharge; conservative management for things that might have been operated before; dispensing six months (rather than three months) of treatment for HIV or other conditions that are stable. While the innovations will differ between contexts, the need for systematic evaluation of the impact, through identification and monitoring of indicators for success of these innovations is common. In order to provide clarity surrounding whether (or not) these innovations should be continued beyond the COVID-19 response period, with awareness of the need for multiple iterations to develop the optimal restructuring of service delivery.
2. Understanding the human resource management changes and implications associated with the COVID-19 response, with health workers being asked to up-skill to work outside of their usual role; troubling issues faced by health workers; and opportunities for improved human resource management, with a flatter hierarchy and better communication among team members, with greater ability to call out bad practice.
3. Learning lessons about embedding improved practices, introduced and reinforced through the COVID-19 response, such as improved cleanliness and hygiene within healthcare settings. Exploring lessons surrounding how to maintain these practices beyond the COVID-19 response.

Phases two and three

During phase two and three a selection of up to two counties in Liberia will be selected for further discussions about sub-national decision-making. These counties will be identified in consultation with national decision-makers and an effort to include one urban and one rural county will be made, to bring differing perspectives.

In addition, depending on findings from phase one and expression of interest in the research study in other settings, there is the possibility that an additional region within UK and/ or another country may be added during phases two and three. This will be fully described in an additional amendment prior to starting phase two.

Aim

To assess the impact of adaptation of health systems as a result of COVID-19 in Merseyside, UK and Liberia and produce guidance for essential service delivery during the crisis and to promote stronger health systems in the immediate recovery phase and beyond.

Objectives

This study will work towards achieving three main objectives:

For peer review only - http://bmjopen.bmj.com/site/about/guidelines.xhtml

Objective 1

To evaluate and support decision-making processes for essential service delivery, including communication and implications of these for the health workforce during periods of health systems adaptation.

Objective 2

To understand the impact of COVID-19 adaptations on equity and quality of routine health care delivery, including laboratory and blood transfusion services.

Objective 3

To document cross-context learnings and innovations (Merseyside, UK and Liberia) of necessary health systems adaptation to maintain essential service delivery and to develop recommendations of best practices during times of crisis.

Research Question

What opportunities are there to support decision-making; to strengthen essential service delivery through health systems adaptations brought about in response to COVID-18 pandemic in the recovery period and beyond?

Study Design and Phases

A three-phase study will seek to respond to the three study objectives using an iterative and layered approach (see figures 2 and 3). This protocol provides an overview of the study methodology for all phases of the study. However, details for the methods, ethical considerations, tools and participant information sheets are detailed for phase one of the study only, since subsequent phases will be informed by findings from phase one. An amendment to this ethics application, including these details, will be submitted at a later date.

Figure 2 Study Timeline

Year	2020							2021												2022				
Month	Jun	Jul	Aug	Sep	Oct	Nov	Dec	Jan	Feb	Mar	Apr	May	Jun	Jul	Aug	Sep	Oct	Nov	Dec	Jan	Feb	Mar	Apr	May
Phase 1																								
Phase 2																								
Phase 3																								

Phase one

Phase one seeks to provide preliminary findings and early guidance with the aim of informing and strengthening resilience within the health system during the COVID19 response (see figure 3). Since the objectives have a wide-ranging scope, potentially encompassing the whole health system, phase one will maintain a relatively 'broad brush' approach. Through this approach we intend to identify tracer conditions, health systems levels, focal areas and participants to study in greater depth during phases two and three.

Methodology for phase one will include a combination of primary and secondary data. Secondary data will involve international literature review and review of key documents in UK and Liberia (see below).

Primary data collection for phase one will focus around key informant interviews with a wide range of health care workers and decision-makers in Merseyside, UK and with informants from the preventive services section of the MOH Liberia.

Questioning within interviews will explore key areas of health systems functioning including: governance and decision making; which ethical guidelines are used, and how and if these ethical guidelines are operationalised at the frontline; human resource management and health care worker support; how innovations are started and evaluated for impact; and perceptions of the equity and quality of service delivery.

For peer review only

For peer review only - http://bmjopen.bmj.com/site/about/guidelines.xhtml

Table 1 Key research questions and methods for each study objective

Objective	Research Questions	Methods
1. To evaluate and support communication of decision-making processes for essential service delivery and implications of these for the health workforce during periods of health systems adaptation.	What is the nature of decisions being made by health workers, particularly those providing routine essential care?<br>What informs these decisions?<br>What is the nature of decisions being made by regional/ national health decision-makers?<br>How are they communicated?<br>What added support do health workers and decision-makers want to guide these decisions?<br>What are the lessons learned from EVD that are being taken forward to the Liberian COVID-19 response planning? What is similar, what is different?	Key informant interviews<br>Document and literature review
2. To understand the impact of COVID-19 adaptations on equity of essential health care delivery, including laboratory and blood transfusion services.	What has worked well?<br>What is the extent of adaptations to routine essential service delivery?<br>How have these adaptations affected patient care?<br>Whose needs are met/unmet? Why?<br>What changes are needed to COVID-19 adaptations to improve use of services according to need?	Key informant interviews<br>Document and literature review
3. To document cross-context learnings and innovations (Merseyside, UK and Liberia) of necessary health systems adaptations to maintain essential service delivery and to develop recommendations of best practices during times of crisis.	What are the key lessons learned about health systems adaptation in response to a disease outbreak?<br>How are decision makers in Liberia planning to address these through their COVID-19 response?	Key informant interviews<br>Document and literature review

Phase two: Phase two will provide a more in-depth study to gain greater depth of understanding of focal areas within the objectives, through in-depth interviews with a targeted, smaller range of interviewees including patients with chronic care and/ or ongoing care needs (e.g. maternal health care/ chronic disease/ chronic cancer care). Phase two may also involve more focus on a particular health system level, e.g. primary health care level or secondary care level, depending on findings from phase one, which includes a broad range of participants across health systems levels. It is hoped that phase two will also involve the tracking of indicators to measure the impact of selected adaptations, identified through phase one.

Other forms of data collection (such as use of existing health systems data; health worker audio diaries; observations and document analyses) may be employed for data collection during phase two, depending on findings from phase one.

Phase three: The third and final phase is an optional phase, subject to securing additional funding, which will seek to provide and consolidate lessons learned through the COVID-19 response to utilise the ‘window of opportunity’ in the period immediately following the COVID-19 pandemic response to strengthen universal health coverage,

For peer review only - http://bmjopen.bmj.com/site/about/guidelines.xhtml

either through successes of the COVID-19 adaptations studied through phases one and two; or to propose recommendations for change based on weaknesses or challenges associated with these adaptations.

Figure 3 Study overview

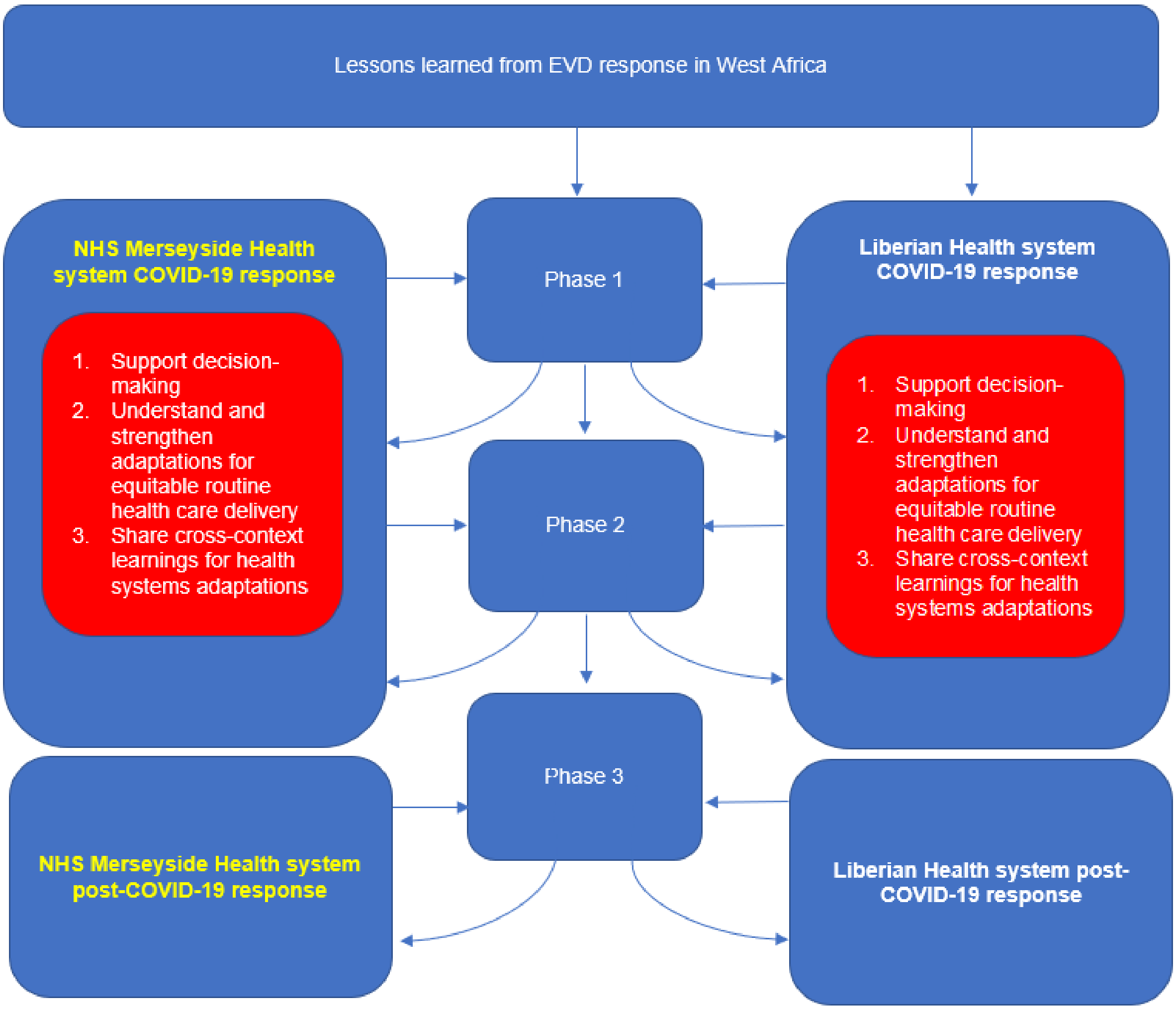

only

Overview of methods for phase one

Literature review

A rapid appraisal of the literature will be carried out to review the literature published regarding lessons for epidemic and pandemic response and the provision of services throughout a response. It is anticipated that much of this literature will originate from the West African Ebola epidemic.

Due to the need for rapidity within the process, peer reviewed literature will be reviewed, with addition of grey literature as time allows. We will develop a search strategy including search terms, search methods and possible databases. Findings from the literature review will be used to guide the formation of a thematic framework for the study. Literature review findings will be summarised into a brief report.

Key documents review

A rapid review of guidance documents available to frontline workers and decision-makers to assist them in their care for patients and in the re-orientation and adaptation of health service will be carried out. This will focus primarily on WHO, MOH Liberia UK government and NHS guidance (1,26). Key documents will be identified

For peer review only - http://bmjopen.bmj.com/site/about/guidelines.xhtml

through online searches; COVID-19 health worker training sites (27), through key informant interviews and by contacting relevant individuals via phone, skype or email. Once documents are obtained, these will inform the development of a thematic framework.

Key Informant Interviews

Within Merseyside primary data collection will focus around 30-55 key informant interviews with a wide range of health care workers involved directly in the COVID-19 health systems adaptations within the UK NHS and those working in other key areas of the health system, for example senior health officials involved with decision making, laboratory personnel, and health staff working on ensuring the continued provision of essential health services, including maternal health care, chronic cancer care and chronic disease management. Within Merseyside key informants will include – regional decision-makers; NHS managers; outpatient clinic staff; IT service staff supporting telemedicine clinics; health workers providing essential health services and laboratory and blood transfusion workers, due to the recent massive push to increase capacity for testing for COVID-19.

In Liberia, key informants will include 15-20 informants from the preventive services section of the MOH Liberia. These informants will include national level directors for key service delivery programs, such as health promotion, family health, NCD program, community health and laboratory and transfusion services, who played key roles in the Ebola epidemic response and who are involved with COVID-19 response planning.

The research team includes researchers who currently work within the UK and Liberia as health workers; NHS laboratory personnel and as members of the public health commission in the UK, in addition to their role as researchers. Liberian colleagues who played instrumental roles in the Ebola response during 2014-2015, and who are presently involved with guiding decision-making for Liberia's COVID-19 response, have been involved with discussions as part of the development of this research protocol.

During phase one, the researchers' existing knowledge of the health system, key individuals leading the response and suitable participants in UK and in Liberia will be used to identify potential participants. Where gaps in understanding persist, a snowball approach will be adopted to identify other participants with key understanding of the study objectives.

Participants will initially be contacted by a member of the research team, where the participant is already known to one of the researchers to introduce the study and to request their participation in the study. Where the participant is not known to the researcher, but is identified through a snowball approach, the known contact of the participant will be requested to seek permission from the participant for their contact details to be shared with the research team.

If the participant is agreeable to participate, and with their permission, their contact details will then be shared with a member of the research team, who will contact the participant by phone and or email to establish a time suitable to the participant for the interview.

Participants will be provided with the participant information about the study via email at least 24 hours before the scheduled time for the phone interview, to allow time for the participant to review the consent form and learn more about recording of the interview (28), please see research toolkit for sample participant information sheets.

Due to physical distancing, interviews will be carried out via phone or skype. As part of the introduction to the research, the researcher will request permission from the interviewee to audio record the consent process (ibid) as well as the content of the interview (see research toolkit for initial participant introduction, including permission to record consent). Should skype be used the participant will be given to use the video option or not, depending on which they feel most comfortable with (ibid). Participants will also be offered the opportunity to respond via email if they prefer.

The consent process will provide an overview of the study, why the participant has been invited to take part, the voluntary nature of participation, option to refuse to participate at any point with no negative consequences, what participation will involve including anticipated length of interview, reimbursement, possible disadvantages and benefits from participation, option to withdraw at any point, confidentiality and data management overview, how the research will be used, data protection and who to contact for further questions.

For peer review only - http://bmjopen.bmj.com/site/about/guidelines.xhtml

Data from phase one will be used to identify suitable participants for phase two of the study. It is currently unknown exactly who will participate in phase two of the study, although it is likely that both health workers and patients will take part. Details for recruitment and informed consent of phase two participants will be shared in a study amendment, following preliminary analysis of phase one data.

Participants will be encouraged to share any suggestions or recommendations they have for making improvements to the COVID-19 response and to improve health services in the immediate recovery period and beyond.

Table 2 Phase one study participants – removed for anonymity

Anticipated Study Methods for Phase Two

Findings from phase one may lead to modification or revision of the study objectives in response to the identified weaknesses or the recognised strengths and innovations of the COVID19 adaptations employed to date.

Phase two will provide a more in-depth study to gain greater depth of understanding of focal areas within the objectives, through in-depth interviews with a targeted, smaller range of interviewees including patients with chronic and/or ongoing care needs (e.g. maternal health care). Since it will not be feasible to explore the effect of COVID-19-related ethics and adaptations on every clinical discipline, we will study in more depth the chronic/ ongoing 'tracer' conditions identified through phase one that reflect different aspects of the health system (these may vary across contexts). Tracer conditions may include maternal health, since it cannot be delayed, it has a pre-set schedule, and learning from other health systems shocks such as Ebola show us that maternal mortality can be negatively impacted during these periods. Breast cancer care may be another possible tracer condition, since it is an excellent marker of a well-functioning health system testing screening, referral and rapid access clinic. There is clinical data available, which could be used as indicators to monitor this. Other tracer conditions will be identified through phase one. Once health systems adaptations and related tracer conditions have been identified options to evaluate the impact of these adaptations on the tracer condition will be explored. This may involve use of existing health data.

In addition, other forms of data collection (such as health worker audio diaries, observations and document analyses) may be employed for data collection during phase two, depending on findings from phase one.

Health worker audio diaries/ review of social media may be employed to gain a more detailed understanding of the everyday practices and reflections of health workers involved with COVID-19 response and in the provision of essential health services. If this method is used health workers will be asked to participate in diary keeping by a record through written and/or videos or voice memos about their everyday practices. The researchers role can be to provide participants with questions or prompts to direct their recordings and documentation (29). These methods may be combined with traditional in-depth interviews to follow up on findings from the diary/ video elicitation methods.

Observation may also be employed as a method. Given the need for physical distancing, this may be incorporated within the health worker diaries, with health workers trained on the use of a structured tool to reflect on their observations about their work over a certain period as part of diary keeping.

Anticipated Study Methods for Phase Three

The third and final phase is an optional phase, subject to securing additional funding, which will seek to provide and consolidate lessons learned through the COVID-19 response to utilise the 'window of opportunity' in the period immediately following the COVID-19 pandemic response to strengthen universal health coverage, either through successes of the COVID-19 adaptations studied through phases one and two; or to propose recommendations for change based on weaknesses or challenges associated with these adaptations.

Data Analysis for Phase One

Due to the need for rapid data collection and analysis, with recommendations and guidance issued as the COVID-19 response is ongoing, along with many of the researchers involved carrying out this work in addition to their usual workload, data analysis will be led by a smaller group of researchers (see research analysis team above), with the

For peer review only - http://bmjopen.bmj.com/site/about/guidelines.xhtml

broader team involved throughout. The whole research team will be invited to review and provide comments and suggestions throughout the data analysis process.

Literature and document review

Initial findings from the literature and document reviews will be used to develop a thematic framework, which speaks to the research objectives. This framework will then be used to guide the full data collation and analysis from the literature and document reviews.

Qualitative data

Interviews with decision-makers in Liberia will identify how they intend to use lessons from the EVD response while implementing their COVID-19 response. This is will be triangulated with findings from interviews with health workers in Merseyside surrounding 1) Decision-making for COVID-19 and 2) Equity and quality of essential service provision amidst COVID-19 adaptations of the health system.

Interviews will be audio-recorded, notes will be taken during and immediately following the interview. During phase one, full transcription will not be carried out, in order to ensure timeliness of analysis and rapid development and issuance of guidance to inform the COVID-19 response. Rather, several researchers will quality check that notes taken accurately capture the content and main discussion points of the interview. Interview notes will then be analysed thematically using a framework approach, as described below (30). Several researchers will initially take the lead with the analysis of the data, while all researchers will have opportunity to review, comment and provide suggestions as part of the analytical process. Data will be shared between researchers using a password protected file within dropbox business, or alternatively through the next cloud platform (hosted by LSTM). Audio recordings will be deleted after checking following transcription/ extensive note taking, in order to preserve anonymity. Data will be stored for seven years. Consent forms and/or participant names will be kept separately from other files in order to protect anonymity.

Stage 1: Data Management

Step 1: Familiarisation - the analysis team will read and re-read the notes to 'familiarise' themselves with the data. Whilst doing this they take note of key themes emerging.

Step 2: Iterative revision of thematic/coding framework initially developed through literature review - a framework through which to sort the data will be developed based on original aims and objectives and any inductive themes identified during the familiarisation process.

Step 3: Indexing/coding data - the thematic/coding framework will be applied to all the data. This will be assisted by the use of NVIVO software.

Stage 2: Data Explanation

Step 4: Charting - data will be lifted from its original context based on its allocation to the coding/thematic framework and placed within a chart.

Step 5: Mapping - the final stage of the process will be to interpret, and map the range of polarities and similarities within the data.

Qualitative analysis software NVIVO 12 PRO will be used to support data management and analysis.

Outcomes

Through this study we hope to contribute towards the following outcomes:

Outcome 1: Pragmatic guidance and support for decision-makers and health workers making decisions about communication of decisions and the implications for the health workforce.

Outcome 2: Evidence about the felt impact of COVID-19 health system adaptations by health workers delivering these and recommendations to promote equity and quality within these.

Outcome 3 (phase 2): Evidence about patient perceptions of equity and quality of services, following adaptations in response to COVID-19 and recommendations to promote equity and quality within these.

For peer review only - http://bmjopen.bmj.com/site/about/guidelines.xhtml

Outcome 4 (phase 2 and 3): Resource documents guiding how to prioritise equity and quality and sustain resilience into the immediate recovery phase.

Outcome 5 (phase 2 and 3): Tracer condition and indicators identified to assess impact assess health systems adaptation identified.

Outcome 6: Recommendations for health systems adaptations which support universal health coverage throughout times of crisis.

Dissemination

Since phase one is a rapid appraisal during which we plan to identify rapid lessons to inform and strengthen the COVID-19 response while it is ongoing, there will necessarily be an early preliminary dissemination of research findings. The details of this have not yet been finalised. This may take the form of recorded teaching session made available on various platforms, such as LSTM website, global health network; a learning brief or other form of dissemination and will be informed by phase one findings, where participants are asked to identify the type of additional support which is needed. It is intended that the findings will be shared both with the study participants and also made available for all relevant colleagues, e.g. health workers involved with decision-making. Dissemination of findings will be tailored if necessary, according to the context Merseyside vs Liberia and to the audience, e.g. doctors, nurses vs laboratory personnel.

Following phase two and three of the research study findings will again be shared with study participants and their colleagues, according to their role.

Ethical approvals

Sponsorship and REC approval will be sought from LSTM, REC approval from University of Liverpool, Confirmation of Capacity from Research, Development and Innovation at Royal Liverpool Hospital (via SPARK).

Potential risks, adverse effects, discomfort or risks and how these will be mitigated

Since this study will involve interviews with frontline health workers, who are already expected to attend many additional meetings, there is the potential risk that the study will add to their workload. To mitigate this, every effort will be made by the researchers to accommodate the health worker's timeframe, with interviews scheduled according to the health workers availability. Questions will be reviewed and prioritised to ensure that interviews with frontline health workers are kept as short as possible, preferably less than 45 minutes per interview. Researchers will be open with participants about the timeframe needed for the interview. Additionally, it will be made clear to participants that they do not have to take part if they do not wish to do so and that non-participation will not bring any negative consequences. Interviews will be carried out online/ by phone which may limit the opportunity for reassurance involved during face-to-face interviews.

Remembering and describing particular patient stories, or issues around PPE as part of the discussion about ethics and the dilemmas involved with decision-making, may be traumatic for some health workers. Some participants may describe burn out and mental health issues. All interviewers involved with carrying out interviews are trained and experienced qualitative researchers. The interviewee will be advised as part of the consent process that he/she can pause or end the interview at any stage. In addition, the interviewer will refer the participant to the NHS mental health hotline for staff tackling COVID-19 if felt to be needed. In Liberia, Links will be made to relevant support services, including the MOH Mental Health team and the Carter Center, if needed.

Participants may disclose ethical issues surrounding unsafe practices. If unsafe practices are disclosed which identify that patients are being put at risk then this would be reported to the study principal investigator, NHS research and development forum, and the ethics board for further action in the UK. In Liberia, this would be reported to the relevant MOH Liberia actor. Participants will be advised of this as part of the consent process.

In light of recent 'gagging' of health workers regarding speaking out about lack of PPE, some health workers may fear repercussions for highlighting challenges experienced. As part of the consent process participants will be advised of the importance of confidentiality. Unless the participant gives additional specific consent to be identified (see consent form in Area D_Toolkit), all data collected within interviews will be anonymized in reports and publications.

For peer review only - http://bmjopen.bmj.com/site/about/guidelines.xhtml

Potential risk of transmission of COVID-19 during the interview. Interviews will be carried out via phone/skype in order to reduce risk of transmission of COVID-19.

References

1. World Health Organization. Operational guidance for maintaining essential health services during an outbreak. 2020.
2. Kruk ME, Myers M, Varpilah ST, Dahn BT. What is a resilient health system? Lessons from Ebola. Lancet [Internet]. 2015;385(9980):1910–2. Available from: http://linkinghub.elsevier.com/retrieve/pii/S0140673615607553
3. Worldometer. Worldometer Coronavirus cases [Internet]. 2020 [cited 2020 Apr 24]. Available from: https://www.worldometers.info/coronavirus/#countries
4. Berger ZD, Evans NG, Phelan AL. Covid-19 : control measures must be equitable and Failure to respect the needs of vulnerable groups will seriously undermine response efforts. BMJ [Internet]. 2020;1141(March):19–20. Available from: http://dx.doi.org/doi:10.1136/bmj.m1141
5. Hanefeld J, Mayhew S, Legido-quigley H, Martineau F, Karanikolos M, Blanchet K, et al. Towards an understanding of resilience : responding to health systems shocks. Heal Policy P. 2018;33(January):355–67.
6. National Institure for Health and Care Excellence. COVID-19 rapid guideline : critical care in adults (Last update : 27 March 2020) [Internet]. 2020. p. 2020. Available from: https://www.nice.org.uk/guidance/ng159/resources/critical-care-admission-algorithm-pdf-8708948893
7. British Medical Association. COVID-19 – ethical issues . A guidance note [Internet]. 2020. Available from: https://www.bma.org.uk/media/2226/bma-covid-19-ethics-guidance.pdf
8. Royal College of Physicians. Ethical dimensions of COVID-19 for frontline staff 7. 2020.
9. Wingfield T, Taegtmeyer M. Healthcare workers and coronavirus: behind the stiff upper lip we are highly vulnerable. The conversation.
10. Hayman KG, Sharma D, Singh S. Burden of cardiovascular morbidity and mortality following humanitarian emergencies : a systematic literature review. Prehosp Disaster Med. 2016;30(1):80–8.
11. United Nations. NONCOMMUNICABLE DISEASES IN EMERGENCIES WHAT ARE NONCOMMUNICABLE DISEASES AND WHY ARE THEY IMPORTANT IN EMERGENCIES ?
12. Krumholz H. Where Have All the Heart Attacks Gone? The New York Times [Internet]. 2020 Apr 6; Available from: https://www.nytimes.com/2020/04/06/well/live/coronavirus-doctors-hospitals-emergency-care-heart-attack-stroke.html
13. NBC New York. Massive Spike in NYC 'Cardiac Arrest' Deaths Seen as Sign of COVID-19 Undercounting. NBC New York [Internet]. 2020 Apr 10; Available from: https://www.nbcnewyork.com/news/local/massive-spike-in-nyc-cardiac-arrest-deaths-seen-as-sign-of-covid-19-undercounting/2368678/
14. Campbell D, Marsh S, Johnson S. Warning as UK coronavirus outbreak leads to sharp rise in deaths at home. The Guardian [Internet]. 2020 Apr 16; Available from: https://www.theguardian.com/society/2020/apr/15/sharp-rise-in-ill-patients-dying-at-home-since-coronavirus-outbreak?utm_term=RWRpdG9yaWFsX0d1YXJkaWFuVG9kYXlVS19XZWVrZGF5cy0yMDA0MTY%3D&utm_source=esp&utm_medium=Email&CMP=GTUK_email&utm_campaign=GuardianTodayUK
15. Knapton S, Yorke H. Nurses told they can refuse to treat coronavirus patients unless they have proper PPE. The Telegraph [Internet]. 2020 Apr 12; Available from: https://www.telegraph.co.uk/news/2020/04/12/nurses-told-can-refuse-treat-coronavirus-patients-unless-have/

For peer review only - http://bmjopen.bmj.com/site/about/guidelines.xhtml

16. BBC News. Coronavirus: NHS doctor who pleaded for PPE dies. 2020 Apr 10; Available from: https://www.bbc.co.uk/news/uk-england-london-52242516

17. Rosenbaum L. Medicine a nd Society The Untold Toll — The Pandemic ' s Effects on Patients without Covid-19. N Engl J Med. 2020;1–4.

18. Zhou F, Yu T, Du R, Fan G, Liu Y, Liu Z, et al. Clinical course and risk factors for mortality of adult inpatients with COVID-19 in Wuhan , China : a retrospective cohort study. Lancet [Internet]. 2020;395(10229):1054–62. Available from: http://dx.doi.org/10.1016/S0140-6736(20)30566-3

19. Ling EJ, Larson E, Macauley RJ, Kodl Y, Vandebogert B, Baawo S, et al. Beyond the crisis : did the Ebola epidemic improve resilience of Liberia ' s health system ? 2017;40–7.

20. Elston JWT, Cartwright C, Ndumbi P, Wright J. The health impact of the 2014 - 15 Ebola outbreak. Public Health [Internet]. 2016;143:60–70. Available from: http://dx.doi.org/10.1016/j.puhe.2016.10.020

21. BBC. Coronavirus UK map. BBC News [Internet]. 2020 Apr 23; Available from: https://www.bbc.co.uk/news/uk-51768274

22. Liverpool City Council. Liverpool City Council: public health transformation six years on [Internet]. 2019. Available from: https://www.local.gov.uk/liverpool-city-council-public-health-transformation-six-years

23. Mayor of Liverpool. A STRONG AND GROWING CITY BUILT ON FAIRNESS. 2018.

24. Merseyside Prepared. Merseyside Resilience Forum [Internet]. 2020. Available from: https://merseysideprepared.org.uk/about-us/

25. The World Bank. The World Bank Data Liberia [Internet]. 2018 [cited 2020 Apr 24]. Available from: https://data.worldbank.org/country/liberia

26. World Health Organization [WHO]. Guidance for Managing Ethical Issues in Infectious Disease Outbreaks. 2016.

27. e-Learning for Healthcare. Coronavirus e-learning [Internet]. 2020 [cited 2020 Apr 24]. Available from: https://www.e-lfh.org.uk/programmes/coronavirus/

28. FUTUREGOV. Making design research work remotely [Internet]. 2020. Available from: https://docs.google.com/presentation/d/1T0sjyDlvCRJG95q9LYmPiPaU8baLG7L2IQwRpNsfq2M/edit#slide=id.g4e808df0f1_2_275

29. Lupton D. DOING FIELDWORK IN A PANDEMIC [Internet]. 2020. Available from: https://docs.google.com/document/d/1clGjGABB2h2qbduTgfqribHmog9B6P0NvMgVuiHZCl8/edit?ts=5e88ae0a#

30. Ritchie J, Lewis J, Nicholls CM, Ormston R. The Foundation of Qualitative Research. Qual Res Pract A Guid Soc Sci Students Res. 2013;0–25.

For peer review only - http://bmjopen.bmj.com/site/about/guidelines.xhtml

BMJ Open

Qualitative study exploring lessons from Liberia and the UK for building a people-centred resilient health systems response to COVID-19

Journal:	*BMJ Open*
Manuscript ID	bmjopen-2021-058626.R1
Article Type:	Original research
Date Submitted by the Author:	25-Apr-2022

Complete List of Authors:	McCollum, Rosalind ; Liverpool School of Tropical Medicine, International Public Health<br>Zaizay, Zeela ; Actions Transforming Lives<br>Dean, Laura; Liverpool School of Tropical Medicine, Department of International Public Health<br>Watson, Victoria; Liverpool School of Tropical Medicine (LSTM), International Public Health<br>Frith, Lucy; The University of Manchester, Centre for Social Ethics & Policy, School of Law<br>Alhassan, Yussif; Liverpool School of Tropical Medicine, International Public Health<br>Kollie, Karsor; Ministry of Health<br>Piotrowski, Helen; Liverpool School of Tropical Medicine, Department of International Public Health<br>Bates, Imelda; Liverpool School of Tropical Medicine<br>Anderson De Cuevas, Rachel; University of Liverpool, Institute of Population Health<br>Harris, Rebecca ; University of Liverpool, Institute of Population Health<br>Chowdhury, Shahreen; Liverpool School of Tropical Medicine, International Public Health<br>Berrian, Hannah; University of Liberia-Pacific Institute for Research and Evaluation<br>Smith, John; University of Liberia-Pacific Institute for Research and Evaluation<br>Tate, Wede; University of Liberia-Pacific Institute for Research and Evaluation<br>El Hajj, Taghreed; Liverpool School of Tropical Medicine<br>Ozano, Kim; Liverpool School of Tropical Medicine<br>Hastie, Olivia; Liverpool School of Tropical Medicine<br>Parker, Colleen; Ministry of Health Liberia<br>Kollie, Jerry; University of Liberia-Pacific Institute for Research and Evaluation<br>Zawolo, Georgina; University of Liberia-Pacific Institute for Research and Evaluation<br>Ding, Yan; Liverpool School of Tropical Medicine, Centre for Capacity Research<br>Dacombe, Russell; Liverpool School of Tropical Medicine, International Public Health<br>Taegtmeyer, Miriam; Liverpool School of Tropical Medicine, International

	Public Health<br>Theobald, Sally ; Liverpool School of Tropical Medicine, International Public Health
**Primary Subject Heading**:	Global health
Secondary Subject Heading:	Health services research, Health policy
Keywords:	COVID-19, HEALTH SERVICES ADMINISTRATION & MANAGEMENT, PUBLIC HEALTH, QUALITATIVE RESEARCH

SCHOLARONE™
Manuscripts

I, the Submitting Author has the right to grant and does grant on behalf of all authors of the Work (as defined in the below author licence), an exclusive licence and/or a non-exclusive licence for contributions from authors who are: i) UK Crown employees; ii) where BMJ has agreed a CC-BY licence shall apply, and/or iii) in accordance with the terms applicable for US Federal Government officers or employees acting as part of their official duties; on a worldwide, perpetual, irrevocable, royalty-free basis to BMJ Publishing Group Ltd ("BMJ") its licensees and where the relevant Journal is co-owned by BMJ to the co-owners of the Journal, to publish the Work in this journal and any other BMJ products and to exploit all rights, as set out in our licence.

The Submitting Author accepts and understands that any supply made under these terms is made by BMJ to the Submitting Author unless you are acting as an employee on behalf of your employer or a postgraduate student of an affiliated institution which is paying any applicable article publishing charge ("APC") for Open Access articles. Where the Submitting Author wishes to make the Work available on an Open Access basis (and intends to pay the relevant APC), the terms of reuse of such Open Access shall be governed by a Creative Commons licence – details of these licences and which Creative Commons licence will apply to this Work are set out in our licence referred to above.

Other than as permitted in any relevant BMJ Author's Self Archiving Policies, I confirm this Work has not been accepted for publication elsewhere, is not being considered for publication elsewhere and does not duplicate material already published. I confirm all authors consent to publication of this Work and authorise the granting of this licence.

For peer review only

Title Qualitative study exploring lessons from Liberia and the UK for building a people-centred resilient health systems response to COVID-19

Authors: Rosalind McCollum*^, Zeela Zaizay*, Laura Dean, Victoria Watson, Lucy Frith, Yussif Alhassan, Karsor Kollie, Helen Piotrowski, Imelda Bates, Rachel Anderson De Cuevas, Rebecca Harris, Shahreen Chowdhury, Hannah Berrian, John Smith, Wede Tate, Taghreed El Hajj, Kim Ozano, Olivia Hastie, Colleen Parker, Jerry Kollie, Georgina Zawolo, Yan Ding, Russell Dacombe, Miriam Taegtmeyer, Sally Theobald

*Joint first authors

^Corresponding author email address: rosalindmccollum@gmail.com

For peer review only

For peer review only - http://bmjopen.bmj.com/site/about/guidelines.xhtml

Abstract

Introduction: COVID-19 has tested the resilience of health systems globally and exposed existing strengths and weaknesses. This study uses the concept of people-centred health systems to explore the applicability of the Foreign, Commonwealth and Development Office (FCDO) principles for health systems' resilience in two contrasting contexts – Liberia and UK.

Methods: We carried out qualitative interviews with 24 health decision-makers at National and County Level in Liberia and 42 actors at County and hospital level in the UK (Merseyside). We explored health systems' decision-making processes and capacity to adapt and continue essential service delivery in response to COVID-19 in both contexts.

Results: Study respondents in Liberia and Merseyside had similar experiences in responding to COVID-19, despite significant differences in health systems context, and there is an opportunity for multi-directional learning between the global south and north. The need for early preparedness; strong community engagement; clear communication within the health system, and health service delivery adaptations for essential health services emerged strongly in both settings. We found the FCDO principles to have value as a framework for reviewing health systems changes, across settings in response to a shock such as a pandemic. In addition to the eight original principles, we identify two additional principles; 1) the need for functional structures and mechanisms for preparation and 2) adaptable governance and leadership structures to facilitate timely decision-making and response coordination. We find the use of a people-centred approach also has value to prompt policy makers to consider the acceptance of service adaptations by, patients and health workers, and to continue the provision of 'routine services' for individuals during health systems shocks.

Conclusion: Our study highlights the importance of a people-centred approach, placing the person at the centre of the health system, and value in applying and adapting the FCDO principles across diverse settings.

For peer review only - http://bmjopen.bmj.com/site/about/guidelines.xhtml

Strengths and Limitations of the Study

- A key strength of this study is the multi-directional learning between health systems in the global south and global north, which involved a wide range of researchers across both settings, and the breadth of perspectives captured from frontline staff and key decision-makers.
- We find that the FCDO principles can be usefully applied across diverse contexts, with identification of two additional new principles, related to mechanisms for advanced preparedness and adaptable governance and leadership structures.
- The greatest limitation of this study is that it was carried out at a single point in time, towards the end of the first wave in the UK and before there had been a large increase in cases in Liberia. Response measures have evolved in both settings in subsequent stages of the pandemic.
- The study was limited by the differing range of respondents across study settings, with participants from across a range of health system levels including primary care, hospital frontline workers and decision-makers as well as regional decision-makers within Merseyside, UK; compared with national and county level decision-makers, technicians and supervisors of frontline staff in Liberia, which may result in differing perspectives.

For peer review only - http://bmjopen.bmj.com/site/about/guidelines.xhtml

For peer review only

Introduction

The COVID-19 pandemic has forever altered our world. Its impact has been felt across all nations, demonstrating the importance of resilient health systems in protecting global health security.[1] Health systems have been forced to adapt to new ways of working alongside the continued provision of essential services including: prevention of communicable diseases; sexual and reproductive health; care for vulnerable populations; ongoing management of chronic illness (including mental health conditions); continuity of critical inpatient therapies; management of emergency health conditions; and auxiliary services, including diagnostic imaging, laboratory and transfusion services.[2]

In April 2020, the United Nations expressed concern that, within Africa, up to 3.3 million people could lose their lives as a direct result of COVID-19 and many more through the indirect effects of disruption to health services and worsening socioeconomic conditions.[3] Conditions considered to increase the risk of infection include overcrowded and poorly serviced slum dwellings; limited access to basic handwashing facilities; high levels of informal employment limiting ability to work from home; high levels of malnutrition and lower ratios of beds and health workers to the population.[3] A commentary published by Agyeman et al. (2020) at the outset of the pandemic highlighted a rapid response within many African settings, including focus on early introduction of screening procedures at ports of entry, need for effective community engagement to educate about the mode of transmission. Key protective behaviours were emphasised, along with the need to prepare intensive care beds and clear government strategies regarding how to deal with hospitalised COVID-19 patients to avoid disrupting the health system and to prevent non-COVID-19 related deaths.[4] Subsequent studies have revealed that indirect health impacts from COVID-19 disproportionately impact women and children.[5,6] Diversion of resources (financial, material, human) from existing health services to address the pandemic, impacts their care.[5,6] This includes supply and demand side disruptions that can result in lower utilization of health care and, in some cases, impact on quality of care.[7] Bayani et al (2021) surmise that "less

For peer review only - http://bmjopen.bmj.com/site/about/guidelines.xhtml

health care will result in more ill health and deaths because health services have been suspended, displaced, or inaccessible."(page 5 [7])

Our study was carried out immediately following the first wave of COVID-19 in Liberia and UK (interviews carried out June to September 2020) in response to an expressed need by stakeholders for this research following dialogue in both contexts. The study was conducted within these two contexts (Merseyside region and Liberia) based on strong prior research relationships within both settings. The differing perspectives from national and county respondents speaking on the national response in Liberia, and frontline health workers and decision makers up to regional level in Merseyside based on their personal experiences and more localised regional response is a key limitation. We chose these settings due to the opportunity and demand for research, not because they are exemplars of COVID-19 response. There is, however, still opportunity for learning and comparison on both the strengths and weaknesses within the COVID-19 initial response in both settings. The pandemic has continued to evolve across both settings, with both Liberia and UK experiencing much larger waves of COVID-19 since this original study was carried out. These findings from the first wave can provide valuable lessons to inform continued response to COVID-19 and other health systems shocks.

The pandemic has revealed monopolies of knowledge production, which disempower lower and middle-income countries;[8] whilst pandemic responses in 'developed democracies' have been inadequate, with cuts to health and social services and limited commitment to equity or governance.[8] So-called "global powerhouses with tried and tested health systems have struggled to contain the COVID-19 pandemic"[9] and health systems have been stretched to the limit, resulting in negative implications for the health of all populations, particularly when access for patients with other acute and chronic illness is limited.[8] As of 01/09/21, UK (population 66.8 million)[10] has 6,821,356 confirmed cases and 132,859 COVID-19 related deaths.[11] In the UK, the National Health Service delivers care for most of the population. Meanwhile during the same time period, Liberia (population 4.9 million)[10] has had 5594 confirmed cases, with 245 confirmed COVID-19 related deaths.[11] There are marked differences

For peer review only - http://bmjopen.bmj.com/site/about/guidelines.xhtml

between settings in the roll-out and scope of testing capacity and uptake of this, with under-reporting in many lower middle income countries, and so these figures cannot be assumed to be accurate. Future comparisons will eventually show the magnitude of all-cause mortality by age, and firm conclusions can be made about the success of different country approaches. Liberia was, initially hailed as one of the top countries in fighting COVID-19, being one of the first countries to start screening at ports of entry (January 2020) and to adopt other control measures such as rapid testing, contact tracing and quarantine.[12,13]

"Improving resilience within health systems can build on pre-existing strengths to enhance the readiness of health system actors to respond to crises, while also maintaining core functions."(page 1 [1]). People-centred health systems are a critical framing in shaping resilience as they place people and communities at the centre whilst also promoting strategic and collaborative multi-sectoral leadership which is necessary in delivering a co-ordinated response to a public health crisis.[14] In this paper, we compare health systems responses at a single point in time (June to September 2020) within Monrovia, Liberia and Merseyside, UK to distil lessons for health systems resilience to a pandemic through comparative case studies which explore aspects of health systems resilience.[15] Within this paper we combine the Foreign, Commonwealth and Development Office (FCDO) eight key principles for promoting resilient health systems with key domains and values of people-centred health systems to frame our findings in relation to the COVID-19 response.[16] Through our discussion we reflect on these principles against our conceptual framework (figure 1), which is based on a people-centred approach. In response to calls for on-the-ground analysis of the response to COVID-19 within the Global South and comparative case studies that use co-creation and coproduction approaches which go beyond researchers including policy makers, practitioners and the public,[15,17] we seek to share learning from the response within Liberia and the UK, along with opportunities for multi-directional knowledge sharing.[17] It is our hope that this paper will help inform health policy makers across global contexts, for the current pandemic response and as they plan towards more resilient people-centred health systems to meet future shocks.

For peer review only - http://bmjopen.bmj.com/site/about/guidelines.xhtml

Methods

Study context

Liberia and UK have had very different strategies and case rates from the outset of the pandemic, although there were some similarities in the adoption of infection prevention control measures across both contexts. Liberia is amongst the world's poorest in terms of GDP and living conditions. According to the World Bank 2016 poverty headcount ratio, 44.4% of Liberians live below the international poverty benchmark of $1.90 USD per day.[18] The UNDP Human Development Report 2020 ranks Liberia low at 175 out of 189 countries and territories.[19] Inequities between females and males are remarkable with literacy rates (secondary education) of 18.5% and 40.1% respectively.[19] Liberia has prior experiences of shocks in the form of two civil wars, and the 2014-2015 Ebola Virus Disease (EVD) epidemic.[20] In response to these experiences, Liberia has prioritised rebuilding a resilient health system, which acknowledges the critical role communities play in addressing their own health needs through the 'Investment Plan for Building a Resilient Health System in Liberia' and the community health services policy (2016-2021).[21,22] By contrast, Merseyside is a Metropolitan County in the North West of England, comprising five boroughs, including the City of Liverpool, including some of the most deprived council areas in England.[23] It has a population of 1.42 million and has had some of the highest numbers of COVID-19 cases in the UK.[24] Within Merseyside, the Liverpool City Region Combined Authority has prioritised tackling deprivation and reducing health inequalities through people-centred care, with integration of health and social care services.[25] Liverpool has a long history of public health innovation, but also a strong sense of local history, culture and place. Throughout the pandemic Liverpool has been at the forefront of community-based innovations and public health strategies, e.g. piloting community open access testing for COVID-19.[26]

Liberia introduced stringent border control measures from January 2020, with the establishment of a Special Presidential Advisory Committee on Coronavirus (SPACOC) over two months prior to the first recorded cases in the country.[27],[28] Liberia's response to COVID-19, prioritised a call to maintain

For peer review only - http://bmjopen.bmj.com/site/about/guidelines.xhtml

the delivery of routine health services at all levels. Hospitals and clinics continued to provide health services with health facility workers trained in infection prevention control (IPC) before the first case was identified in country.[28] Physical distancing measures were introduced and use of face masks encouraged.[29]

Within the UK, health service delivery was restructured as part of the COVID-19 response, with routine non-urgent elective care suspended and later re-started in April 2020.[30] Adaptations to minimise potential risk of COVID-19 infection include the use of telemedicine and phone consultations; and changes to essential services for patients, such as changed treatment plans and delays to surgeries.[31] Hospital patient pathways were altered to appropriately triage and cohort the care of COVID-19 patients, reducing the risk of transmission to others and allowing essential services to continue. There was also reduction in routine blood test screening to prioritize COVID-19 PCR testing in response to the UKs 'test and trace' strategy.

Study aim, design and conceptual framework

Aim: To understand COVID-19 adaptations and decision-making in Liberia and Merseyside, UK

This qualitative study explored inductively the differing experiences, perspectives and recommendations of participants in order to understand COVID-19 adaptations and decision-making in Liberia and Merseyside, UK.[32,33] We selected qualitative methods to give “due emphasis to the meanings, experiences, and views of all the participants”(page 43 [32]) and understand decision-making and the impact of health systems adaptations as a result of COVID-19.

A conceptual framework was jointly developed, following a series of meetings held with researchers in each setting (7 Liberia-based researchers and 18 UK-based researchers). This framework sought to consider a people-centred approach towards the health system’s ability to respond to shock, whilst reflecting the realities experienced in the face of multiple routine challenges (Figure 1).[34] The nature of a shock to the health system, whether due to infectious disease outbreak, natural disaster,

For peer review only - http://bmjopen.bmj.com/site/about/guidelines.xhtml

or conflict, influences the rest of the framework.[35] It adopts a people-centred approach at its heart,[14,36,37] while incorporating literature relating to the health system's ability to respond to a sudden shock, and the extent to which it is able to absorb, adapt and transform in response (Figure 1).[35,38–42]

People-centred health systems prioritise the collective right to health through integrated and targeted approaches that favour the needs of the most vulnerable.[14,43] Collective action and social solidarity are viewed as essential to the art and science of the development of people centred systems that are organised around people's health care needs and expectations as opposed to diseases, ensuring a continuum of care throughout the life course.[14] This approach embraces the human character of health systems, by viewing individuals, communities and health workers as co-producers of health care, placing people and families at the centre.[44] Systems must adapt to meet a range of challenges to support the development of strategies that seek to improve health care access and encourage universal coverage. This is particularly important as many individuals transition and oscillate between multiple roles of patient, family and sometimes health care provider within one system.

Interview topic guides were informed by the framework and developed across both settings to explore key areas of health systems functioning in response to COVID-19 (Appendix 1). Questions included: governance and decision-making; use of ethical guidelines; human resource management, infrastructure (information technology and communications) and health care worker support; introduction of innovations; and perceptions of the equity and quality of service delivery. Adaptations were made according to the health systems context in each country, for example in Liberia, additional questions were included to explore how learning from the EVD epidemic and other health systems shocks informed COVID-19 response planning.

Figure 1 placed here

<u>Study participants and data collection</u>

For peer review only - http://bmjopen.bmj.com/site/about/guidelines.xhtml

The study was carried out at different levels of the health system across both settings (Table 1). In Liberia, we conducted key informant interviews in June and July 2020 with 21 national level and three county level decision-makers (Nimba, Margibi and Montserrado Counties) purposively selected because of their involvement with COVID-19 planning and/or routine service delivery. Some had also played key roles in the EVD epidemic response. In Merseyside we conducted 42 key informant interviews between July to September 2020, with regional, hospital and primary care decision-makers (general practitioners and residential care home manager) and front-line workers selected because of their involvement with COVID-19 planning and/ or the delivery of COVID-19 or routine services (see Table 1). More interviews were carried out within the UK across health systems levels, due to demand for research across multiple levels and the presence of a larger team of researchers. In Liberia, by contrast the demand for research was focused at national level, and the research team was smaller in size. The national and county level actors in Liberia, spoke about Liberia's response as a country. In contrast study participants in Merseyside from across health systems levels, including frontline health workers, spoke of their own direct experience within a particular hospital or setting, or on behalf of Merseyside City Region. We acknowledge the limitation that including national and county level actors only within Liberia, creates a somewhat limited perspective. It would have been preferable to have included a larger number and range of participants from sub-national health systems levels to provide more depth of understanding about the COVID-19 response.

Table 1 Study participants' role

**Participant Role**	**Number of Participants Interviewed**
**Merseyside, UK**	
Regional decision-maker	5
Hospital decision-maker (Clinical director, medical director, ward manager)	4
Hospital consultant	11
Hospital health worker (junior doctors, nurses)	10
Health worker in community (GP, district nurse, residential care home)	7
Liverpool Clinical Laboratory staff	5
**Total**	**42**

For peer review only - http://bmjopen.bmj.com/site/about/guidelines.xhtml

Liberia participants	
National decision-maker	21
County decision-maker	3
**Total**	**24**

Interviews were predominantly carried out remotely by researchers experienced in qualitative interviewing in English language, via online platforms such as Microsoft Teams or Skype. A minority were carried out in person with physical distancing measures in place, according to local guidance at the time. All interviews were audio-recorded. Data collection stopped when no new themes emerged from additional data collected.[45] Interviews lasted approximately 30 to 60 minutes. Audio recordings were transcribed verbatim, with quality assurance conducted by a second researcher against the recording.

Data Analysis

The study has sought to use a pragmatic approach to research, working through existing networks to carry out timely research to support the ongoing COVID-19 response in both settings. Both inductive and deductive approaches were blended within data analysis, in keeping with other health systems research [46–49]. In both Liberia and UK, preliminary data analysis workshops were held separately with the research team members involved with data collection. Prior to the workshops all participants reviewed transcripts to familiarise and immerse themselves within the data in order to inductively identify emerging themes which arose from within the study findings. Through these separate country workshops key themes were identified and used to generate a separate coding framework for each setting. All transcripts were imported into NVivo Version 12 qualitative data analysis software for coding (QSR International Pty Ltd. Version 12, 2018). Following review of the initial themes which emerged inductively from within the data, there was found to be strong alignment with the eight FCDO principles. These principles were then deductively applied to assist with mapping the findings and enabling comparison between settings. The research team did not simply accept the eight FCDO principles, rather the team reviewed them and found that they did not fully cover all the aspects of

For peer review only - http://bmjopen.bmj.com/site/about/guidelines.xhtml

resilience which emerged from the data. As a result two further principles were identified, relating to "mechanisms for advance preparation" (Principle 9) and "adaptable governance and leadership structures" (Principle 10). The two new principles were applied to adequately compare findings between both settings. The application of the expanded FCDO principles has helped to showcase how Liberia's experience with responding to prior shocks and their learned need for early advance preparedness provided an important element working towards resilience. This study is not funded by FCDO, nor were FCDO involved in any way as researchers or co-authors within the research team.

Detailed findings and recommendations were developed into two policy briefs in accordance with these principles and were shared and discussed with relevant stakeholders from both study settings.[29,50] The relationship of the findings to the original conceptual framework was reviewed and findings compared between settings during a final on-line workshop, attended by all those involved with data collection in both settings, with key similarities and differences jointly discussed.

Ethics

Ethical approval was received from the Liverpool School of Tropical Medicine Research Ethics Committee (Protocol ID 20-045); the University of Liverpool Ethics Committee (Reference 7811) and the University of Liberia-Pacific Institute for Research and Evaluation Institutional Review Board; National Health Service Health Research Authority and Health and Care Research, Research Ethics Committee (Reference 20/HRA/2597); Integrated Research Application System (Project ID 284143). All study participants were provided with a participation information leaflet at least 48 hours prior to interview. All participants provided written, or audio recorded consent to participate.

Patient and public involvement

Neither patients nor the general public were involved in the design, conduct, reporting or dissemination of our research.

Results

For peer review only - http://bmjopen.bmj.com/site/about/guidelines.xhtml

We present findings according to the FCDO principles (Box 1) (key illustrative quotes are summarised for each principle in table 2). We then reflect on the findings in light of people-centred health systems within the discussion.

Box 1 Ten Principles of Health Systems Resilience in the Context of COVID-19 Response

Principle 1 Develop flexible pathways for medical supplies
Principle 2 Prioritise a list of essential health services [*and continued provision of quality and equitable routine services*]
Principle 3 Build trust with local communities
Principle 4 Foster good communication at all system levels
Principle 5 Support, recognise and encourage staff
Principle 6 Facilitate rapid resource flow and greater flexibility in its use
Principle 7 Ensure agile tracking of health information
Principle 8 Cultivate effective partnerships and networks
Principle 9 Structures and mechanisms for advanced preparedness (**New principle**)
Principle 10 Adapt governance and leadership structures to facilitate timely decision-making and effective coordination of response **(New principle)**

Table 2 Illustrative quotations from Liberia and Merseyside related to each FCDO Principle

Principle	Comparison	Quotations
**Principle 1: Develop flexible pathways for medical supplies**	Supply chains disturbed across settings due to global shortages and price inflation.<br>Lack of buffer stock in both settings.<br>Restructuring of supply chains in Liberia led to disturbance for routine supplies.	*"Supply chain are affected greatly because their concentration is on how to provide the COVID response activities meaning the …medicines and medical supplies that are needed [for] NTDs (Neglected Tropical Diseases), lack of attention will now be paid to that." (LIB national decision maker 029)*<br>*"With regards to PPE, there was national guidance about what we should do and there was a huge amount of fear amongst nurses and medics and everyone else understandably. Everyone was scared. I was scared. If someone said they weren't scared, then they're lying or they're a fool. The national guidance was confused, and availability of PPE fluctuated. Procurement here [NHS hospital] did a very good job, but sometimes it just wasn't delivered nationally. And we went through other supply chains…" (LIV hospital decision maker, Merseyside UK 014)*
**Principle 2: Prioritise a list of essential health services [and continued provision of quality and equitable routine services]**	Discontinuation of elective non-urgent care in UK, contrasts with early emphasis on continued routine care in Liberia.	*"So we just have to be robust and do the necessary investment into routine health services, preventive in terms of creating awareness and education among health workers about covid and how we can continue to care for our patients, with fighting the infection at the same time." (LIB national decision maker 001)*<br>*"There's the whole big risk around the screening program…the screening program was stopped, restarting that it's gonna be really*

For peer review only - http://bmjopen.bmj.com/site/about/guidelines.xhtml

Principle	Comparison	Quotations
		challenging. And I suppose that's another risk in terms of people with delayed diagnosis and the right treatment, as a result of not having had that screening mammograms." (LIV hospital decision maker Merseyside UK 051)
**Principle 3: Build trust with local communities**	Both settings experiences reduced service utilisation due to loss in community trust. Introduction of innovative follow-up visits to patients led to increased service use in Liberia.	*"Some of the useful things that we have been using from Ebola time is, as I said before, to involve the communities …The community aspect is very important because it will help us for the COVID-19 where communities, family members, all of those at the community level are influential group they will be able to comply like we did in the Ebola." (LIB national decision maker 005)* *"The elderly population have been shielding because of comorbidities and all that. I think they probably not being as vocal about things that they're concerned about because they're worried about that they will be asked to come in. They fear that that they will catch Covid when they come here." (LIV hospital health worker Merseyside UK 048)*
**Principle 4: Foster good communication at all system levels**	Expansion of virtual communication in both settings. In Merseyside frequently changing guidance from multiple sources created confusion.	*"One of the things that quickly used to come to me is to able to adapt to working with social media technology and all of that, because that's the first thing if you have to communicate with people in this manner you need to understand zooming, skyping, how to take notes.." (LIB national decision maker 029)* *"And there's so many different sources of information that say different things from what people hear within the hospital talking to friends on the corridor, that you've got to come out with a consistent message. And I think it took longer than was ideal to get a central source of information…But people need to be told what the situation is rather than try to be falsely reassured sometimes as well." (LIV hospital decision maker, Merseyside UK 004)*
**Principle 5: Support, recognise and encourage staff**	Health worker redeployment was common across settings. Health worker training varied in UK according to cadre.	*"Like take for example, when COVID came some of our workers from the [name] Hospital was recruited to go at the front line and [hospital name] is for routine services so taking employees from there to go at the front line that tells you it kind of understaff… So routine services kind of slow down and every attention was placed on COVID but going forward, with the system in place, routine services have gotten back on its feet." (LIB national decision maker 010)* *"And it felt like there was unequal share of knowledge and also an unequal kind of confidence in protective clothing. … And I think the people that spent the most time with the patient, the patient areas, for instance, the health care assistants and the cleaning staff didn't have all of the information*

For peer review only - http://bmjopen.bmj.com/site/about/guidelines.xhtml

Principle	Comparison	Quotations
		[at the] beginning or any PPE training." (LIV hospital health worker Merseyside UK 017)
**Principle 6: Facilitate rapid resource flow and greater flexibility in it's use**	Prior under-investment in health was common across settings.<br>In Merseyside there was increased funding available and removal of bottlenecks, which enabled swifter action.	*"The first thing is, we need ownership by government, ownership is not depending on other countries to provide us the resources, to provide the technical capacity. So that is the best recommendation I would say. The ownership has to be there, resources have to be available and the infrastructure has to be available in terms of being resilient." (LIB national decision maker 029)*<br>*"To be honest, it was a fairly novel experience because it was a situation where if we asked we more or less got [funding]." (LIV hospital decision maker, Merseyside UK 004)*
**Principle 7: Ensure agile tracking of health information**	Data quality reduced in Liberia.<br>In Merseyside increased data was collected, but inadequate data analysis measures were put in place.	*"Another recommendation is that we could include COVID-19 to our regular disease surveillance. Like we have the measles, the Lassa, and thing. I think we should include COVID because COVID maybe all around. Like we included Ebola, there should be a document on COVID-19 that will form part of our regular surveillance." (LIB county decision maker 024)*<br>*""…there's some value in looking at the things that we were looking at before COVID, because at least we have some longitudinal data on that so that we can see what the effect of COVID is." (LIV hospital health worker, Merseyside UK 020)*
**Principle 8: Cultivate effective partnerships and networks**	Liberia was able to call upon prior decision-making structures (established during Ebola response) to enable swift decisions.<br>Need for stronger engagement between primary and secondary care in Merseyside.	*"Involvement of multi-sectorial stakeholders in the response; that was one major thing that we learned from Ebola. And that has been brought to be on this response, so there has been a spark from the level of the presidency where they have key ministries and agency heads heading pillars on the COVID-19 response, involving the community people." (LIB national decision maker 028)*<br>*"I think one thing, it's really highlighted is the divide between hospital and primary care. We didn't work together very well before the epidemic, and we are still not working together very well. And I think if things were to get better, the whole health system needs to work better." (LIV community-level health worker, Merseyside UK 033)*
**Principle 9: Structures and mechanisms for advanced preparedness**	Learning from Ebola prompted rapid preparedness in Liberia, in contrast to Merseyside.	*"If you don't prepare well and you are caught unaware you will have a lot of issues, so we didn't wait for COVID to enter Liberia before we prepositioned basic PPE and those are all part of the preparedness phase." (LIB county decision maker 026)*<br>*""It was blatantly obvious that anything we've ever planned for in relation to a pandemic or anything along those lines was not the plans that we needed… So I think going forward there needs to be almost a better planning system in place…it's not just a matter of just saying any pandemic it's about*

For peer review only - http://bmjopen.bmj.com/site/about/guidelines.xhtml

Principle	Comparison	Quotations
		what kind of pandemic." (LIV hospital decision maker, Merseyside UK 069)
**Principle 10: Adapt governance and leadership structures to facilitate timely decision making and effective coordination of response**	Need for rapid guidance from national level to enable sub-national decision making was common in both settings.	*"So, at this point in time we think if you give the resources, put the money in the hands of the county health team to buy what they need, that will be more effective … So, we want decision should be given back to the people on the frontline so that they make the decision rather than a centralized point in Monrovia where people sit and decide for people in the lower level and the people choices made the right kind of thing they might need at that level." (LIB national decision maker 028)*<br>*"… we were having to work, to a large extent, in the dark. The amount of guidance that came through nationally and even regionally, was actually relatively limited at that stage and we were having to do what felt like quite a lot of planning in isolation." (LIV decision maker Merseyside UK 008)*

<u>Principle 1 Develop flexible pathways for medical supplies</u>: Across both settings supply chains were disturbed due to global shortages and price inflation. In Merseyside there was a lack of personal protective equipment (PPE) and laboratory reagents needed for COVID-19 testing. Meanwhile, in Liberia, the disturbances related to routine supplies as supply chains shifted to focus on COVID-19 related procurement. In both settings, these challenges were felt to relate to global shortages, but were worsened by failure to maintain buffer stocks at local and national levels. In both settings, participants expressed the need for greater decentralisation of procurement decisions.

<u>Principle 2 Prioritise a list of essential health services</u> [*and continued provision of quality and equitable routine services*]: Participants from Merseyside expressed fears that there was too much emphasis on COVID-19 care, at times creating redundant capacity, while limiting access and quality of routine essential services. The blanket discontinuation of all elective non-urgent care at the height of the first wave in Merseyside, UK was felt to be unhelpful, and a more nuanced approach which seeks to balance long-term as well as short term risks associated with health conditions was recommended. In contrast, Liberia's early emphasis on routine health services was described as a key learning prioritised by decision-making platforms following the country's experience with the EVD epidemic.

For peer review only - http://bmjopen.bmj.com/site/about/guidelines.xhtml

287 COVID-19 adaptations in the UK led to increased telemedicine, with some respondents raising access-related equity concerns, particularly for elderly populations, who may struggle to engage with telemedicine. There were also concerns raised about quality of care, with some participants in Merseyside fearing delayed-diagnosis, misdiagnosis or sub-optimal care due to restrictions limiting physical contact with patients. In Liberia, limited opportunities for supervision, diversion of funds and staff for routine services towards COVID-19 response, and limited community outreach activities (due to physical distancing) were felt to impact quality of care. Across both settings innovations in service delivery have emerged (see policy briefs for details).[29,50]

295 Principle 3 Build trust with local communities: In both settings, community trust to seek health services declined, which reduced utilisation of services. In Liberia, fear among the population during the start of the pandemic led to reduction in the uptake of health services including national routine vaccination programmes and health facility-based delivery. This was felt to relate to a combination of fear of contracting COVID-19 at facilities and to reduced community outreach activities. Innovative community engagement and social mobilization strategies were introduced, for example follow-up visits to pregnant women, which led to patients returning to use services after a few months. Another example is the selective outreach home visits by the Neglected Tropical Disease (NTD) programme to NTD affected patients, in order to avoid interruption in treatment provision. In Merseyside, utilisation of non-COVID related services remained supressed for much longer. This was deemed to relate to widespread community mistrust, and Government campaigns which initially discouraged the public from visiting health facilities via the national 'Stay at home' messaging. Applying learning from Liberia's experience with EVD, the Government of Liberia placed a strong emphasis on working alongside community governance structures, involving local authorities as part of COVID-19 response.

309 Principle 4 Foster good communication at all system levels: The need for effective communication within the health system appeared to be a significant theme, particularly within findings from Merseyside. The rapidly changing context during the early months of the pandemic created a wealth

For peer review only - http://bmjopen.bmj.com/site/about/guidelines.xhtml

312 of daily new information. Virtual forms of communication rapidly expanded in both settings, with
313 WhatsApp and online meeting platforms used extensively. Within Merseyside, referred to challenges
314 such as multiple sources of guidance and communication channels struggling to keep pace with the
315 changing guidance, which at times created contradictory messaging and confusion among health
316 workers. By contrast, Liberia developed a centralised messaging procedure with approval needed
317 from the department of Health Promotion before dissemination. In Merseyside, use of emails were
318 typically less popular with staff as these could often be too long and wordy. Participants expressed
319 limited scope for frontline staff to feedback on the information that had been shared.

320 <u>Principle 5 Support, recognise and encourage staff</u>: Staff redeployment was common across both
321 settings, contributing to varied workloads. In Liberia, health worker redeployment to COVID-19
322 treatment centres, alongside largely unchanged utilisation rates contributed to increased workload
323 for remaining health workers responsible for provision of routine services. By contrast in Merseyside,
324 redeployment resulted in over-staffing in certain COVID-19 wards. Although there was disparity
325 between health workers, with nurses experiencing increased workload. Due to the reduced volume
326 of patients seeking routine care in the UK, workload was variable for those providing these services.
327 The degree to which health workers received training about COVID-19 prior to having to manage
328 COVID-19 patients varied between settings, with Liberia carrying out training in identification,
329 isolation and infection, prevention and control before the first case of COVID-19 arrived in country, as
330 a result of lessons learned following experiences responding to EVD. By contrast in Merseyside, the
331 roll out of training varied widely by cadre, with some participants identifying that health care
332 assistants and cleaning staff did not receive PPE training until later in the pandemic, compared with
333 doctors and nurses (see table 2).

334 Anticipated mental health implications for health workers emerged from the Merseyside data, due to
335 high rates of COVID-19 infection, exhaustion and high future anticipated post-traumatic stress
336 disorder (PTSD). This was associated with fear of making treatment mistakes, stress surrounding

For peer review only

patient escalation decision making, anxiety over potential COVID-19 infection (both personal and for family), trauma surrounding high COVID-19 infections and deaths and reduced psychosocial support due to remote working. Measures to support staff wellbeing were introduced (including counselling, reflective therapy, peer support and mentoring, information made available about local support services), with varied levels of uptake. This was not widely discussed in Liberia. Although measures in Liberia to support staff wellbeing include psychosocial teams, roaming mental health counsellors providing services to health workers are in place. In Merseyside, community support, strong solidarity and teamwork were considered enablers of staff resilience.

Principle 6 Facilitate rapid resource flow and greater flexibility in its use: Historic underfunding of the health system in both settings has been highlighted by the pandemic. In Merseyside, this was considered to be due to nearly a decade of austerity, which has created weariness and uncertainty; whereas in Liberia it related to perception of reliance on external donors which predated the pandemic. Our findings confirmed the need for adequate funding to ensure the building blocks of the health system have received investment prior to the onset of any shock. With the arrival of the pandemic the availability and flexibility of funding differed between settings. In Merseyside, UK there was increased central government funding, which was mostly freed of usual bureaucratic checks. Managers noted that the removal of these bottlenecks allowed for swift action and rapid adoption of innovations. Frontline managers' ability to make operational decisions was viewed as central to resilience. In Liberia, however, there was an identified need for greater Government of Liberia ownership. Some sectors of the health system, particularly those which are donor reliant struggled in response to reduced partner support following the pandemic. Initially funding was not made available, however funds for routine service delivery were re-allocated to COVID-19 response, with implications for quality (see principle 2). Participants complained about excessive bureaucracy associated with use of funds, which created delays.

For peer review only - http://bmjopen.bmj.com/site/about/guidelines.xhtml

Principle 7 Ensure agile tracking of health information: Health information systems (HIS) were rapidly developed in the UK to collect huge quantities of surveillance data on COVID-19 and essential services. However, there was need for improved skills to usefully interpret this data. Respondents in Liberia stated that regular and timely submission of data, particularly from the community level had declined since the onset of COVID-19. This was considered to relate to reduced data validation, with decreased supervision visits due to physical distancing. In Merseyside complex new systems were designed to collect pandemic surveillance data, however, data was frequently not analysed or made readily accessible to staff to influence timely monitoring and quality improvement in services. In Merseyside, respondents also noted that a number of new initiatives were introduced during the pandemic, such as virtual consultations, but have not yet been systematically evaluated.

Principle 8 Cultivate effective partnerships and networks: The need for well-established partnerships emerged in both settings, with Liberia already having clear multi-sectoral participation in decision-making following the Incident Management System developed following EVD. Merseyside data highlighted pre-existing weaknesses in collaboration between primary and secondary/ tertiary care have been exacerbated. In both settings the need for greater engagement with the private sector was affirmed, with respondents from UK highlighting the need for stronger links regarding PPE supply chain shortages and in Liberia the need to strengthen collaboration given perceived weakness in private facility IPC standards. Partnerships were established within Merseyside, in a range of aspects of service delivery, including: regional network of laboratory providers to address equipment challenges and ensure COVID testing; between GPs to create service hubs; between disciplines and departments within hospital to address staff shortages and share information. In Liberia, a reduction in the number of partners providing response support was noted. This was a marked contrast to the EVD response.

Principle 9 Structures and mechanisms for advanced preparedness (newly identified principle from our findings): Within Liberia in particular, but also in Merseyside, there was discussion about

For peer review only - http://bmjopen.bmj.com/site/about/guidelines.xhtml

advanced preparedness. Respondents in Liberia emphasised how their experiences with previous shocks, particularly EVD, had facilitated learning around early recognition of the need for preparedness. For instance, there was consensus among respondents that waiting for COVID-19 to reach Liberia before responding would be too late. There was early rapid mobilisation of existing emergency response systems which had been established during the EVD response including; health check controls and quarantines at border points from January 2020; health worker COVID-19 training before the first confirmed case; enhanced hygiene practices; restriction of physical contact and sustained use of PPE, building on institutional memory gained through the EVD epidemic. In contrast, respondents in Merseyside expressed that the COVID-19 response was impeded by a lack of pandemic preparedness for new emerging infectious diseases.

Principle 10 Adapt governance and leadership structures to facilitate timely decision-making and effective coordination of response (newly identified principle from our findings): Being able to adapt governance and leadership structures to facilitate timely response coordination emerged from both settings. Liberia had previously established the incident management system (IMS) in 2014 as part of the response to EVD. It was re-activated in March 2020 to guide planning their pandemic response, led by the Minister of Health. This multi-sectoral team included a range of political and public health decision-makers, donors and partner representatives. At the time the study was carried out, most decisions were made centrally, with implementation at county level. In Merseyside, early response was hindered by slow and centralised guidance and decision-making, which was perceived to be oriented towards achieving political goals, rather than providing much needed clarity and recognition of local reality. The limited scope for local autonomy was considered to strain relationships between local senior leadership who sought to enforce central directives, and frontline staff, who wanted scope to influence them. In both settings, there was interest in greater de-centralisation of decision-making to lower levels.

Discussion

For peer review only - http://bmjopen.bmj.com/site/about/guidelines.xhtml

411 Our findings demonstrate the commonalities between the principles for resilience and people-centred
412 health systems (Figure 2). We believe that maintaining a people-centred approach can help ensure
413 that COVID-19 related adaptations are acceptable, understood and meet the needs of individuals
414 (both patients and health workers). The values which underpin people-centred health systems
415 emphasise the need for equity, orienting health services towards a health system which puts "people
416 and communities at their centre, and surrounds them with responsive services that are coordinated
417 both within and beyond the health sector, irrespectively of country setting and development
418 status."(page 9 [14])

419 **Adapting a people-centred framework**

420 All ten FCDO principles (eight original principles and two principles identified through this study) are
421 mapped against the original conceptual framework, to demonstrate the connection between our
422 findings and existing literature about resilience (Figure 2) and recommendations in response to each
423 principle are outlined in box 2.

424 Figure 2 placed here

For peer review only

For peer review only - http://bmjopen.bmj.com/site/about/guidelines.xhtml

Capacity and knowledge exchange

The continuation of routine essential service delivery following a shock to the health system, has previously been highlighted as an area of concern across a range of sectors.[51,52] Health systems

Box 2 Recommendations from our adaptation of FCDO principles

1. Supply chains should pre-position adequate stocks, diversify sources and seek decentralisation of procurement. Collaboration between providers can prove valuable in securing continuity of supplies.
2. Routine services should be prioritised with a view to long term as well as short term impact, with prioritisation re-evaluated regularly as the pandemic progresses.
3. Maintain consistent communication and engagement with community leaders as partners to participate in pandemic planning within their respective communities.
4. Keep communication channels open, with regular updates for staff which highlight the key information, preferably through meetings, rather than email.
5. Ensure adequate provision of training, with sufficient PPE for health staff particularly for those staff at highest risk of COVID-19 infection, alongside measures to balance workload and promote staff wellbeing. Prioritise compassionate leadership which is supportive of staffing levels and rotas, along with staff mental wellbeing. Investment in psychosocial wellbeing throughout and after the pandemic response.
6. Health systems need to be adequately funded during 'normal times' if they are to be able to respond when a shock arises. There is urgent need for investment to clear the backlog of delayed routine services.
7. Health information systems need greater investment in both the systems and the human element to be able to analyse, interpret and respond to emerging data trends.
8. Opportunities for multisectoral collaboration should be sought out, with engagement with private sector where possible.
9. Develop a proactive approach, with advance plans for health shocks, along with escalation and de-escalation plans throughout the crisis.
10. Promote greater opportunities for de-centralised staff involvement in decision-making where feasible. Governments to prioritise an outward focus towards global solidarity.

need the capacity to continue to deliver services of good quality alongside responding to wider health challenges.[42] Our findings for principle 2 highlighted that COVID-19 adaptations in the UK led to the cancelling or postponing of many essential services, including those related to cancer care, which has been anticipated to decrease life expectancy and survival.[52,53] Meanwhile, Liberia emphasised the need for continuation of routine services and the promotion of patient confidence to use these services. This is in contrast to the EVD epidemic, where over 80% reductions in maternal delivery care

in EVD affected areas were described and form part of the reason why routine care was prioritised so strongly as part of the COVID-19 response.[54]

Our findings relating to supply chain (principle 1) resonate with literature from previous shocks and research emerging from the COVID-19 pandemic.[55,56] We found the need for greater flexibility, with engagement with a more diverse range of suppliers and greater decentralised control over supply chain across both settings. This is in keeping with a recent systematic review of supply chain resilience literature, which identified the importance of diversity and the social aspects of supply chains during a pandemic response.[55] Supplying commodities without investing in health systems strengthening will not produce a robust supply chain, limiting ability to respond quickly and effectively to future demands.[55]

We found a strong focus on the need for support for the health workforce, particularly in UK (principle 5). This was not as widely discussed in Liberia (though this may be a limitation relating to differing levels of participants between countries). However, a previous study in Sierra Leone and Liberia, highlighted that many providers may carry unresolved trauma from earlier shocks (including the Ebola epidemic), which may have implications for them during the COVID-19 response.[57,58] Research among health workers treating patients with COVID-19 in China, revealed health workers had a higher prevalence of insomnia, anxiety, depression, somatisation and obsessive-compulsive symptoms compared with nonmedical health workers, indicating the need for support and recovery programs for these staff.[59] Stressors identified among workers in China, include many of those described by participants in both settings within our study, particularly within Merseyside, including difficulties feeling safe at work, lack of infection prevention and control (IPC) measures and COVID-19 knowledge, long term workload, high risk of exposure to COVID-19, shortage of PPE and lack of rest, among others.[59]

Our findings regarding resource flow to frontline providers (Principle 6), are in keeping with previous study which identified funding as a core dimension within a health systems' ability to adapt and

For peer review only - http://bmjopen.bmj.com/site/about/guidelines.xhtml

respond to shocks.[60] A recent systematic review found aggregate public spending for health is associated with improved life expectancy, reduced child and infant mortality and more equitable health outcomes.[56]

Relational and teamwork components

The relational components which exist are shaped by risk, trust, values, power, norms, and culture.[42] These components play a role in determining the success (or failure) in response to a health systems shock or crisis. In contrast to the FCDO recommendation for good communication between actors (principle 4), our findings highlight challenges, particularly in the UK, where communication channels struggled to keep pace with changing guidance creating contradictory messaging and confusion among health workers. This is in keeping with previous study which found differences in lines of authority and acceptability of communication pathways can contribute to problems in communication.[34] In response, key principles were identified including participation for all, respect, information sharing, collaboration and problem-solving.[34]

The need for strong governance structures and leadership which adapts to the response (principle 10), was identified as a gap within early response in Merseyside. This was felt to have been hindered by slow and centralised guidance and decision-making with a perceived limited scope for autonomy within decision-making at lower levels. Within Liberia learning from the EVD response, and establishing an incident management system (IMS) (led by the Minister of Health) and Special Presidential Advisory Committee on Coronavirus (SPACC) (led by the President) early in planning their pandemic response enabled timely decision-making.[27] In both settings, there was interest in greater de-centralisation of decision-making to lower levels. Blanchet et al (2017) emphasised the need for legitimacy within resilience, with requirement of capacity to develop socially and contextually accepted institutions and norms.[40]

Looking more broadly, the conceptual framework highlights community engagement, with the community being active participants of any health systems response (principle 3).[39] Our findings

For peer review only - http://bmjopen.bmj.com/site/about/guidelines.xhtml

485 emphasise the value of community engagement within the response within Liberia, based on lessons
486 from the EVD pandemic and in keeping with WHO recommendation that this be a key pillar within
487 COVID-19 country response.[8] Liberians across all socio-demographic groups responding to a recent
488 survey said they were very well, or somewhat well informed about the COVID-19 pandemic, with only
489 5% feeling not very well/ not at all informed.[27] This also emerged as a key finding in Singapore, with
490 engagement through new and social media channels monitored, with clarification of misinformation
491 by MOH.[61] In contrast to the findings from Liberia, participants from Merseyside highlighted the
492 need for stronger communication (although there were some examples of creative ways to engage
493 with diverse communities).

494 Learning from our study has emphasised the need to better prepare for, and respond to, health
495 emergency crises through integrated services (Principle 9).[44] A recent survey found most of the
496 population felt the Liberian government was doing well in managing the pandemic.[44] This
497 contrasted with findings from the UK where there was felt to have been a lack of adequate advance
498 planning and preparation. Two previous literature reviews highlighted that "preparedness depends
499 on health systems ability to learn from prior pandemics", with responses often reactive rather than
500 proactive.[56,62]

501 The people-centred approach stresses the need for awareness and recognition of the
502 interdependencies of the health system with the community and other social systems, including
503 education, social protection and food security and their relationship with social determinants of health
504 (principle 8).[63] Our findings emphasise the need for strong partnerships with other sectors across
505 settings, in keeping with an identified success in Singapore's response,[61] and is a key aspect of
506 Blanchet et al.'s resilience framework, ensuring the capacity to engage with and handle multiple actors
507 and dynamics.[40]

508 Our findings, particularly from Merseyside emphasise the vast quantities of data being generated
509 through the COVID-19 response, but there are gaps in how this data is analysed and utilised within the

For peer review only

For peer review only - http://bmjopen.bmj.com/site/about/guidelines.xhtml

health system. The importance of adequate HIS is in keeping with previous studies.[40,60] A health system's ability to identify and respond to an emerging threat is needed if it is to appropriately meet emerging needs during a rapidly evolving health crisis or shock (principle 7).[40,41] A robust health management information system (HMIS) is crucial to a health systems capacity to respond to shock.[60] Health systems need to have the ability to combine and integrate different forms of knowledge and to anticipate and cope with uncertainties and unplanned events.[40]

COVID-19 has reflected and exacerbated existing social inequalities and emphasised the importance of global collective action, rather than an individual response for genuine resilience. [8] Vaccine inequity and a lack of global solidarity on the part of some richer countries, are dominating the current phase of the pandemic. Our findings seek to highlight opportunity for shared learning across settings in the Global South and North, emphasising the need for a global response to this and future shocks.

Strengths and Limitations

The strengths of this study include the quality of data analysis, which involved a wide range of researchers across both settings, and the breadth of perspectives captured from frontline staff and key decision-makers early in the course of the pandemic. Our study had a number of limitations. Within Merseyside, study participants were selected from across a range of health system levels including primary care, hospital frontline workers and decision-makers as well as regional decision-makers. By contrast, in Liberia participants included national and county level decision-makers, technicians and supervisors of frontline staff, with no direct frontline workers included. This may result in some of the differences in findings, related to these differing perspectives. Perhaps the greatest limitation of this study is that it was carried out at a single point in time. In Merseyside we collected data towards the end of the first wave, at a time when there were few inpatients and people were reflecting on the first wave. Meanwhile in Liberia it was carried out before there had been a large increase in cases. Since the study was carried out there have been subsequent even greater waves of cases within Merseyside, UK and Liberia has experienced a large surge in cases of the delta

For peer review only - http://bmjopen.bmj.com/site/about/guidelines.xhtml

variant (59% of cases recorded in Liberia up until 17th July 2021, occurred during a six week period from June 1 2021 to 17th July 2021).[64] By the weeks beginning July 24th to August 7th 2021 number of confirmed cases had declined between zero to 43. Response measures have evolved in both settings, and limitations identified through the study may have been addressed in subsequent stages of the pandemic.

Conclusion

We found the ability of health systems to be able to absorb, adapt and transform in response to the COVID-19 pandemic in two very different settings closely relates to the eight FCDO principles of resilience.[16,40] We expanded these principles to include strong structures and mechanisms for advance preparation, and adaptable governance and leadership structures to facilitate timely decision-making and response coordination. At the heart of our findings lies the centrality of the people-centred health system, where the person, is placed within their family, community and the health system.[14] When all aspects work together the outcome is the extent of resilience demonstrated within a health system in response to shock.[40] This includes both the provision of specific services in response to the shock experienced, as well as continued provision of and demand for 'routine care'. Our study highlights the need to maintain a people-centred approach for a resilient health system response.

For peer review only

Acknowledgement

We would like to thank all the participants who made time to share their experience and reflections to make this research possible. We recognise and thank Abiola Aiyenigba, who sadly passed away during the study, for her inputs. This research was funded by the NIHR Health Protection Research Unit in Emerging and Zoonotic Infections, and the Centre of Excellence in Infectious Diseases Research, and the Alder Hey Charity. We also acknowledge support of Liverpool Health Partners and the Liverpool-Malawi-Covid-19 Consortium. Additional funding to support this work came from the NIHR REDRESS Programme (NIHR2001129) and FCDO COUNTDOWN (PO6407) programme. We thank Tim

For peer review only - http://bmjopen.bmj.com/site/about/guidelines.xhtml

Martineau, and Joanna Raven (LSTM) for early inputs into study design and Susie Crossman for managing the study budget.

Competing Interests

None declared.

Author Statement

RM prepared the first draft of the paper with inputs from all; Study design, conceptualisation, ethics (ST, LD, MT, LF, IB, ZZ, RM, VW, HP, RAdC, RH, KK); conducted interviews in UK – RM, VW, MT, KO, HP, SC, ST, TEH, RH, RD, YD, OH; conducted interviews in Liberia - ZZ, WT, HB, JK, JSS, CP, GZ, RM. All interviewers participated in the cross-country analysis which was led by YA in the UK with inputs from those who conducted UK interviews and LD, RM, ZZ, HB, WT, JK, JSS, GZ, CP in Liberia. All authors were involved in critical review of the approach, inputted into and approved the final draft of the manuscript.

Figure 1 caption: Conceptual framework

Figure 2: Principles for resilience and people-centred health systems framework

For peer review only - http://bmjopen.bmj.com/site/about/guidelines.xhtml

References

1. Meyer D, Bishai D, Ravi SJ, et al. A checklist to improve health system resilience to infectious disease outbreaks and natural hazards. *BMJ Glob Heal*. 2020;5(8):1–7.

2. World Health Organization. Operational guidance for maintaining essential health services during an outbreak [Internet]. 2020. Available from: https://apps.who.int/iris/bitstream/handle/10665/331561/WHO-2019-nCoV-essential_health_services-2020.1-eng.pdf?sequence=1&isAllowed=y

3. United Nations Economic Commission for Africa. COVID-19 in Africa: Protecting Lives and Economies [Internet]. 2020. Available from: https://repository.uneca.org/bitstream/handle/10855/43756/b11983206.pdf?sequence=1&isAllowed=y

4. Agyeman AA, Laar A, Ofori-Asenso R. Will COVID-19 be a litmus test for post-Ebola sub-Saharan Africa? *J Med Virol*. 2020;92(9):1373–5.

5. Zar HJ, Dawa J, Bueno G, et al. Challenges of COVID-19 in children in low- and middle- income countries. *Paediatr Respir Rev Rev*. 2020;(January).

6. Burki T. The indirect impact of COVID-19 on women. *Lancet Infect Dis* [Internet]. 2020;20(8):904–5. Available from: http://dx.doi.org/10.1016/S1473-3099(20)30568-5

7. Bayani DB, Krubiner C, Barasa E, et al. The Indirect Health Effects of COVID-19: Emerging Findings from Kenya, the Philippines, South Africa, and Uganda. *Cent Glob Dev*. (July 2021).

8. D'Ambruoso L, Abbott P, Binagwaho A. Building back fairer in public health policy requires collective action with and for the most vulnerable in society. *BMJ Glob Heal*. 2021;6(3):8–11.

9. Dzinamarira T, Dzobo M, Chitungo I. COVID-19: A perspective on Africa's capacity and response. *J Med Virol*. 2020;92(11):2465–72.

For peer review only

For peer review only - http://bmjopen.bmj.com/site/about/guidelines.xhtml

10. Das Gupta M, Mansuri G, Sinha N, et al. Overcoming gender-based constraints to utilization of maternal and child health services in Pakistan: the role of the doorstep delivery system. TT - [Internet]. Washington D.C.: [Unpublished] 2007. Presented at the 2007 Annual Meeting of the Population Association of America, New York, New York, March 29-31, 2007.; 2007. Available from: http://paa2007.princeton.edu/download.aspx?submissionId=71659

11. JHU. John Hopkins Coronavirus Resource Center [Internet]. Johns Hopkins University & Medicine. 2021 [cited 2021 Jun 7]. Available from: https://coronavirus.jhu.edu/

12. FPA Reporter. Former Center for Disease Control Boss Hails Liberia for COVID-19 Prevention [Internet]. Front Page Africa. [cited 2021 Jun 7]. Available from: https://frontpageafricaonline.com/news/former-center-for-disease-control-boss-hails-liberia-for-covid-19-prevention/

13. Frieden T. Which Countries Have Responded Best to Covid-19? Wall Street Journal [Internet]. 2021 Jan 1; Available from: https://www.wsj.com/articles/which-countries-have-responded-best-to-covid-19-11609516800

14. World Health Organisation. WHO Global Strategy on people-centred and integrated health services, Interim Report [Internet]. Geneva: World Health Organisation; 2015. Available from: https://apps.who.int/iris/bitstream/handle/10665/155002/WHO_HIS_SDS_2015.6_eng.pdf;jsessionid=B2F4B22FC042ACD2BEF20C5F34803E56?sequence=1

15. Saulnier DD, Blanchet K, Canila C, et al. A health systems resilience research agenda: moving from concept to practice. *BMJ Glob Heal*. 2021;6(8):e006779.

16. Department for International Development [DFID]. Principles of Health Systems Resilience in the Context of Covid-19 Response [Internet]. 2020. Available from: https://assets.publishing.service.gov.uk/media/5ea2b33de90e070498c5538c/Principles_of_Health_Systems_Resilience_in_the_Context_of_COVID_Research_Brief_April_2020.pdf

For peer review only

For peer review only - http://bmjopen.bmj.com/site/about/guidelines.xhtml

621 17. Van Damme W, Dahake R, Delamou A, et al. The COVID-19 pandemic: Diverse contexts;
622 Different epidemics - How and why? *BMJ Glob Heal*. 2020;5(7):1–16.

623 18. The World Bank. Liberia poverty headcount ratio [Internet]. 2016. Available from:
624 https://data.worldbank.org/indicator/SI.POV.DDAY?locations=LR

625 19. UNDP. Human Development Report 2020: The Next Frontier Human Development and the
626 Anthropocene [Internet]. UNDP: New York, NY, USA. 2020. Available from:
627 http://hdr.undp.org/sites/all/themes/hdr_theme/country-notes/LBR.pdf

628 20. Ling EJ, Larson E, Macauley RJ, et al. Beyond the crisis : did the Ebola epidemic improve
629 resilience of Liberia ' s health system? *Health Policy Plan*. 2017;32:40–7.

630 21. Ministry of Health and Social Welfare (MoHSW) L. Liberia National Community Health
631 Services Policy.

632 22. Ministry of Health Liberia. Investment plan for building a resilient health system in Liberia
633 [Internet]. Vol. 12, Monrovia, Ministry of Health. Monrovia, Liberia; 2015. Available from:
634 https://www.resilientinstitutionsafrica.org/sites/default/files/2018-09/%5BLIBERIA%5D
635 Investment Plan for Building a Resilient Health System %282015-2021%29.pdf

636 23. Liverpool City Council. Liverpool City Council: public health transformation six years on
637 [Internet]. 2019. Available from: https://www.local.gov.uk/liverpool-city-council-public-
638 health-transformation-six-years

639 24. Statista. Number of coronavirus (COVID-19) cases in the United Kingdom (UK) as of April 5,
640 2021, by country/region [Internet]. 2020 [cited 2021 Jun 7]. Available from:
641 https://www.statista.com/statistics/1102151/coronavirus-cases-by-region-in-the-uk/

642 25. Mayor of Liverpool. A STRONG AND GROWING CITY BUILT ON FAIRNESS. 2018.

643 26. University of Liverpool, Public Health England, Joint Biosecurity Centre, et al. Liverpool Covid-

For peer review only

For peer review only - http://bmjopen.bmj.com/site/about/guidelines.xhtml

644 SMART Community Testing Pilot [Internet]. Liverpool; 2021. Available from:
645 https://www.liverpool.ac.uk/media/livacuk/research/Mass,testing,evaluation.pdf

646 27. Sanny JA-N, Bloh O. Liberians laud government ' s COVID -19 response but claim unfair
647 distribution of relief assistance. *Afrobarometer*. 2021;(420):1–16.

648 28. Kateh F. How Ebola helped Liberia prepare for covid [Internet]. MIT Technology Review.
649 2020. Available from: https://www.technologyreview.com/2020/08/19/1007139/liberia-
650 kateh-covid-coronavirus-ebola/

651 29. Parker C, Zawolo G, Kollie J, et al. PRINCIPLES FOR PROMOTING RESILIENT HEALTH SYSTEMS
652 COVID-19 RESPONSE : LEARNING FROM LIBERIA Principle One : Develop flexible. 2020;

653 30. Stevens S, Pritchard A. Letter to Chief Executives of all NHS trusts and foundation trusts
654 regarding second phase of NHS response to COVID19 [Internet]. NHS; 2020 p. 1–9. Available
655 from: https://www.england.nhs.uk/coronavirus/wp-
656 content/uploads/sites/52/2020/04/second-phase-of-nhs-response-to-covid-19-letter-to-
657 chief-execs-29-april-2020.pdf

658 31. Rosenbaum L. Medicine a nd Society The Untold Toll — The Pandemic ' s Effects on Patients
659 without Covid-19. *N Engl J Med*. 2020;1–4.

660 32. Pope C, Mays N. Qualitative Research: Reaching the parts other methods cannot reach: an
661 introduction to qualitative methods in health and health services research. *BMJ*. 1995 Jul
662 1;311:42–5.

663 33. Kuper A, Reeves S, Levinson W. An introduction to reading and appraising qualitative
664 research. *BMJ Br Med J*. 2008;337:404–9.

665 34. Gilson L, Barasa E, Nxumalo N, et al. Everyday resilience in district health systems: emerging
666 insights from the front lines in Kenya and South Africa. *BMJ Glob Heal* [Internet].
667 2017;2(2):e000224. Available from: http://gh.bmj.com/lookup/doi/10.1136/bmjgh-2016-

For peer review only - http://bmjopen.bmj.com/site/about/guidelines.xhtml

000224

35. Biddle L, Wahedi K, Bozorgmehr K. Health system resilience: a literature review of empirical research. *Health Policy Plan*. 2020;1–26.

36. Sheikh K, George A, Gilson L. People-centred science: strengthening the practice of health policy and systems research. *Heal Res Policy Syst*. 2014;12(1):19.

37. Sheikh K, Ranson MK, Gilson L. Explorations on people centredness in health systems. *Health Policy Plan*. 2014;29(Suppl 2):ii1.

38. Gilson L, Barasa E, Nxumalo N, et al. Everyday resilience in district health systems: Emerging insights from the front lines in Kenya and South Africa. *BMJ Glob Heal*. 2017;2(2):1–15.

39. Newton-lewis T, Witter S, Fortnam M, et al. Working Paper : What is a Shock-Responsive Health System ? *Maint Res Support Soc Serv to Adapt to Shock*. 2020;(June):1–11.

40. Blanchet K, Nam SL, Ramalingam B, et al. Governance and capacity to manage resilience of health systems: Towards a new conceptual framework. *Int J Heal Policy Manag* [Internet]. 2017;6(8):431–5. Available from: http://dx.doi.org/10.15171/ijhpm.2017.36

41. Kruk ME, Ling EJ, Bitton A, et al. Building resilient health systems: A proposal for a resilience index. *BMJ* [Internet]. 2017;357(May):1–8. Available from: http://dx.doi.org/doi:10.1136/bmj.j2323

42. Barasa EW, Cloete K, Gilson L. From bouncing back, to nurturing emergence: Reframing the concept of resilience in health systems strengthening. *Health Policy Plan*. 2017;32(1):iii91–4.

43. Starfield B. Is patient-centered care the same as person-focused care? *Perm J*. 2011;15(2):63.

44. World Health Organization (WHO). The WHO global strategy on people- centred integrated health services services delivery and safety department [Internet]. Service Delivery and Safety. 2015. Available from:

For peer review only

For peer review only - http://bmjopen.bmj.com/site/about/guidelines.xhtml

https://apps.who.int/iris/bitstream/handle/10665/155002/WHO_HIS_SDS_2015.6_eng.pdf?sequence=1&isAllowed=y

45. Saunders B, Sim J, Kingstone T, et al. Saturation in qualitative research: exploring its conceptualization and operationalization. *Qual Quant*. 2018;52(4):1893–907.

46. Fereday J, Muir-Cochrane E. Demonstrating Rigor Using Thematic Analysis: A Hybrid Approach of Inductive and Deductive Coding and Theme Development. *Int J Qual Methods*. 2006;5(1):80–92.

47. Roberts K, Dowell A, Nie JB. Attempting rigour and replicability in thematic analysis of qualitative research data; A case study of codebook development. *BMC Med Res Methodol*. 2019;19(1):1–8.

48. Ritchie J, Lewis J, Nicholls CM, et al. The Foundation of Qualitative Research. *Qual Res Pract A Guid Soc Sci Students Res*. 2013;0–25.

49. Gale NK, Heath G, Cameron E, et al. Using the framework method for the analysis of qualitative data in multi-disciplinary health research. *BMC Med Res Methodol* [Internet]. 2013 Jan [cited 2014 May 1];13(1):117. Available from: http://www.pubmedcentral.nih.gov/articlerender.fcgi?artid=3848812&tool=pmcentrez&rendertype=abstract

50. Alhassan Y, McCollum R, Watson V, et al. PRINCIPLES FOR PROMOTING RESILIENT HEALTH SYSTEMS COVID-19 RESPONSE : [Internet]. 2021 [cited 2021 Jun 7]. Available from: https://www.redressliberia.org/wp-content/uploads/2021/06/Merseyside-Policy-Brief.pdf

51. Akseer N, Kandru G, Keats EC, et al. COVID-19 pandemic and mitigation strategies: Implications for maternal and child health and nutrition. *Am J Clin Nutr*. 2020;112(2):251–6.

52. Rajan S, Chakrabarti D, Akhtar N, et al. Searching for answers: Cancer care during the COVID pandemic. *Clin Epidemiol Glob Heal*. 2021;10(January).

For peer review only - http://bmjopen.bmj.com/site/about/guidelines.xhtml

53. Thorlby R, Fraser C, Gardner T. Non-COVID-19 NHS Care During the Pandemic [Internet]. The Health Foundation. 2020. Available from: https://www.health.org.uk/news-and-comment/charts-and-infographics/non-covid-19-nhs-care-during-the-pandemic

54. Elston JWT, Cartwright C, Ndumbi P, et al. The health impact of the 2014 - 15 Ebola outbreak. *Public Health* [Internet]. 2016;143:60–70. Available from: http://dx.doi.org/10.1016/j.puhe.2016.10.020

55. Golan MS, Jernegan LH, Linkov I. Trends and applications of resilience analytics in supply chain modeling: systematic literature review in the context of the COVID-19 pandemic. *Environ Syst Decis* [Internet]. 2020;40(2):222–43. Available from: https://doi.org/10.1007/s10669-020-09777-w

56. Witter S, Palmer N, Balabanova D, et al. Evidence review of what works for health system strenthening, where and when? 2021;(February):99. Available from: https://rebuildconsortium.com/resources/research-reports/evidence-review-of-what-works-for-health-systems-strengthening-where-and-when/

57. Dean L, Cooper J, Wurie H, et al. Psychological resilience, fragility and the health workforce: Lessons on pandemic preparedness from Liberia and Sierra Leone. *BMJ Glob Heal*. 2020;5(9).

58. Tengbe S. World Mental Health Day 2020 one LSTM student's research on mental health amongst health workers in Sierra Leone [Internet]. Available from: https://www.lstmed.ac.uk/news-events/news/world-mental-health-day-2020-one-lstm-student's-research-on-mental-health-amongst

59. Zhang WR, Wang K, Yin L, et al. Mental Health and Psychosocial Problems of Medical Health Workers during the COVID-19 Epidemic in China. *Psychother Psychosom*. 2020;89(4):242–50.

60. Hanefeld J, Mayhew S, Legido-Quigley H, et al. Towards an understanding of resilience: Responding to health systems shocks. *Health Policy Plan*. 2018;33(3):355–67.

For peer review only

61. Chua AQ, Tan MMJ, Verma M, et al. Health system resilience in managing the COVID-19 pandemic: lessons from Singapore. *BMJ Glob Heal*. 2020;5(9):e003317.

62. Sharma A, Borah S, Moses A. Responses to COVID-19: The role of governance, healthcare infrastructure, and learning from past pandemics. *J Bus Res*. 2021;(January):597–607.

63. UNICEF. Children and AIDS: third stocktaking report. TT - [Internet]. New York, New York, UNICEF, 2008.; 2008. Available from: http://www.unicef.org/devpro/files/CnA_TSR_LoRes_PDF_EN_USLetter_11202008.pdf

64. Ministry of Health Liberia. Liberia COVID-19 Daily Sitrep 510, 7th August 2021. Monrovia, Liberia; 2021.

For peer review only

For peer review only - http://bmjopen.bmj.com/site/about/guidelines.xhtml

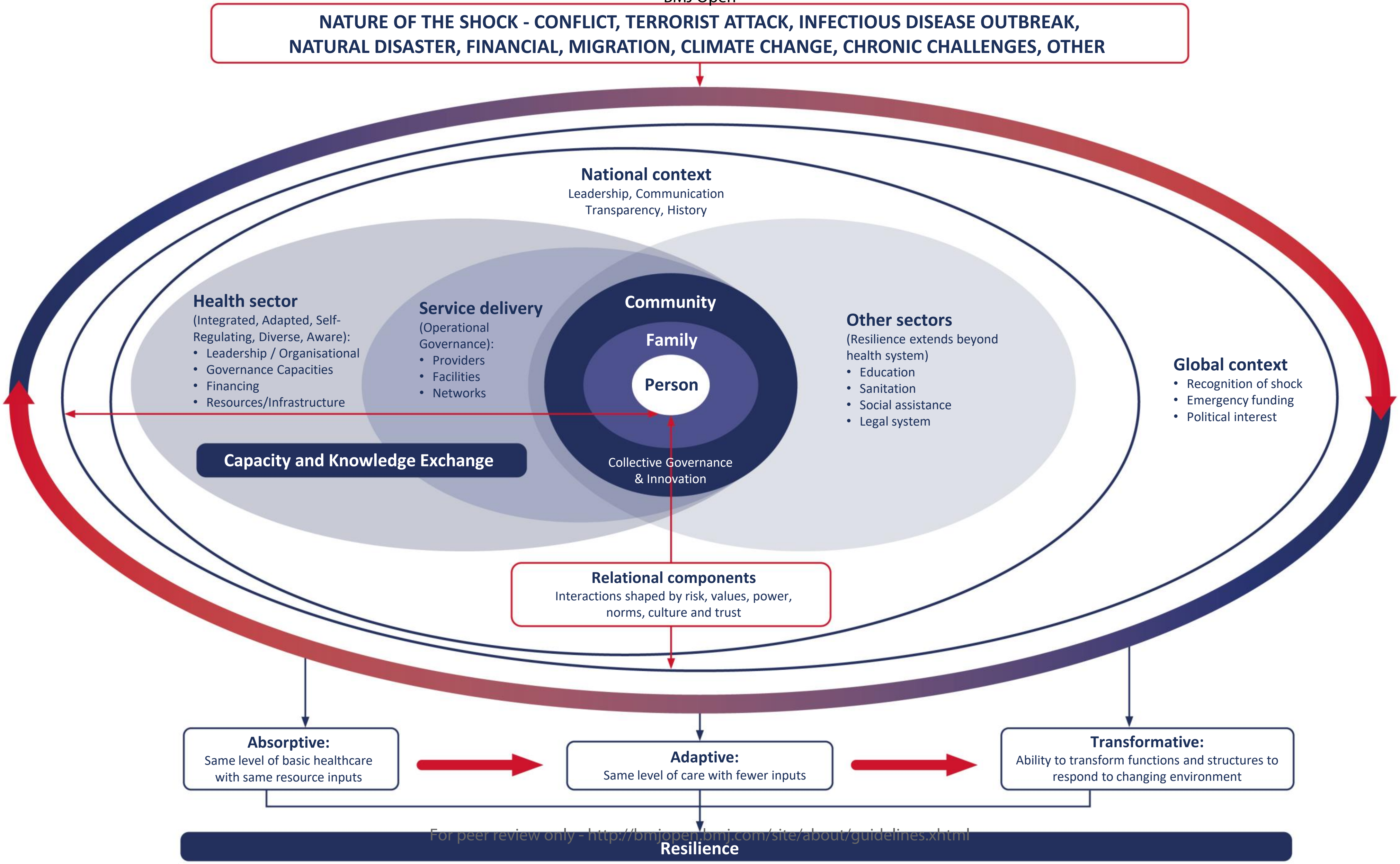

For peer review only - http://bmjopen.bmj.com/site/about/guidelines.xhtml

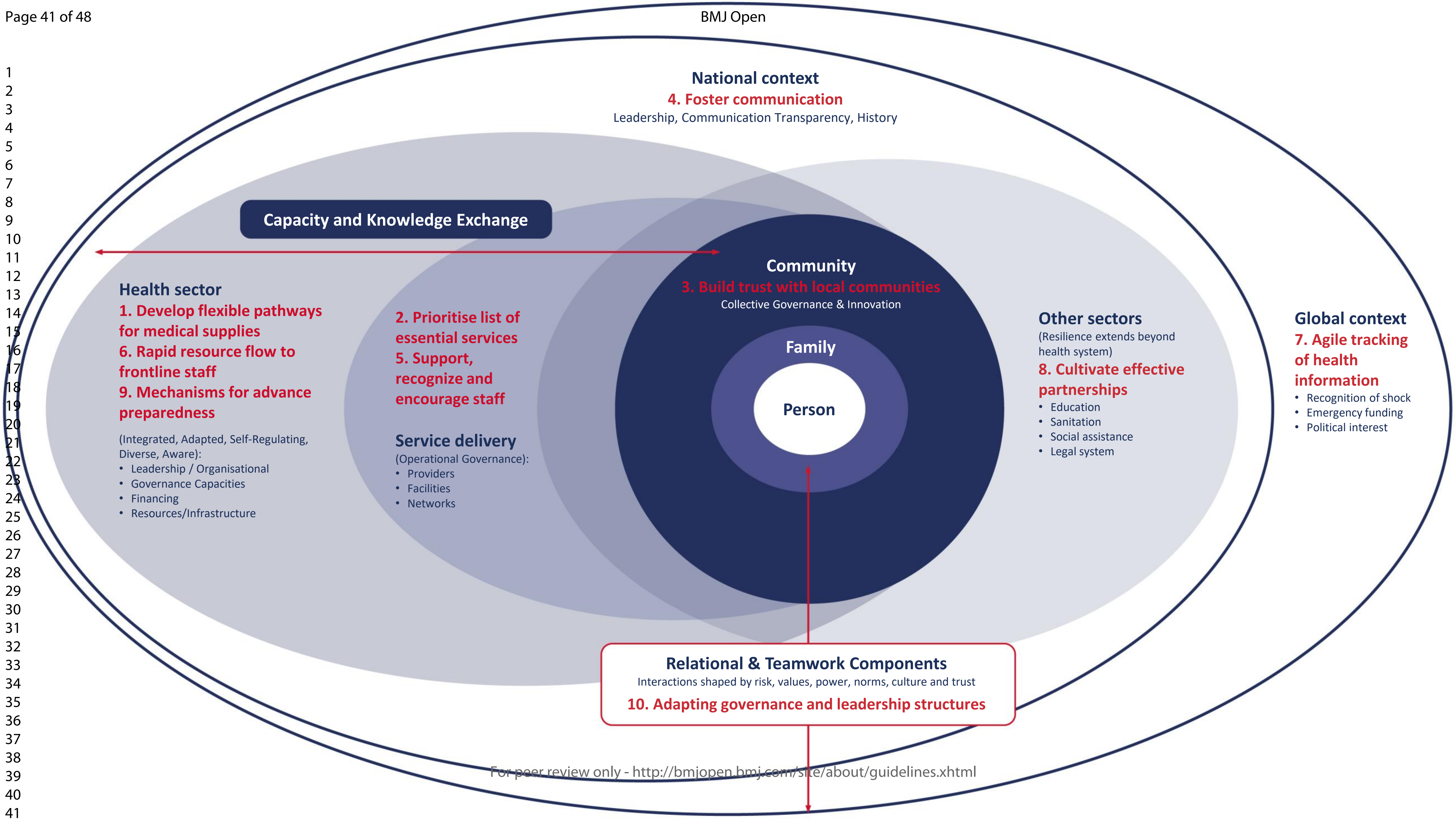

For peer review only - http://bmjopen.bmj.com/site/about/guidelines.xhtml

Appendix 1: COVID-19 Key Informant Interview Topic Guides

Contents

Appendix 1: COVID-19 Key Informant Interview Topic Guides 1

Key Informant Interviews Topic Guide –MOH Liberia 2

Key Informant Interviews Topic Guide –Merseyside Regional Decision Makers 4

Key Informant Interviews Topic Guide – Health Workers 6

Key Informant Interviews Topic Guide –Merseyside Laboratory and Blood Transfusion Staff 8

For peer review only

For peer review only - http://bmjopen.bmj.com/site/about/guidelines.xhtml

Key Informant Interviews Topic Guide –MOH Liberia

All possible questions to be asked of key informants are described in the following guide. Prior to interviewing each stakeholder, specific guides for these individuals will be made. One interview that covers relevant research themes will be completed with each stakeholder. Ordering of questions will also be revised to ensure logical flow through the interview and to avoid repetition.

Background

Please can you tell me your position and how long you have worked in your current role?
How has your role changed due to the current COVID-19 crisis?

Responses to Shock and the General Health System

1. How do you think the health system has coped with the COVID-19 crisis? How did it compare with previous crisis? How have routine services been impacted?
2. How is the current shock (COVID-19) the health system is experiencing similar or different to those you have experienced before?
3. What are the key learnings from previous shocks (Ebola/ conflict/ economic crisis)? How are they being used to respond now?
4. How do you think routine health systems functions are being impacted by the current crisis (COVID19/economic)?
5. What do you think could be done to support continuation of routine services? How is this informed or shaped by learnings from during the Ebola period?
    a. How would you describe the quality of services usually? How is quality of care being maintained throughout the COVID-19 response?

6. What policy or guidelines are supporting with the current COVID response? What additional guidelines or policies could be helpful for the COVID response?

Service Specific Impacts

Questions in this section to be reviewed/modified for cross-cutting MOH functions, e.g. M&E, research division prior to starting interview

7. Can you tell me about how service delivery within your programme/section (adapt to include name of section depending on who talking too) has been affected by the COVID pandemic?
    a. Which of your services would you say have been most impacted so far? Why?
    b. Which services would you envisage will be most impacted moving forwards? Why?
8. How have your routine services been modified or adapted? Which components of your service do you view as essential? Why?
9. Which specific sub-populations is routine care most impacted for? Are there any marginalised groups who may struggle to use services since the onset of the COVID-19 crisis? (Probe: e.g gender, dis/ability, rural/urban; wealth; geographic regions; age etc)
10. Have there been any innovations within service delivery in response to the COVID-19 crisis, and have they been useful in any way?
11. Has there been any innovations in response to COVID-19 that have concerned you?

Human Resource Management

12. How have you planned for staffing to meet the changing additional workload in response to COVID? Any tools/ guidance from the human resource section? Successes and challenges? (Prompt for role of new community health cadres, for those providing face to face care and for MOH staff)
13. What additional skill development have you provided and how in response to COVID? Successes and challenges?
14. How are you able to support staff so they can continue to work effectively during the COVID pandemic
    a. How have you supported staff through communication?
    b. How have you supported staff for occupational safety including PPE?

COVID-19 Key Informant Interview Topic Guides

c. How have you supported staff through with psychosocial support?
d. What have been the successes and challenges with supporting staff?

Service and System Impacts: Governance and Decision Making
Questions in this section to be reviewed/modified to make these questions more service-specific, depending on the interviewee's programme area

15. How are decisions made about which services should or should not be prioritised as part of the COVID response? (prompt for in relation to their specific service and also in relation to general health system, prompt for donor influence)
16. How does decision-making as part of the COVID response influence routine planning activities? What has been the impact of resource re-distribution as part of the COVID response?
17. Who is involved in this decision making and what are the processes? What are the challenges?

18. <u>What do you think are the key ethical impacts of making these decisions? What ethical guidelines are currently in place and important in decision making during this period?</u>
19. <u>What guidance documents are available to support you in making decisions regarding COVID?</u>
20. <u>What guidance documents would help to support maintaining routine services?</u>

Closing Questions

21. What does a resilient health system look like to you? What are your three recommendations would you make to improve or maintain the resilience of the Liberian health system during this period?
22. What are your three recommendations would you make post crisis to ensure the return to routine function of the health system as effectively as possible?

Additional questions for Director of personnel only

23. What are the main sources of additional staffing (e.g. secondment/redeployment, task-shifting, improved productivity, early graduation/students, returnees, volunteers)? Successes and challenges? Optional: Impact on the wage bill?
24. What areas of service are now struggling with staffing?
25. What are you able to do to retain staff? Successes and challenges?
26. What impact did/is down-sizing of "non-essential staff' have on your programme during the crisis?

Thank-you
Any other comments?

For peer review only - http://bmjopen.bmj.com/site/about/guidelines.xhtml

Key Informant Interviews Topic Guide –Merseyside Regional Decision Makers

Version1.1_01052020

All possible questions to be asked of key informants are described in the following guide. Prior to interviewing each stakeholder, specific guides for these individuals will be made. One interview that covers relevant research themes will be completed with each stakeholder. Ordering of questions will also be revised to ensure logical flow through the interview and to avoid repetition.

Question List

Background

Please can you tell me your position and how long you have worked in your current role?

Impact of COVID 19 on Routine Service Delivery

1. What are defined as essential routine services?
2. Which are the main scheduled and unscheduled services affected by COVID-19 and how have these been adapted over time?
3. Have there been any innovations within service delivery, and what have these been?
4. Have there been any changes that have concerned you? Why?
5. What would help to support maintaining routine services?

Governance and Decision Making

6. What has informed your decision-making, such as guidance documents or governance decision-making processes?
7. Who is involved in decisions made about which services should or should not be prioritised?
8. How are decisions made about which services should or should not be prioritised?
9. Describe how and who is involved in operationalising decisions?
10. What challenges have you faced in making these decisions?
11. What are the main differences between various sites in the trust, especially between Aintree and the Royal Hospitals?
12. How are changes in service delivery communicated? How can this be improved? There are multiple guidelines at national and local levels, how are these disseminated? How well does this work? How rapidly? How do health care workers respond to these changes?

Human Resource Management

13. How have you [may be the employer in general] planned for staffing to meet the changing additional workload? Any tools/ guidance from national authorities? Successes and challenges?
14. How have you planned for the increase in staff absence?
15. What additional skill development have you provided and how? What have been the successes and challenges?
16. How are you able to support staff so they can continue to work effectively (e.g. communication, occupational safety including PPE, psychosocial support)? What have been the successes and challenges?

Recovery post COVID-19

17. Are there any COVID-19-related changes to routine health services that you think it would be useful to continue after COVID-19? Which ones and why?
18. What next steps do you believe should be taken now to support the health system to recover post COVID-19?

For peer review only - http://bmjopen.bmj.com/site/about/guidelines.xhtml

Thank-you

Do you have any further suggestions for improvements to delivery of routine services?

Any other comments?

For peer review only

COVID-19 Key Informant Interview Topic Guides

Key Informant Interviews Topic Guide – Health Workers

Version1.1_01052020

All possible questions to be asked of key informants are described in the following guide. Prior to interviewing each stakeholder, specific guides for these individuals will be made. One interview that covers relevant research themes will be completed with each stakeholder. Ordering of questions will also be revised to ensure logical flow through the interview and to avoid repetition.

Question List

Background

Please can you tell me your usual position and how long you have worked in that role?
Are you currently working in your usual role and department?
If no, what role and department are you now working in?

Impact of COVID 19 on Routine Essential Service Delivery

1. Can you tell me about how health service delivery has been affected by the COVID pandemic? What was the processes for this, how was it communicated and do you have any ideas about how this can be improved? How prepared did you feel for these?
2. What do you consider to be routine essential health services in your work?
3. Which are the main scheduled and unscheduled services affected by COVID-19 in your department and how have these been adapted over time?
4. What have been the strengths and challenges with these changes? How has quality been affected?
5. How should these changes be evaluated? What indicators should be used?
6. What is worrying you most about your service now?
7. Which services would you envisage will be most impacted moving forwards as the pandemic progresses? (e.g. hospital based, community care, disease specific services, etc) Why?
8. Who do you think are the people most impacted by the changes in routine service delivery? Would you say that patients with specific socio-demographic characteristics are more impacted by service disruption/ distortion than others? Why? (e.g. gender, dis/ability; rural/urban; wealth; geographic regions; age etc) What can be done to ensure that these patients can still use health services when they need them?

Ethics and Decision Making

9. Have you encountered any health systems issues which you found troubling since the start of the COVID-19 pandemic? Would you be willing to tell me more about these issues?
10. What is the impact of these issues on you as a health worker? What would be helpful to support you in dealing with these issues?
11. Do you know of any ethical guidelines in place to guide you as you make difficult decisions during this time? What are these? How are these ethical guidelines operationalised? Are they useful?
12. Have you been involved with making decisions about the changes to health services since the COVID-19 pandemic? What was your role in making these decisions? How were these decisions made?
13. When there are changes in how health services are delivered how are these communicated with you? How has this worked? What do you think is the best way to be informed?

Human Resource Management

14. How has your role changed since the start of the COVID-19 pandemic? What have been the successes and challenges with how your role has changed? Probe workload
15. Is there anything about your role that concerns you? What?
    a. Probe working outside are of expertise
    b. No indemnity if make an error
    c. Communication about working across disciplines

For peer review only - http://bmjopen.bmj.com/site/about/guidelines.xhtml

16. What preparation for the changes to your role have you had and how was it delivered (skills - key ones, psychological support)? What have been the successes and challenges?
    a. Probe PPE training
    b. COVID clinical training
    c. Support mechanisms
    d. Team formation
17. What kind of support (e.g. communication, occupational safety including PPE, psychosocial support) are you receiving to do your job from your team/manager/employer? What have been the successes and challenges?

Recovery post COVID-19

18 . Are there any COVID-19-related changes or innovations to routine health services that you think it would be useful to continue after COVID-19? Which ones and why?

19. What next steps do you believe should be taken now to support the health system to recover post COVID-19?

20. What is worrying you most as the response moves forward?

Thank-you

Do you have any further suggestions for improvements to delivery of routine services?

Any other comments?

For peer review only

For peer review only - http://bmjopen.bmj.com/site/about/guidelines.xhtml

Key Informant Interviews Topic Guide –Merseyside Laboratory and Blood Transfusion Staff

Version1.1_01052020

All possible questions to be asked of key informants are described in the following guide. Prior to interviewing each stakeholder, specific guides for these individuals will be made. One interview that covers relevant research themes will be completed with each stakeholder. Ordering of questions will also be revised to ensure logical flow through the interview and to avoid repetition.

Background

Please can you tell me your position and how long you have worked in your current role?

Governance and Decision Making - Relating Directly to COVID-19

1. What has been the decision-making process for the laboratory's response to COVID-19 testing services and when did discussions start around re-adjusting services for COVID-19?
2. Who held overall responsibility for how COVID-19 testing was going to be conducted at LCL?
3. In addition to PHE, have the Liverpool Clinical Laboratory services worked closely/ collaborated with any other external partners for COVID-19 testing? If so whom and in what capacity?

Governance and Decision Making - Relating to Maintaining Routine Service Delivery

4. How are decisions made about which services should or should not be prioritised; which ones were considered to be essential and why? Who is involved in this decision making? How were these decisions communicated?
5. What guidance documents were most useful to you in making these decisions? In what way were they useful?
6. What key challenges have you faced in making these decisions? Do you have any support needs here?

Impact of COVID-19 on Routine Laboratory Service Delivery

7. Can you tell me about how routine clinical laboratory service delivery has been affected by the COVID pandemic?

COVID-19 Testing service specific

8. How did the laboratories adapt to scale up COVID-19 testing? (analysers, staff capacity, staff training, standard operating procedures, risk assessments)
9. What challenges did the laboratory face when implementing COVID-19 testing? How were they overcome? What worked well? (e.g. resources, human resource, process change, governance, culture, leadership etc)
10. Which routine services would you envisage will be most impacted moving forwards? (e.g. hospital based-testing, disease specific services, etc) Why?

Recovery post COVID-19

11. Are there any COVID-19-related changes to the laboratory service that you think it would be useful to continue after COVID-19? Which ones and why?
12. What next steps do you believe should be taken now to support the laboratory system to recover post COVID-19?
13. Are there any changes/ innovations introduced in response to COVID-19 changes which you think should be continued? Why?

Thank you

Do you have any questions for me? Resources (re labs) link https://www.rcpath.org/uploads/assets/90111431-8aca-4614-b06633d07e2a3dd9/Guidance-and-SOP-COVID-19-Testing-NHS-Laboratories.pdf

For peer review only - http://bmjopen.bmj.com/site/about/guidelines.xhtml

Table 1 Standards for Reporting Qualitative Research (SRQR)

Standard	Page number
S1 Title	P1, line 1-2
S2 Abstract	P2, line12-35
S3 Problem formulation	P3 line 53-75
S4 Purpose of research question	P4, line 78-124
S5 Qualitative approach and research paradigm	P11 line229-232
S6 Research characteristics and reflexivity	P8 line 165-190
S7 Context	P7 line 129-164
S8 Sampling strategy	P10line200-212
S9 Ethical issues pertaining to human subjects	P11 line 255-262
S10 Data collection methods	P8 line 167-171
S11 Data collection instruments and technologies	P9 line 191-198
S12 Units of study	P10 line219-224
S13 Data processing	P11 line 224-226
S14 Data analysis	P11 line 232-249
S15 Techniques to enhance trustworthiness	P 11 line 226-227
S16 Synthesis and interpretation	P 13 line 267-410
S17 Links to empirical data	P13 line 267-272
S18 Integration with prior work, implications, transferability, and contributions to the field	P21 line 411-523
S19 Limitations	P27 line 524-542
S20 Conflicts of interest	P28 line 565-566
S21 Funding	P28 line558-562

For peer review only

BMJ Open

Qualitative study exploring lessons from Liberia and the UK for building a people-centred resilient health systems response to COVID-19

Journal:	*BMJ Open*
Manuscript ID	bmjopen-2021-058626.R2
Article Type:	Original research
Date Submitted by the Author:	15-Jun-2022

Complete List of Authors:	McCollum, Rosalind ; Liverpool School of Tropical Medicine, International Public Health<br>Zaizay, Zeela ; Actions Transforming Lives<br>Dean, Laura; Liverpool School of Tropical Medicine, Department of International Public Health<br>Watson, Victoria; Liverpool School of Tropical Medicine (LSTM), International Public Health<br>Frith, Lucy; The University of Manchester, Centre for Social Ethics & Policy, School of Law<br>Alhassan, Yussif; Liverpool School of Tropical Medicine, International Public Health<br>Kollie, Karsor; Ministry of Health<br>Piotrowski, Helen; Liverpool School of Tropical Medicine, Department of International Public Health<br>Bates, Imelda; Liverpool School of Tropical Medicine<br>Anderson De Cuevas, Rachel; University of Liverpool, Institute of Population Health<br>Harris, Rebecca ; University of Liverpool, Institute of Population Health<br>Chowdhury, Shahreen; Liverpool School of Tropical Medicine, International Public Health<br>Berrian, Hannah; University of Liberia-Pacific Institute for Research and Evaluation<br>Smith, John; University of Liberia-Pacific Institute for Research and Evaluation<br>Tate, Wede; University of Liberia-Pacific Institute for Research and Evaluation<br>El Hajj, Taghreed; Liverpool School of Tropical Medicine<br>Ozano, Kim; Liverpool School of Tropical Medicine<br>Hastie, Olivia; Liverpool School of Tropical Medicine<br>Parker, Colleen; Ministry of Health Liberia<br>Kollie, Jerry; University of Liberia-Pacific Institute for Research and Evaluation<br>Zawolo, Georgina; University of Liberia-Pacific Institute for Research and Evaluation<br>Ding, Yan; Liverpool School of Tropical Medicine, Centre for Capacity Research<br>Dacombe, Russell; Liverpool School of Tropical Medicine, International Public Health<br>Taegtmeyer, Miriam; Liverpool School of Tropical Medicine, International

	Public Health<br>Theobald, Sally ; Liverpool School of Tropical Medicine, International Public Health
**Primary Subject Heading**:	Global health
Secondary Subject Heading:	Health services research, Health policy
Keywords:	COVID-19, HEALTH SERVICES ADMINISTRATION & MANAGEMENT, PUBLIC HEALTH, QUALITATIVE RESEARCH

SCHOLARONE™
Manuscripts

I, the Submitting Author has the right to grant and does grant on behalf of all authors of the Work (as defined in the below author licence), an exclusive licence and/or a non-exclusive licence for contributions from authors who are: i) UK Crown employees; ii) where BMJ has agreed a CC-BY licence shall apply, and/or iii) in accordance with the terms applicable for US Federal Government officers or employees acting as part of their official duties; on a worldwide, perpetual, irrevocable, royalty-free basis to BMJ Publishing Group Ltd ("BMJ") its licensees and where the relevant Journal is co-owned by BMJ to the co-owners of the Journal, to publish the Work in this journal and any other BMJ products and to exploit all rights, as set out in our licence.

The Submitting Author accepts and understands that any supply made under these terms is made by BMJ to the Submitting Author unless you are acting as an employee on behalf of your employer or a postgraduate student of an affiliated institution which is paying any applicable article publishing charge ("APC") for Open Access articles. Where the Submitting Author wishes to make the Work available on an Open Access basis (and intends to pay the relevant APC), the terms of reuse of such Open Access shall be governed by a Creative Commons licence – details of these licences and which Creative Commons licence will apply to this Work are set out in our licence referred to above.

Other than as permitted in any relevant BMJ Author's Self Archiving Policies, I confirm this Work has not been accepted for publication elsewhere, is not being considered for publication elsewhere and does not duplicate material already published. I confirm all authors consent to publication of this Work and authorise the granting of this licence.

For peer review only

Title Qualitative study exploring lessons from Liberia and the UK for building a people-centred resilient health systems response to COVID-19

Authors: Rosalind McCollum*^, Zeela Zaizay*, Laura Dean, Victoria Watson, Lucy Frith, Yussif Alhassan, Karsor Kollie, Helen Piotrowski, Imelda Bates, Rachel Anderson De Cuevas, Rebecca Harris, Shahreen Chowdhury, Hannah Berrian, John Smith, Wede Tate, Taghreed El Hajj, Kim Ozano, Olivia Hastie, Colleen Parker, Jerry Kollie, Georgina Zawolo, Yan Ding, Russell Dacombe, Miriam Taegtmeyer, Sally Theobald

*Joint first authors

^Corresponding author email address: rosalindmccollum@gmail.com

For peer review only

For peer review only - http://bmjopen.bmj.com/site/about/guidelines.xhtml

Abstract

Introduction: COVID-19 has tested the resilience of health systems globally and exposed existing strengths and weaknesses. We sought to understand health systems COVID-19 adaptations and decision-making in Liberia and Merseyside, UK.

Methods: We used people centred-approach to carry out qualitative interviews with 24 health decision-makers at National and County Level in Liberia and 42 actors at County and hospital level in the UK (Merseyside). We explored health systems' decision-making processes and capacity to adapt and continue essential service delivery in response to COVID-19 in both contexts.

Results: Study respondents in Liberia and Merseyside had similar experiences in responding to COVID-19, despite significant differences in health systems context, and there is an opportunity for multi-directional learning between the global south and north. The need for early preparedness; strong community engagement; clear communication within the health system, and health service delivery adaptations for essential health services emerged strongly in both settings. We found the Foreign, Commonwealth and Development Office (FCDO) principles to have value as a framework for reviewing health systems changes, across settings in response to a shock such as a pandemic. In addition to the eight original principles, we expanded to include two additional principles; 1) the need for functional structures and mechanisms for preparation and 2) adaptable governance and leadership structures to facilitate timely decision-making and response coordination. We find the use of a people-centred approach also has value to prompt policy makers to consider the acceptance of service adaptations by, patients and health workers, and to continue the provision of 'routine services' for individuals during health systems shocks.

Conclusion: Our study highlights the importance of a people-centred approach, placing the person at the centre of the health system, and value in applying and adapting the FCDO principles across diverse settings.

For peer review only - http://bmjopen.bmj.com/site/about/guidelines.xhtml

Strengths and Limitations of the Study

- A key strength of this study is the multi-directional learning between health systems in the global south and global north, which involved a wide range of researchers across both settings, and the breadth of perspectives captured from frontline staff and key decision-makers.
- We find that the FCDO principles can be usefully applied across diverse contexts, with identification of two additional new principles, related to mechanisms for advanced preparedness and adaptable governance and leadership structures.
- The greatest limitation of this study is that it was carried out at a single point in time, towards the end of the first wave in the UK and before there had been a large increase in cases in Liberia. Response measures have evolved in both settings in subsequent stages of the pandemic.
- The study was limited by the differing range of respondents across study settings, with participants from across a range of health system levels including primary care, hospital frontline workers and decision-makers as well as regional decision-makers within Merseyside, UK; compared with national and county level decision-makers, technicians and supervisors of frontline staff in Liberia, which may result in differing perspectives.

For peer review only - http://bmjopen.bmj.com/site/about/guidelines.xhtml

Introduction

The COVID-19 pandemic has forever altered our world. Its impact has been felt across all nations, demonstrating the importance of resilient health systems in protecting global health security.[1] Health systems have been forced to adapt to new ways of working alongside the continued provision of essential services including: prevention of communicable diseases; sexual and reproductive health; care for vulnerable populations; ongoing management of chronic illness (including mental health conditions); continuity of critical inpatient therapies; management of emergency health conditions; and auxiliary services, including diagnostic imaging, laboratory and transfusion services.[2]

In April 2020, the United Nations expressed concern that, within Africa, up to 3.3 million people could lose their lives as a direct result of COVID-19 and many more through the indirect effects of disruption to health services and worsening socioeconomic conditions.[3] Conditions considered to increase the risk of infection include overcrowded and poorly serviced slum dwellings; limited access to basic handwashing facilities; high levels of informal employment limiting ability to work from home; high levels of malnutrition and lower ratios of beds and health workers to the population.[3] A commentary published by Agyeman et al. (2020) at the outset of the pandemic highlighted a rapid response within many African settings, including focus on early introduction of screening procedures at ports of entry, need for effective community engagement to educate about the mode of transmission. Key protective behaviours were emphasised, along with the need to prepare intensive care beds and clear government strategies regarding how to deal with hospitalised COVID-19 patients to avoid disrupting the health system and to prevent non-COVID-19 related deaths.[4] Subsequent studies have revealed that indirect health impacts from COVID-19 disproportionately impact women and children.[5,6] Diversion of resources (financial, material, human) from existing health services to address the pandemic, impacts their care.[5,6] This includes supply and demand side disruptions that can result in lower utilization of health care and, in some cases, impact on quality of care.[7] Bayani et al (2021) surmise that "less

For peer review only - http://bmjopen.bmj.com/site/about/guidelines.xhtml

health care will result in more ill health and deaths because health services have been suspended, displaced, or inaccessible."(page 5 [7])

Our study was carried out immediately following the first wave of COVID-19 in Liberia and UK (interviews carried out June to September 2020) in response to an expressed need by stakeholders for this research following dialogue in both contexts. The study was conducted within these two contexts (Merseyside region and Liberia) based on strong prior research relationships within both settings. The differing perspectives from national and county respondents speaking on the national response in Liberia, and frontline health workers and decision makers up to regional level in Merseyside based on their personal experiences and more localised regional response is a key limitation. We chose these settings due to the opportunity and demand for research, not because they are exemplars of COVID-19 response. There is, however, still opportunity for learning and comparison on both the strengths and weaknesses within the COVID-19 initial response in both settings. The pandemic has continued to evolve across both settings, with both Liberia and UK experiencing much larger waves of COVID-19 since this original study was carried out. These findings from the first wave can provide valuable lessons to inform continued response to COVID-19 and other health systems shocks.

The pandemic has revealed monopolies of knowledge production, which disempower lower and middle-income countries;[8] whilst pandemic responses in 'developed democracies' have been inadequate, with cuts to health and social services and limited commitment to equity or governance.[8] So-called "global powerhouses with tried and tested health systems have struggled to contain the COVID-19 pandemic"[9] and health systems have been stretched to the limit, resulting in negative implications for the health of all populations, particularly when access for patients with other acute and chronic illness is limited.[8] As of 01/09/21, UK (population 66.8 million)[10] has 6,821,356 confirmed cases and 132,859 COVID-19 related deaths.[11] In the UK, the National Health Service delivers care for most of the population. Meanwhile during the same time period, Liberia (population 4.9 million)[10] has had 5594 confirmed cases, with 245 confirmed COVID-19 related deaths.[11] There are marked differences

For peer review only - http://bmjopen.bmj.com/site/about/guidelines.xhtml

between settings in the roll-out and scope of testing capacity and uptake of this, with under-reporting in many lower middle income countries, and so these figures cannot be assumed to be accurate. Future comparisons will eventually show the magnitude of all-cause mortality by age, and firm conclusions can be made about the success of different country approaches. Liberia was, initially hailed as one of the top countries in fighting COVID-19, being one of the first countries to start screening at ports of entry (January 2020) and to adopt other control measures such as rapid testing, contact tracing and quarantine.[12,13]

"Improving resilience within health systems can build on pre-existing strengths to enhance the readiness of health system actors to respond to crises, while also maintaining core functions."(page 1 [1]). People-centred health systems are a critical framing in shaping resilience as they place people and communities at the centre whilst also promoting strategic and collaborative multi-sectoral leadership which is necessary in delivering a co-ordinated response to a public health crisis.[14] In this paper, we compare health systems responses at a single point in time (June to September 2020) within Monrovia, Liberia and Merseyside, UK to distil lessons for health systems resilience to a pandemic through comparative case studies which explore aspects of health systems resilience.[15] Within this paper we combine the Foreign, Commonwealth and Development Office (FCDO) eight key principles for promoting resilient health systems with key domains and values of people-centred health systems to frame our findings in relation to the COVID-19 response.[16] Through our discussion we reflect on these expanded principles for resilience against our conceptual framework (figure 1), which is based on a people-centred approach. In response to calls for on-the-ground analysis of the response to COVID-19 within the Global South and comparative case studies that use co-creation and coproduction approaches which go beyond researchers including policy makers, practitioners and the public,[15,17] we seek to share learning from the response within Liberia and the UK, along with opportunities for multi-directional knowledge sharing.[17] It is our hope that this paper will help inform health policy makers across global contexts, for the current pandemic response and as they plan towards more resilient people-centred health systems to meet future shocks.

For peer review only - http://bmjopen.bmj.com/site/about/guidelines.xhtml

Methods

Study context

Liberia and UK have had very different strategies and case rates from the outset of the pandemic, although there were some similarities in the adoption of infection prevention control measures across both contexts. Liberia is amongst the world's poorest in terms of GDP and living conditions. According to the World Bank 2016 poverty headcount ratio, 44.4% of Liberians live below the international poverty benchmark of $1.90 USD per day.[18] The UNDP Human Development Report 2020 ranks Liberia low at 175 out of 189 countries and territories.[19] Inequities between females and males are remarkable with literacy rates (secondary education) of 18.5% and 40.1% respectively.[19] Liberia has prior experiences of shocks in the form of two civil wars, and the 2014-2015 Ebola Virus Disease (EVD) epidemic.[20] In response to these experiences, Liberia has prioritised rebuilding a resilient health system, which acknowledges the critical role communities play in addressing their own health needs through the 'Investment Plan for Building a Resilient Health System in Liberia' and the community health services policy (2016-2021).[21,22] By contrast, Merseyside is a Metropolitan County in the North West of England, comprising five boroughs, including the City of Liverpool, including some of the most deprived council areas in England.[23] It has a population of 1.42 million and has had some of the highest numbers of COVID-19 cases in the UK.[24] Within Merseyside, the Liverpool City Region Combined Authority has prioritised tackling deprivation and reducing health inequalities through people-centred care, with integration of health and social care services.[25] Liverpool has a long history of public health innovation, but also a strong sense of local history, culture and place. Throughout the pandemic Liverpool has been at the forefront of community-based innovations and public health strategies, e.g. piloting community open access testing for COVID-19.[26]

Liberia introduced stringent border control measures from January 2020, with the establishment of a Special Presidential Advisory Committee on Coronavirus (SPACOC) over two months prior to the first recorded cases in the country.[27],[28] Liberia's response to COVID-19, prioritised a call to maintain

For peer review only - http://bmjopen.bmj.com/site/about/guidelines.xhtml

the delivery of routine health services at all levels. Hospitals and clinics continued to provide health services with health facility workers trained in infection prevention control (IPC) before the first case was identified in country.[28] Physical distancing measures were introduced and use of face masks encouraged.[29]

Within the UK, health service delivery was restructured as part of the COVID-19 response, with routine non-urgent elective care suspended and later re-started in April 2020.[30] Adaptations to minimise potential risk of COVID-19 infection include the use of telemedicine and phone consultations; and changes to essential services for patients, such as changed treatment plans and delays to surgeries.[31] Hospital patient pathways were altered to appropriately triage and cohort the care of COVID-19 patients, reducing the risk of transmission to others and allowing essential services to continue. There was also reduction in routine blood test screening to prioritize COVID-19 PCR testing in response to the UKs 'test and trace' strategy.

Study aim, design and conceptual framework

Aim: To understand COVID-19 adaptations and decision-making in Liberia and Merseyside, UK

This qualitative study explored inductively the differing experiences, perspectives and recommendations of participants in order to understand COVID-19 adaptations and decision-making in Liberia and Merseyside, UK.[32,33] We selected qualitative methods to give “due emphasis to the meanings, experiences, and views of all the participants”(page 43 [32]) and understand decision-making and the impact of health systems adaptations as a result of COVID-19.

A conceptual framework was jointly developed, following a series of meetings held with researchers in each setting (7 Liberia-based researchers and 18 UK-based researchers). This framework sought to consider a people-centred approach towards the health system’s ability to respond to shock, whilst reflecting the realities experienced in the face of multiple routine challenges (Figure 1).[34] The nature of a shock to the health system, whether due to infectious disease outbreak, natural disaster,

For peer review only - http://bmjopen.bmj.com/site/about/guidelines.xhtml

or conflict, influences the rest of the framework.[35] It adopts a people-centred approach at its heart,[14,36,37] while incorporating literature relating to the health system's ability to respond to a sudden shock, and the extent to which it is able to absorb, adapt and transform in response (Figure 1).[35,38–42]

People-centred health systems prioritise the collective right to health through integrated and targeted approaches that favour the needs of the most vulnerable.[14,43] Collective action and social solidarity are viewed as essential to the art and science of the development of people centred systems that are organised around people's health care needs and expectations as opposed to diseases, ensuring a continuum of care throughout the life course.[14] This approach embraces the human character of health systems, by viewing individuals, communities and health workers as co-producers of health care, placing people and families at the centre.[44] Systems must adapt to meet a range of challenges to support the development of strategies that seek to improve health care access and encourage universal coverage. This is particularly important as many individuals transition and oscillate between multiple roles of patient, family and sometimes health care provider within one system.

Interview topic guides were informed by the framework and developed across both settings to explore key areas of health systems functioning in response to COVID-19 (Appendix 1). Questions included: governance and decision-making; use of ethical guidelines; human resource management, infrastructure (information technology and communications) and health care worker support; introduction of innovations; and perceptions of the equity and quality of service delivery. Adaptations were made according to the health systems context in each country, for example in Liberia, additional questions were included to explore how learning from the EVD epidemic and other health systems shocks informed COVID-19 response planning.

Figure 1 placed here

<u>Study participants and data collection</u>

For peer review only - http://bmjopen.bmj.com/site/about/guidelines.xhtml

200 The study was carried out at different levels of the health system across both settings (Table 1). In Liberia, we conducted key informant interviews in June and July 2020 with 21 national level and three county level decision-makers (Nimba, Margibi and Montserrado Counties) purposively selected because of their involvement with COVID-19 planning and/or routine service delivery. Some had also played key roles in the EVD epidemic response. In Merseyside we conducted 42 key informant interviews between July to September 2020, with regional, hospital and primary care decision-makers (general practitioners and residential care home manager) and front-line workers selected because of their involvement with COVID-19 planning and/ or the delivery of COVID-19 or routine services (see Table 1). More interviews were carried out within the UK across health systems levels, due to demand for research across multiple levels and the presence of a larger team of researchers. In Liberia, by contrast the demand for research was focused at national level, and the research team was smaller in size. The national and county level actors in Liberia, spoke about Liberia's response as a country. In contrast study participants in Merseyside from across health systems levels, including frontline health workers, spoke of their own direct experience within a particular hospital or setting, or on behalf of Merseyside City Region. We acknowledge the limitation that including national and county level actors only within Liberia, creates a somewhat limited perspective. It would have been preferable to have included a larger number and range of participants from sub-national health systems levels to provide more depth of understanding about the COVID-19 response.

218 *Table 1 Study participants' role*

Participant Role	Number of Participants Interviewed
**Merseyside, UK**	
Regional decision-maker	5
Hospital decision-maker (Clinical director, medical director, ward manager)	4
Hospital consultant	11
Hospital health worker (junior doctors, nurses)	10
Health worker in community (GP, district nurse, residential care home)	7
Liverpool Clinical Laboratory staff	5
**Total**	**42**

For peer review only - http://bmjopen.bmj.com/site/about/guidelines.xhtml

**Liberia participants**	
National decision-maker	21
County decision-maker	3
**Total**	**24**

Interviews were predominantly carried out remotely by researchers experienced in qualitative interviewing in English language, via online platforms such as Microsoft Teams or Skype. A minority were carried out in person with physical distancing measures in place, according to local guidance at the time. All interviews were audio-recorded. Data collection stopped when no new themes emerged from additional data collected.[45] Interviews lasted approximately 30 to 60 minutes. Audio recordings were transcribed verbatim, with quality assurance conducted by a second researcher against the recording.

Data Analysis

The study has sought to use a pragmatic approach to research, working through existing networks to carry out timely research to support the ongoing COVID-19 response in both settings. Both inductive and deductive approaches were blended within data analysis, in keeping with other health systems research [46–49]. In both Liberia and UK, preliminary data analysis workshops were held separately with the research team members involved with data collection. Prior to the workshops all participants reviewed transcripts to familiarise and immerse themselves within the data in order to inductively identify emerging themes which arose from within the study findings. Through these separate country workshops key themes were identified and used to generate a separate coding framework for each setting. All transcripts were imported into NVivo Version 12 qualitative data analysis software for coding (QSR International Pty Ltd. Version 12, 2018). Following review of the initial themes which emerged inductively from within the data, there was found to be strong alignment with the eight FCDO principles. These principles were then deductively applied to assist with mapping the findings and enabling comparison between settings. The research team did not simply accept the eight FCDO principles, rather the team reviewed them and found that they did not fully cover all the aspects of

For peer review only

For peer review only - http://bmjopen.bmj.com/site/about/guidelines.xhtml

resilience which emerged from the data. As a result two further principles were identified and applied to adequately compare findings between both settings, relating to "mechanisms for advance preparation" (Principle 9) and "adaptable governance and leadership structures" (Principle 10). The application of the expanded FCDO principles for resilience has helped to showcase how Liberia's experience with responding to prior shocks and their learned need for early advance preparedness provided an important element working towards resilience. This study is not funded by FCDO, nor were FCDO involved in any way as researchers or co-authors within the research team.

Detailed findings and recommendations were developed into two policy briefs in accordance with these expanded principles for resilience and were shared and discussed with relevant stakeholders from both study settings.[29,50] The relationship of the findings to the original conceptual framework was reviewed and findings compared between settings during a final on-line workshop, attended by all those involved with data collection in both settings, with key similarities and differences jointly discussed.

Ethics

Ethical approval was received from the Liverpool School of Tropical Medicine Research Ethics Committee (Protocol ID 20-045); the University of Liverpool Ethics Committee (Reference 7811) and the University of Liberia-Pacific Institute for Research and Evaluation Institutional Review Board; National Health Service Health Research Authority and Health and Care Research, Research Ethics Committee (Reference 20/HRA/2597); Integrated Research Application System (Project ID 284143). All study participants were provided with a participation information leaflet at least 48 hours prior to interview. All participants provided written, or audio recorded consent to participate.

Patient and public involvement

Neither patients nor the general public were involved in the design, conduct, reporting or dissemination of our research.

For peer review only - http://bmjopen.bmj.com/site/about/guidelines.xhtml

Results

We present findings according to the expanded FCDO principles for resilience (Box 1) (key illustrative quotes are summarised for each principle in table 2). We then reflect on the findings in light of people-centred health systems within the discussion.

Box 1 ExpandedPrinciples of Health Systems Resilience in the Context of COVID-19 Response

Principle 1 Develop flexible pathways for medical supplies
Principle 2 Prioritise a list of essential health services [*and continued provision of quality and equitable routine services*]
Principle 3 Build trust with local communities
Principle 4 Foster good communication at all system levels
Principle 5 Support, recognise and encourage staff
Principle 6 Facilitate rapid resource flow and greater flexibility in its use
Principle 7 Ensure agile tracking of health information
Principle 8 Cultivate effective partnerships and networks
Principle 9 Structures and mechanisms for advanced preparedness (**New principle**)
Principle 10 Adapt governance and leadership structures to facilitate timely decision-making and effective coordination of response **(New principle)**

Table 2 Illustrative quotations from Liberia and Merseyside related to each FCDO Principle

**Principle**	**Comparison**	**Quotations**
**Principle 1: Develop flexible pathways for medical supplies**	Supply chains disturbed across settings due to global shortages and price inflation.<br>Lack of buffer stock in both settings.<br>Restructuring of supply chains in Liberia led to disturbance for routine supplies.	*"Supply chain are affected greatly because their concentration is on how to provide the COVID response activities meaning the …medicines and medical supplies that are needed [for] NTDs (Neglected Tropical Diseases), lack of attention will now be paid to that." (LIB national decision maker 029)*<br>*"With regards to PPE, there was national guidance about what we should do and there was a huge amount of fear amongst nurses and medics and everyone else understandably. Everyone was scared. I was scared. If someone said they weren't scared, then they're lying or they're a fool. The national guidance was confused, and availability of PPE fluctuated. Procurement here [NHS hospital] did a very good job, but sometimes it just wasn't delivered nationally. And we went through other supply chains…" (LIV hospital decision maker, Merseyside UK 014)*
**Principle 2: Prioritise a list of essential health services [and continued provision of quality and equitable routine services]**	Discontinuation of elective non-urgent care in UK, contrasts with early emphasis on continued routine care in Liberia.	*"So we just have to be robust and do the necessary investment into routine health services, preventive in terms of creating awareness and education among health workers about covid and how we can continue to care for our patients, with fighting the infection at the same time." (LIB national decision maker 001)*

For peer review only - http://bmjopen.bmj.com/site/about/guidelines.xhtml

Principle	Comparison	Quotations
		"There's the whole big risk around the screening program…the screening program was stopped, restarting that it's gonna be really challenging. And I suppose that's another risk in terms of people with delayed diagnosis and the right treatment, as a result of not having had that screening mammograms." (LIV hospital decision maker Merseyside UK 051)
**Principle 3: Build trust with local communities**	Both settings experiences reduced service utilisation due to loss in community trust. Introduction of innovative follow-up visits to patients led to increased service use in Liberia.	*"Some of the useful things that we have been using from Ebola time is, as I said before, to involve the communities …The community aspect is very important because it will help us for the COVID-19 where communities, family members, all of those at the community level are influential group they will be able to comply like we did in the Ebola." (LIB national decision maker 005)* *"The elderly population have been shielding because of comorbidities and all that. I think they probably not being as vocal about things that they're concerned about because they're worried about that they will be asked to come in. They fear that that they will catch Covid when they come here." (LIV hospital health worker Merseyside UK 048)*
**Principle 4: Foster good communication at all system levels**	Expansion of virtual communication in both settings. In Merseyside frequently changing guidance from multiple sources created confusion.	*"One of the things that quickly used to come to me is to able to adapt to working with social media technology and all of that, because that's the first thing if you have to communicate with people in this manner you need to understand zooming, skyping, how to take notes.." (LIB national decision maker 029)* *"And there's so many different sources of information that say different things from what people hear within the hospital talking to friends on the corridor, that you've got to come out with a consistent message. And I think it took longer than was ideal to get a central source of information…But people need to be told what the situation is rather than try to be falsely reassured sometimes as well." (LIV hospital decision maker, Merseyside UK 004)*
**Principle 5: Support, recognise and encourage staff**	Health worker redeployment was common across settings. Health worker training varied in UK according to cadre.	*"Like take for example, when COVID came some of our workers from the [name] Hospital was recruited to go at the front line and [hospital name] is for routine services so taking employees from there to go at the front line that tells you it kind of understaff… So routine services kind of slow down and every attention was placed on COVID but going forward, with the system in place, routine services have gotten back on its feet." (LIB national decision maker 010)* *"And it felt like there was unequal share of knowledge and also an unequal kind of confidence in protective clothing. … And I think the people that spent the most time with the patient, the patient*

For peer review only - http://bmjopen.bmj.com/site/about/guidelines.xhtml

Principle	Comparison	Quotations
		areas, for instance, the health care assistants and the cleaning staff didn't have all of the information [at the] beginning or any PPE training." (LIV hospital health worker Merseyside UK 017)
**Principle 6: Facilitate rapid resource flow and greater flexibility in it's use**	Prior under-investment in health was common across settings.<br>In Merseyside there was increased funding available and removal of bottlenecks, which enabled swifter action.	*"The first thing is, we need ownership by government, ownership is not depending on other countries to provide us the resources, to provide the technical capacity. So that is the best recommendation I would say. The ownership has to be there, resources have to be available and the infrastructure has to be available in terms of being resilient." (LIB national decision maker 029)*<br>*"To be honest, it was a fairly novel experience because it was a situation where if we asked we more or less got [funding]." (LIV hospital decision maker, Merseyside UK 004)*
**Principle 7: Ensure agile tracking of health information**	Data quality reduced in Liberia.<br>In Merseyside increased data was collected, but inadequate data analysis measures were put in place.	*"Another recommendation is that we could include COVID-19 to our regular disease surveillance. Like we have the measles, the Lassa, and thing. I think we should include COVID because COVID maybe all around. Like we included Ebola, there should be a document on COVID-19 that will form part of our regular surveillance." (LIB county decision maker 024)*<br>*""…there's some value in looking at the things that we were looking at before COVID, because at least we have some longitudinal data on that so that we can see what the effect of COVID is." (LIV hospital health worker, Merseyside UK 020)*
**Principle 8: Cultivate effective partnerships and networks**	Liberia was able to call upon prior decision-making structures (established during Ebola response) to enable swift decisions.<br>Need for stronger engagement between primary and secondary care in Merseyside.	*"Involvement of multi-sectorial stakeholders in the response; that was one major thing that we learned from Ebola. And that has been brought to be on this response, so there has been a spark from the level of the presidency where they have key ministries and agency heads heading pillars on the COVID-19 response, involving the community people." (LIB national decision maker 028)*<br>*"I think one thing, it's really highlighted is the divide between hospital and primary care. We didn't work together very well before the epidemic, and we are still not working together very well. And I think if things were to get better, the whole health system needs to work better." (LIV community-level health worker, Merseyside UK 033)*
**Principle 9: Structures and mechanisms for advanced preparedness**	Learning from Ebola prompted rapid preparedness in Liberia, in contrast to Merseyside.	*"If you don't prepare well and you are caught unaware you will have a lot of issues, so we didn't wait for COVID to enter Liberia before we prepositioned basic PPE and those are all part of the preparedness phase." (LIB county decision maker 026)*<br>*""It was blatantly obvious that anything we've ever planned for in relation to a pandemic or anything along those lines was not the plans that we needed… So I think going forward there needs to be almost a better planning system in place…it's not*

For peer review only - http://bmjopen.bmj.com/site/about/guidelines.xhtml

Principle	Comparison	Quotations
		just a matter of just saying any pandemic it's about what kind of pandemic." (LIV hospital decision maker, Merseyside UK 069)
**Principle 10: Adapt governance and leadership structures to facilitate timely decision making and effective coordination of response**	Need for rapid guidance from national level to enable sub-national decision making was common in both settings.	*"So, at this point in time we think if you give the resources, put the money in the hands of the county health team to buy what they need, that will be more effective … So, we want decision should be given back to the people on the frontline so that they make the decision rather than a centralized point in Monrovia where people sit and decide for people in the lower level and the people choices made the right kind of thing they might need at that level."* (LIB national decision maker 028) *"… we were having to work, to a large extent, in the dark. The amount of guidance that came through nationally and even regionally, was actually relatively limited at that stage and we were having to do what felt like quite a lot of planning in isolation."* (LIV decision maker Merseyside UK 008)

Principle 1 Develop flexible pathways for medical supplies: Across both settings supply chains were disturbed due to global shortages and price inflation. In Merseyside there was a lack of personal protective equipment (PPE) and laboratory reagents needed for COVID-19 testing. Meanwhile, in Liberia, the disturbances related to routine supplies as supply chains shifted to focus on COVID-19 related procurement. In both settings, these challenges were felt to relate to global shortages, but were worsened by failure to maintain buffer stocks at local and national levels. In both settings, participants expressed the need for greater decentralisation of procurement decisions.

Principle 2 Prioritise a list of essential health services [*and continued provision of quality and equitable routine services*]: Participants from Merseyside expressed fears that there was too much emphasis on COVID-19 care, at times creating redundant capacity, while limiting access and quality of routine essential services. The blanket discontinuation of all elective non-urgent care at the height of the first wave in Merseyside, UK was felt to be unhelpful, and a more nuanced approach which seeks to balance long-term as well as short term risks associated with health conditions was recommended. In contrast, Liberia's early emphasis on routine health services was described as a key learning prioritised by decision-making platforms following the country's experience with the EVD epidemic.

For peer review only - http://bmjopen.bmj.com/site/about/guidelines.xhtml

COVID-19 adaptations in the UK led to increased telemedicine, with some respondents raising access-related equity concerns, particularly for elderly populations, who may struggle to engage with telemedicine. There were also concerns raised about quality of care, with some participants in Merseyside fearing delayed-diagnosis, misdiagnosis or sub-optimal care due to restrictions limiting physical contact with patients. In Liberia, limited opportunities for supervision, diversion of funds and staff for routine services towards COVID-19 response, and limited community outreach activities (due to physical distancing) were felt to impact quality of care. Across both settings innovations in service delivery have emerged (see policy briefs for details).[29,50]

Principle 3 Build trust with local communities: In both settings, community trust to seek health services declined, which reduced utilisation of services. In Liberia, fear among the population during the start of the pandemic led to reduction in the uptake of health services including national routine vaccination programmes and health facility-based delivery. This was felt to relate to a combination of fear of contracting COVID-19 at facilities and to reduced community outreach activities. Innovative community engagement and social mobilization strategies were introduced, for example follow-up visits to pregnant women, which led to patients returning to use services after a few months. Another example is the selective outreach home visits by the Neglected Tropical Disease (NTD) programme to NTD affected patients, in order to avoid interruption in treatment provision. In Merseyside, utilisation of non-COVID related services remained supressed for much longer. This was deemed to relate to widespread community mistrust, and Government campaigns which initially discouraged the public from visiting health facilities via the national 'Stay at home' messaging. Applying learning from Liberia's experience with EVD, the Government of Liberia placed a strong emphasis on working alongside community governance structures, involving local authorities as part of COVID-19 response.

Principle 4 Foster good communication at all system levels: The need for effective communication within the health system appeared to be a significant theme, particularly within findings from Merseyside. The rapidly changing context during the early months of the pandemic created a wealth

For peer review only - http://bmjopen.bmj.com/site/about/guidelines.xhtml

of daily new information. Virtual forms of communication rapidly expanded in both settings, with WhatsApp and online meeting platforms used extensively. Within Merseyside, referred to challenges such as multiple sources of guidance and communication channels struggling to keep pace with the changing guidance, which at times created contradictory messaging and confusion among health workers. By contrast, Liberia developed a centralised messaging procedure with approval needed from the department of Health Promotion before dissemination. In Merseyside, use of emails were typically less popular with staff as these could often be too long and wordy. Participants expressed limited scope for frontline staff to feedback on the information that had been shared.

Principle 5 Support, recognise and encourage staff: Staff redeployment was common across both settings, contributing to varied workloads. In Liberia, health worker redeployment to COVID-19 treatment centres, alongside largely unchanged utilisation rates contributed to increased workload for remaining health workers responsible for provision of routine services. By contrast in Merseyside, redeployment resulted in over-staffing in certain COVID-19 wards. Although there was disparity between health workers, with nurses experiencing increased workload. Due to the reduced volume of patients seeking routine care in the UK, workload was variable for those providing these services. The degree to which health workers received training about COVID-19 prior to having to manage COVID-19 patients varied between settings, with Liberia carrying out training in identification, isolation and infection, prevention and control before the first case of COVID-19 arrived in country, as a result of lessons learned following experiences responding to EVD. By contrast in Merseyside, the roll out of training varied widely by cadre, with some participants identifying that health care assistants and cleaning staff did not receive PPE training until later in the pandemic, compared with doctors and nurses (see table 2).

Anticipated mental health implications for health workers emerged from the Merseyside data, due to high rates of COVID-19 infection, exhaustion and high future anticipated post-traumatic stress disorder (PTSD). This was associated with fear of making treatment mistakes, stress surrounding

For peer review only - http://bmjopen.bmj.com/site/about/guidelines.xhtml

patient escalation decision making, anxiety over potential COVID-19 infection (both personal and for family), trauma surrounding high COVID-19 infections and deaths and reduced psychosocial support due to remote working. Measures to support staff wellbeing were introduced (including counselling, reflective therapy, peer support and mentoring, information made available about local support services), with varied levels of uptake. This was not widely discussed in Liberia. Although measures in Liberia to support staff wellbeing include psychosocial teams, roaming mental health counsellors providing services to health workers are in place. In Merseyside, community support, strong solidarity and teamwork were considered enablers of staff resilience.

Principle 6 Facilitate rapid resource flow and greater flexibility in its use: Historic underfunding of the health system in both settings has been highlighted by the pandemic. In Merseyside, this was considered to be due to nearly a decade of austerity, which has created weariness and uncertainty; whereas in Liberia it related to perception of reliance on external donors which predated the pandemic. Our findings confirmed the need for adequate funding to ensure the building blocks of the health system have received investment prior to the onset of any shock. With the arrival of the pandemic the availability and flexibility of funding differed between settings. In Merseyside, UK there was increased central government funding, which was mostly freed of usual bureaucratic checks. Managers noted that the removal of these bottlenecks allowed for swift action and rapid adoption of innovations. Frontline managers' ability to make operational decisions was viewed as central to resilience. In Liberia, however, there was an identified need for greater Government of Liberia ownership. Some sectors of the health system, particularly those which are donor reliant struggled in response to reduced partner support following the pandemic. Initially funding was not made available, however funds for routine service delivery were re-allocated to COVID-19 response, with implications for quality (see principle 2). Participants complained about excessive bureaucracy associated with use of funds, which created delays.

For peer review only - http://bmjopen.bmj.com/site/about/guidelines.xhtml

Principle 7 Ensure agile tracking of health information: Health information systems (HIS) were rapidly developed in the UK to collect huge quantities of surveillance data on COVID-19 and essential services. However, there was need for improved skills to usefully interpret this data. Respondents in Liberia stated that regular and timely submission of data, particularly from the community level had declined since the onset of COVID-19. This was considered to relate to reduced data validation, with decreased supervision visits due to physical distancing. In Merseyside complex new systems were designed to collect pandemic surveillance data, however, data was frequently not analysed or made readily accessible to staff to influence timely monitoring and quality improvement in services. In Merseyside, respondents also noted that a number of new initiatives were introduced during the pandemic, such as virtual consultations, but have not yet been systematically evaluated.

Principle 8 Cultivate effective partnerships and networks: The need for well-established partnerships emerged in both settings, with Liberia already having clear multi-sectoral participation in decision-making following the Incident Management System developed following EVD. Merseyside data highlighted pre-existing weaknesses in collaboration between primary and secondary/ tertiary care have been exacerbated. In both settings the need for greater engagement with the private sector was affirmed, with respondents from UK highlighting the need for stronger links regarding PPE supply chain shortages and in Liberia the need to strengthen collaboration given perceived weakness in private facility IPC standards. Partnerships were established within Merseyside, in a range of aspects of service delivery, including: regional network of laboratory providers to address equipment challenges and ensure COVID testing; between GPs to create service hubs; between disciplines and departments within hospital to address staff shortages and share information. In Liberia, a reduction in the number of partners providing response support was noted. This was a marked contrast to the EVD response.

Principle 9 Structures and mechanisms for advanced preparedness (newly identified principle from our findings): Within Liberia in particular, but also in Merseyside, there was discussion about

For peer review only - http://bmjopen.bmj.com/site/about/guidelines.xhtml

387 advanced preparedness. Respondents in Liberia emphasised how their experiences with previous shocks, particularly EVD, had facilitated learning around early recognition of the need for preparedness. For instance, there was consensus among respondents that waiting for COVID-19 to reach Liberia before responding would be too late. There was early rapid mobilisation of existing emergency response systems which had been established during the EVD response including; health check controls and quarantines at border points from January 2020; health worker COVID-19 training before the first confirmed case; enhanced hygiene practices; restriction of physical contact and sustained use of PPE, building on institutional memory gained through the EVD epidemic. In contrast, respondents in Merseyside expressed that the COVID-19 response was impeded by a lack of pandemic preparedness for new emerging infectious diseases.

397 <u>Principle 10 Adapt governance and leadership structures to facilitate timely decision-making and effective coordination of response</u> (newly identified principle from our findings): Being able to adapt governance and leadership structures to facilitate timely response coordination emerged from both settings. Liberia had previously established the incident management system (IMS) in 2014 as part of the response to EVD. It was re-activated in March 2020 to guide planning their pandemic response, led by the Minister of Health. This multi-sectoral team included a range of political and public health decision-makers, donors and partner representatives. At the time the study was carried out, most decisions were made centrally, with implementation at county level. In Merseyside, early response was hindered by slow and centralised guidance and decision-making, which was perceived to be oriented towards achieving political goals, rather than providing much needed clarity and recognition of local reality. The limited scope for local autonomy was considered to strain relationships between local senior leadership who sought to enforce central directives, and frontline staff, who wanted scope to influence them. In both settings, there was interest in greater de-centralisation of decision-making to lower levels.

411 **Discussion**

For peer review only - http://bmjopen.bmj.com/site/about/guidelines.xhtml

Our findings demonstrate the commonalities between the principles for resilience and people-centred health systems (Figure 2). We believe that maintaining a people-centred approach can help ensure that COVID-19 related adaptations are acceptable, understood and meet the needs of individuals (both patients and health workers). The values which underpin people-centred health systems emphasise the need for equity, orienting health services towards a health system which puts "people and communities at their centre, and surrounds them with responsive services that are coordinated both within and beyond the health sector, irrespectively of country setting and development status."(page 9 [14])

Adapting a people-centred framework

All ten FCDO principles (eight original principles and two principles identified through this study) are mapped against the original conceptual framework, to demonstrate the connection between our findings and existing literature about resilience (Figure 2) and recommendations in response to each principle are outlined in box 2.

Figure 2 placed here

For peer review only - http://bmjopen.bmj.com/site/about/guidelines.xhtml

Capacity and knowledge exchange

The continuation of routine essential service delivery following a shock to the health system, has previously been highlighted as an area of concern across a range of sectors.[51,52] Health systems

Box 2 Recommendations from expanded FCDO principles for resilience

1. Supply chains should pre-position adequate stocks, diversify sources and seek decentralisation of procurement. Collaboration between providers can prove valuable in securing continuity of supplies.
2. Routine services should be prioritised with a view to long term as well as short term impact, with prioritisation re-evaluated regularly as the pandemic progresses.
3. Maintain consistent communication and engagement with community leaders as partners to participate in pandemic planning within their respective communities.
4. Keep communication channels open, with regular updates for staff which highlight the key information, preferably through meetings, rather than email.
5. Ensure adequate provision of training, with sufficient PPE for health staff particularly for those staff at highest risk of COVID-19 infection, alongside measures to balance workload and promote staff wellbeing. Prioritise compassionate leadership which is supportive of staffing levels and rotas, along with staff mental wellbeing. Investment in psychosocial wellbeing throughout and after the pandemic response.
6. Health systems need to be adequately funded during 'normal times' if they are to be able to respond when a shock arises. There is urgent need for investment to clear the backlog of delayed routine services.
7. Health information systems need greater investment in both the systems and the human element to be able to analyse, interpret and respond to emerging data trends.
8. Opportunities for multisectoral collaboration should be sought out, with engagement with private sector where possible.
9. Develop a proactive approach, with advance plans for health shocks, along with escalation and de-escalation plans throughout the crisis.
10. Promote greater opportunities for de-centralised staff involvement in decision-making where feasible. Governments to prioritise an outward focus towards global solidarity.

need the capacity to continue to deliver services of good quality alongside responding to wider health challenges.[42] Our findings for principle 2 highlighted that COVID-19 adaptations in the UK led to the cancelling or postponing of many essential services, including those related to cancer care, which has been anticipated to decrease life expectancy and survival.[52,53] Meanwhile, Liberia emphasised the need for continuation of routine services and the promotion of patient confidence to use these services. This is in contrast to the EVD epidemic, where over 80% reductions in maternal delivery care

For peer review only - http://bmjopen.bmj.com/site/about/guidelines.xhtml

in EVD affected areas were described and form part of the reason why routine care was prioritised so strongly as part of the COVID-19 response.[54]

Our findings relating to supply chain (principle 1) resonate with literature from previous shocks and research emerging from the COVID-19 pandemic.[55,56] We found the need for greater flexibility, with engagement with a more diverse range of suppliers and greater decentralised control over supply chain across both settings. This is in keeping with a recent systematic review of supply chain resilience literature, which identified the importance of diversity and the social aspects of supply chains during a pandemic response.[55] Supplying commodities without investing in health systems strengthening will not produce a robust supply chain, limiting ability to respond quickly and effectively to future demands.[55]

We found a strong focus on the need for support for the health workforce, particularly in UK (principle 5). This was not as widely discussed in Liberia (though this may be a limitation relating to differing levels of participants between countries). However, a previous study in Sierra Leone and Liberia, highlighted that many providers may carry unresolved trauma from earlier shocks (including the Ebola epidemic), which may have implications for them during the COVID-19 response.[57,58] Research among health workers treating patients with COVID-19 in China, revealed health workers had a higher prevalence of insomnia, anxiety, depression, somatisation and obsessive-compulsive symptoms compared with nonmedical health workers, indicating the need for support and recovery programs for these staff.[59] Stressors identified among workers in China, include many of those described by participants in both settings within our study, particularly within Merseyside, including difficulties feeling safe at work, lack of infection prevention and control (IPC) measures and COVID-19 knowledge, long term workload, high risk of exposure to COVID-19, shortage of PPE and lack of rest, among others.[59]

Our findings regarding resource flow to frontline providers (Principle 6), are in keeping with previous study which identified funding as a core dimension within a health systems' ability to adapt and

For peer review only - http://bmjopen.bmj.com/site/about/guidelines.xhtml

For peer review only

respond to shocks.[60] A recent systematic review found aggregate public spending for health is associated with improved life expectancy, reduced child and infant mortality and more equitable health outcomes.[56]

Relational and teamwork components

The relational components which exist are shaped by risk, trust, values, power, norms, and culture.[42] These components play a role in determining the success (or failure) in response to a health systems shock or crisis. In contrast to the FCDO recommendation for good communication between actors (principle 4), our findings highlight challenges, particularly in the UK, where communication channels struggled to keep pace with changing guidance creating contradictory messaging and confusion among health workers. This is in keeping with previous study which found differences in lines of authority and acceptability of communication pathways can contribute to problems in communication.[34] In response, key principles were identified including participation for all, respect, information sharing, collaboration and problem-solving.[34]

The need for strong governance structures and leadership which adapts to the response (principle 10), was identified as a gap within early response in Merseyside. This was felt to have been hindered by slow and centralised guidance and decision-making with a perceived limited scope for autonomy within decision-making at lower levels. Within Liberia learning from the EVD response, and establishing an incident management system (IMS) (led by the Minister of Health) and Special Presidential Advisory Committee on Coronavirus (SPACC) (led by the President) early in planning their pandemic response enabled timely decision-making.[27] In both settings, there was interest in greater de-centralisation of decision-making to lower levels. Blanchet et al (2017) emphasised the need for legitimacy within resilience, with requirement of capacity to develop socially and contextually accepted institutions and norms.[40]

Looking more broadly, the conceptual framework highlights community engagement, with the community being active participants of any health systems response (principle 3).[39] Our findings

For peer review only - http://bmjopen.bmj.com/site/about/guidelines.xhtml

emphasise the value of community engagement within the response within Liberia, based on lessons from the EVD pandemic and in keeping with WHO recommendation that this be a key pillar within COVID-19 country response.[8] Liberians across all socio-demographic groups responding to a recent survey said they were very well, or somewhat well informed about the COVID-19 pandemic, with only 5% feeling not very well/ not at all informed.[27] This also emerged as a key finding in Singapore, with engagement through new and social media channels monitored, with clarification of misinformation by MOH.[61] In contrast to the findings from Liberia, participants from Merseyside highlighted the need for stronger communication (although there were some examples of creative ways to engage with diverse communities).

Learning from our study has emphasised the need to better prepare for, and respond to, health emergency crises through integrated services (Principle 9).[44] A recent survey found most of the population felt the Liberian government was doing well in managing the pandemic.[44] This contrasted with findings from the UK where there was felt to have been a lack of adequate advance planning and preparation. Two previous literature reviews highlighted that “preparedness depends on health systems ability to learn from prior pandemics”, with responses often reactive rather than proactive.[56,62]

The people-centred approach stresses the need for awareness and recognition of the interdependencies of the health system with the community and other social systems, including education, social protection and food security and their relationship with social determinants of health (principle 8).[63] Our findings emphasise the need for strong partnerships with other sectors across settings, in keeping with an identified success in Singapore’s response,[61] and is a key aspect of Blanchet et al.’s resilience framework, ensuring the capacity to engage with and handle multiple actors and dynamics.[40]

Our findings, particularly from Merseyside emphasise the vast quantities of data being generated through the COVID-19 response, but there are gaps in how this data is analysed and utilised within the

For peer review only - http://bmjopen.bmj.com/site/about/guidelines.xhtml

511 health system. The importance of adequate HIS is in keeping with previous studies.[40,60] A health system's ability to identify and respond to an emerging threat is needed if it is to appropriately meet emerging needs during a rapidly evolving health crisis or shock (principle 7).[40,41] A robust health management information system (HMIS) is crucial to a health systems capacity to respond to shock.[60] Health systems need to have the ability to combine and integrate different forms of knowledge and to anticipate and cope with uncertainties and unplanned events.[40]

517 COVID-19 has reflected and exacerbated existing social inequalities and emphasised the importance of global collective action, rather than an individual response for genuine resilience. [8] Vaccine inequity and a lack of global solidarity on the part of some richer countries, are dominating the current phase of the pandemic. Our findings seek to highlight opportunity for shared learning across settings in the Global South and North, emphasising the need for a global response to this and future shocks.

522 **Strengths and Limitations**

523 The strengths of this study include the quality of data analysis, which involved a wide range of researchers across both settings, and the breadth of perspectives captured from frontline staff and key decision-makers early in the course of the pandemic. Our study had a number of limitations. Within Merseyside, study participants were selected from across a range of health system levels including primary care, hospital frontline workers and decision-makers as well as regional decision-makers. By contrast, in Liberia participants included national and county level decision-makers, technicians and supervisors of frontline staff, with no direct frontline workers included. This may result in some of the differences in findings, related to these differing perspectives. Perhaps the greatest limitation of this study is that it was carried out at a single point in time. In Merseyside we collected data towards the end of the first wave, at a time when there were few inpatients and people were reflecting on the first wave. Meanwhile in Liberia it was carried out before there had been a large increase in cases. Since the study was carried out there have been subsequent even greater waves of cases within Merseyside, UK and Liberia has experienced a large surge in cases of the delta

For peer review only - http://bmjopen.bmj.com/site/about/guidelines.xhtml

variant (59% of cases recorded in Liberia up until 17th July 2021, occurred during a six week period from June 1 2021 to 17th July 2021).[64] By the weeks beginning July 24th to August 7th 2021 number of confirmed cases had declined between zero to 43. Response measures have evolved in both settings, and limitations identified through the study may have been addressed in subsequent stages of the pandemic.

Conclusion

We found the ability of health systems to be able to absorb, adapt and transform in response to the COVID-19 pandemic in two very different settings closely relates to the eight FCDO principles of resilience.[16,40] We expanded these principles to include strong structures and mechanisms for advance preparation, and adaptable governance and leadership structures to facilitate timely decision-making and response coordination. At the heart of our findings lies the centrality of the people-centred health system, where the person, is placed within their family, community and the health system.[14] When all aspects work together the outcome is the extent of resilience demonstrated within a health system in response to shock.[40] This includes both the provision of specific services in response to the shock experienced, as well as continued provision of and demand for 'routine care'. Our study highlights the need to maintain a people-centred approach for a resilient health system response.

For peer review only

Acknowledgement

We would like to thank all the participants who made time to share their experience and reflections to make this research possible. We recognise and thank Abiola Aiyenigba, who sadly passed away during the study, for her inputs. This research was funded by the NIHR Health Protection Research Unit in Emerging and Zoonotic Infections, and the Centre of Excellence in Infectious Diseases Research, and the Alder Hey Charity. We also acknowledge support of Liverpool Health Partners and the Liverpool-Malawi-Covid-19 Consortium. Additional funding to support this work came from the NIHR REDRESS Programme (NIHR2001129) and FCDO COUNTDOWN (PO6407) programme. We thank Tim

Martineau, and Joanna Raven (LSTM) for early inputs into study design and Susie Crossman for managing the study budget.

Competing Interests

None declared.

Author Statement

RM prepared the first draft of the paper with inputs from all; Study design, conceptualisation, ethics (ST, LD, MT, LF, IB, ZZ, RM, VW, HP, RAdC, RH, KK); conducted interviews in UK – RM, VW, MT, KO, HP, SC, ST, TEH, RH, RD, YD, OH; conducted interviews in Liberia - ZZ, WT, HB, JK, JSS, CP, GZ, RM. All interviewers participated in the cross-country analysis which was led by YA in the UK with inputs from those who conducted UK interviews and LD, RM, ZZ, HB, WT, JK, JSS, GZ, CP in Liberia. All authors were involved in critical review of the approach, inputted into and approved the final draft of the manuscript.

Figure 1 caption: Conceptual framework

Figure 2: Expanded principles for resilience and people-centred health systems framework

Funding

This research was funded by the NIHR Health Protection Research Unit in Emerging and Zoonotic Infections, and the Centre of Excellence in Infectious Diseases Research, and the Alder Hey Charity. We also acknowledge support of Liverpool Health Partners and the Liverpool-Malawi-Covid-19 Consortium. Additional funding to support this work came from the NIHR REDRESS Programme (NIHR2001129) and FCDO COUNTDOWN (PO6407) programme.

Data availability

Data are available upon reasonable request.

For peer review only - http://bmjopen.bmj.com/site/about/guidelines.xhtml

584 **References**

585 1. Meyer D, Bishai D, Ravi SJ, et al. A checklist to improve health system resilience to infectious
586 disease outbreaks and natural hazards. *BMJ Glob Heal*. 2020;5(8):1–7.

587 2. World Health Organization. Operational guidance for maintaining essential health services
588 during an outbreak [Internet]. 2020. Available from:
589 https://apps.who.int/iris/bitstream/handle/10665/331561/WHO-2019-nCoV-
590 essential_health_services-2020.1-eng.pdf?sequence=1&isAllowed=y

591 3. United Nations Economic Commission for Africa. COVID-19 in Africa: Protecting Lives and
592 Economies [Internet]. 2020. Available from:
593 https://repository.uneca.org/bitstream/handle/10855/43756/b11983206.pdf?sequence=1&i
594 sAllowed=y

595 4. Agyeman AA, Laar A, Ofori-Asenso R. Will COVID-19 be a litmus test for post-Ebola sub-
596 Saharan Africa? *J Med Virol*. 2020;92(9):1373–5.

597 5. Zar HJ, Dawa J, Bueno G, et al. Challenges of COVID-19 in children in low- and middle- income
598 countries. *Paediatr Respir Rev Rev*. 2020;(January).

599 6. Burki T. The indirect impact of COVID-19 on women. *Lancet Infect Dis* [Internet].
600 2020;20(8):904–5. Available from: http://dx.doi.org/10.1016/S1473-3099(20)30568-5

601 7. Bayani DB, Krubiner C, Barasa E, et al. The Indirect Health Effects of COVID-19: Emerging
602 Findings from Kenya, the Philippines, South Africa, and Uganda. *Cent Glob Dev*. (July 2021).

603 8. D'Ambruoso L, Abbott P, Binagwaho A. Building back fairer in public health policy requires
604 collective action with and for the most vulnerable in society. *BMJ Glob Heal*. 2021;6(3):8–11.

605 9. Dzinamarira T, Dzobo M, Chitungo I. COVID-19: A perspective on Africa's capacity and
606 response. *J Med Virol*. 2020;92(11):2465–72.

For peer review only - http://bmjopen.bmj.com/site/about/guidelines.xhtml

10. Das Gupta M, Mansuri G, Sinha N, et al. Overcoming gender-based constraints to utilization of maternal and child health services in Pakistan: the role of the doorstep delivery system. TT - [Internet]. Washington D.C.: [Unpublished] 2007. Presented at the 2007 Annual Meeting of the Population Association of America, New York, New York, March 29-31, 2007.; 2007. Available from: http://paa2007.princeton.edu/download.aspx?submissionId=71659

11. JHU. John Hopkins Coronavirus Resource Center [Internet]. Johns Hopkins University & Medicine. 2021 [cited 2021 Jun 7]. Available from: https://coronavirus.jhu.edu/

12. FPA Reporter. Former Center for Disease Control Boss Hails Liberia for COVID-19 Prevention [Internet]. Front Page Africa. [cited 2021 Jun 7]. Available from: https://frontpageafricaonline.com/news/former-center-for-disease-control-boss-hails-liberia-for-covid-19-prevention/

13. Frieden T. Which Countries Have Responded Best to Covid-19? Wall Street Journal [Internet]. 2021 Jan 1; Available from: https://www.wsj.com/articles/which-countries-have-responded-best-to-covid-19-11609516800

14. World Health Organisation. WHO Global Strategy on people-centred and integrated health services, Interim Report [Internet]. Geneva: World Health Organisation; 2015. Available from: https://apps.who.int/iris/bitstream/handle/10665/155002/WHO_HIS_SDS_2015.6_eng.pdf;jsessionid=B2F4B22FC042ACD2BEF20C5F34803E56?sequence=1

15. Saulnier DD, Blanchet K, Canila C, et al. A health systems resilience research agenda: moving from concept to practice. *BMJ Glob Heal*. 2021;6(8):e006779.

16. Department for International Development [DFID]. Principles of Health Systems Resilience in the Context of Covid-19 Response [Internet]. 2020. Available from: https://assets.publishing.service.gov.uk/media/5ea2b33de90e070498c5538c/Principles_of_Health_Systems_Resilience_in_the_Context_of_COVID_Research_Brief_April_2020.pdf

For peer review only

For peer review only - http://bmjopen.bmj.com/site/about/guidelines.xhtml

17. Van Damme W, Dahake R, Delamou A, et al. The COVID-19 pandemic: Diverse contexts; Different epidemics - How and why? *BMJ Glob Heal*. 2020;5(7):1–16.

18. The World Bank. Liberia poverty headcount ratio [Internet]. 2016. Available from: https://data.worldbank.org/indicator/SI.POV.DDAY?locations=LR

19. UNDP. Human Development Report 2020: The Next Frontier Human Development and the Anthropocene [Internet]. UNDP: New York, NY, USA. 2020. Available from: http://hdr.undp.org/sites/all/themes/hdr_theme/country-notes/LBR.pdf

20. Ling EJ, Larson E, Macauley RJ, et al. Beyond the crisis : did the Ebola epidemic improve resilience of Liberia ' s health system? *Health Policy Plan*. 2017;32:40–7.

21. Ministry of Health and Social Welfare (MoHSW) L. Liberia National Community Health Services Policy.

22. Ministry of Health Liberia. Investment plan for building a resilient health system in Liberia [Internet]. Vol. 12, Monrovia, Ministry of Health. Monrovia, Liberia; 2015. Available from: https://www.resilientinstitutionsafrica.org/sites/default/files/2018-09/%5BLIBERIA%5D Investment Plan for Building a Resilient Health System %282015-2021%29.pdf

23. Liverpool City Council. Liverpool City Council: public health transformation six years on [Internet]. 2019. Available from: https://www.local.gov.uk/liverpool-city-council-public-health-transformation-six-years

24. Statista. Number of coronavirus (COVID-19) cases in the United Kingdom (UK) as of April 5, 2021, by country/region [Internet]. 2020 [cited 2021 Jun 7]. Available from: https://www.statista.com/statistics/1102151/coronavirus-cases-by-region-in-the-uk/

25. Mayor of Liverpool. A STRONG AND GROWING CITY BUILT ON FAIRNESS. 2018.

26. University of Liverpool, Public Health England, Joint Biosecurity Centre, et al. Liverpool Covid-

For peer review only - http://bmjopen.bmj.com/site/about/guidelines.xhtml

SMART Community Testing Pilot [Internet]. Liverpool; 2021. Available from: https://www.liverpool.ac.uk/media/livacuk/research/Mass,testing,evaluation.pdf

27. Sanny JA-N, Bloh O. Liberians laud government ’ s COVID -19 response but claim unfair distribution of relief assistance. *Afrobarometer*. 2021;(420):1–16.

28. Kateh F. How Ebola helped Liberia prepare for covid [Internet]. MIT Technology Review. 2020. Available from: https://www.technologyreview.com/2020/08/19/1007139/liberia-kateh-covid-coronavirus-ebola/

29. Parker C, Zawolo G, Kollie J, et al. PRINCIPLES FOR PROMOTING RESILIENT HEALTH SYSTEMS COVID-19 RESPONSE : LEARNING FROM LIBERIA Principle One : Develop flexible. 2020;

30. Stevens S, Pritchard A. Letter to Chief Executives of all NHS trusts and foundation trusts regarding second phase of NHS response to COVID19 [Internet]. NHS; 2020 p. 1–9. Available from: https://www.england.nhs.uk/coronavirus/wp-content/uploads/sites/52/2020/04/second-phase-of-nhs-response-to-covid-19-letter-to-chief-execs-29-april-2020.pdf

31. Rosenbaum L. Medicine a nd Society The Untold Toll — The Pandemic ’ s Effects on Patients without Covid-19. *N Engl J Med*. 2020;1–4.

32. Pope C, Mays N. Qualitative Research: Reaching the parts other methods cannot reach: an introduction to qualitative methods in health and health services research. *BMJ*. 1995 Jul 1;311:42–5.

33. Kuper A, Reeves S, Levinson W. An introduction to reading and appraising qualitative research. *BMJ Br Med J*. 2008;337:404–9.

34. Gilson L, Barasa E, Nxumalo N, et al. Everyday resilience in district health systems: emerging insights from the front lines in Kenya and South Africa. *BMJ Glob Heal* [Internet]. 2017;2(2):e000224. Available from: http://gh.bmj.com/lookup/doi/10.1136/bmjgh-2016-

For peer review only - http://bmjopen.bmj.com/site/about/guidelines.xhtml

000224

35. Biddle L, Wahedi K, Bozorgmehr K. Health system resilience: a literature review of empirical research. *Health Policy Plan*. 2020;1–26.

36. Sheikh K, George A, Gilson L. People-centred science: strengthening the practice of health policy and systems research. *Heal Res Policy Syst*. 2014;12(1):19.

37. Sheikh K, Ranson MK, Gilson L. Explorations on people centredness in health systems. *Health Policy Plan*. 2014;29(Suppl 2):ii1.

38. Gilson L, Barasa E, Nxumalo N, et al. Everyday resilience in district health systems: Emerging insights from the front lines in Kenya and South Africa. *BMJ Glob Heal*. 2017;2(2):1–15.

39. Newton-lewis T, Witter S, Fortnam M, et al. Working Paper : What is a Shock-Responsive Health System ? *Maint Res Support Soc Serv to Adapt to Shock*. 2020;(June):1–11.

40. Blanchet K, Nam SL, Ramalingam B, et al. Governance and capacity to manage resilience of health systems: Towards a new conceptual framework. *Int J Heal Policy Manag* [Internet]. 2017;6(8):431–5. Available from: http://dx.doi.org/10.15171/ijhpm.2017.36

41. Kruk ME, Ling EJ, Bitton A, et al. Building resilient health systems: A proposal for a resilience index. *BMJ* [Internet]. 2017;357(May):1–8. Available from: http://dx.doi.org/doi:10.1136/bmj.j2323

42. Barasa EW, Cloete K, Gilson L. From bouncing back, to nurturing emergence: Reframing the concept of resilience in health systems strengthening. *Health Policy Plan*. 2017;32(1):iii91–4.

43. Starfield B. Is patient-centered care the same as person-focused care? *Perm J*. 2011;15(2):63.

44. World Health Organization (WHO). The WHO global strategy on people- centred integrated health services services delivery and safety department [Internet]. Service Delivery and Safety. 2015. Available from:

For peer review only

For peer review only - http://bmjopen.bmj.com/site/about/guidelines.xhtml

https://apps.who.int/iris/bitstream/handle/10665/155002/WHO_HIS_SDS_2015.6_eng.pdf?sequence=1&isAllowed=y

45. Saunders B, Sim J, Kingstone T, et al. Saturation in qualitative research: exploring its conceptualization and operationalization. *Qual Quant*. 2018;52(4):1893–907.

46. Fereday J, Muir-Cochrane E. Demonstrating Rigor Using Thematic Analysis: A Hybrid Approach of Inductive and Deductive Coding and Theme Development. *Int J Qual Methods*. 2006;5(1):80–92.

47. Roberts K, Dowell A, Nie JB. Attempting rigour and replicability in thematic analysis of qualitative research data; A case study of codebook development. *BMC Med Res Methodol*. 2019;19(1):1–8.

48. Ritchie J, Lewis J, Nicholls CM, et al. The Foundation of Qualitative Research. *Qual Res Pract A Guid Soc Sci Students Res*. 2013;0–25.

49. Gale NK, Heath G, Cameron E, et al. Using the framework method for the analysis of qualitative data in multi-disciplinary health research. *BMC Med Res Methodol* [Internet]. 2013 Jan [cited 2014 May 1];13(1):117. Available from: http://www.pubmedcentral.nih.gov/articlerender.fcgi?artid=3848812&tool=pmcentrez&rendertype=abstract

50. Alhassan Y, McCollum R, Watson V, et al. PRINCIPLES FOR PROMOTING RESILIENT HEALTH SYSTEMS COVID-19 RESPONSE : [Internet]. 2021 [cited 2021 Jun 7]. Available from: https://www.redressliberia.org/wp-content/uploads/2021/06/Merseyside-Policy-Brief.pdf

51. Akseer N, Kandru G, Keats EC, et al. COVID-19 pandemic and mitigation strategies: Implications for maternal and child health and nutrition. *Am J Clin Nutr*. 2020;112(2):251–6.

52. Rajan S, Chakrabarti D, Akhtar N, et al. Searching for answers: Cancer care during the COVID pandemic. *Clin Epidemiol Glob Heal*. 2021;10(January).

For peer review only

For peer review only - http://bmjopen.bmj.com/site/about/guidelines.xhtml

53. Thorlby R, Fraser C, Gardner T. Non-COVID-19 NHS Care During the Pandemic [Internet]. The Health Foundation. 2020. Available from: https://www.health.org.uk/news-and-comment/charts-and-infographics/non-covid-19-nhs-care-during-the-pandemic

54. Elston JWT, Cartwright C, Ndumbi P, et al. The health impact of the 2014 - 15 Ebola outbreak. *Public Health* [Internet]. 2016;143:60–70. Available from: http://dx.doi.org/10.1016/j.puhe.2016.10.020

55. Golan MS, Jernegan LH, Linkov I. Trends and applications of resilience analytics in supply chain modeling: systematic literature review in the context of the COVID-19 pandemic. *Environ Syst Decis* [Internet]. 2020;40(2):222–43. Available from: https://doi.org/10.1007/s10669-020-09777-w

56. Witter S, Palmer N, Balabanova D, et al. Evidence review of what works for health system strenthening, where and when? 2021;(February):99. Available from: https://rebuildconsortium.com/resources/research-reports/evidence-review-of-what-works-for-health-systems-strengthening-where-and-when/

57. Dean L, Cooper J, Wurie H, et al. Psychological resilience, fragility and the health workforce: Lessons on pandemic preparedness from Liberia and Sierra Leone. *BMJ Glob Heal*. 2020;5(9).

58. Tengbe S. World Mental Health Day 2020 one LSTM student's research on mental health amongst health workers in Sierra Leone [Internet]. Available from: https://www.lstmed.ac.uk/news-events/news/world-mental-health-day-2020-one-lstm-student's-research-on-mental-health-amongst

59. Zhang WR, Wang K, Yin L, et al. Mental Health and Psychosocial Problems of Medical Health Workers during the COVID-19 Epidemic in China. *Psychother Psychosom*. 2020;89(4):242–50.

60. Hanefeld J, Mayhew S, Legido-Quigley H, et al. Towards an understanding of resilience: Responding to health systems shocks. *Health Policy Plan*. 2018;33(3):355–67.

For peer review only - http://bmjopen.bmj.com/site/about/guidelines.xhtml

61. Chua AQ, Tan MMJ, Verma M, et al. Health system resilience in managing the COVID-19 pandemic: lessons from Singapore. *BMJ Glob Heal*. 2020;5(9):e003317.

62. Sharma A, Borah S, Moses A. Responses to COVID-19: The role of governance, healthcare infrastructure, and learning from past pandemics. *J Bus Res*. 2021;(January):597–607.

63. UNICEF. Children and AIDS: third stocktaking report. TT - [Internet]. New York, New York, UNICEF, 2008.; 2008. Available from: http://www.unicef.org/devpro/files/CnA_TSR_LoRes_PDF_EN_USLetter_11202008.pdf

64. Ministry of Health Liberia. Liberia COVID-19 Daily Sitrep 510, 7th August 2021. Monrovia, Liberia; 2021.

For peer review only

For peer review only - http://bmjopen.bmj.com/site/about/guidelines.xhtml

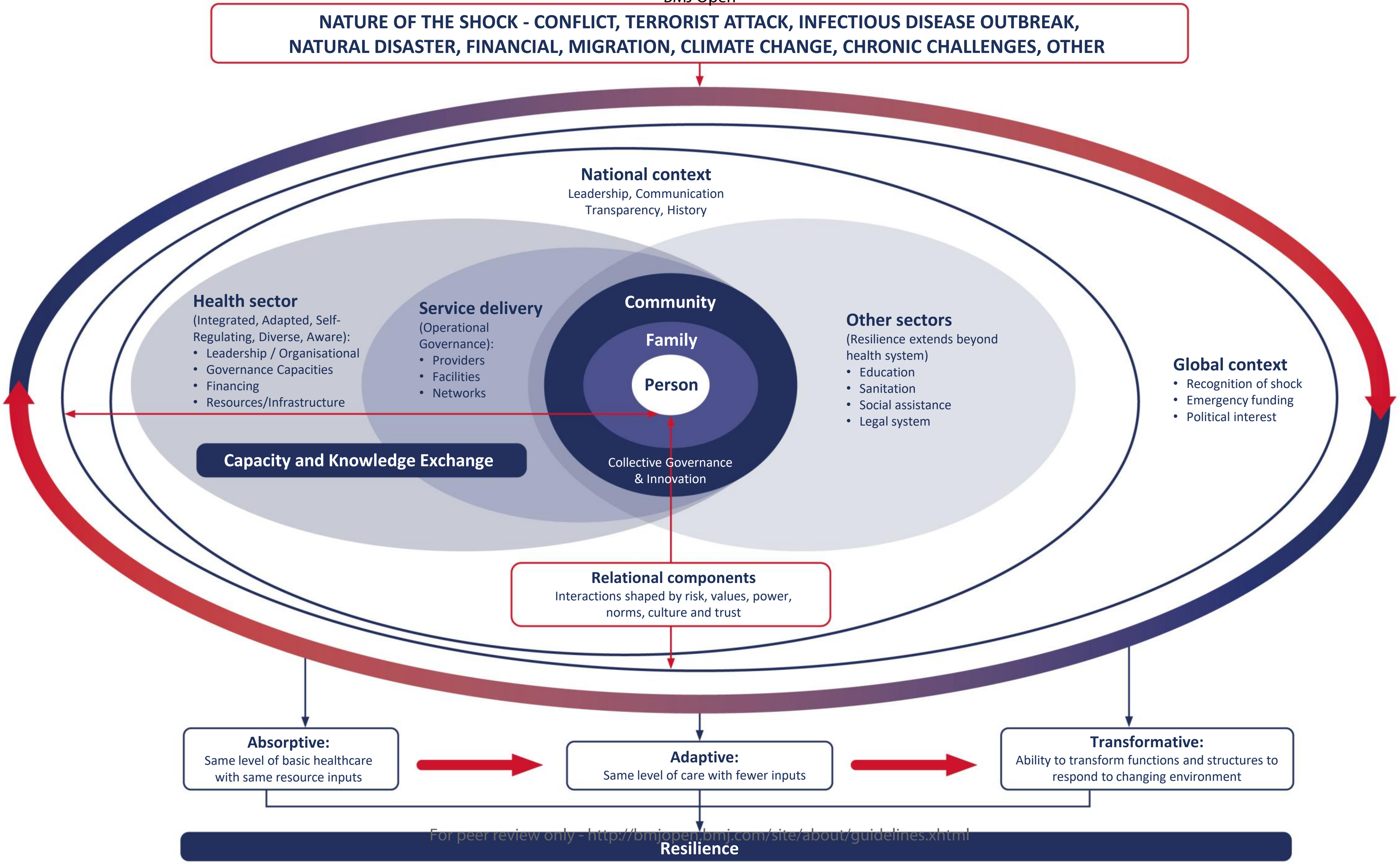

For peer review only - http://bmjopen.bmj.com/site/about/guidelines.xhtml

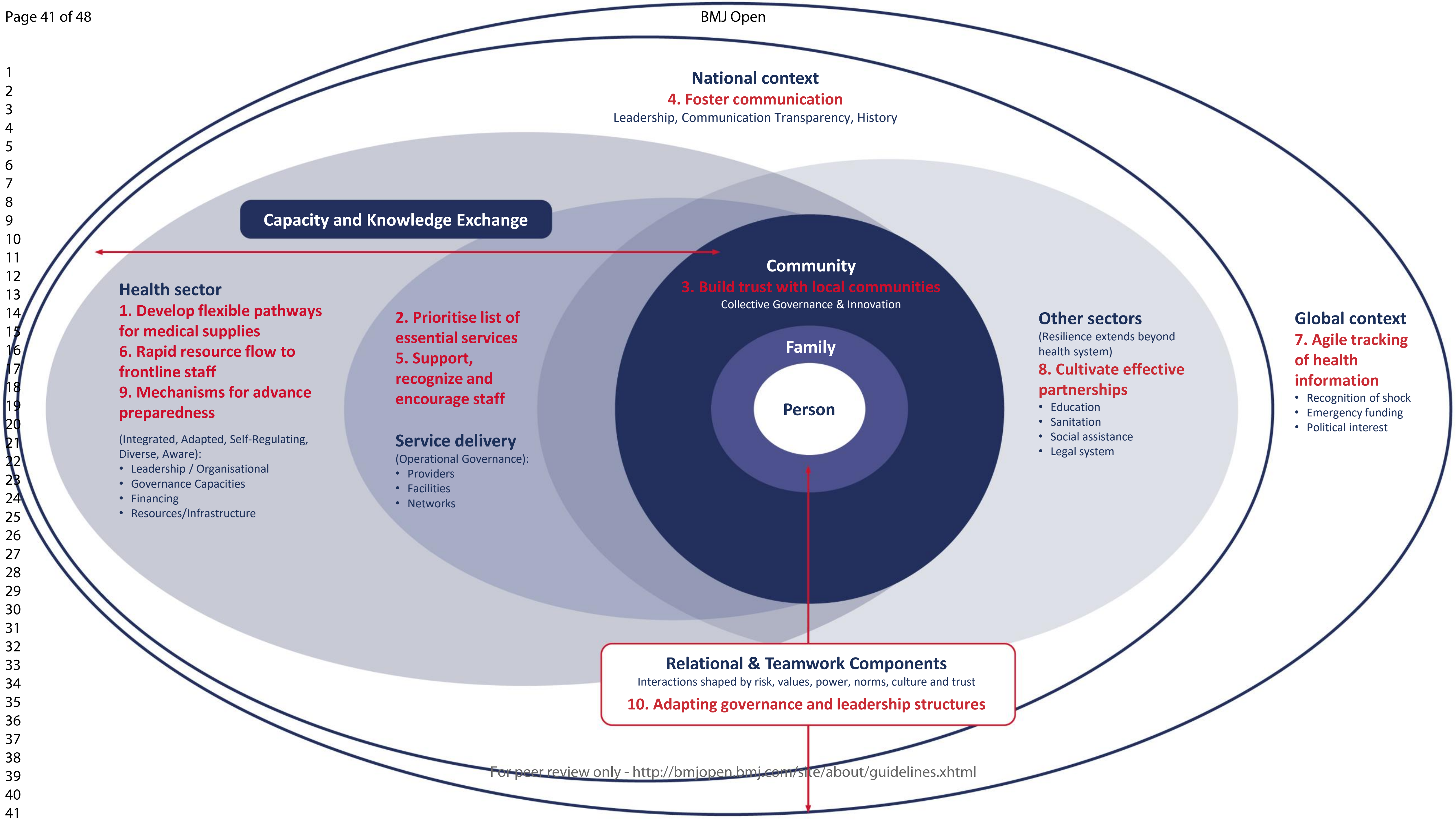

For peer review only - http://bmjopen.bmj.com/site/about/guidelines.xhtml

Appendix 1: COVID-19 Key Informant Interview Topic Guides

Contents

Appendix 1: COVID-19 Key Informant Interview Topic Guides 1

Key Informant Interviews Topic Guide –MOH Liberia 2

Key Informant Interviews Topic Guide –Merseyside Regional Decision Makers 4

Key Informant Interviews Topic Guide – Health Workers 6

Key Informant Interviews Topic Guide –Merseyside Laboratory and Blood Transfusion Staff 8

For peer review only

For peer review only - http://bmjopen.bmj.com/site/about/guidelines.xhtml

Key Informant Interviews Topic Guide –MOH Liberia

All possible questions to be asked of key informants are described in the following guide. Prior to interviewing each stakeholder, specific guides for these individuals will be made. One interview that covers relevant research themes will be completed with each stakeholder. Ordering of questions will also be revised to ensure logical flow through the interview and to avoid repetition.

Background

Please can you tell me your position and how long you have worked in your current role?
How has your role changed due to the current COVID-19 crisis?

Responses to Shock and the General Health System

1. How do you think the health system has coped with the COVID-19 crisis? How did it compare with previous crisis? How have routine services been impacted?
2. How is the current shock (COVID-19) the health system is experiencing similar or different to those you have experienced before?
3. What are the key learnings from previous shocks (Ebola/ conflict/ economic crisis)? How are they being used to respond now?
4. How do you think routine health systems functions are being impacted by the current crisis (COVID19/economic)?
5. What do you think could be done to support continuation of routine services? How is this informed or shaped by learnings from during the Ebola period?
    a. How would you describe the quality of services usually? How is quality of care being maintained throughout the COVID-19 response?

6. What policy or guidelines are supporting with the current COVID response? What additional guidelines or policies could be helpful for the COVID response?

Service Specific Impacts

Questions in this section to be reviewed/modified for cross-cutting MOH functions, e.g. M&E, research division prior to starting interview

7. Can you tell me about how service delivery within your programme/section (adapt to include name of section depending on who talking too) has been affected by the COVID pandemic?
    a. Which of your services would you say have been most impacted so far? Why?
    b. Which services would you envisage will be most impacted moving forwards? Why?
8. How have your routine services been modified or adapted? Which components of your service do you view as essential? Why?
9. Which specific sub-populations is routine care most impacted for? Are there any marginalised groups who may struggle to use services since the onset of the COVID-19 crisis? (Probe: e.g gender, dis/ability, rural/urban; wealth; geographic regions; age etc)
10. Have there been any innovations within service delivery in response to the COVID-19 crisis, and have they been useful in any way?
11. Has there been any innovations in response to COVID-19 that have concerned you?

Human Resource Management

12. How have you planned for staffing to meet the changing additional workload in response to COVID? Any tools/ guidance from the human resource section? Successes and challenges? (Prompt for role of new community health cadres, for those providing face to face care and for MOH staff)
13. What additional skill development have you provided and how in response to COVID? Successes and challenges?
14. How are you able to support staff so they can continue to work effectively during the COVID pandemic
    a. How have you supported staff through communication?
    b. How have you supported staff for occupational safety including PPE?

COVID-19 Key Informant Interview Topic Guides

c. How have you supported staff through with psychosocial support?
d. What have been the successes and challenges with supporting staff?

Service and System Impacts: Governance and Decision Making
Questions in this section to be reviewed/modified to make these questions more service-specific, depending on the interviewee's programme area

15. How are decisions made about which services should or should not be prioritised as part of the COVID response? (prompt for in relation to their specific service and also in relation to general health system, prompt for donor influence)
16. How does decision-making as part of the COVID response influence routine planning activities? What has been the impact of resource re-distribution as part of the COVID response?
17. Who is involved in this decision making and what are the processes? What are the challenges?

18. What do you think are the key ethical impacts of making these decisions? What ethical guidelines are currently in place and important in decision making during this period?
19. What guidance documents are available to support you in making decisions regarding COVID?
20. What guidance documents would help to support maintaining routine services?

Closing Questions

21. What does a resilient health system look like to you? What are your three recommendations would you make to improve or maintain the resilience of the Liberian health system during this period?
22. What are your three recommendations would you make post crisis to ensure the return to routine function of the health system as effectively as possible?

Additional questions for Director of personnel only

23. What are the main sources of additional staffing (e.g. secondment/redeployment, task-shifting, improved productivity, early graduation/students, returnees, volunteers)? Successes and challenges? Optional: Impact on the wage bill?
24. What areas of service are now struggling with staffing?
25. What are you able to do to retain staff? Successes and challenges?
26. What impact did/is down-sizing of "non-essential staff' have on your programme during the crisis?

Thank-you
Any other comments?

For peer review only - http://bmjopen.bmj.com/site/about/guidelines.xhtml

Key Informant Interviews Topic Guide –Merseyside Regional Decision Makers

Version1.1_01052020

All possible questions to be asked of key informants are described in the following guide. Prior to interviewing each stakeholder, specific guides for these individuals will be made. One interview that covers relevant research themes will be completed with each stakeholder. Ordering of questions will also be revised to ensure logical flow through the interview and to avoid repetition.

Question List

Background

Please can you tell me your position and how long you have worked in your current role?

Impact of COVID 19 on Routine Service Delivery

1. What are defined as essential routine services?
2. Which are the main scheduled and unscheduled services affected by COVID-19 and how have these been adapted over time?
3. Have there been any innovations within service delivery, and what have these been?
4. Have there been any changes that have concerned you? Why?
5. What would help to support maintaining routine services?

Governance and Decision Making

6. What has informed your decision-making, such as guidance documents or governance decision-making processes?
7. Who is involved in decisions made about which services should or should not be prioritised?
8. How are decisions made about which services should or should not be prioritised?
9. Describe how and who is involved in operationalising decisions?
10. What challenges have you faced in making these decisions?
11. What are the main differences between various sites in the trust, especially between Aintree and the Royal Hospitals?
12. How are changes in service delivery communicated? How can this be improved? There are multiple guidelines at national and local levels, how are these disseminated? How well does this work? How rapidly? How do health care workers respond to these changes?

Human Resource Management

13. How have you [may be the employer in general] planned for staffing to meet the changing additional workload? Any tools/ guidance from national authorities? Successes and challenges?
14. How have you planned for the increase in staff absence?
15. What additional skill development have you provided and how? What have been the successes and challenges?
16. How are you able to support staff so they can continue to work effectively (e.g. communication, occupational safety including PPE, psychosocial support)? What have been the successes and challenges?

Recovery post COVID-19

17. Are there any COVID-19-related changes to routine health services that you think it would be useful to continue after COVID-19? Which ones and why?

18. What next steps do you believe should be taken now to support the health system to recover post COVID-19?

For peer review only - http://bmjopen.bmj.com/site/about/guidelines.xhtml

Thank-you

Do you have any further suggestions for improvements to delivery of routine services?

Any other comments?

For peer review only

For peer review only - http://bmjopen.bmj.com/site/about/guidelines.xhtml

Key Informant Interviews Topic Guide – Health Workers

Version1.1_01052020

All possible questions to be asked of key informants are described in the following guide. Prior to interviewing each stakeholder, specific guides for these individuals will be made. One interview that covers relevant research themes will be completed with each stakeholder. Ordering of questions will also be revised to ensure logical flow through the interview and to avoid repetition.

Question List

Background

Please can you tell me your usual position and how long you have worked in that role?
Are you currently working in your usual role and department?
If no, what role and department are you now working in?

Impact of COVID 19 on Routine Essential Service Delivery

1. Can you tell me about how health service delivery has been affected by the COVID pandemic? What was the processes for this, how was it communicated and do you have any ideas about how this can be improved? How prepared did you feel for these?
2. What do you consider to be routine essential health services in your work?
3. Which are the main scheduled and unscheduled services affected by COVID-19 in your department and how have these been adapted over time?
4. What have been the strengths and challenges with these changes? How has quality been affected?
5. How should these changes be evaluated? What indicators should be used?
6. What is worrying you most about your service now?
7. Which services would you envisage will be most impacted moving forwards as the pandemic progresses? (e.g. hospital based, community care, disease specific services, etc) Why?
8. Who do you think are the people most impacted by the changes in routine service delivery? Would you say that patients with specific socio-demographic characteristics are more impacted by service disruption/ distortion than others? Why? (e.g. gender, dis/ability; rural/urban; wealth; geographic regions; age etc) What can be done to ensure that these patients can still use health services when they need them?

Ethics and Decision Making

9. Have you encountered any health systems issues which you found troubling since the start of the COVID-19 pandemic? Would you be willing to tell me more about these issues?
10. What is the impact of these issues on you as a health worker? What would be helpful to support you in dealing with these issues?
11. Do you know of any ethical guidelines in place to guide you as you make difficult decisions during this time? What are these? How are these ethical guidelines operationalised? Are they useful?
12. Have you been involved with making decisions about the changes to health services since the COVID-19 pandemic? What was your role in making these decisions? How were these decisions made?
13. When there are changes in how health services are delivered how are these communicated with you? How has this worked? What do you think is the best way to be informed?

Human Resource Management

14. How has your role changed since the start of the COVID-19 pandemic? What have been the successes and challenges with how your role has changed? Probe workload
15. Is there anything about your role that concerns you? What?
    a. Probe working outside are of expertise
    b. No indemnity if make an error
    c. Communication about working across disciplines

For peer review only - http://bmjopen.bmj.com/site/about/guidelines.xhtml

16. What preparation for the changes to your role have you had and how was it delivered (skills - key ones, psychological support)? What have been the successes and challenges?
    a. Probe PPE training
    b. COVID clinical training
    c. Support mechanisms
    d. Team formation
17. What kind of support (e.g. communication, occupational safety including PPE, psychosocial support) are you receiving to do your job from your team/manager/employer? What have been the successes and challenges?

Recovery post COVID-19

18 . Are there any COVID-19-related changes or innovations to routine health services that you think it would be useful to continue after COVID-19? Which ones and why?

19. What next steps do you believe should be taken now to support the health system to recover post COVID-19?

20. What is worrying you most as the response moves forward?

Thank-you

Do you have any further suggestions for improvements to delivery of routine services?

Any other comments?

For peer review only

For peer review only - http://bmjopen.bmj.com/site/about/guidelines.xhtml

Key Informant Interviews Topic Guide –Merseyside Laboratory and Blood Transfusion Staff

Version1.1_01052020

All possible questions to be asked of key informants are described in the following guide. Prior to interviewing each stakeholder, specific guides for these individuals will be made. One interview that covers relevant research themes will be completed with each stakeholder. Ordering of questions will also be revised to ensure logical flow through the interview and to avoid repetition.

Background

Please can you tell me your position and how long you have worked in your current role?

Governance and Decision Making - Relating Directly to COVID-19

1. What has been the decision-making process for the laboratory's response to COVID-19 testing services and when did discussions start around re-adjusting services for COVID-19?
2. Who held overall responsibility for how COVID-19 testing was going to be conducted at LCL?
3. In addition to PHE, have the Liverpool Clinical Laboratory services worked closely/ collaborated with any other external partners for COVID-19 testing? If so whom and in what capacity?

Governance and Decision Making - Relating to Maintaining Routine Service Delivery

4. How are decisions made about which services should or should not be prioritised; which ones were considered to be essential and why? Who is involved in this decision making? How were these decisions communicated?
5. What guidance documents were most useful to you in making these decisions? In what way were they useful?
6. What key challenges have you faced in making these decisions? Do you have any support needs here?

Impact of COVID-19 on Routine Laboratory Service Delivery

7. Can you tell me about how routine clinical laboratory service delivery has been affected by the COVID pandemic?

COVID-19 Testing service specific

8. How did the laboratories adapt to scale up COVID-19 testing? (analysers, staff capacity, staff training, standard operating procedures, risk assessments)
9. What challenges did the laboratory face when implementing COVID-19 testing? How were they overcome? What worked well? (e.g. resources, human resource, process change, governance, culture, leadership etc)
10. Which routine services would you envisage will be most impacted moving forwards? (e.g. hospital based-testing, disease specific services, etc) Why?

Recovery post COVID-19

11. Are there any COVID-19-related changes to the laboratory service that you think it would be useful to continue after COVID-19? Which ones and why?
12. What next steps do you believe should be taken now to support the laboratory system to recover post COVID-19?
13. Are there any changes/ innovations introduced in response to COVID-19 changes which you think should be continued? Why?

Thank you

Do you have any questions for me? Resources (re labs) link https://www.rcpath.org/uploads/assets/90111431-8aca-4614-b06633d07e2a3dd9/Guidance-and-SOP-COVID-19-Testing-NHS-Laboratories.pdf

COVID-19 Key Informant Interview Topic Guides

For peer review only

Table 1 Standards for Reporting Qualitative Research (SRQR)

Standard	Page number
S1 Title	P1, line 1-2
S2 Abstract	P2, line12-35
S3 Problem formulation	P3 line 53-75
S4 Purpose of research question	P4, line 78-124
S5 Qualitative approach and research paradigm	P11 line229-232
S6 Research characteristics and reflexivity	P8 line 165-190
S7 Context	P7 line 129-164
S8 Sampling strategy	P10line200-212
S9 Ethical issues pertaining to human subjects	P11 line 255-262
S10 Data collection methods	P8 line 167-171
S11 Data collection instruments and technologies	P9 line 191-198
S12 Units of study	P10 line219-224
S13 Data processing	P11 line 224-226
S14 Data analysis	P11 line 232-249
S15 Techniques to enhance trustworthiness	P 11 line 226-227
S16 Synthesis and interpretation	P 13 line 267-410
S17 Links to empirical data	P13 line 267-272
S18 Integration with prior work, implications, transferability, and contributions to the field	P21 line 411-523
S19 Limitations	P27 line 524-542
S20 Conflicts of interest	P28 line 565-566
S21 Funding	P28 line558-562

For peer review only

BMJ Open

Qualitative study exploring lessons from Liberia and the UK for building a people-centred resilient health systems response to COVID-19

Journal:	*BMJ Open*
Manuscript ID	bmjopen-2021-058626.R3
Article Type:	Original research
Date Submitted by the Author:	27-Jun-2022

Complete List of Authors:	McCollum, Rosalind ; Liverpool School of Tropical Medicine, International Public Health<br>Zaizay, Zeela ; Actions Transforming Lives<br>Dean, Laura; Liverpool School of Tropical Medicine, Department of International Public Health<br>Watson, Victoria; Liverpool School of Tropical Medicine (LSTM), International Public Health<br>Frith, Lucy; The University of Manchester, Centre for Social Ethics & Policy, School of Law<br>Alhassan, Yussif; Liverpool School of Tropical Medicine, International Public Health<br>Kollie, Karsor; Ministry of Health<br>Piotrowski, Helen; Liverpool School of Tropical Medicine, Department of International Public Health<br>Bates, Imelda; Liverpool School of Tropical Medicine<br>Anderson De Cuevas, Rachel; University of Liverpool, Institute of Population Health<br>Harris, Rebecca ; University of Liverpool, Institute of Population Health<br>Chowdhury, Shahreen; Liverpool School of Tropical Medicine, International Public Health<br>Berrian, Hannah; University of Liberia-Pacific Institute for Research and Evaluation<br>Smith, John; University of Liberia-Pacific Institute for Research and Evaluation<br>Tate, Wede; University of Liberia-Pacific Institute for Research and Evaluation<br>El Hajj, Taghreed; Liverpool School of Tropical Medicine<br>Ozano, Kim; Liverpool School of Tropical Medicine<br>Hastie, Olivia; Liverpool School of Tropical Medicine<br>Parker, Colleen; Ministry of Health Liberia<br>Kollie, Jerry; University of Liberia-Pacific Institute for Research and Evaluation<br>Zawolo, Georgina; University of Liberia-Pacific Institute for Research and Evaluation<br>Ding, Yan; Liverpool School of Tropical Medicine, Centre for Capacity Research<br>Dacombe, Russell; Liverpool School of Tropical Medicine, International Public Health<br>Taegtmeyer, Miriam; Liverpool School of Tropical Medicine, International

	Public Health<br>Theobald, Sally ; Liverpool School of Tropical Medicine, International Public Health
**Primary Subject Heading**:	Global health
Secondary Subject Heading:	Health services research, Health policy
Keywords:	COVID-19, HEALTH SERVICES ADMINISTRATION & MANAGEMENT, PUBLIC HEALTH, QUALITATIVE RESEARCH

SCHOLARONE™ Manuscripts

I, the Submitting Author has the right to grant and does grant on behalf of all authors of the Work (as defined in the below author licence), an exclusive licence and/or a non-exclusive licence for contributions from authors who are: i) UK Crown employees; ii) where BMJ has agreed a CC-BY licence shall apply, and/or iii) in accordance with the terms applicable for US Federal Government officers or employees acting as part of their official duties; on a worldwide, perpetual, irrevocable, royalty-free basis to BMJ Publishing Group Ltd ("BMJ") its licensees and where the relevant Journal is co-owned by BMJ to the co-owners of the Journal, to publish the Work in this journal and any other BMJ products and to exploit all rights, as set out in our licence.

The Submitting Author accepts and understands that any supply made under these terms is made by BMJ to the Submitting Author unless you are acting as an employee on behalf of your employer or a postgraduate student of an affiliated institution which is paying any applicable article publishing charge ("APC") for Open Access articles. Where the Submitting Author wishes to make the Work available on an Open Access basis (and intends to pay the relevant APC), the terms of reuse of such Open Access shall be governed by a Creative Commons licence – details of these licences and which Creative Commons licence will apply to this Work are set out in our licence referred to above.

Other than as permitted in any relevant BMJ Author's Self Archiving Policies, I confirm this Work has not been accepted for publication elsewhere, is not being considered for publication elsewhere and does not duplicate material already published. I confirm all authors consent to publication of this Work and authorise the granting of this licence.

For peer review only

Title Qualitative study exploring lessons from Liberia and the UK for building a people-centred resilient health systems response to COVID-19

Authors: Rosalind McCollum*^, Zeela Zaizay*, Laura Dean, Victoria Watson, Lucy Frith, Yussif Alhassan, Karsor Kollie, Helen Piotrowski, Imelda Bates, Rachel Anderson De Cuevas, Rebecca Harris, Shahreen Chowdhury, Hannah Berrian, John Smith, Wede Tate, Taghreed El Hajj, Kim Ozano, Olivia Hastie, Colleen Parker, Jerry Kollie, Georgina Zawolo, Yan Ding, Russell Dacombe, Miriam Taegtmeyer, Sally Theobald

*Joint first authors

^Corresponding author email address: Rosalind.mccollum@lstmed.ac.uk

For peer review only

For peer review only - http://bmjopen.bmj.com/site/about/guidelines.xhtml

Abstract

Introduction: COVID-19 has tested the resilience of health systems globally and exposed existing strengths and weaknesses. We sought to understand health systems COVID-19 adaptations and decision-making in Liberia and Merseyside, UK.

Methods: We used a people centred-approach to carry out qualitative interviews with 24 health decision-makers at National and County Level in Liberia and 42 actors at County and hospital level in the UK (Merseyside). We explored health systems' decision-making processes and capacity to adapt and continue essential service delivery in response to COVID-19 in both contexts.

Results: Study respondents in Liberia and Merseyside had similar experiences in responding to COVID-19, despite significant differences in health systems context, and there is an opportunity for multi-directional learning between the global south and north. The need for early preparedness; strong community engagement; clear communication within the health system, and health service delivery adaptations for essential health services emerged strongly in both settings. We found the Foreign, Commonwealth and Development Office (FCDO) principles to have value as a framework for reviewing health systems changes, across settings, in response to a shock such as a pandemic. In addition to the eight original principles, we expanded to include two additional principles; 1) the need for functional structures and mechanisms for preparation and 2) adaptable governance and leadership structures to facilitate timely decision-making and response coordination. We find the use of a people-centred approach also has value to prompt policy makers to consider the acceptance of service adaptations by patients and health workers, and to continue the provision of 'routine services' for individuals during health systems shocks.

Conclusion: Our study highlights the importance of a people-centred approach, placing the person at the centre of the health system, and value in applying and adapting the FCDO principles across diverse settings.

For peer review only - http://bmjopen.bmj.com/site/about/guidelines.xhtml

Strengths and Limitations of the Study

- A key strength of this study is the multi-directional learning between health systems in the global south and global north, which involved a wide range of researchers across both settings, and the breadth of perspectives captured from frontline staff and key decision-makers.
- The greatest limitation of this study is that it was carried out at a single point in time, towards the end of the first wave in the UK and before there had been a large increase in cases in Liberia. Response measures have evolved in both settings in subsequent stages of the pandemic.
- The study was limited by the differing range of respondents across study settings, with participants from across a range of health system levels including primary care, hospital frontline workers and decision-makers, as well as regional decision-makers within Merseyside, UK; compared with national and county level decision-makers, technicians and supervisors of frontline staff in Liberia, which may result in differing perspectives.

For peer review only

Introduction

The COVID-19 pandemic has forever altered our world. Its impact has been felt across all nations, demonstrating the importance of resilient health systems in protecting global health security.[1] Health systems have been forced to adapt to new ways of working alongside the continued provision of essential services including: prevention of communicable diseases; sexual and reproductive health; care for vulnerable populations; ongoing management of chronic illness (including mental health conditions); continuity of critical inpatient therapies; management of emergency health conditions; and auxiliary services, including diagnostic imaging, laboratory and transfusion services.[2]

In April 2020, the United Nations expressed concern that, within Africa, up to 3.3 million people could lose their lives as a direct result of COVID-19 and many more through the indirect effects of disruption to health services and worsening socioeconomic conditions.[3] Conditions considered to increase the risk of infection include overcrowded and poorly serviced slum dwellings; limited access to basic handwashing facilities; high levels of informal employment limiting ability to work from home; high levels of malnutrition and lower ratios of beds and health workers to the population.[3] A commentary published by Agyeman et al. (2020) at the outset of the pandemic highlighted a rapid response within many African settings, including a focus on early introduction of screening procedures at ports of entry, and a need for effective community engagement to educate about the mode of transmission. Key protective behaviours were emphasised, along with the need to prepare intensive care beds, and clear government strategies regarding how to deal with hospitalised COVID-19 patients to avoid disrupting the health system and to prevent non-COVID-19 related deaths.[4] Subsequent studies have revealed that indirect health impacts from COVID-19 disproportionately impact women and children.[5,6] Diversion of resources (financial, material, human) from existing health services to address the pandemic, impacts their care.[5,6] This includes supply and demand-side disruptions that can result in lower utilization of healthcare and, in some cases, impact on quality of care.[7] Bayani et al (2021)

For peer review only

surmise that “less healthcare will result in more ill health and deaths because health services have been suspended, displaced, or inaccessible.”(page 5 [7])

Our study was carried out immediately following the first wave of COVID-19 in Liberia and UK (interviews carried out June to September 2020) in response to an expressed need by stakeholders for this research following dialogue in both contexts. The study was conducted within these two contexts (Merseyside region and Liberia) based on strong prior research relationships within both settings. The differing perspectives from national and county respondents speaking on the national response in Liberia, and frontline health workers and decision makers up to regional level in Merseyside, based on their personal experiences and more localised regional response, is a key limitation. We chose these settings due to the opportunity and demand for research, not because they are exemplars of COVID-19 response. There is, however, still opportunity for learning and comparison on both the strengths and weaknesses within the COVID-19 initial response in both settings. The pandemic has continued to evolve across both settings, with both Liberia and UK experiencing much larger waves of COVID-19 since this original study was carried out. These findings from the first wave can provide valuable lessons to inform continued response to COVID-19 and other health systems shocks.

The pandemic has revealed monopolies of knowledge production, which disempower lower and middle-income countries;[8] whilst pandemic responses in ‘developed democracies’ have been inadequate, with cuts to health and social services and limited commitment to equity or governance.[8] So-called “global powerhouses with tried and tested health systems have struggled to contain the COVID-19 pandemic”[9] and health systems have been stretched to the limit, resulting in negative implications for the health of all populations, particularly when access for patients with other acute and chronic illness is limited.[8] As of September 1st 2021, the UK (population 66.8 million)[10] has 6,821,356 confirmed cases and 132,859 COVID-19 related deaths.[11] In the UK, the National Health Service delivers care for most of the population. Meanwhile during the same time period, Liberia (population 4.9 million)[10] has had 5594 confirmed cases, with 245 confirmed COVID-19 related deaths.[11] There are marked

For peer review only - http://bmjopen.bmj.com/site/about/guidelines.xhtml

differences between settings in the roll-out and scope of testing capacity and uptake of this, with under-reporting in many lower middle income countries, and so these figures cannot be assumed to be accurate. Future comparisons will eventually show the magnitude of all-cause mortality by age, and firm conclusions can be made about the success of different country approaches. Liberia was initially hailed as one of the top countries in fighting COVID-19, being one of the first countries to start screening at ports of entry (January 2020) and to adopt other control measures such as rapid testing, contact tracing and quarantine.[12,13]

Improving resilience within health systems can build on pre-existing strengths to enhance the readiness of health system actors to respond to crises, while also maintaining core functions.[1] People-centred health systems are a critical framing in shaping resilience as they place people and communities at the centre, whilst also promoting strategic and collaborative multi-sectoral leadership which is necessary in delivering a co-ordinated response to a public health crisis.[14] In this paper, we compare health systems responses at a single point in time (June to September 2020) within Monrovia, Liberia and Merseyside, UK, to distil lessons for health systems resilience to a pandemic through comparative case studies which explore aspects of health systems resilience.[15] Within this paper we combine the Foreign, Commonwealth and Development Office (FCDO) eight key principles for promoting resilient health systems with key domains and values of people-centred health systems to frame our findings in relation to the COVID-19 response.[16] Through our discussion we reflect on these expanded principles for resilience against our conceptual framework (figure 1), which is based on a people-centred approach. In response to calls for on-the-ground analysis of the response to COVID-19 within the Global South and comparative case studies that use co-creation and co-production approaches which go beyond researchers, including policy makers, practitioners and the public,[15,17] we seek to share learning from the response within Liberia and the UK, along with opportunities for multi-directional knowledge sharing.[17] It is our hope that this paper will help inform health policy makers across global contexts, for the current pandemic response and as they plan towards more resilient people-centred health systems to meet future shocks.

For peer review only - http://bmjopen.bmj.com/site/about/guidelines.xhtml

Methods

Study context

Liberia and UK have had very different strategies and case rates from the outset of the pandemic, although there were some similarities in the adoption of infection prevention control measures across both contexts. Liberia is amongst the world's poorest in terms of GDP and living conditions. According to the World Bank 2016 poverty headcount ratio, 44.4% of Liberians live below the international poverty benchmark of $1.90 USD per day.[18] The UNDP Human Development Report 2020 ranks Liberia low at 175 out of 189 countries and territories.[19] Inequities between females and males are remarkable with literacy rates (secondary education) of 18.5% and 40.1% respectively.[19] Liberia has prior experiences of shocks in the form of two civil wars, and the 2014-2015 Ebola Virus Disease (EVD) epidemic.[20] In response to these experiences, Liberia has prioritised rebuilding a resilient health system, which acknowledges the critical role communities play in addressing their own health needs through the 'Investment Plan for Building a Resilient Health System in Liberia' and the community health services policy (2016-2021).[21,22] By contrast, Merseyside is a Metropolitan County in the North West of England, comprising five boroughs, including the City of Liverpool, including some of the most deprived council areas in England.[23] It has a population of 1.42 million and has had some of the highest numbers of COVID-19 cases in the UK.[24] Within Merseyside, the Liverpool City Region Combined Authority has prioritised tackling deprivation and reducing health inequalities through people-centred care, with integration of health and social care services.[25] Liverpool has a long history of public health innovation, but also a strong sense of local history, culture and place. Throughout the pandemic Liverpool has been at the forefront of community-based innovations and public health strategies, e.g. piloting community open access testing for COVID-19.[26]

Liberia introduced stringent border control measures from January 2020, with the establishment of a Special Presidential Advisory Committee on Coronavirus (SPACOC) over two months prior to the first recorded cases in the country.[27],[28] Liberia's response to COVID-19, prioritised a call to maintain

For peer review only - http://bmjopen.bmj.com/site/about/guidelines.xhtml

the delivery of routine health services at all levels. Hospitals and clinics continued to provide health services with health facility workers trained in infection prevention control (IPC) before the first case was identified in country.[28] Physical distancing measures were introduced and use of face masks encouraged.[29]

Within the UK, health service delivery was restructured as part of the COVID-19 response, with routine non-urgent elective care suspended and later re-started in April 2020.[30] Adaptations to minimise potential risk of COVID-19 infection include the use of telemedicine and phone consultations; and changes to essential services for patients, such as changed treatment plans and delays to surgeries.[31] Hospital patient pathways were altered to appropriately triage and cohort the care of COVID-19 patients, reducing the risk of transmission to others and allowing essential services to continue. There was also reduction in routine blood test screening to prioritize COVID-19 PCR testing in response to the UKs 'test and trace' strategy.

Study aim, design and conceptual framework

Aim: To understand COVID-19 adaptations and decision-making in Liberia and Merseyside, UK

This qualitative study explored inductively the differing experiences, perspectives and recommendations of participants in order to understand COVID-19 adaptations and decision-making in Liberia and Merseyside, UK.[32,33] We selected qualitative methods to give "due emphasis to the meanings, experiences, and views of all the participants"(page 43 [32]) and understand decision-making and the impact of health systems adaptations as a result of COVID-19.

A conceptual framework was jointly developed, following a series of meetings held with researchers in each setting (7 Liberia-based researchers and 18 UK-based researchers). This framework sought to consider a people-centred approach towards the health system's ability to respond to shock, whilst reflecting the realities experienced in the face of multiple routine challenges (Figure 1).[34] The nature of a shock to the health system, whether due to infectious disease outbreak, natural disaster,

For peer review only - http://bmjopen.bmj.com/site/about/guidelines.xhtml

or conflict, influences the rest of the framework.[35] It adopts a people-centred approach at its heart,[14,36,37] while incorporating literature relating to the health system's ability to respond to a sudden shock, and the extent to which it is able to absorb, adapt and transform in response (Figure 1).[35,38–42]

People-centred health systems prioritise the collective right to health through integrated and targeted approaches that favour the needs of the most vulnerable.[14,43] Collective action and social solidarity are viewed as essential to the art and science of the development of people centred systems that are organised around people's healthcare needs and expectations as opposed to diseases, ensuring a continuum of care throughout the life course.[14] This approach embraces the human character of health systems, by viewing individuals, communities and health workers as co-producers of healthcare, placing people and families at the centre.[44] Systems must adapt to meet a range of challenges to support the development of strategies that seek to improve healthcare access and encourage universal coverage. This is particularly important as many individuals transition and oscillate between multiple roles of patient, family and sometimes healthcare provider within one system.

Interview topic guides were informed by the framework and developed across both settings to explore key areas of health systems functioning in response to COVID-19 (Appendix 1). Questions included: governance and decision-making; use of ethical guidelines; human resource management, infrastructure (information technology and communications) and healthcare worker support; introduction of innovations; and perceptions of the equity and quality of service delivery. Adaptations were made according to the health systems context in each country, for example in Liberia, additional questions were included to explore how learning from the EVD epidemic and other health systems shocks informed COVID-19 response planning.

Figure 1 placed here

<u>Study participants and data collection</u>

For peer review only - http://bmjopen.bmj.com/site/about/guidelines.xhtml

The study was carried out at different levels of the health system across both settings (Table 1). In Liberia, we conducted key informant interviews in June and July 2020 with 21 national level and 3 county level decision-makers (Nimba, Margibi and Montserrado Counties) purposively selected because of their involvement with COVID-19 planning and/or routine service delivery. Some had also played key roles in the EVD epidemic response. In Merseyside we conducted 42 key informant interviews between July to September 2020, with regional, hospital and primary care decision-makers (general practitioners and residential care home managers) and front-line workers selected because of their involvement with COVID-19 planning and/or the delivery of COVID-19 or routine services (see Table 1). More interviews were carried out within the UK across health systems levels, due to demand for research across multiple levels and the presence of a larger team of researchers. In Liberia, by contrast the demand for research was focused at national level, and the research team was smaller in size. The national and county level actors in Liberia, spoke about Liberia's response as a country. In contrast study participants in Merseyside from across health systems levels, including frontline health workers, spoke of their own direct experience within a particular hospital or setting, or on behalf of Merseyside City Region. We acknowledge the limitation that including national and county level actors only within Liberia, creates a somewhat limited perspective. It would have been preferable to have included a larger number and range of participants from sub-national health systems levels to provide more depth of understanding about the COVID-19 response.

Table 1 Study participants' role

**Participant Role**	**Number of Participants Interviewed**
**Merseyside, UK**	
Regional decision-maker	5
Hospital decision-maker (Clinical director, medical director, ward manager)	4
Hospital consultant	11
Hospital health worker (junior doctors, nurses)	10
Health worker in community (GP, district nurse, residential care home)	7
Liverpool Clinical Laboratory staff	5
**Total**	**42**

For peer review only - http://bmjopen.bmj.com/site/about/guidelines.xhtml

**Liberia participants**	
National decision-maker	21
County decision-maker	3
**Total**	**24**

Interviews were predominantly carried out remotely by researchers experienced in qualitative interviewing in English language, via online platforms such as Microsoft Teams or Skype. A minority were carried out in person with physical distancing measures in place, according to local guidance at the time. All interviews were audio-recorded. Data collection stopped when no new themes emerged from additional data collected.[45] Interviews lasted approximately 30 to 60 minutes. Audio recordings were transcribed verbatim, with quality assurance conducted by a second researcher against the recording.

Data Analysis

The study has sought to use a pragmatic approach to research, working through existing networks to carry out timely research to support the ongoing COVID-19 response in both settings. Both inductive and deductive approaches were blended within data analysis, in keeping with other health systems research [46–49]. In both Liberia and UK, preliminary data analysis workshops were held separately with the research team members involved with data collection. Prior to the workshops all participants reviewed transcripts to familiarise and immerse themselves within the data in order to inductively identify emerging themes which arose from within the study findings. Through these separate country workshops key themes were identified and used to generate a separate coding framework for each setting. All transcripts were imported into NVivo Version 12 qualitative data analysis software for coding (QSR International Pty Ltd. Version 12, 2018). Following review of the initial themes which emerged inductively from within the data, there was found to be strong alignment with the eight FCDO principles. These principles were then deductively applied to assist with mapping the findings and enabling comparison between settings. The research team did not simply accept the eight FCDO principles, rather the team reviewed them and found that they did not fully cover all the aspects of

For peer review only

240 resilience which emerged from the data. As a result, two further principles were identified and applied
241 to adequately compare findings between both settings, relating to "mechanisms for advance
242 preparation" (Principle 9) and "adaptable governance and leadership structures" (Principle 10). The
243 application of the expanded FCDO principles for resilience has helped to showcase how Liberia's
244 experience with responding to prior shocks and their learned need for early advance preparedness
245 provided an important element working towards resilience. This study is not funded by FCDO, nor
246 were FCDO involved in any way as researchers or co-authors within the research team.

247 Detailed findings and recommendations were developed into two policy briefs in accordance with
248 these expanded principles for resilience and were shared and discussed with relevant stakeholders
249 from both study settings.[29,50] The relationship of the findings to the original conceptual
250 framework was reviewed and findings compared between settings during a final on-line workshop,
251 attended by all those involved with data collection in both settings, with key similarities and
252 differences jointly discussed.

253 Ethics

254 Ethical approval was received from the Liverpool School of Tropical Medicine Research Ethics
255 Committee (Protocol ID 20-045); the University of Liverpool Ethics Committee (Reference 7811) and
256 the University of Liberia-Pacific Institute for Research and Evaluation Institutional Review Board;
257 National Health Service Health Research Authority and Health and Care Research, Research Ethics
258 Committee (Reference 20/HRA/2597); Integrated Research Application System (Project ID 284143).
259 All study participants were provided with a participation information leaflet at least 48 hours prior to
260 interview. All participants provided written, or audio recorded consent to participate.

261 Patient and public involvement

262 Neither patients nor the general public were involved in the design, conduct, reporting or
263 dissemination of our research.

For peer review only - http://bmjopen.bmj.com/site/about/guidelines.xhtml

Results

We present findings according to the expanded FCDO principles for resilience (Box 1) (key illustrative quotes are summarised for each principle in table 2). We then reflect on the findings in light of people-centred health systems within the discussion.

Box 1 Expanded Principles of Health Systems Resilience in the Context of COVID-19 Response

Principle 1 Develop flexible pathways for medical supplies
Principle 2 Prioritise a list of essential health services [*and continued provision of quality and equitable routine services*]
Principle 3 Build trust with local communities
Principle 4 Foster good communication at all system levels
Principle 5 Support, recognise and encourage staff
Principle 6 Facilitate rapid resource flow and greater flexibility in its use
Principle 7 Ensure agile tracking of health information
Principle 8 Cultivate effective partnerships and networks
Principle 9 Structures and mechanisms for advanced preparedness (**New principle**)
Principle 10 Adapt governance and leadership structures to facilitate timely decision-making and effective coordination of response **(New principle)**

Table 2 Illustrative quotations from Liberia and Merseyside related to each FCDO Principle

Principle	Comparison	Quotations
**Principle 1: Develop flexible pathways for medical supplies**	Supply chains disturbed across settings due to global shortages and price inflation.<br>Lack of buffer stock in both settings.<br>Restructuring of supply chains in Liberia led to disturbance for routine supplies.	*"Supply chain are affected greatly because their concentration is on how to provide the COVID response activities meaning the …medicines and medical supplies that are needed [for] NTDs (Neglected Tropical Diseases), lack of attention will now be paid to that." (LIB national decision maker 029)*<br>*"With regards to PPE, there was national guidance about what we should do and there was a huge amount of fear amongst nurses and medics and everyone else understandably. Everyone was scared. I was scared. If someone said they weren't scared, then they're lying or they're a fool. The national guidance was confused, and availability of PPE fluctuated. Procurement here [NHS hospital] did a very good job, but sometimes it just wasn't delivered nationally. And we went through other supply chains…" (LIV hospital decision maker, Merseyside UK 014)*
**Principle 2: Prioritise a list of essential health services [and continued provision of quality and equitable routine services]**	Discontinuation of elective non-urgent care in UK, contrasts with early emphasis on continued routine care in Liberia.	*"So we just have to be robust and do the necessary investment into routine health services, preventive in terms of creating awareness and education among health workers about covid and how we can continue to care for our patients, with fighting the infection at the same time." (LIB national decision maker 001)*

review only

Principle	Comparison	Quotations
		"There's the whole big risk around the screening program…the screening program was stopped, restarting that it's gonna be really challenging. And I suppose that's another risk in terms of people with delayed diagnosis and the right treatment, as a result of not having had that screening mammograms." (LIV hospital decision maker Merseyside UK 051)
**Principle 3: Build trust with local communities**	Both settings experiences reduced service utilisation due to loss in community trust. Introduction of innovative follow-up visits to patients led to increased service use in Liberia.	*"Some of the useful things that we have been using from Ebola time is, as I said before, to involve the communities …The community aspect is very important because it will help us for the COVID-19 where communities, family members, all of those at the community level are influential group they will be able to comply like we did in the Ebola." (LIB national decision maker 005)* *"The elderly population have been shielding because of comorbidities and all that. I think they probably not being as vocal about things that they're concerned about because they're worried about that they will be asked to come in. They fear that that they will catch Covid when they come here." (LIV hospital health worker Merseyside UK 048)*
**Principle 4: Foster good communication at all system levels**	Expansion of virtual communication in both settings. In Merseyside frequently changing guidance from multiple sources created confusion.	*"One of the things that quickly used to come to me is to able to adapt to working with social media technology and all of that, because that's the first thing if you have to communicate with people in this manner you need to understand zooming, skyping, how to take notes.." (LIB national decision maker 029)* *"And there's so many different sources of information that say different things from what people hear within the hospital talking to friends on the corridor, that you've got to come out with a consistent message. And I think it took longer than was ideal to get a central source of information…But people need to be told what the situation is rather than try to be falsely reassured sometimes as well." (LIV hospital decision maker, Merseyside UK 004)*
**Principle 5: Support, recognise and encourage staff**	Health worker redeployment was common across settings. Health worker training varied in UK according to cadre.	*"Like take for example, when COVID came some of our workers from the [name] Hospital was recruited to go at the front line and [hospital name] is for routine services so taking employees from there to go at the front line that tells you it kind of understaff… So routine services kind of slow down and every attention was placed on COVID but going forward, with the system in place, routine services have gotten back on its feet." (LIB national decision maker 010)* *"And it felt like there was unequal share of knowledge and also an unequal kind of confidence in protective clothing. … And I think the people that spent the most time with the patient, the patient*

For peer review only - http://bmjopen.bmj.com/site/about/guidelines.xhtml

Principle	Comparison	Quotations
		areas, for instance, the healthcare assistants and the cleaning staff didn't have all of the information [at the] beginning or any PPE training." (LIV hospital health worker Merseyside UK 017)
**Principle 6: Facilitate rapid resource flow and greater flexibility in it's use**	Prior under-investment in health was common across settings.<br>In Merseyside there was increased funding available and removal of bottlenecks, which enabled swifter action.	*"The first thing is, we need ownership by government, ownership is not depending on other countries to provide us the resources, to provide the technical capacity. So that is the best recommendation I would say. The ownership has to be there, resources have to be available and the infrastructure has to be available in terms of being resilient." (LIB national decision maker 029)*<br>*"To be honest, it was a fairly novel experience because it was a situation where if we asked we more or less got [funding]." (LIV hospital decision maker, Merseyside UK 004)*
**Principle 7: Ensure agile tracking of health information**	Data quality reduced in Liberia.<br>In Merseyside increased data was collected, but inadequate data analysis measures were put in place.	*"Another recommendation is that we could include COVID-19 to our regular disease surveillance. Like we have the measles, the Lassa, and thing. I think we should include COVID because COVID maybe all around. Like we included Ebola, there should be a document on COVID-19 that will form part of our regular surveillance." (LIB county decision maker 024)*<br>*""...there's some value in looking at the things that we were looking at before COVID, because at least we have some longitudinal data on that so that we can see what the effect of COVID is." (LIV hospital health worker, Merseyside UK 020)*
**Principle 8: Cultivate effective partnerships and networks**	Liberia was able to call upon prior decision-making structures (established during Ebola response) to enable swift decisions.<br>Need for stronger engagement between primary and secondary care in Merseyside.	*"Involvement of multi-sectorial stakeholders in the response; that was one major thing that we learned from Ebola. And that has been brought to be on this response, so there has been a spark from the level of the presidency where they have key ministries and agency heads heading pillars on the COVID-19 response, involving the community people." (LIB national decision maker 028)*<br>*"I think one thing, it's really highlighted is the divide between hospital and primary care. We didn't work together very well before the epidemic, and we are still not working together very well. And I think if things were to get better, the whole health system needs to work better." (LIV community-level health worker, Merseyside UK 033)*
**Principle 9: Structures and mechanisms for advanced preparedness**	Learning from Ebola prompted rapid preparedness in Liberia, in contrast to Merseyside.	*"If you don't prepare well and you are caught unaware you will have a lot of issues, so we didn't wait for COVID to enter Liberia before we prepositioned basic PPE and those are all part of the preparedness phase." (LIB county decision maker 026)*<br>*""It was blatantly obvious that anything we've ever planned for in relation to a pandemic or anything along those lines was not the plans that we needed... So I think going forward there needs to be almost a better planning system in place...it's not*

For peer review only - http://bmjopen.bmj.com/site/about/guidelines.xhtml

Principle	Comparison	Quotations
		just a matter of just saying any pandemic it's about what kind of pandemic." (LIV hospital decision maker, Merseyside UK 069)
**Principle 10: Adapt governance and leadership structures to facilitate timely decision making and effective coordination of response**	Need for rapid guidance from national level to enable sub-national decision making was common in both settings.	*"So, at this point in time we think if you give the resources, put the money in the hands of the county health team to buy what they need, that will be more effective … So, we want decision should be given back to the people on the frontline so that they make the decision rather than a centralized point in Monrovia where people sit and decide for people in the lower level and the people choices made the right kind of thing they might need at that level."* (LIB national decision maker 028)<br>*"… we were having to work, to a large extent, in the dark. The amount of guidance that came through nationally and even regionally, was actually relatively limited at that stage and we were having to do what felt like quite a lot of planning in isolation."* (LIV decision maker Merseyside UK 008)

For peer review only

Principle 1 Develop flexible pathways for medical supplies: Across both settings supply chains were disturbed due to global shortages and price inflation. In Merseyside there was a lack of personal protective equipment (PPE) and laboratory reagents needed for COVID-19 testing. Meanwhile, in Liberia, the disturbances related to routine supplies as supply chains shifted to focus on COVID-19 related procurement. In both settings, these challenges were felt to relate to global shortages, but were worsened by failure to maintain buffer stocks at local and national levels. In both settings, participants expressed the need for greater decentralisation of procurement decisions.

Principle 2 Prioritise a list of essential health services [*and continued provision of quality and equitable routine services*]: Participants from Merseyside expressed fears that there was too much emphasis on COVID-19 care, at times creating redundant capacity, while limiting access and quality of routine essential services. The blanket discontinuation of all elective non-urgent care at the height of the first wave in Merseyside, UK was felt to be unhelpful, and a more nuanced approach which seeks to balance long-term as well as short term risks associated with health conditions was recommended. In contrast, Liberia's early emphasis on routine health services was described as a key learning prioritised by decision-making platforms following the country's experience with the EVD epidemic.

For peer review only - http://bmjopen.bmj.com/site/about/guidelines.xhtml

286 COVID-19 adaptations in the UK led to increased telemedicine, with some respondents raising access-
287 related equity concerns, particularly for elderly populations, who may struggle to engage with
288 telemedicine. There were also concerns raised about quality of care, with some participants in
289 Merseyside fearing delayed-diagnosis, misdiagnosis or sub-optimal care due to restrictions limiting
290 physical contact with patients. In Liberia, limited opportunities for supervision, diversion of funds and
291 staff for routine services towards COVID-19 response, and limited community outreach activities (due
292 to physical distancing) were felt to impact quality of care. Across both settings innovations in service
293 delivery have emerged (see policy briefs for details).[29,50]

294 <u>Principle 3 Build trust with local communities</u>: In both settings, community trust to seek health
295 services declined, which reduced utilisation of services. In Liberia, fear among the population during
296 the start of the pandemic led to reduction in the uptake of health services including national routine
297 vaccination programmes and health facility-based delivery. This was felt to relate to a combination of
298 fear of contracting COVID-19 at facilities and to reduced community outreach activities. Innovative
299 community engagement and social mobilization strategies were introduced, for example follow-up
300 visits to pregnant women, which led to patients returning to use services after a few months. Another
301 example is the selective outreach home visits by the Neglected Tropical Disease (NTD) programme to
302 NTD affected patients, in order to avoid interruption in treatment provision. In Merseyside, utilisation
303 of non-COVID related services remained supressed for much longer. This was deemed to relate to
304 widespread community mistrust, and Government campaigns which initially discouraged the public
305 from visiting health facilities via the national 'Stay at home' messaging. Applying learning from
306 Liberia's experience with EVD, the Government of Liberia placed a strong emphasis on working
307 alongside community governance structures, involving local authorities as part of COVID-19 response.

308 <u>Principle 4 Foster good communication at all system levels</u>: The need for effective communication
309 within the health system appeared to be a significant theme, particularly within findings from
310 Merseyside. The rapidly changing context during the early months of the pandemic created a wealth

For peer review only

of daily new information. Virtual forms of communication rapidly expanded in both settings, with WhatsApp and online meeting platforms used extensively. Within Merseyside, referred to challenges such as multiple sources of guidance and communication channels struggling to keep pace with the changing guidance, which at times created contradictory messaging and confusion among health workers. By contrast, Liberia developed a centralised messaging procedure with approval needed from the department of Health Promotion before dissemination. In Merseyside, use of emails were typically less popular with staff as these could often be too long and wordy. Participants expressed limited scope for frontline staff to feedback on the information that had been shared.

Principle 5 Support, recognise and encourage staff: Staff redeployment was common across both settings, contributing to varied workloads. In Liberia, health worker redeployment to COVID-19 treatment centres, alongside largely unchanged utilisation rates contributed to increased workload for remaining health workers responsible for provision of routine services. By contrast in Merseyside, redeployment resulted in over-staffing in certain COVID-19 wards. Although there was disparity between health workers, with nurses experiencing increased workload. Due to the reduced volume of patients seeking routine care in the UK, workload was variable for those providing these services. The degree to which health workers received training about COVID-19 prior to having to manage COVID-19 patients varied between settings, with Liberia carrying out training in identification, isolation and infection, prevention and control, before the first case of COVID-19 arrived in country, as a result of lessons learned following experiences responding to EVD. By contrast in Merseyside, the roll out of training varied widely by cadre, with some participants identifying that healthcare assistants and cleaning staff did not receive PPE training until later in the pandemic, compared with doctors and nurses (see table 2).

Anticipated mental health implications for health workers emerged from the Merseyside data, due to high rates of COVID-19 infection, exhaustion and high future anticipated post-traumatic stress disorder (PTSD). This was associated with fear of making treatment mistakes, stress surrounding

For peer review only

336 patient escalation decision making, anxiety over potential COVID-19 infection (both personal and for family), trauma surrounding high COVID-19 infections and deaths and reduced psychosocial support due to remote working. Measures to support staff wellbeing were introduced (including counselling, reflective therapy, peer support and mentoring, information made available about local support services), with varied levels of uptake. This was not widely discussed in Liberia. Although measures in Liberia to support staff wellbeing include psychosocial teams, roaming mental health counsellors providing services to health workers are in place. In Merseyside, community support, strong solidarity and teamwork were considered enablers of staff resilience.

<u>Principle 6 Facilitate rapid resource flow and greater flexibility in its use</u>: Historic underfunding of the health system in both settings has been highlighted by the pandemic. In Merseyside, this was considered to be due to nearly a decade of austerity, which has created weariness and uncertainty; whereas in Liberia it related to perception of reliance on external donors which predated the pandemic. Our findings confirmed the need for adequate funding to ensure the building blocks of the health system have received investment prior to the onset of any shock. With the arrival of the pandemic the availability and flexibility of funding differed between settings. In Merseyside, UK, there was increased central government funding, which was mostly freed of usual bureaucratic checks. Managers noted that the removal of these bottlenecks allowed for swift action and rapid adoption of innovations. Frontline managers' ability to make operational decisions was viewed as central to resilience. In Liberia, however, there was an identified need for greater Government of Liberia ownership. Some sectors of the health system, particularly those which are donor reliant struggled in response to reduced partner support following the pandemic. Initially funding was not made available, however funds for routine service delivery were re-allocated to COVID-19 response, with implications for quality (see principle 2). Participants complained about excessive bureaucracy associated with use of funds, which created delays.

For peer review only - http://bmjopen.bmj.com/site/about/guidelines.xhtml

Principle 7 Ensure agile tracking of health information: Health information systems (HIS) were rapidly developed in the UK to collect huge quantities of surveillance data on COVID-19 and essential services. However, there was need for improved skills to usefully interpret this data. Respondents in Liberia stated that regular and timely submission of data, particularly from the community level had declined since the onset of COVID-19. This was considered to relate to reduced data validation, with decreased supervision visits due to physical distancing. In Merseyside complex new systems were designed to collect pandemic surveillance data, however, data was frequently not analysed or made readily accessible to staff to influence timely monitoring and quality improvement in services. In Merseyside, respondents also noted that a number of new initiatives were introduced during the pandemic, such as virtual consultations, but have not yet been systematically evaluated.

Principle 8 Cultivate effective partnerships and networks: The need for well-established partnerships emerged in both settings, with Liberia already having clear multi-sectoral participation in decision-making following the Incident Management System developed following EVD. Merseyside data highlighted pre-existing weaknesses in collaboration between primary and secondary/tertiary care have been exacerbated. In both settings the need for greater engagement with the private sector was affirmed, with respondents from UK highlighting the need for stronger links regarding PPE supply chain shortages and in Liberia the need to strengthen collaboration given perceived weakness in private facility IPC standards. Partnerships were established within Merseyside, in a range of aspects of service delivery, including: regional network of laboratory providers to address equipment challenges and ensure COVID testing; between GPs to create service hubs; between disciplines and departments within hospital to address staff shortages and share information. In Liberia, a reduction in the number of partners providing response support was noted. This was a marked contrast to the EVD response.

Principle 9 Structures and mechanisms for advanced preparedness (newly identified principle from our findings): Within Liberia in particular, but also in Merseyside, there was discussion about

For peer review only - http://bmjopen.bmj.com/site/about/guidelines.xhtml

385 advanced preparedness. Respondents in Liberia emphasised how their experiences with previous shocks, particularly EVD, had facilitated learning around early recognition of the need for preparedness. For instance, there was consensus among respondents that waiting for COVID-19 to reach Liberia before responding would be too late. There was early rapid mobilisation of existing emergency response systems which had been established during the EVD response, including; health check controls and quarantines at border points from January 2020; health worker COVID-19 training before the first confirmed case; enhanced hygiene practices; restriction of physical contact and sustained use of PPE, building on institutional memory gained through the EVD epidemic. In contrast, respondents in Merseyside expressed that the COVID-19 response was impeded by a lack of pandemic preparedness for new emerging infectious diseases.

395 Principle 10 Adapt governance and leadership structures to facilitate timely decision-making and effective coordination of response (newly identified principle from our findings): Being able to adapt governance and leadership structures to facilitate timely response coordination emerged from both settings. Liberia had previously established the incident management system (IMS) in 2014 as part of the response to EVD. It was re-activated in March 2020 to guide planning their pandemic response, led by the Minister of Health. This multi-sectoral team included a range of political and public health decision-makers, donors and partner representatives. At the time the study was carried out, most decisions were made centrally, with implementation at county level. In Merseyside, early response was hindered by slow and centralised guidance and decision-making, which was perceived to be oriented towards achieving political goals, rather than providing much needed clarity and recognition of local reality. The limited scope for local autonomy was considered to strain relationships between local senior leadership who sought to enforce central directives, and frontline staff, who wanted scope to influence them. In both settings, there was interest in greater de-centralisation of decision-making to lower levels.

Discussion

For peer review only - http://bmjopen.bmj.com/site/about/guidelines.xhtml

410 Our findings demonstrate the commonalities between the principles for resilience and people-centred health systems (Figure 2). We believe that maintaining a people-centred approach can help ensure that COVID-19 related adaptations are acceptable, understood and meet the needs of individuals (both patients and health workers). The values which underpin people-centred health systems emphasise the need for equity, orienting health services towards a health system which puts "people and communities at their centre, and surrounds them with responsive services that are coordinated both within and beyond the health sector, irrespectively of country setting and development status."(page 9 [14])

418 **Adapting a people-centred framework**

419 All ten FCDO principles (eight original principles and two principles identified through this study) are mapped against the original conceptual framework, to demonstrate the connection between our findings and existing literature about resilience (Figure 2) and recommendations in response to each principle are outlined in box 2.

423 Figure 2 placed here

For peer review only

For peer review only - http://bmjopen.bmj.com/site/about/guidelines.xhtml

Capacity and knowledge exchange

The continuation of routine essential service delivery following a shock to the health system, has previously been highlighted as an area of concern across a range of sectors.[51,52] Health systems

Box 2 Recommendations from expanded FCDO principles for resilience

1. Supply chains should pre-position adequate stocks, diversify sources and seek decentralisation of procurement. Collaboration between providers can prove valuable in securing continuity of supplies.
2. Routine services should be prioritised with a view to long term as well as short term impact, with prioritisation re-evaluated regularly as the pandemic progresses.
3. Maintain consistent communication and engagement with community leaders, as partners, to participate in pandemic planning within their respective communities.
4. Keep communication channels open, with regular updates for staff which highlight the key information, preferably through meetings, rather than email.
5. Ensure adequate provision of training, with sufficient PPE for health staff, particularly for those staff at highest risk of COVID-19 infection, alongside measures to balance workload and promote staff wellbeing. Prioritise compassionate leadership which is supportive of staffing levels and rotas, along with staff mental wellbeing. Investment in psychosocial wellbeing throughout and after the pandemic response.
6. Health systems need to be adequately funded during 'normal times' if they are to be able to respond when a shock arises. There is urgent need for investment to clear the backlog of delayed routine services.
7. Health information systems need greater investment in both the systems and the human element to be able to analyse, interpret and respond to emerging data trends.
8. Opportunities for multisectoral collaboration should be sought out, with engagement with private sector where possible.
9. Develop a proactive approach, with advance plans for health shocks, along with escalation and de-escalation plans throughout the crisis.
10. Promote greater opportunities for de-centralised staff involvement in decision-making, where feasible. Governments to prioritise an outward focus towards global solidarity.

need the capacity to continue to deliver services of good quality alongside responding to wider health challenges.[42] Our findings for principle 2 highlighted that COVID-19 adaptations in the UK led to the cancelling or postponing of many essential services, including those related to cancer care, which has been anticipated to decrease life expectancy and survival.[52,53] Meanwhile, Liberia emphasised the need for continuation of routine services and the promotion of patient confidence to use these services. This is in contrast to the EVD epidemic, where over 80% reductions in maternal delivery care

For peer review only - http://bmjopen.bmj.com/site/about/guidelines.xhtml

in EVD affected areas were described and form part of the reason why routine care was prioritised so strongly as part of the COVID-19 response.[54]

Our findings relating to supply chain (principle 1) resonate with literature from previous shocks and research emerging from the COVID-19 pandemic.[55,56] We found the need for greater flexibility, with engagement with a more diverse range of suppliers and greater decentralised control over supply chain across both settings. This is in keeping with a recent systematic review of supply chain resilience literature, which identified the importance of diversity and the social aspects of supply chains during a pandemic response.[55] Supplying commodities without investing in health systems strengthening will not produce a robust supply chain, limiting ability to respond quickly and effectively to future demands.[55]

We found a strong focus on the need for support for the health workforce, particularly in UK (principle 5). This was not as widely discussed in Liberia (though this may be a limitation relating to differing levels of participants between countries). However, a previous study in Sierra Leone and Liberia, highlighted that many providers may carry unresolved trauma from earlier shocks (including the Ebola epidemic), which may have implications for them during the COVID-19 response.[57,58] Research among health workers treating patients with COVID-19 in China, revealed health workers had a higher prevalence of insomnia, anxiety, depression, somatisation and obsessive-compulsive symptoms compared with nonmedical health workers, indicating the need for support and recovery programs for these staff.[59] Stressors identified among workers in China, include many of those described by participants in both settings within our study, particularly within Merseyside, including difficulties feeling safe at work, lack of infection prevention and control (IPC) measures and COVID-19 knowledge, long term workload, high risk of exposure to COVID-19, shortage of PPE and lack of rest, among others.[59]

Our findings regarding resource flow to frontline providers (Principle 6), are in keeping with previous study which identified funding as a core dimension within a health systems' ability to adapt and

For peer review only - http://bmjopen.bmj.com/site/about/guidelines.xhtml

respond to shocks.[60] A recent systematic review found aggregate public spending for health is associated with improved life expectancy, reduced child and infant mortality and more equitable health outcomes.[56]

Relational and teamwork components

The relational components which exist are shaped by risk, trust, values, power, norms, and culture.[42] These components play a role in determining the success (or failure) in response to a health systems shock or crisis. In contrast to the FCDO recommendation for good communication between actors (principle 4), our findings highlight challenges, particularly in the UK, where communication channels struggled to keep pace with changing guidance creating contradictory messaging and confusion among health workers. This is in keeping with previous study which found differences in lines of authority and acceptability of communication pathways can contribute to problems in communication.[34] In response, key principles were identified including participation for all, respect, information sharing, collaboration and problem-solving.[34]

The need for strong governance structures and leadership which adapts to the response (principle 10), was identified as a gap within early response in Merseyside. This was felt to have been hindered by slow and centralised guidance and decision-making with a perceived limited scope for autonomy within decision-making at lower levels. Within Liberia learning from the EVD response, and establishing an incident management system (IMS) (led by the Minister of Health) and Special Presidential Advisory Committee on Coronavirus (SPACC) (led by the President) early in planning their pandemic response enabled timely decision-making.[27] In both settings, there was interest in greater de-centralisation of decision-making to lower levels. Blanchet et al (2017) emphasised the need for legitimacy within resilience, with requirement of capacity to develop socially and contextually accepted institutions and norms.[40]

Looking more broadly, the conceptual framework highlights community engagement, with the community being active participants of any health systems response (principle 3).[39] Our findings

For peer review only - http://bmjopen.bmj.com/site/about/guidelines.xhtml

emphasise the value of community engagement within the response within Liberia, based on lessons from the EVD pandemic and in keeping with WHO recommendation that this be a key pillar within COVID-19 country response.[8] Liberians across all socio-demographic groups responding to a recent survey said they were very well, or somewhat well informed about the COVID-19 pandemic, with only 5% feeling not very well/ not at all informed.[27] This also emerged as a key finding in Singapore, with engagement through new and social media channels monitored, with clarification of misinformation by MOH.[61] In contrast to the findings from Liberia, participants from Merseyside highlighted the need for stronger communication (although there were some examples of creative ways to engage with diverse communities).

Learning from our study has emphasised the need to better prepared for, and respond to, health emergency crises through integrated services (Principle 9).[44] A recent survey found most of the population felt the Liberian government was doing well in managing the pandemic.[44] This contrasted with findings from the UK where there was felt to have been a lack of adequate advance planning and preparation. Two previous literature reviews highlighted that "preparedness depends on health systems ability to learn from prior pandemics", with responses often reactive rather than proactive.[56,62]

The people-centred approach stresses the need for awareness and recognition of the interdependencies of the health system with the community and other social systems, including education, social protection and food security and their relationship with social determinants of health (principle 8).[63] Our findings emphasise the need for strong partnerships with other sectors across settings, in keeping with an identified success in Singapore's response,[61] and is a key aspect of Blanchet et al's resilience framework, ensuring the capacity to engage with, and handle, multiple actors and dynamics.[40]

Our findings, particularly from Merseyside emphasise the vast quantities of data being generated through the COVID-19 response, but there are gaps in how this data is analysed and utilised within the

For peer review only - http://bmjopen.bmj.com/site/about/guidelines.xhtml

health system. The importance of adequate HIS is in keeping with previous studies.[40,60] A health system's ability to identify and respond to an emerging threat is needed if it is to appropriately meet emerging needs during a rapidly evolving health crisis or shock (principle 7).[40,41] A robust health management information system (HMIS) is crucial to a health systems capacity to respond to shock.[60] Health systems need to have the ability to combine and integrate different forms of knowledge and to anticipate and cope with uncertainties and unplanned events.[40]

COVID-19 has reflected and exacerbated existing social inequalities and emphasised the importance of global collective action, rather than an individual response for genuine resilience. [8] Vaccine inequity and a lack of global solidarity on the part of some richer countries, are dominating the current phase of the pandemic. Our findings seek to highlight opportunity for shared learning across settings in the Global South and North, emphasising the need for a global response to this and future shocks.

Strengths and Limitations

The strengths of this study include the quality of data analysis, which involved a wide range of researchers across both settings, and the breadth of perspectives captured from frontline staff and key decision-makers early in the course of the pandemic. Our study had a number of limitations. Within Merseyside, study participants were selected from across a range of health system levels including primary care, hospital frontline workers and decision-makers, as well as regional decision-makers. By contrast, in Liberia participants included national and county level decision-makers, technicians and supervisors of frontline staff, with no direct frontline workers included. This may result in some of the differences in findings, related to these differing perspectives. Perhaps the greatest limitation of this study is that it was carried out at a single point in time. In Merseyside we collected data towards the end of the first wave, at a time when there were few inpatients and people were reflecting on the first wave. Meanwhile in Liberia it was carried out before there had been a large increase in cases. Since the study was carried out there have been subsequent even greater waves of cases within Merseyside, UK, and Liberia has experienced a large surge in cases of the delta

For peer review only

For peer review only - http://bmjopen.bmj.com/site/about/guidelines.xhtml

variant (59% of cases recorded in Liberia up until 17th July 2021, occurred during a six week period from June 1 2021 to 17th July 2021).[64] By the weeks beginning July 24th to August 7th 2021 number of confirmed cases had declined between zero to 43. Response measures have evolved in both settings, and limitations identified through the study may have been addressed in subsequent stages of the pandemic.

Conclusion

We found the ability of health systems to be able to absorb, adapt and transform in response to the COVID-19 pandemic, in two very different settings, closely relates to the eight FCDO principles of resilience.[16,40] We expanded these principles to include strong structures and mechanisms for advance preparation, and adaptable governance and leadership structures to facilitate timely decision-making and response coordination. At the heart of our findings lies the centrality of the people-centred health system, where the person, is placed within their family, community and the health system.[14] When all aspects work together the outcome is the extent of resilience demonstrated within a health system in response to shock.[40] This includes both the provision of specific services in response to the shock experienced, as well as continued provision of, and demand for, 'routine care'. Our study highlights the need to maintain a people-centred approach for a resilient health system response.

Acknowledgement

We would like to thank all the participants who made time to share their experience and reflections to make this research possible. We recognise and thank Abiola Aiyenigba, who sadly passed away during the study, for her inputs. This research was funded by the NIHR Health Protection Research Unit in Emerging and Zoonotic Infections, and the Centre of Excellence in Infectious Diseases Research, and the Alder Hey Charity. We also acknowledge support of Liverpool Health Partners and the Liverpool-Malawi-Covid-19 Consortium. Additional funding to support this work came from the NIHR REDRESS Programme (NIHR2001129) and FCDO COUNTDOWN (PO6407) programme. We thank Tim

For peer review only

For peer review only - http://bmjopen.bmj.com/site/about/guidelines.xhtml

Martineau, and Joanna Raven (LSTM) for early inputs into study design and Susie Crossman for managing the study budget.

Competing Interests

None declared.

Author Statement

RM prepared the first draft of the paper with inputs from all; Study design, conceptualisation, ethics (ST, LD, MT, LF, IB, ZZ, RM, VW, HP, RAdC, RH, KK); conducted interviews in UK – RM, VW, MT, KO, HP, SC, ST, TEH, RH, RD, YD, OH; conducted interviews in Liberia - ZZ, WT, HB, JK, JSS, CP, GZ, RM. All interviewers participated in the cross-country analysis which was led by YA in the UK with inputs from those who conducted UK interviews and LD, RM, ZZ, HB, WT, JK, JSS, GZ, CP in Liberia. All authors were involved in critical review of the approach, inputted into and approved the final draft of the manuscript.

Figure 1 caption: Conceptual framework

Figure 2: Expanded principles for resilience and people-centred health systems framework

Funding

This research was funded by the NIHR Health Protection Research Unit in Emerging and Zoonotic Infections, and the Centre of Excellence in Infectious Diseases Research, and the Alder Hey Charity. We also acknowledge support of Liverpool Health Partners and the Liverpool-Malawi-Covid-19 Consortium. Additional funding to support this work came from the NIHR REDRESS Programme (NIHR2001129) and FCDO COUNTDOWN (PO6407) programme.

Data availability

Data are available upon reasonable request.

For peer review only

For peer review only - http://bmjopen.bmj.com/site/about/guidelines.xhtml

References

1. Meyer D, Bishai D, Ravi SJ, et al. A checklist to improve health system resilience to infectious disease outbreaks and natural hazards. *BMJ Glob Heal*. 2020;5(8):1–7.

2. World Health Organization. Operational guidance for maintaining essential health services during an outbreak [Internet]. 2020. Available from: https://apps.who.int/iris/bitstream/handle/10665/331561/WHO-2019-nCoV-essential_health_services-2020.1-eng.pdf?sequence=1&isAllowed=y

3. United Nations Economic Commission for Africa. COVID-19 in Africa: Protecting Lives and Economies [Internet]. 2020. Available from: https://repository.uneca.org/bitstream/handle/10855/43756/b11983206.pdf?sequence=1&isAllowed=y

4. Agyeman AA, Laar A, Ofori-Asenso R. Will COVID-19 be a litmus test for post-Ebola sub-Saharan Africa? *J Med Virol*. 2020;92(9):1373–5.

5. Zar HJ, Dawa J, Bueno G, et al. Challenges of COVID-19 in children in low- and middle- income countries. *Paediatr Respir Rev Rev*. 2020;(January).

6. Burki T. The indirect impact of COVID-19 on women. *Lancet Infect Dis* [Internet]. 2020;20(8):904–5. Available from: http://dx.doi.org/10.1016/S1473-3099(20)30568-5

7. Bayani DB, Krubiner C, Barasa E, et al. The Indirect Health Effects of COVID-19: Emerging Findings from Kenya, the Philippines, South Africa, and Uganda. *Cent Glob Dev*. (July 2021).

8. D'Ambruoso L, Abbott P, Binagwaho A. Building back fairer in public health policy requires collective action with and for the most vulnerable in society. *BMJ Glob Heal*. 2021;6(3):8–11.

9. Dzinamarira T, Dzobo M, Chitungo I. COVID-19: A perspective on Africa's capacity and response. *J Med Virol*. 2020;92(11):2465–72.

For peer review only

For peer review only - http://bmjopen.bmj.com/site/about/guidelines.xhtml

10. Das Gupta M, Mansuri G, Sinha N, et al. Overcoming gender-based constraints to utilization of maternal and child health services in Pakistan: the role of the doorstep delivery system. TT - [Internet]. Washington D.C.: [Unpublished] 2007. Presented at the 2007 Annual Meeting of the Population Association of America, New York, New York, March 29-31, 2007.; 2007. Available from: http://paa2007.princeton.edu/download.aspx?submissionId=71659

11. JHU. John Hopkins Coronavirus Resource Center [Internet]. Johns Hopkins University & Medicine. 2021 [cited 2021 Jun 7]. Available from: https://coronavirus.jhu.edu/

12. FPA Reporter. Former Center for Disease Control Boss Hails Liberia for COVID-19 Prevention [Internet]. Front Page Africa. [cited 2021 Jun 7]. Available from: https://frontpageafricaonline.com/news/former-center-for-disease-control-boss-hails-liberia-for-covid-19-prevention/

13. Frieden T. Which Countries Have Responded Best to Covid-19? Wall Street Journal [Internet]. 2021 Jan 1; Available from: https://www.wsj.com/articles/which-countries-have-responded-best-to-covid-19-11609516800

14. World Health Organisation. WHO Global Strategy on people-centred and integrated health services, Interim Report [Internet]. Geneva: World Health Organisation; 2015. Available from: https://apps.who.int/iris/bitstream/handle/10665/155002/WHO_HIS_SDS_2015.6_eng.pdf;jsessionid=B2F4B22FC042ACD2BEF20C5F34803E56?sequence=1

15. Saulnier DD, Blanchet K, Canila C, et al. A health systems resilience research agenda: moving from concept to practice. *BMJ Glob Heal*. 2021;6(8):e006779.

16. Department for International Development [DFID]. Principles of Health Systems Resilience in the Context of Covid-19 Response [Internet]. 2020. Available from: https://assets.publishing.service.gov.uk/media/5ea2b33de90e070498c5538c/Principles_of_Health_Systems_Resilience_in_the_Context_of_COVID_Research_Brief_April_2020.pdf

For peer review only

For peer review only - http://bmjopen.bmj.com/site/about/guidelines.xhtml

17. Van Damme W, Dahake R, Delamou A, et al. The COVID-19 pandemic: Diverse contexts; Different epidemics - How and why? *BMJ Glob Heal*. 2020;5(7):1–16.

18. The World Bank. Liberia poverty headcount ratio [Internet]. 2016. Available from: https://data.worldbank.org/indicator/SI.POV.DDAY?locations=LR

19. UNDP. Human Development Report 2020: The Next Frontier Human Development and the Anthropocene [Internet]. UNDP: New York, NY, USA. 2020. Available from: http://hdr.undp.org/sites/all/themes/hdr_theme/country-notes/LBR.pdf

20. Ling EJ, Larson E, Macauley RJ, et al. Beyond the crisis : did the Ebola epidemic improve resilience of Liberia ' s health system? *Health Policy Plan*. 2017;32:40–7.

21. Ministry of Health and Social Welfare (MoHSW) L. Liberia National Community Health Services Policy.

22. Ministry of Health Liberia. Investment plan for building a resilient health system in Liberia [Internet]. Vol. 12, Monrovia, Ministry of Health. Monrovia, Liberia; 2015. Available from: https://www.resilientinstitutionsafrica.org/sites/default/files/2018-09/%5BLIBERIA%5D Investment Plan for Building a Resilient Health System %282015-2021%29.pdf

23. Liverpool City Council. Liverpool City Council: public health transformation six years on [Internet]. 2019. Available from: https://www.local.gov.uk/liverpool-city-council-public-health-transformation-six-years

24. Statista. Number of coronavirus (COVID-19) cases in the United Kingdom (UK) as of April 5, 2021, by country/region [Internet]. 2020 [cited 2021 Jun 7]. Available from: https://www.statista.com/statistics/1102151/coronavirus-cases-by-region-in-the-uk/

25. Mayor of Liverpool. A STRONG AND GROWING CITY BUILT ON FAIRNESS. 2018.

26. University of Liverpool, Public Health England, Joint Biosecurity Centre, et al. Liverpool Covid-

For peer review only

For peer review only - http://bmjopen.bmj.com/site/about/guidelines.xhtml

SMART Community Testing Pilot [Internet]. Liverpool; 2021. Available from: https://www.liverpool.ac.uk/media/livacuk/research/Mass,testing,evaluation.pdf

27. Sanny JA-N, Bloh O. Liberians laud government ’ s COVID -19 response but claim unfair distribution of relief assistance. *Afrobarometer*. 2021;(420):1–16.

28. Kateh F. How Ebola helped Liberia prepare for covid [Internet]. MIT Technology Review. 2020. Available from: https://www.technologyreview.com/2020/08/19/1007139/liberia-kateh-covid-coronavirus-ebola/

29. Parker C, Zawolo G, Kollie J, et al. PRINCIPLES FOR PROMOTING RESILIENT HEALTH SYSTEMS COVID-19 RESPONSE : LEARNING FROM LIBERIA Principle One : Develop flexible. 2020;

30. Stevens S, Pritchard A. Letter to Chief Executives of all NHS trusts and foundation trusts regarding second phase of NHS response to COVID19 [Internet]. NHS; 2020 p. 1–9. Available from: https://www.england.nhs.uk/coronavirus/wp-content/uploads/sites/52/2020/04/second-phase-of-nhs-response-to-covid-19-letter-to-chief-execs-29-april-2020.pdf

31. Rosenbaum L. Medicine a nd Society The Untold Toll — The Pandemic ’ s Effects on Patients without Covid-19. *N Engl J Med*. 2020;1–4.

32. Pope C, Mays N. Qualitative Research: Reaching the parts other methods cannot reach: an introduction to qualitative methods in health and health services research. *BMJ*. 1995 Jul 1;311:42–5.

33. Kuper A, Reeves S, Levinson W. An introduction to reading and appraising qualitative research. *BMJ Br Med J*. 2008;337:404–9.

34. Gilson L, Barasa E, Nxumalo N, et al. Everyday resilience in district health systems: emerging insights from the front lines in Kenya and South Africa. *BMJ Glob Heal* [Internet]. 2017;2(2):e000224. Available from: http://gh.bmj.com/lookup/doi/10.1136/bmjgh-2016-

For peer review only

For peer review only - http://bmjopen.bmj.com/site/about/guidelines.xhtml

000224

35. Biddle L, Wahedi K, Bozorgmehr K. Health system resilience: a literature review of empirical research. *Health Policy Plan*. 2020;1–26.

36. Sheikh K, George A, Gilson L. People-centred science: strengthening the practice of health policy and systems research. *Heal Res Policy Syst*. 2014;12(1):19.

37. Sheikh K, Ranson MK, Gilson L. Explorations on people centredness in health systems. *Health Policy Plan*. 2014;29(Suppl 2):ii1.

38. Gilson L, Barasa E, Nxumalo N, et al. Everyday resilience in district health systems: Emerging insights from the front lines in Kenya and South Africa. *BMJ Glob Heal*. 2017;2(2):1–15.

39. Newton-lewis T, Witter S, Fortnam M, et al. Working Paper : What is a Shock-Responsive Health System ? *Maint Res Support Soc Serv to Adapt to Shock*. 2020;(June):1–11.

40. Blanchet K, Nam SL, Ramalingam B, et al. Governance and capacity to manage resilience of health systems: Towards a new conceptual framework. *Int J Heal Policy Manag* [Internet]. 2017;6(8):431–5. Available from: http://dx.doi.org/10.15171/ijhpm.2017.36

41. Kruk ME, Ling EJ, Bitton A, et al. Building resilient health systems: A proposal for a resilience index. *BMJ* [Internet]. 2017;357(May):1–8. Available from: http://dx.doi.org/doi:10.1136/bmj.j2323

42. Barasa EW, Cloete K, Gilson L. From bouncing back, to nurturing emergence: Reframing the concept of resilience in health systems strengthening. *Health Policy Plan*. 2017;32(1):iii91–4.

43. Starfield B. Is patient-centered care the same as person-focused care? *Perm J*. 2011;15(2):63.

44. World Health Organization (WHO). The WHO global strategy on people- centred integrated health services services delivery and safety department [Internet]. Service Delivery and Safety. 2015. Available from:

For peer review only

For peer review only - http://bmjopen.bmj.com/site/about/guidelines.xhtml

https://apps.who.int/iris/bitstream/handle/10665/155002/WHO_HIS_SDS_2015.6_eng.pdf?sequence=1&isAllowed=y

45. Saunders B, Sim J, Kingstone T, et al. Saturation in qualitative research: exploring its conceptualization and operationalization. *Qual Quant*. 2018;52(4):1893–907.

46. Fereday J, Muir-Cochrane E. Demonstrating Rigor Using Thematic Analysis: A Hybrid Approach of Inductive and Deductive Coding and Theme Development. *Int J Qual Methods*. 2006;5(1):80–92.

47. Roberts K, Dowell A, Nie JB. Attempting rigour and replicability in thematic analysis of qualitative research data; A case study of codebook development. *BMC Med Res Methodol*. 2019;19(1):1–8.

48. Ritchie J, Lewis J, Nicholls CM, et al. The Foundation of Qualitative Research. *Qual Res Pract A Guid Soc Sci Students Res*. 2013;0–25.

49. Gale NK, Heath G, Cameron E, et al. Using the framework method for the analysis of qualitative data in multi-disciplinary health research. *BMC Med Res Methodol* [Internet]. 2013 Jan [cited 2014 May 1];13(1):117. Available from: http://www.pubmedcentral.nih.gov/articlerender.fcgi?artid=3848812&tool=pmcentrez&rendertype=abstract

50. Alhassan Y, McCollum R, Watson V, et al. PRINCIPLES FOR PROMOTING RESILIENT HEALTH SYSTEMS COVID-19 RESPONSE : [Internet]. 2021 [cited 2021 Jun 7]. Available from: https://www.redressliberia.org/wp-content/uploads/2021/06/Merseyside-Policy-Brief.pdf

51. Akseer N, Kandru G, Keats EC, et al. COVID-19 pandemic and mitigation strategies: Implications for maternal and child health and nutrition. *Am J Clin Nutr*. 2020;112(2):251–6.

52. Rajan S, Chakrabarti D, Akhtar N, et al. Searching for answers: Cancer care during the COVID pandemic. *Clin Epidemiol Glob Heal*. 2021;10(January).

For peer review only

For peer review only - http://bmjopen.bmj.com/site/about/guidelines.xhtml

53. Thorlby R, Fraser C, Gardner T. Non-COVID-19 NHS Care During the Pandemic [Internet]. The Health Foundation. 2020. Available from: https://www.health.org.uk/news-and-comment/charts-and-infographics/non-covid-19-nhs-care-during-the-pandemic

54. Elston JWT, Cartwright C, Ndumbi P, et al. The health impact of the 2014 - 15 Ebola outbreak. *Public Health* [Internet]. 2016;143:60–70. Available from: http://dx.doi.org/10.1016/j.puhe.2016.10.020

55. Golan MS, Jernegan LH, Linkov I. Trends and applications of resilience analytics in supply chain modeling: systematic literature review in the context of the COVID-19 pandemic. *Environ Syst Decis* [Internet]. 2020;40(2):222–43. Available from: https://doi.org/10.1007/s10669-020-09777-w

56. Witter S, Palmer N, Balabanova D, et al. Evidence review of what works for health system strenthening, where and when? 2021;(February):99. Available from: https://rebuildconsortium.com/resources/research-reports/evidence-review-of-what-works-for-health-systems-strengthening-where-and-when/

57. Dean L, Cooper J, Wurie H, et al. Psychological resilience, fragility and the health workforce: Lessons on pandemic preparedness from Liberia and Sierra Leone. *BMJ Glob Heal*. 2020;5(9).

58. Tengbe S. World Mental Health Day 2020 one LSTM student's research on mental health amongst health workers in Sierra Leone [Internet]. Available from: https://www.lstmed.ac.uk/news-events/news/world-mental-health-day-2020-one-lstm-student's-research-on-mental-health-amongst

59. Zhang WR, Wang K, Yin L, et al. Mental Health and Psychosocial Problems of Medical Health Workers during the COVID-19 Epidemic in China. *Psychother Psychosom*. 2020;89(4):242–50.

60. Hanefeld J, Mayhew S, Legido-Quigley H, et al. Towards an understanding of resilience: Responding to health systems shocks. *Health Policy Plan*. 2018;33(3):355–67.

For peer review only

61. Chua AQ, Tan MMJ, Verma M, et al. Health system resilience in managing the COVID-19 pandemic: lessons from Singapore. *BMJ Glob Heal*. 2020;5(9):e003317.

62. Sharma A, Borah S, Moses A. Responses to COVID-19: The role of governance, healthcare infrastructure, and learning from past pandemics. *J Bus Res*. 2021;(January):597–607.

63. UNICEF. Children and AIDS: third stocktaking report. TT - [Internet]. New York, New York, UNICEF, 2008.; 2008. Available from: http://www.unicef.org/devpro/files/CnA_TSR_LoRes_PDF_EN_USLetter_11202008.pdf

64. Ministry of Health Liberia. Liberia COVID-19 Daily Sitrep 510, 7th August 2021. Monrovia, Liberia; 2021.

For peer review only

For peer review only - http://bmjopen.bmj.com/site/about/guidelines.xhtml

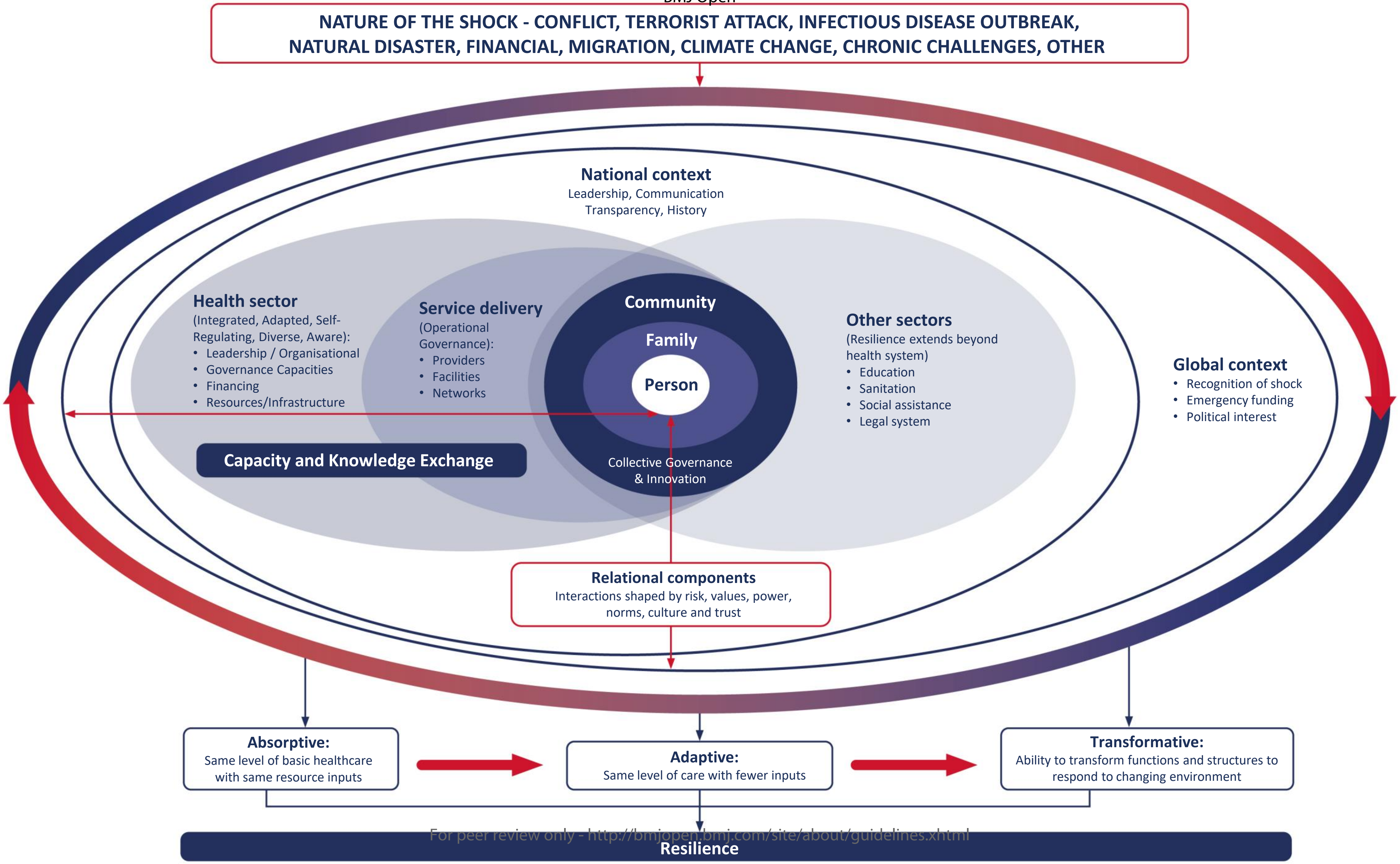

For peer review only - http://bmjopen.bmj.com/site/about/guidelines.xhtml

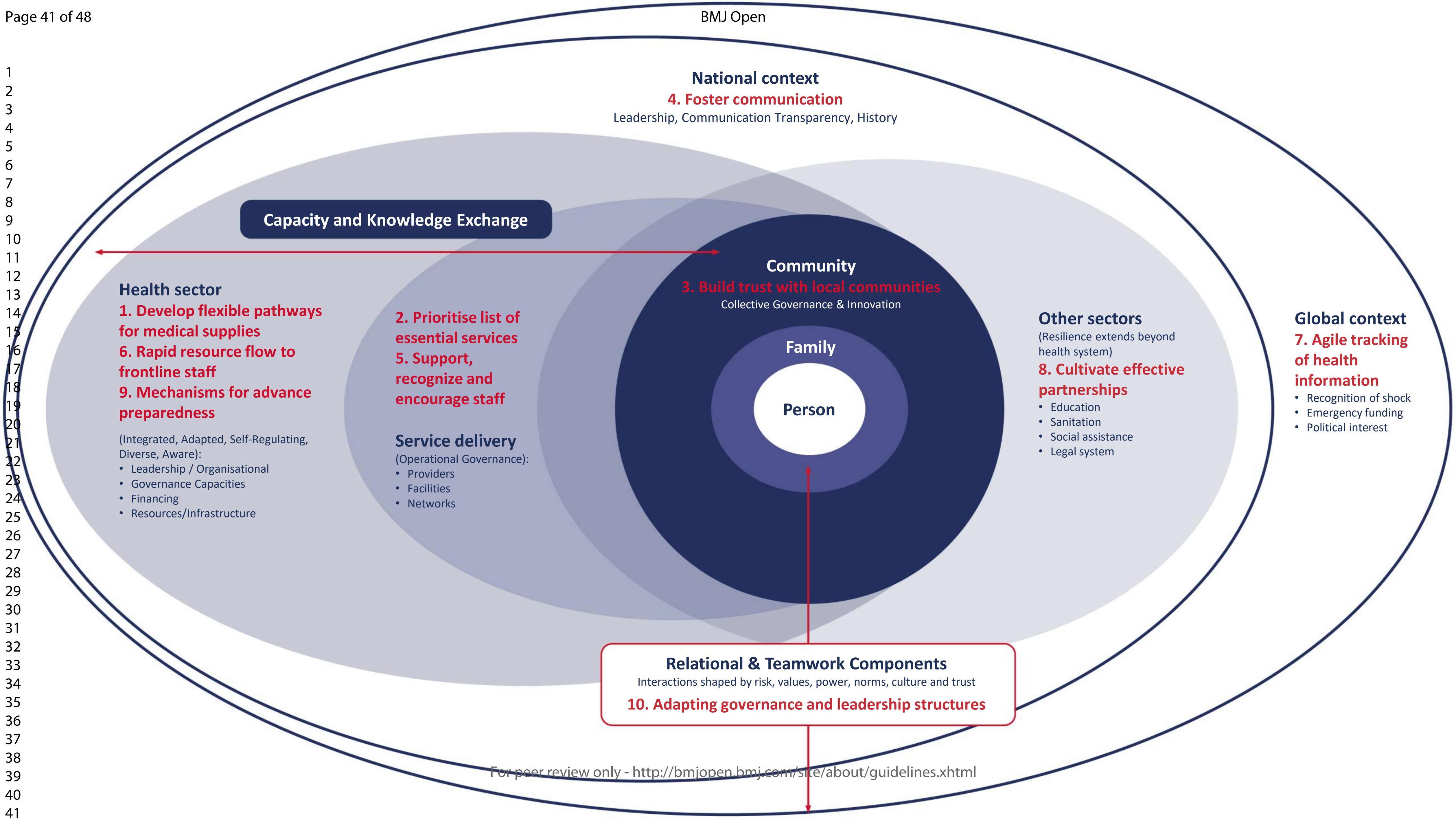

For peer review only - http://bmjopen.bmj.com/site/about/guidelines.xhtml

Appendix 1: COVID-19 Key Informant Interview Topic Guides

Contents

Appendix 1: COVID-19 Key Informant Interview Topic Guides 1

Key Informant Interviews Topic Guide –MOH Liberia 2

Key Informant Interviews Topic Guide –Merseyside Regional Decision Makers 4

Key Informant Interviews Topic Guide – Health Workers 6

Key Informant Interviews Topic Guide –Merseyside Laboratory and Blood Transfusion Staff........ 8

For peer review only

For peer review only - http://bmjopen.bmj.com/site/about/guidelines.xhtml

Key Informant Interviews Topic Guide –MOH Liberia

All possible questions to be asked of key informants are described in the following guide. Prior to interviewing each stakeholder, specific guides for these individuals will be made. One interview that covers relevant research themes will be completed with each stakeholder. Ordering of questions will also be revised to ensure logical flow through the interview and to avoid repetition.

Background

Please can you tell me your position and how long you have worked in your current role?
How has your role changed due to the current COVID-19 crisis?

Responses to Shock and the General Health System

1. How do you think the health system has coped with the COVID-19 crisis? How did it compare with previous crisis? How have routine services been impacted?
2. How is the current shock (COVID-19) the health system is experiencing similar or different to those you have experienced before?
3. What are the key learnings from previous shocks (Ebola/ conflict/ economic crisis)? How are they being used to respond now?
4. How do you think routine health systems functions are being impacted by the current crisis (COVID19/economic)?
5. What do you think could be done to support continuation of routine services? How is this informed or shaped by learnings from during the Ebola period?
    a. How would you describe the quality of services usually? How is quality of care being maintained throughout the COVID-19 response?

6. What policy or guidelines are supporting with the current COVID response? What additional guidelines or policies could be helpful for the COVID response?

Service Specific Impacts

Questions in this section to be reviewed/modified for cross-cutting MOH functions, e.g. M&E, research division prior to starting interview

7. Can you tell me about how service delivery within your programme/section (adapt to include name of section depending on who talking too) has been affected by the COVID pandemic?
    a. Which of your services would you say have been most impacted so far? Why?
    b. Which services would you envisage will be most impacted moving forwards? Why?
8. How have your routine services been modified or adapted? Which components of your service do you view as essential? Why?
9. Which specific sub-populations is routine care most impacted for? Are there any marginalised groups who may struggle to use services since the onset of the COVID-19 crisis? (Probe: e.g gender, dis/ability, rural/urban; wealth; geographic regions; age etc)
10. Have there been any innovations within service delivery in response to the COVID-19 crisis, and have they been useful in any way?
11. Has there been any innovations in response to COVID-19 that have concerned you?

Human Resource Management

12. How have you planned for staffing to meet the changing additional workload in response to COVID? Any tools/ guidance from the human resource section? Successes and challenges? (Prompt for role of new community health cadres, for those providing face to face care and for MOH staff)
13. What additional skill development have you provided and how in response to COVID? Successes and challenges?
14. How are you able to support staff so they can continue to work effectively during the COVID pandemic
    a. How have you supported staff through communication?
    b. How have you supported staff for occupational safety including PPE?

COVID-19 Key Informant Interview Topic Guides

For peer review only

c. How have you supported staff through with psychosocial support?
d. What have been the successes and challenges with supporting staff?

Service and System Impacts: Governance and Decision Making
Questions in this section to be reviewed/modified to make these questions more service-specific, depending on the interviewee's programme area

15. How are decisions made about which services should or should not be prioritised as part of the COVID response? (prompt for in relation to their specific service and also in relation to general health system, prompt for donor influence)
16. How does decision-making as part of the COVID response influence routine planning activities? What has been the impact of resource re-distribution as part of the COVID response?
17. Who is involved in this decision making and what are the processes? What are the challenges?

18. What do you think are the key ethical impacts of making these decisions? What ethical guidelines are currently in place and important in decision making during this period?
19. What guidance documents are available to support you in making decisions regarding COVID?
20. What guidance documents would help to support maintaining routine services?

Closing Questions

21. What does a resilient health system look like to you? What are your three recommendations would you make to improve or maintain the resilience of the Liberian health system during this period?
22. What are your three recommendations would you make post crisis to ensure the return to routine function of the health system as effectively as possible?

Additional questions for Director of personnel only

23. What are the main sources of additional staffing (e.g. secondment/redeployment, task-shifting, improved productivity, early graduation/students, returnees, volunteers)? Successes and challenges? Optional: Impact on the wage bill?
24. What areas of service are now struggling with staffing?
25. What are you able to do to retain staff? Successes and challenges?
26. What impact did/is down-sizing of "non-essential staff' have on your programme during the crisis?

Thank-you
Any other comments?

For peer review only - http://bmjopen.bmj.com/site/about/guidelines.xhtml

Key Informant Interviews Topic Guide –Merseyside Regional Decision Makers

Version1.1_01052020

All possible questions to be asked of key informants are described in the following guide. Prior to interviewing each stakeholder, specific guides for these individuals will be made. One interview that covers relevant research themes will be completed with each stakeholder. Ordering of questions will also be revised to ensure logical flow through the interview and to avoid repetition.

Question List

Background

Please can you tell me your position and how long you have worked in your current role?

Impact of COVID 19 on Routine Service Delivery

1. What are defined as essential routine services?
2. Which are the main scheduled and unscheduled services affected by COVID-19 and how have these been adapted over time?
3. Have there been any innovations within service delivery, and what have these been?
4. Have there been any changes that have concerned you? Why?
5. What would help to support maintaining routine services?

Governance and Decision Making

6. What has informed your decision-making, such as guidance documents or governance decision-making processes?
7. Who is involved in decisions made about which services should or should not be prioritised?
8. How are decisions made about which services should or should not be prioritised?
9. Describe how and who is involved in operationalising decisions?
10. What challenges have you faced in making these decisions?
11. What are the main differences between various sites in the trust, especially between Aintree and the Royal Hospitals?
12. How are changes in service delivery communicated? How can this be improved? There are multiple guidelines at national and local levels, how are these disseminated? How well does this work? How rapidly? How do health care workers respond to these changes?

Human Resource Management

13. How have you [may be the employer in general] planned for staffing to meet the changing additional workload? Any tools/ guidance from national authorities? Successes and challenges?
14. How have you planned for the increase in staff absence?
15. What additional skill development have you provided and how? What have been the successes and challenges?
16. How are you able to support staff so they can continue to work effectively (e.g. communication, occupational safety including PPE, psychosocial support)? What have been the successes and challenges?

Recovery post COVID-19

17. Are there any COVID-19-related changes to routine health services that you think it would be useful to continue after COVID-19? Which ones and why?

18. What next steps do you believe should be taken now to support the health system to recover post COVID-19?

For peer review only - http://bmjopen.bmj.com/site/about/guidelines.xhtml

Thank-you
Do you have any further suggestions for improvements to delivery of routine services?
Any other comments?

For peer review only

COVID-19 Key Informant Interview Topic Guides

Key Informant Interviews Topic Guide – Health Workers

Version1.1_01052020

All possible questions to be asked of key informants are described in the following guide. Prior to interviewing each stakeholder, specific guides for these individuals will be made. One interview that covers relevant research themes will be completed with each stakeholder. Ordering of questions will also be revised to ensure logical flow through the interview and to avoid repetition.

Question List

Background

Please can you tell me your usual position and how long you have worked in that role?

Are you currently working in your usual role and department?

If no, what role and department are you now working in?

Impact of COVID 19 on Routine Essential Service Delivery

1. Can you tell me about how health service delivery has been affected by the COVID pandemic? What was the processes for this, how was it communicated and do you have any ideas about how this can be improved? How prepared did you feel for these?
2. What do you consider to be routine essential health services in your work?
3. Which are the main scheduled and unscheduled services affected by COVID-19 in your department and how have these been adapted over time?
4. What have been the strengths and challenges with these changes? How has quality been affected?
5. How should these changes be evaluated? What indicators should be used?
6. What is worrying you most about your service now?
7. Which services would you envisage will be most impacted moving forwards as the pandemic progresses? (e.g. hospital based, community care, disease specific services, etc) Why?
8. Who do you think are the people most impacted by the changes in routine service delivery? Would you say that patients with specific socio-demographic characteristics are more impacted by service disruption/ distortion than others? Why? (e.g. gender, dis/ability; rural/urban; wealth; geographic regions; age etc) What can be done to ensure that these patients can still use health services when they need them?

Ethics and Decision Making

9. Have you encountered any health systems issues which you found troubling since the start of the COVID-19 pandemic? Would you be willing to tell me more about these issues?
10. What is the impact of these issues on you as a health worker? What would be helpful to support you in dealing with these issues?
11. Do you know of any ethical guidelines in place to guide you as you make difficult decisions during this time? What are these? How are these ethical guidelines operationalised? Are they useful?
12. Have you been involved with making decisions about the changes to health services since the COVID-19 pandemic? What was your role in making these decisions? How were these decisions made?
13. When there are changes in how health services are delivered how are these communicated with you? How has this worked? What do you think is the best way to be informed?

Human Resource Management

14. How has your role changed since the start of the COVID-19 pandemic? What have been the successes and challenges with how your role has changed? Probe workload
15. Is there anything about your role that concerns you? What?
    a. Probe working outside are of expertise
    b. No indemnity if make an error
    c. Communication about working across disciplines

For peer review only - http://bmjopen.bmj.com/site/about/guidelines.xhtml

16. What preparation for the changes to your role have you had and how was it delivered (skills - key ones, psychological support)? What have been the successes and challenges?
    a. Probe PPE training
    b. COVID clinical training
    c. Support mechanisms
    d. Team formation
17. What kind of support (e.g. communication, occupational safety including PPE, psychosocial support) are you receiving to do your job from your team/manager/employer? What have been the successes and challenges?

Recovery post COVID-19

18 . Are there any COVID-19-related changes or innovations to routine health services that you think it would be useful to continue after COVID-19? Which ones and why?

19. What next steps do you believe should be taken now to support the health system to recover post COVID-19?

20. What is worrying you most as the response moves forward?

Thank-you

Do you have any further suggestions for improvements to delivery of routine services?

Any other comments?

For peer review only - http://bmjopen.bmj.com/site/about/guidelines.xhtml

For peer review only

Key Informant Interviews Topic Guide –Merseyside Laboratory and Blood Transfusion Staff

Version1.1_01052020

All possible questions to be asked of key informants are described in the following guide. Prior to interviewing each stakeholder, specific guides for these individuals will be made. One interview that covers relevant research themes will be completed with each stakeholder. Ordering of questions will also be revised to ensure logical flow through the interview and to avoid repetition.

Background

Please can you tell me your position and how long you have worked in your current role?

Governance and Decision Making - Relating Directly to COVID-19

1. What has been the decision-making process for the laboratory's response to COVID-19 testing services and when did discussions start around re-adjusting services for COVID-19?
2. Who held overall responsibility for how COVID-19 testing was going to be conducted at LCL?
3. In addition to PHE, have the Liverpool Clinical Laboratory services worked closely/ collaborated with any other external partners for COVID-19 testing? If so whom and in what capacity?

Governance and Decision Making - Relating to Maintaining Routine Service Delivery

4. How are decisions made about which services should or should not be prioritised; which ones were considered to be essential and why? Who is involved in this decision making? How were these decisions communicated?
5. What guidance documents were most useful to you in making these decisions? In what way were they useful?
6. What key challenges have you faced in making these decisions? Do you have any support needs here?

Impact of COVID-19 on Routine Laboratory Service Delivery

7. Can you tell me about how routine clinical laboratory service delivery has been affected by the COVID pandemic?

COVID-19 Testing service specific

8. How did the laboratories adapt to scale up COVID-19 testing? (analysers, staff capacity, staff training, standard operating procedures, risk assessments)
9. What challenges did the laboratory face when implementing COVID-19 testing? How were they overcome? What worked well? (e.g. resources, human resource, process change, governance, culture, leadership etc)
10. Which routine services would you envisage will be most impacted moving forwards? (e.g. hospital based-testing, disease specific services, etc) Why?

Recovery post COVID-19

11. Are there any COVID-19-related changes to the laboratory service that you think it would be useful to continue after COVID-19? Which ones and why?
12. What next steps do you believe should be taken now to support the laboratory system to recover post COVID-19?
13. Are there any changes/ innovations introduced in response to COVID-19 changes which you think should be continued? Why?

Thank you

Do you have any questions for me? Resources (re labs) link https://www.rcpath.org/uploads/assets/90111431-8aca-4614-b06633d07e2a3dd9/Guidance-and-SOP-COVID-19-Testing-NHS-Laboratories.pdf

For peer review only - http://bmjopen.bmj.com/site/about/guidelines.xhtml

Table 1 Standards for Reporting Qualitative Research (SRQR)

**Standard**	**Page number**
S1 Title	P1, line 1-2
S2 Abstract	P2, line12-35
S3 Problem formulation	P3 line 53-75
S4 Purpose of research question	P4, line 78-124
S5 Qualitative approach and research paradigm	P11 line229-232
S6 Research characteristics and reflexivity	P8 line 165-190
S7 Context	P7 line 129-164
S8 Sampling strategy	P10line200-212
S9 Ethical issues pertaining to human subjects	P11 line 255-262
S10 Data collection methods	P8 line 167-171
S11 Data collection instruments and technologies	P9 line 191-198
S12 Units of study	P10 line219-224
S13 Data processing	P11 line 224-226
S14 Data analysis	P11 line 232-249
S15 Techniques to enhance trustworthiness	P 11 line 226-227
S16 Synthesis and interpretation	P 13 line 267-410
S17 Links to empirical data	P13 line 267-272
S18 Integration with prior work, implications, transferability, and contributions to the field	P21 line 411-523
S19 Limitations	P27 line 524-542
S20 Conflicts of interest	P28 line 565-566
S21 Funding	P28 line558-562

For peer review only
